# Binding‐Site Purification of Actives (B‐SPA) Enables Efficient Large‐Scale Progression of Fragment Hits by Combining Multi‐Step Array Synthesis With HT Crystallography

**DOI:** 10.1002/anie.202424373

**Published:** 2025-03-18

**Authors:** Harold Grosjean, Anthony Aimon, Storm Hassell‐Hart, Warren Thompson, Lizbé Koekemoer, James Bennett, Anthony Bradley, Cameron Anderson, Conor Wild, William J. Bradshaw, Edward A. FitzGerald, Tobias Krojer, Oleg Fedorov, Philip C. Biggin, John Spencer, Frank von Delft

**Affiliations:** ^1^ Diamond Light Source Ltd Harwell Science and Innovation Campus OX11 0QX Didcot UK; ^2^ Structural Bioinformatics and Computational Biochemistry Departement of Biochemistry University of Oxford South Parks Road OX1 3QU Oxford UK; ^3^ Research Complex at Harwell Harwell Science and Innovation Campus OX11 0FA Didcot UK; ^4^ Centre for Medicines Discovery University of Oxford Old Road Campus, Roosevelt Drive OX3 7DQ Headington UK; ^5^ Structural Genomics Consortium University of Oxford Old Road Campus, Roosevelt Drive OX3 7DQ Headington UK; ^6^ Department of Chemistry School of Life Sciences University of Sussex Falmer BN1 9QJ UK; ^7^ Creoptix AG Zugerstrasse 76 8820 Wädenswil Switzerland; ^8^ Sussex Drug Discovery Centre (SDDC) School of Life Sciences University of Sussex Falmer BN1 9QJ UK.; ^9^ Department of Biochemistry University of Johannesburg, Auckland Park 2006 South Africa

**Keywords:** Fragment-based drug discovery, high-throughput x-ray crystallography, automated synthesis, array synthesis, bromodomain inhibitor

## Abstract

Fragment approaches are long‐established in target‐based ligand discovery, yet their full transformative potential lies dormant because progressing the initial weakly binding hits to potency remains a formidable challenge. The only credible progression paradigm involves multiple cycles of costly conventional design‐make‐test‐analyse medicinal chemistry. We propose an alternative approach to fragment elaboration, namely performing large numbers of parallel and diverse automated multiple step reactions, and evaluating the binding of the crude reaction products by high‐throughput protein X‐ray crystallography. We show it is effective and low‐cost to perform, in parallel, large numbers of non‐uniform multi‐step reactions, because, even without compound purification, crystallography provides a high‐quality readout of binding. This can detect low‐level binding of weakly active compounds, which the target binding site extracts directly from crude reaction mixtures. In this proof‐of‐concept study, we have expanded a fragment hit, from a crystal‐based screen of the second bromodomain of pleckstrin homology domain‐interacting protein (PHIP(2)), using array synthesis on low‐cost robotics. We were able to implement 6 independent multi‐step reaction routes of up to 5 steps, attempting the synthesis of 1876 diverse expansions, designs entirely driven by synthetic tractability. The expected product was present in 1108 (59%) crude reaction mixtures, detected by liquid chromatography mass spectrometry (LCMS). 22 individual products were resolved in the crystal structures of crude reaction mixtures added to crystals, providing an initial structure activity relationship map. 19 of these showed binding pose stability, while, through binding instability in the remaining 3 products, we could resolve a stereochemical preference for mixtures containing racemic compounds. One compound showed biochemical potency (IC_50_=34 μM) and affinity (K_d_=50 μM) after resynthesis. This approach therefore lends itself to routine fragment progression, if coupled with algorithmically guided compound and reaction design and new formalisms for data analysis.

## Graphical abstract

“Ne te lave pas, j'arrive.” No work‐up or purification required! Cheap robotics‐based high throughput synthesis of crude reactions mixtures, married to high throughput X‐Ray crystallography, leads to savings in time, energy and solvent use, and was applied to fragment elaboration *vs* the atypical bromodomain PHIP(2). Overall, >1800 reactions were performed in a few weeks and 22 crystal structures were solved showing that binding can be detected on even weak fragments, with 3 showing an unexpected binding pose. A final compound displayed acceptable binding to the bromodomain (IC_50_=34 μM).

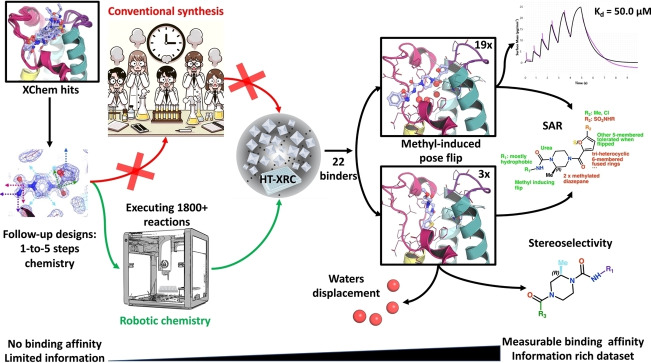

## Introduction

Fragment approaches have established themselves over the last few decades as a powerful approach for interrogating drug targets and discovering new biologically active compounds in a relatively short time.[^1^, ^2^] By the end of 2021 there were 6 fragment‐derived drugs in the clinic, in order of approval: Zelboraf (vemurafenib),^[3]^ Venetoclax (ABT‐199),^[4]^ Erdafitinib,^[5]^ Pexidartinib (PLX2297),^[6]^ Sotorasib (AMG‐510)^[7]^ and Asciminib (ABL001).^[8]^ Two of these moved through the drug discovery pipeline comparatively quickly: Zelboraf progressed from an unselective fragment to a highly selective cancer drug in the span of 6 years and Sotorasib took 8 years from a publication^[9]^ demonstrating the druggability of its target K‐RAS^G12C^ to an FDA approved drug.

Fragment screening is usually performed using biophysical methods or X‐Ray crystallography with rule‐of‐three (Ro3), diversity‐orientated (DOS) or natural product‐inspired libraries that sample a large portion of the accessible chemical space.[^10^, ^11^, ^12^, ^13^] These tend also to be biased towards readily adaptable drug‐like leads.[^14^, ^15^] However, due to their size, fragments seldom display useful affinity, and the initial screen is generally followed by numerous design‐make‐test‐analyse (DMTA) cycles,^[16]^ to progress one or more observed hits to biologically‐relevant potency. Design strategies are commonly classed as fragment linking, merging, or growing, and are ideally supported by advanced computational approaches such as molecular dynamics[^17^, ^18^] or informed “SAR (structure activity relationship) by catalogue.”[^19^, ^20^]

A particular challenge is to first produce compounds with measurable and preferably on‐scale (<50 μM) activity or affinity before common medicinal chemistry design principles become applicable. For this reason, fragment‐based efforts have come to rely on large libraries and careful assay cascades to identify strong fragment hits that can be progressed with confidence.^[21]^ This draws on best‐practice paradigms from High‐Throughput Screening (HTS), where rigorous secondary and even tertiary assays are essential for confirming initial hits.^[22]^

Increasingly, however, fragment hits are being exploited that have no measurable affinity yet are readily identifiable and structurally compelling; these are common where protein crystallography is the primary screen and assay concentrations high, a now accessible technique.[^23^, ^24^] Examples include Resnick et al.^[25]^ where such a fragment was merged with a covalent electrophilic fragment hit to achieve on‐scale potency, and Boby et al.^[26]^ who reported how the merging of two such fragments^[27]^ led to an entire drug discovery effort. Algorithmic approaches have also become available: one approach is to ensure structural motifs observed in the fragments are directly recapitulated in the follow‐up designs.^[28]^ Another uses the pharmacophoric information from multiple fragments to guide docking.^[29]^ Alternatively, interactions can be assessed and prioritised by Dynamic Undocking.^[30]^

Nevertheless, there invariably remains a long journey, both conceptually and experimentally, from merely on‐scale potency to the nanomolar potency required of a lead compound or chemical probe. The ability to cycle DMTA effectively exists within the purview of only expert and well‐funded organisations.^[31]^ A dominant cost driver is the continued requirement for sufficiently purified compound, to minimise confounding assay results.[^32^, ^33^] Certainly, access to such compounds has been transformed over the last decade, thanks to a vastly expanded landscape of commercial vendors and suppliers, who have invested heavily in expertise, technology, building block collections and algorithms, thereby eliminating the need for up‐front laboratory investment by individual discovery efforts, even for synthetically complex targets.^[29]^ Additionally, progress has been made in recent years in developing low‐cost HTS platforms^[34]^ for the synthesis,^[35]^ purification and analysis of compounds.[^32^, ^36^, ^37^, ^38^, ^39^]

However, it appears that the scale of compound exploration must be at least an order of magnitude larger than can be supported by these evolutions on typical early discovery budgets, exemplified by our recent *Covid Moonshot* exercise, necessitating 500 compounds to find one lead series.^[26]^ For instance, Gao et al.^[40]^ have used “on‐the‐fly” nanoscale library syntheses by acoustic dispensing to generate large arrays. Elsewhere, click chemistry,^[41]^ surface plasmon resonance (SPR),^[42]^ NMR,^[43]^ stereo‐dynamic metal probes,^[44]^ mass spectrometry‐based assays^[45]^ as well as direct to biology protocols (D2B) have been used,[^46^, ^47^] with great success, to identify hits from crude reaction mixtures (CRMs). Such approaches not only increase the chances of directly identifying potent compound series, they also generate a critical quantity of data to support robust modelling of SAR.[^48^, ^49^, ^50^]

The key limitation of these approaches, however, is their lack of generality: the synthetic repertoire is strictly curtailed by the constraints of the assay read‐outs, so that they can explore only focused regions of chemical space;^[51]^ only strong assay readouts are informative; and protein‐ligand interactions must be inferred. They are therefore too limiting to generate up front the breadth and depth of data required to achieve accurate potency predictions for diverse compound designs, because extant computational approaches do not generalise at all well.^[52]^

Here, we demonstrate an approach we call *“Binding‐site Purification of Actives”* (B‐SPA), that can indeed, coupled with low cost of synthesis, generate data at both requisite scale and breadth of sampling of chemical space. We have previously shown that CRMs, from a single array of 2‐component single‐step reactions, yield eminently interpretable results of protein‐ligand binding, when using protein crystallography in combination with sensor‐based affinity measurement (surface plasmon resonance, SPR).^[53]^

We now describe a more general synthetic approach, using array chemistry of diverse multi‐step reactions that yield bespoke compounds in CRMs. We exploit the fact that the binding site of the protein will extract the potent species from complex mixtures of compounds, and that the effect can be observed by both biophysics^[42]^ and crystallography,^[54]^ in a highly sensitive manner in the latter case, thanks to recent developments in signal extraction.^[55]^

We previously completed a crystallographic fragment screen (PDB deposition ID: G_1002162) against the second bromodomain of the Pleckstrin Homology Domain‐Interacting Protein (PHIP). This multidomain protein is involved in various cellular processes, including cellular growth and mobility.^[14]^ It has also been implicated in aggressive cancers including BRAF‐negative melanomas, breast, and lung cancer.[^56^, ^57^] However, the specific role of the second bromodomain (hereafter: PHIP(2)) is unknown.

**Figure 1:**
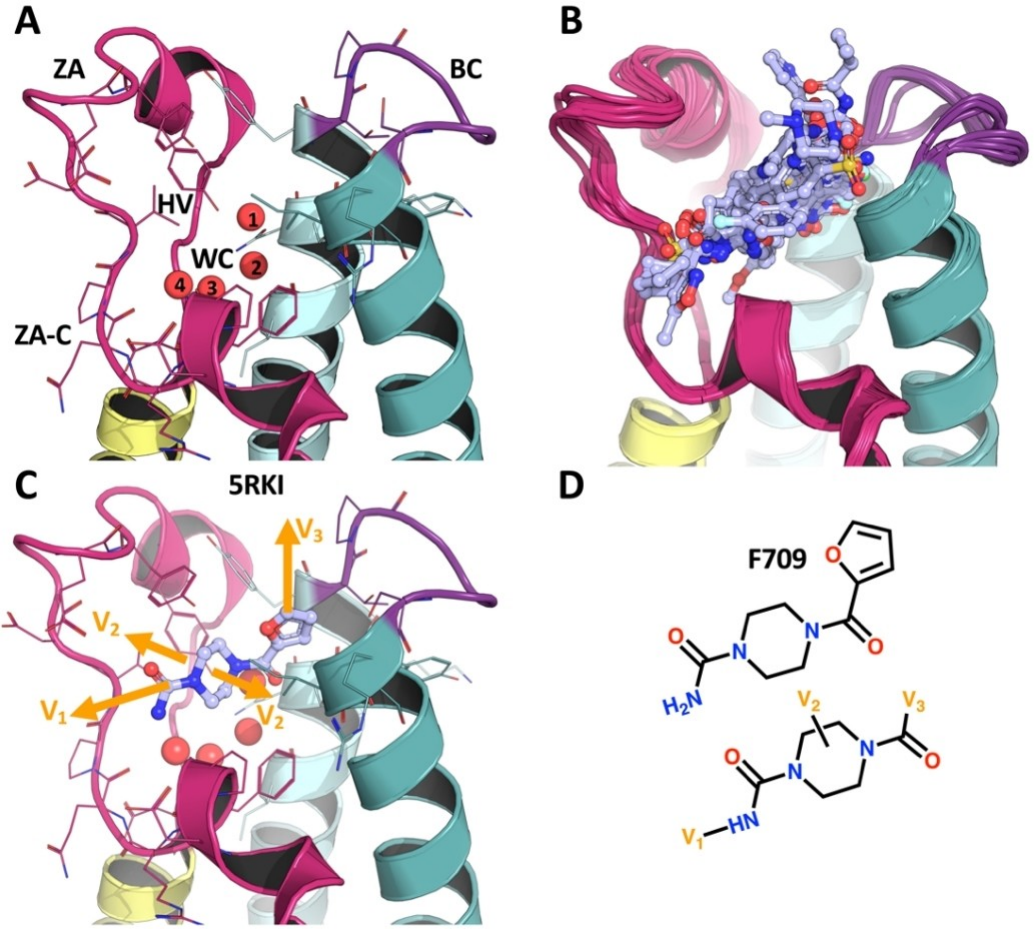
**Crystallographic screening of PHIP(2) identified a fragment with good vectors for automated chemistry elaborations**. Panel A shows the structure of PHIP(2) acetylated‐lysine binding site with the bromodomain waters displayed in red spheres. Helices Z, A, B and C are shown in pink, yellow, cyan and teal, respectively. ZA (in fuchsia) and BC (in purple) indicate the connecting loops between the corresponding α‐helices. ZA−C, WC and HV indicate the ZA‐channel, water cavity and hydrophobic void, respectively. Panel B shows all cocrystals identified by our previous fragment screening. Panel C shows the F709‐bound cocrystal structure (PDB ID: 5RKI) with elaboration vectors in orange. Panel D shows the chemical structure of F709, also with the elaboration vectors V1‐ V3 in orange.

In this study, we used array synthesis to rapidly survey the chemical space growth opportunities around fragment F709 (Figure 1D, Suppl info. 11.1, S180), a piperazine hit from this screen. Synthesis was implemented on a robotics prototype platform based on the low‐cost OpenTrons OT‐1^TM^ liquid handler, with initial quality control (QC) of the CRMs performed by LCMS analysis; all further bottlenecks (*viz*. extraction, solvent evaporation, purification, spectroscopic characterisation) were bypassed by the X‐ray crystallographic analysis of the CRMs.[^2^, ^58^] The workflow enabled the synthesis of a diverse >1000‐member library of compounds in only 2 weeks, with analysis requiring a further 2 weeks, and yielding early‐stage, exploitable SAR derived from 3D structures, as well as a compound with on‐scale biophysical affinity.

## Results and Discussion

### A Previously Identified Fragment Offers Good Opportunities for Robotic Chemistry

**Figure 2:**
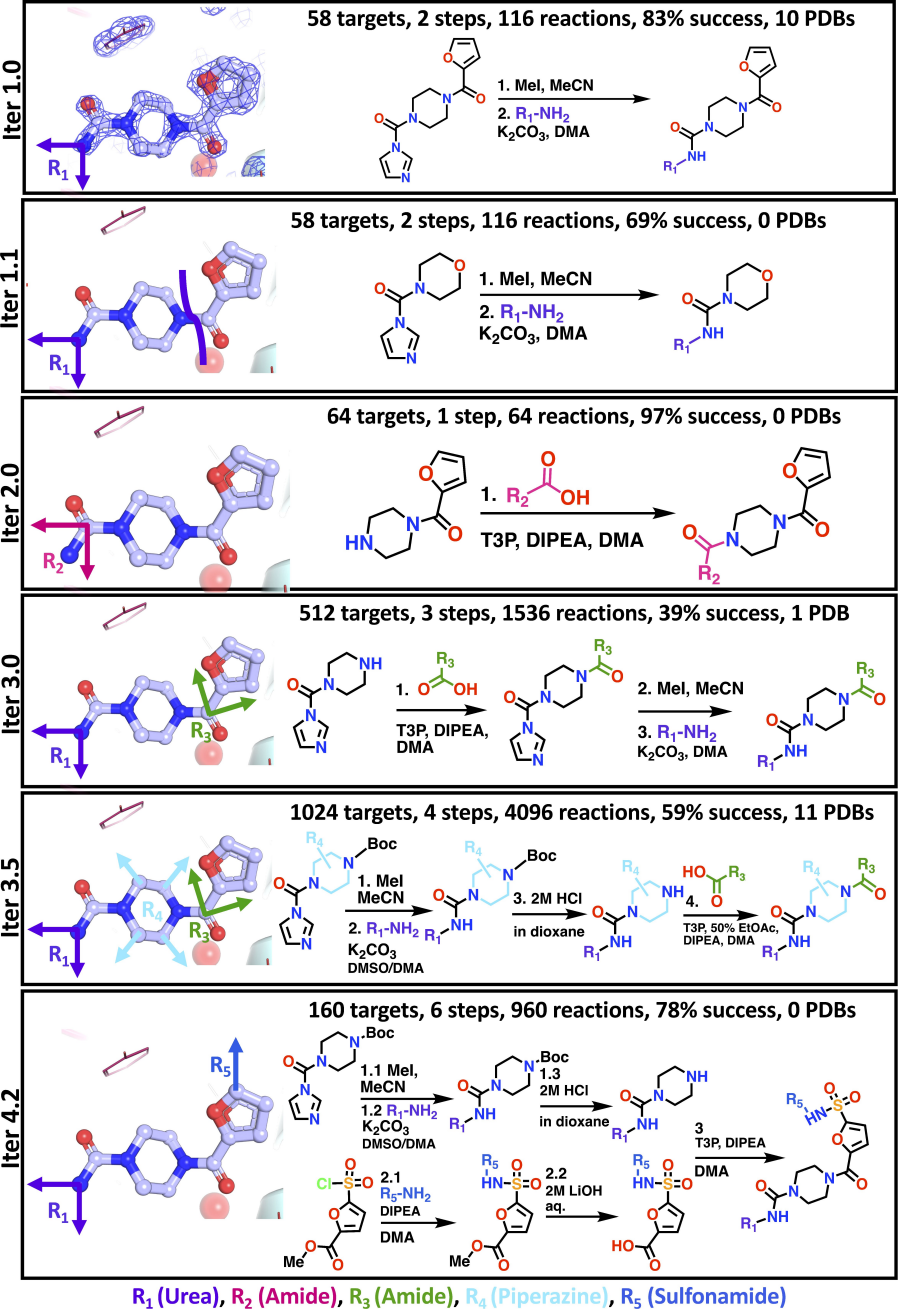
**OpenTrons synthesis achieves reliable synthetic outcomes for diverse growth vectors**. Each panel shows an iteration (”Iter”) of synthetic routes and conditions, as well as (along the top): the number of fragment growth targets, number of steps performed on the robot, total number of reactions, success rates and number of product‐bound 3D protein structures (detailed below). Success rates were calculated as the fraction of CRMs for which the mass expected from the respective product could be detected in the LCMS‐based quality control pipeline. The various expansion vectors are coloured like the respective functional groups listed below the final panel and reactants are oriented to correspond to the original fragment on the left.

Our previous high‐throughput crystallographic fragment screening efforts yielded multiple hits against PHIP(2) (Figure 1A,B & Suppl info. 11.1, S180). These experiments were used in the assembly of the DSiPoised (DSiP) fragment library,^[14]^ and the fragment hits used as the starting point for the SAMPL7 challenge^[59]^ as well as for this automated chemistry study. We selected fragment F709, as it not only had unambiguous electron density at the binding site (Figure 2), but also revealed expansion vectors that could be diversely extended by the reaction repertoire of our robotically‐enabled array chemistry (Figure 1D), and thereby sample the chemical landscape sufficiently to provide initial SAR. Specifically, these vectors enabled fragment growth into 3 different regions of the binding site: (i) across the ZA‐channel; (ii) within the central hydrophobic cavity; and (iii) opposite the water cavity (respectively V1, V2 and V3 on Figure 1C&D). The key interactions of F709 with the protein appear to be: H‐bonds (hydrogen bonds) with the backbone oxygen of proline 1340 and the side chain oxygen of serine 1392; a pi‐pi interaction of the 5‐membered furan ring positioned perpendicularly to tyrosine 1395; and the piperazine ring occupying the central hydrophobic cavity. A detailed view of F709 binding interactions with PHIP(2) is provided in Suppl info. 11.1, S181.

### Robotic Chemistry Enables Batches of Parallel Complex Reactions With Minimal Resources

Several synthetic routes were identified that were simultaneously able to expand into the identified vectors. These relied solely on commercially available building blocks and reactions that were tractable at ambient temperature and pressure (Figure 2). The feasibility of the reactions was initially tested and developed using standard bench chemistry (Suppl info. 2,3,5,6, S9–S39), focusing on solvent selection and solubility. Dimethylacetamide (DMA) proved to be a general solvent and was thus used in all the chemistry developed for and executed on the OpenTrons OT‐1. Thereafter, the reaction conditions were translated into a set of OpenTrons OT‐1 protocols consisting only of steps of liquid transfer by pipette (Suppl info. 7, S40).

We next attempted rapid structure scoping using the OpenTrons OT‐1. The first three iterations were one‐ and two‐step chemistry for urea and amide formation (Figure 2, Iter 1.0, 1.1, 2.0), a total of 180 targets via 296 reactions, with success rates of 83%, 69% and 97% respectively. Success was defined as the identification of the expected molecular ion peak in the LCMS spectrum of the CRM.

The subsequent three iterations were more complex multi‐step reaction routes, aiming for a total of 1696 multi‐step targets, using a combined total of 6592 reactions; this achieved success rates of 39%, 59% and 78% (Figure 2, Iter 3.0, 3.5, 4.2). The most complex synthesis, Iter 4.2, combined the products of two parallel two‐step routes for a total of 160 attempted products.

The time, effort, solvent and space requirements were all strikingly low: we estimate reductions of almost 3 orders of magnitude over performing the equivalent experiment by conventional medicinal chemistry (Table 1), based on the manual synthesis and purification of control compounds (Suppl info. 6, S37). These savings arise almost entirely from the absence of large‐scale aqueous workups and column purifications, which our method circumvents.

**Table 1:** **The automated chemistry workflow reduces time and solvent usage compared to human operations**. Comparison of the estimated time, columns/work‐ups, and litres of solvent, between automated and manual synthetic approaches to the target libraries. (Red=Manual synthesis, Green=Robotic Synthesis).

		Time		Columns and workups		Solvent volume	
Iteration	Number targets	Per target (days)	Total (days)	Per target	Total	Per target (mL)	Total (L)
**1**	58	**3** / –	**174** >3	**1**	**58** >1	**1000** >5	**58** >0.29
**1.1**	58	**3** / –	**174** >3	**1**	**58** >1	**1000** >5	**58** >0.29
**2**	64	**1** / –	**64** >2	**1**	**64** >1	**1000** >5	**64** >0.32
**3**	512	**5** / –	**2560** >4	**2**	**1024** >2	**2500** >10	**1280** >5.12
**3.5**	1024	**5** / –	**5120** >4	**2**	**2048** >2	**2500** >10	**2560** >10.3
**4.2**	160	**8** / –	**1280** >4	**3**	**480** >3	**4500** >15	**720** >2.4
**Total targets**	**1876**	**Total Time**	**25.68y** **>** **20d**	**Total** **columns** **& workups**	**3732** **≫** **10**	**Total solvent volume (L)**	**4740** **≫** **18.70**
*Saving*		*Fraction Time*	***0.2%***	*Fraction*	***0.3%***	*Fraction* *solvent*	***0.4%***

Specifically, we estimate 1–8 working days per analogue by conventional methods, depending on the number of synthetic steps and purifications. This equates to 25 years of linear synthesis for the complete library, although self‐evidently significant time reductions can be achieved by techniques such as parallel synthesis (see below) and bulk preparation of common intermediates. A classical approach would necessitate up to 3732 chromatography stages, and combined with the work‐up stages, this would result in a total solvent usage of 4740 L. In contrast, the automated protocol requires 5–15 mL of solvent per analogue, a total of 18.7 L and a more than 250‐fold reduction in solvent usage.

Finally, the high‐throughput robotics set‐up has a small synthetic footprint and is readily contained in a single fumehood, and performs all steps by automated protocol: stock solution preparation, reaction set‐up/running, work‐up, final sample solution preparation, and QC sample preparation. In contrast, a classical synthetic approach would require substantial equipment, take up substantially more space, and continual operation by the project team of multiple chemists.

For completeness, there are several complementary techniques worthy of mention, representative examples of which are outlined hereafter. DNA‐encoded libraries (DELs) generate millions of CRMS for direct analysis, although conditions and reaction compatibility can be limited (aqueous mixtures, pH, temperature).[^60^, ^61^] A mass spectrometry‐triggered purification assay cascade yielded *ca*. 30‐member pure compound libraries in under 30 h, moreover with sufficient material to enable secondary investigations.^[62]^ A library (>500 reactions achieved in 3 months) of broad‐spectrum antivirals was synthesised using an automated parallel synthesiser, coupled with solid phase extraction (SPE), yielding nanomolar‐active lead molecules.^[62]^ Others have generated large amounts of data *via* photochemical reaction arrays coupled with automatic purification[^63^, ^64^] or through miniaturized high‐throughput experimentation (HTE).^[65]^

### High‐Throughput LCMS Combined With Algorithmic Peak‐Finding and m/z‐Matching Provide Rapid and Sufficient Quality Control

Conventional quality control (QC) of the 1876 attempted targets required seventeen days of manual analysis using the vendor‐supplied graphical user interface (GUI) with most time spent assessing the post‐work‐up presence of product (Table 2). The goal was to annotate each CRM with a qualitative *yes/no* success label, justifying the presence of sufficient product for the post‐structural analysis. The procedure entailed first locating peaks in the Total Ion Chromatogram (TIC) of the CRMs, and then scanning each mass spectrum associated with the respective TIC peaks, for significant ion signals that matched the expected M+ mass.

**Table 2:** **MSCheck enables significant time gains for quality control with a good recall against human operations**. Summary of reaction LCMS samples run, and comparison of MSCheck with the human‐analysis for the six iterations of the chemistry run.

Iteration	Number of LCMS samples	MSCheck match with human (%)	Review of 17% MSCheck mismatches					
			Number of MSCheck true positives	Number of MSCheck false negatives	Number of MSCheck false positives	MSCheck true positives & negatives (%)	MSCheck false negatives (%)	MSCheck false positives (%)
1	58	81	4	7	0	88	12	0
1.1	58	–*	–*	–*	–*	–*	–*	–*
2	64	96.87	0	2	0	97	2	0
3	512^≠^	78.1	66	12	20	93	3	4
3.5	1024	74.4	172	30	55	91	3	5
4.2	160	84.4	1	16	8	85	10	5
**Total**	**1876**	**83^†^**	**243**	**67**	**83**	**91^†^**	**6^†^**	**3^†^**
*Analysis time (days)*	*17* ^*≠*^	*5*						

*Not completed. ≠ 448 samples analysed by MSCheck. † Average of percentages. ≠ Manual analysis

Automated LCMS analysis tools have been published that span parsing of vendor report‐files (such as PyParse) through to deep learning frameworks that predict structures from mass spectra.[^66^, ^67^] However, for the manual analysis required for CRM screening, a quality control tool was required that combined automation with capturing of final manual annotations. *MSCheck* was therefore developed to perform automated TIC peak finding using Scipy′s established *find peaks* function, which it follows up with an ion signal matching search.^[68]^ It is agnostic of the vendor reports, using the open mass spectrometry .mzML file format. ^[69]^

Retrospective evaluation of iterations 1.0, 2.0, 3.0, 3.5 and 4.2 final showed that *MSCheck* matched with 83% of the human‐analysis of synthesis outcomes that included true positive and true negative reaction successes (Table 2). Review of the 17% (383 samples) mismatches between human analysis and *MSCheck*, found that it identified 243 additional true‐positive reaction successes, with the balance split between: *MSCheck* not locating the ion signal match (false negatives, 67 samples); the ion signal not being the most prominent in the mass spectrum; and ion signals picked from the noise (false positive, 83 samples) (Table 2). These examples are also highlighted in Suppl info (1.7.1, S5). *MSCheck* thus correctly identified 91% of the true positive and true negative reaction successes; 6% were false negatives due to ion count signals not being the most prominent; and 3% were false positives due to matching ion signals in noise. *MSCheck* took five days to complete: most of the time was spent on data preparation and script preparation, and only several minutes of run time, all without the onerous task of constantly staring at a screen, scanning for mass matches (Table 2).

### X‐Ray Crystallography of CRMs Can be Performed at High‐Throughput Levels

High‐throughput X‐ray crystallography requires that protein crystals diffract at a good resolution and resist fracture during soaking. Previous work demonstrated that CRMs can be prepared and soaked onto protein crystals and that reaction products can be resolved by X‐ray diffraction.^[2]^ Here, CRM preparation was also included in our robotic framework where the products were concentrated in the organic phase and solvent exchanged. Due to the intrinsic affinity of bromodomains for DMSO (dimethyl sulfoxide) and/ or DMA, these solvents were replaced with ethylene glycol.^[70]^

**Figure 3:**
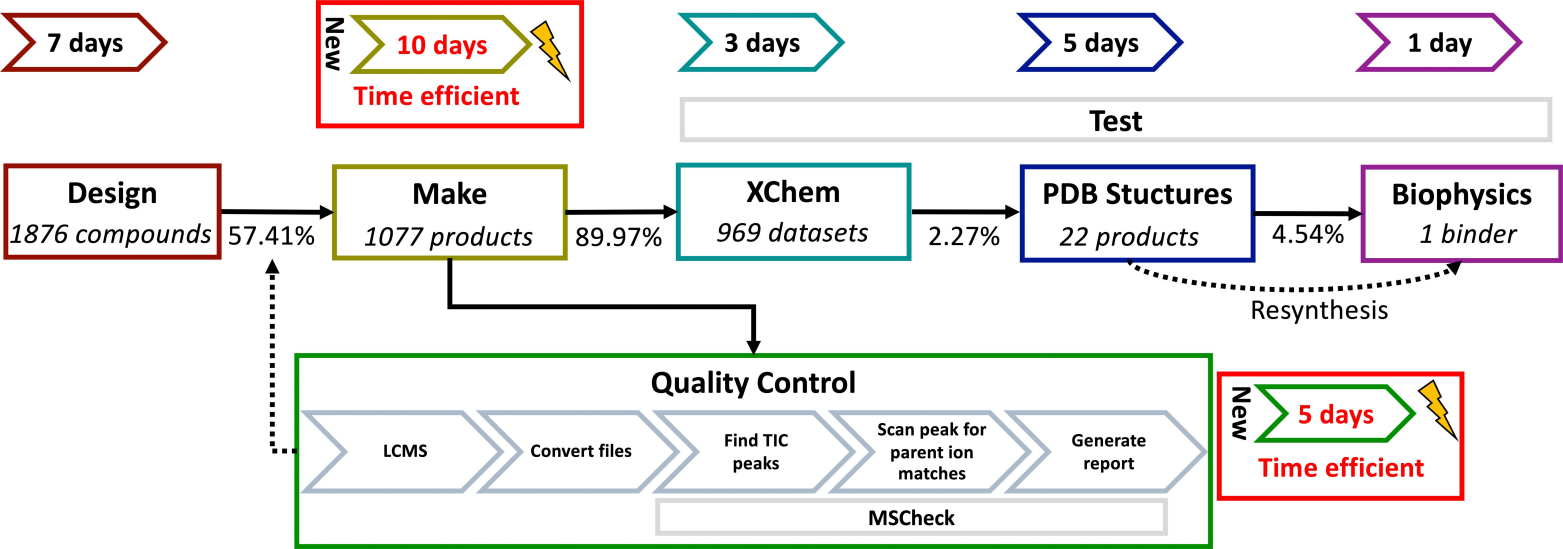
**Automated chemistry and QC fit with the XChem workflow and reduces human labour**. Each step of the process is represented in a coloured box. The molecular and reaction designs were made with RDKit. Preparation of CRMs performed on an OpenTrons OT‐1; CRM QC via LC–MS and analysed with MSCheck. Crystals were prepared and resolved at XChem, product hits were identified from electron density maps with Coot and PanDDahits were confirmed via Creoptix and an alpha‐screening assay. The quality control step is parallel to the main workflow because crystals were soaked with all CRMs while the LC–MS outcome determined the number of successful reactions, as indicated by the dotted arrow. Workflow steps where significant resources were saved are indicated with a red box and a lightening logo. The validation required ordering the pure compounds from Enamine or resynthesis in house. Success rates are shown above the arrow connecting the steps and are indicative of the number of successful outcomes for a given step over the number of successful outcomes of the previous step.

Desired products were detected in 1077 out of 1876 syntheses by our LCMS quality control protocol, representing a 57% success rate across all iterations. CRMs were soaked onto protein crystals at the XChem fragment screening facility at Diamond Light Source (Figure 3), yielding 969 usable X‐ray diffraction datasets for the 1077 CRMs with successful syntheses. This translates into a crystal deterioration rate, upon soaking of CRMs, of only 8.7% (Figure 3), demonstrating excellent compatibility of this streamlined downstream workflow with the pre‐existing high‐throughput crystallographic pipeline. Therefore, we were able to generate large volumes of high‐quality diffraction data in a relatively small amount of time while maintaining crystal integrity. Furthermore, the quality control step supplements the workflow by allowing reaction outcomes to facilitate the tracking of which electron density maps could be expected to have bound reaction product.

Here, we achieve a 22‐fold gain in processing compounds, or 7‐fold gain in performing experiments, compared to Baker et al., who obtained more product‐bound structures, from 83 reaction mixtures in triplicate. Instead, while our one‐shot protocol is likely to have missed some binders,^[2]^ our aim was to increase the number of synthesised follow‐ups using cheap robotics whilst minimising bottlenecks. Although larger arrays are conceivable through our streamlined approach,^[40]^ we kept the size of the iterations at levels that are manageable for downstream XChem processing, which involves human interventions at certain steps, such as crystal fishing.

**Figure 4:**
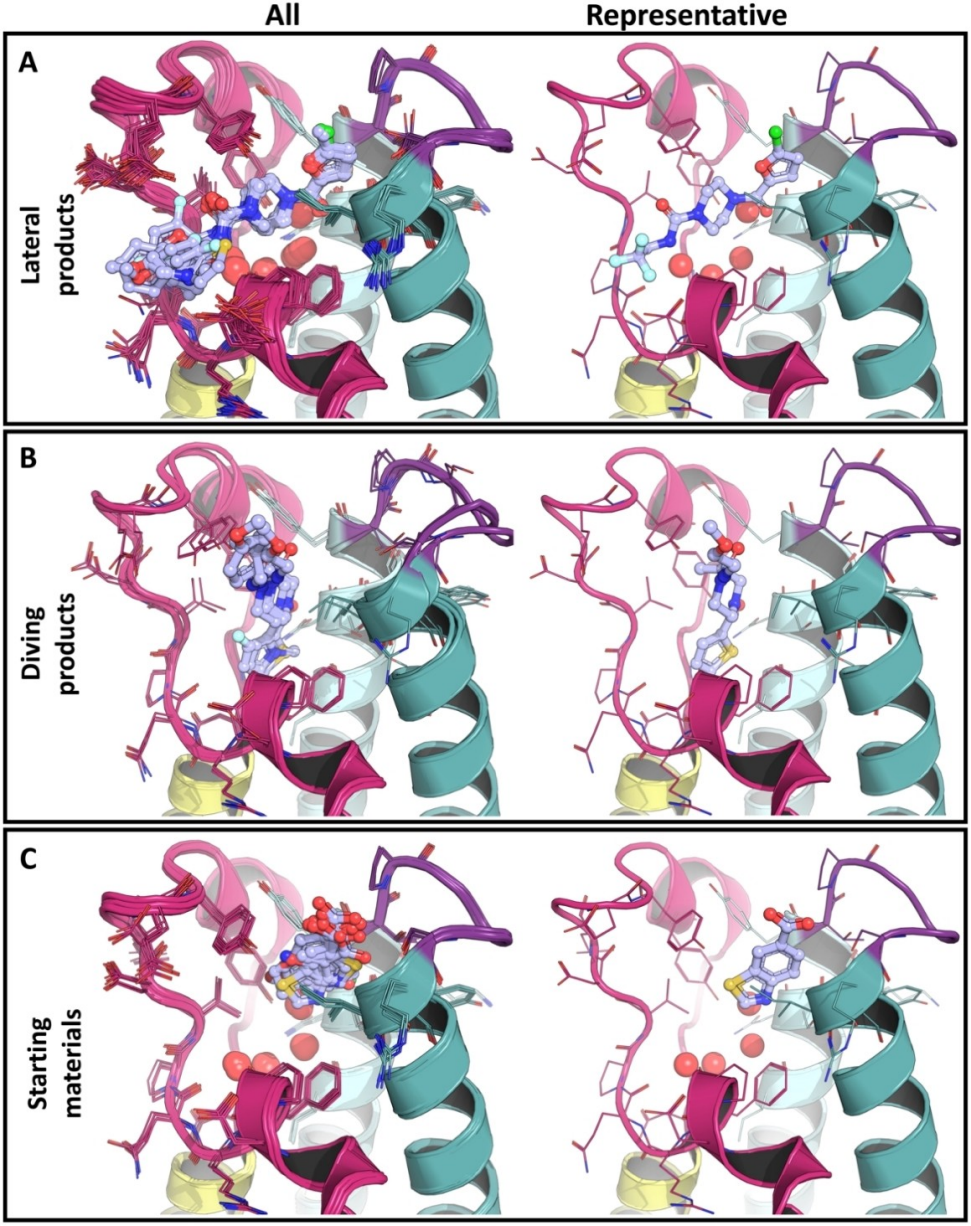
**XChem screening of CRMs yielded bound product structures, with conserved and non‐conserved binding poses, and starting materials**. The first and second panels show structures of the laterally (A) and diving (B) bound products, respectively. The third panel shows structures of the reaction starting material‐bound (C) proteins. Each panel is subdivided into 2 columns. The first (All) aggregates all bound structures while the second (representative) show a single binder that has a binding mode representative of the others. The representative binders were arbitrarily selected to illustrate the corresponding binding mode.

Our crystallographic experiments resolved a total of 29 unique compound‐bound structures, which included 7 starting materials and 22 reaction products (Figure 4). Considering target‐bound reaction products from successful syntheses and usable X‐ray diffraction data after soaking, we achieved a hit rate of 2.3% (22/969) (Figure 3). This is a lower hit rate than the original crystallographic XChem fragment screen against this crystal form, which yielded a 6.5% hit rate with similar soaking conditions,^[59]^ but we anticipate that improved compound library designs, coupled with biological assay feedback loops, will dramatically increase hit rates in future experiments.

### Structural Analysis Rationalises the Binding Landscape and Resolves an Unexpected Pose

19 of the unique products observed in crystals (Figure 4) have a conserved binding mode relative to the starting fragment, here referred to as the *lateral pose*. These offer rapid access to significant constructive “green light” and fail‐fast/fail‐cheap “red light” SAR. Iter 1.0, the exploration of the urea vector, was the only single‐step iteration to yield follow‐up crystal hits (10) (Figure 2). The importance of the furan ring for binding was highlighted because no structural hits were found for Iter 1.1’s urea vector exploration in its absence (Figure 2). Iter 2.0 confirmed the importance of retaining the urea group of the initial fragments; no hits were found unless it was retained, presumably because it forms a hydrogen bond with the proline 1340 backbone oxygen (Figure 4 **, S**uppl info. 11.2, S181).

Unexpectedly, 3 products were resolved in an alternative orientation, termed the *diving pose*, relative to the original fragment F709 as shown in Figure 4 (and Suppl info. 11.1, S180), demonstrating the effect of modifying the piperazine ring in Iter 3.5. The compound names and associated PDB codes are available in Suppl info. 10, S179. This pose is rotated by about 90° around the piperazine core with respect to the original pose, and remarkably, the 5‐membered aromatic ring displaces waters 2 to 4 while the nearby amide displaces water 1 of the water network (Figure 1). The aromatic 5‐membered rings seem to bind mostly via hydrophobic interactions within the water cavity. In addition, the *cis‐*orientation of the two carbonyl oxygens was observed in the diving products whereas the lateral products displayed a *trans‐*orientation. A significant change in protein conformation is revealed by this novel diving binding pose: the ZA‐loop adopts a more relaxed conformation, resulting in a more voluminous binding site that accommodates the flipped products, thus illustrating that some level of conformational motion is present in our crystal system (Figure 4).

All starting materials bind at the same location between helices B and C (Figure 1) and display a conserved binding mode where they interact with the protein via two pi‐pi stackings and hydrogen bonding (Figure 4, Suppl info. 11.3, S183). All product‐bound cocrystals maintain a 4‐formylpiperazine‐1‐carboxamide scaffold, where the piperazine moiety occupies the central hydrophobic cavity. Poses for the products also retained the original furan or the analogous thiophene or pyrrole rings, with some hits having either a 2‐chloro‐or methyl furan.

Detailed views of the lateral, diving and starting material binding interactions with PHIP(2) are shown in Suppl info. 11, S180 onwards.

**Figure 5:**
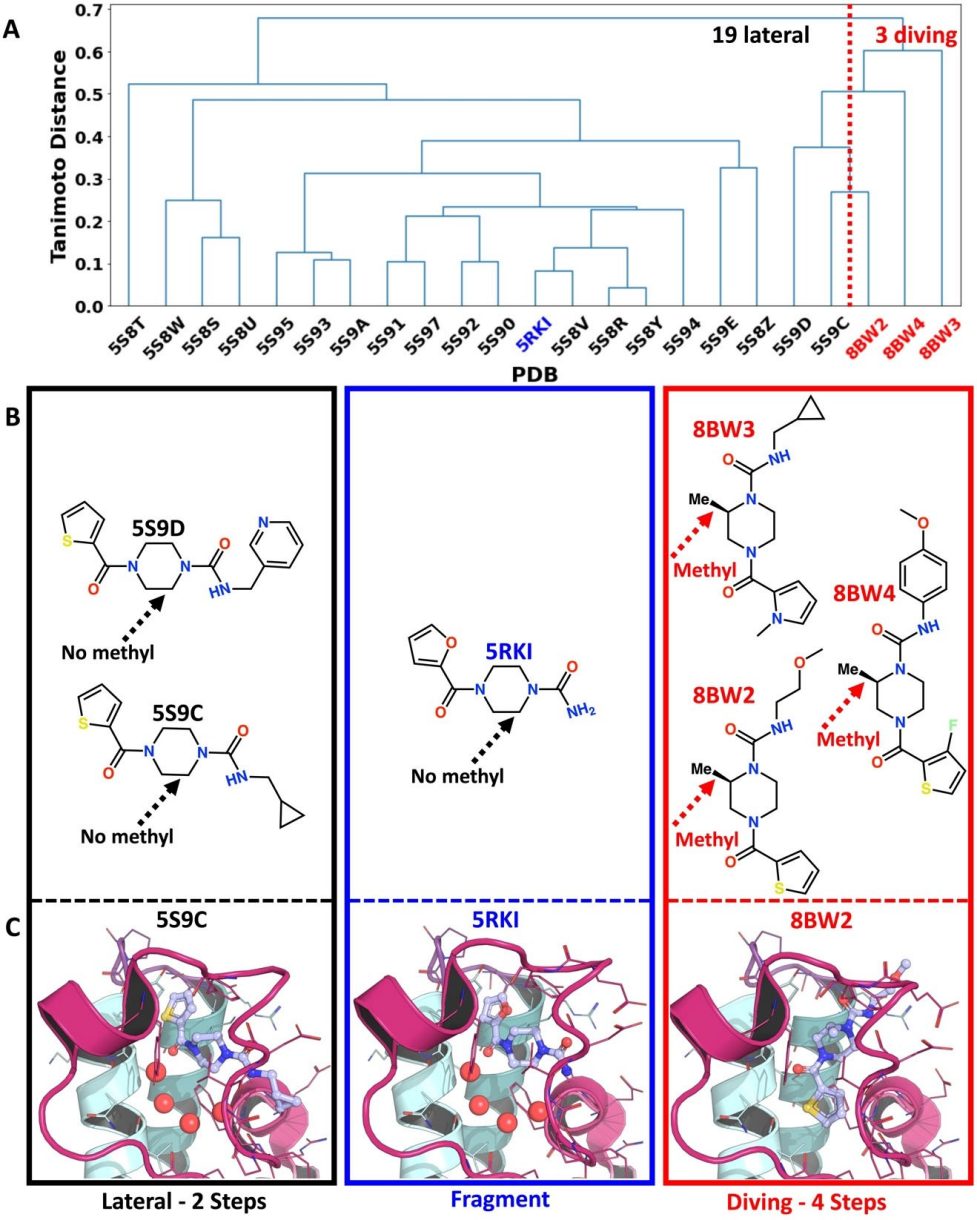
**Unexpected diving binding pose appears to be triggered by the addition of a methyl to the core piperazine ring**. A dendrogram showing the chemical fingerprint (Tanimoto) similarity between bound reaction products is shown on top (A). The compounds are labelled by PDB accession IDs. Information related to starting fragment, lateral and diving compounds is highlighted in blue, black and red respectively. The 2D structures for the fragment (B), the divers′ closest neighbour (laterals) and divers with a representative 3D structure (C) are shown in the first, second and third columns, respectively. The number of synthetic steps required to obtain those compounds is shown under the columns for lateral and diving bound reaction products.

A striking feature of the diving products is that they all have a methyl‐substituted piperazine ring. When looking at their closest lateral neighbours based on chemical similarity distance, it seems that this alkyl substitution is required to change binding orientation (Figure 5).

**Figure 6:**
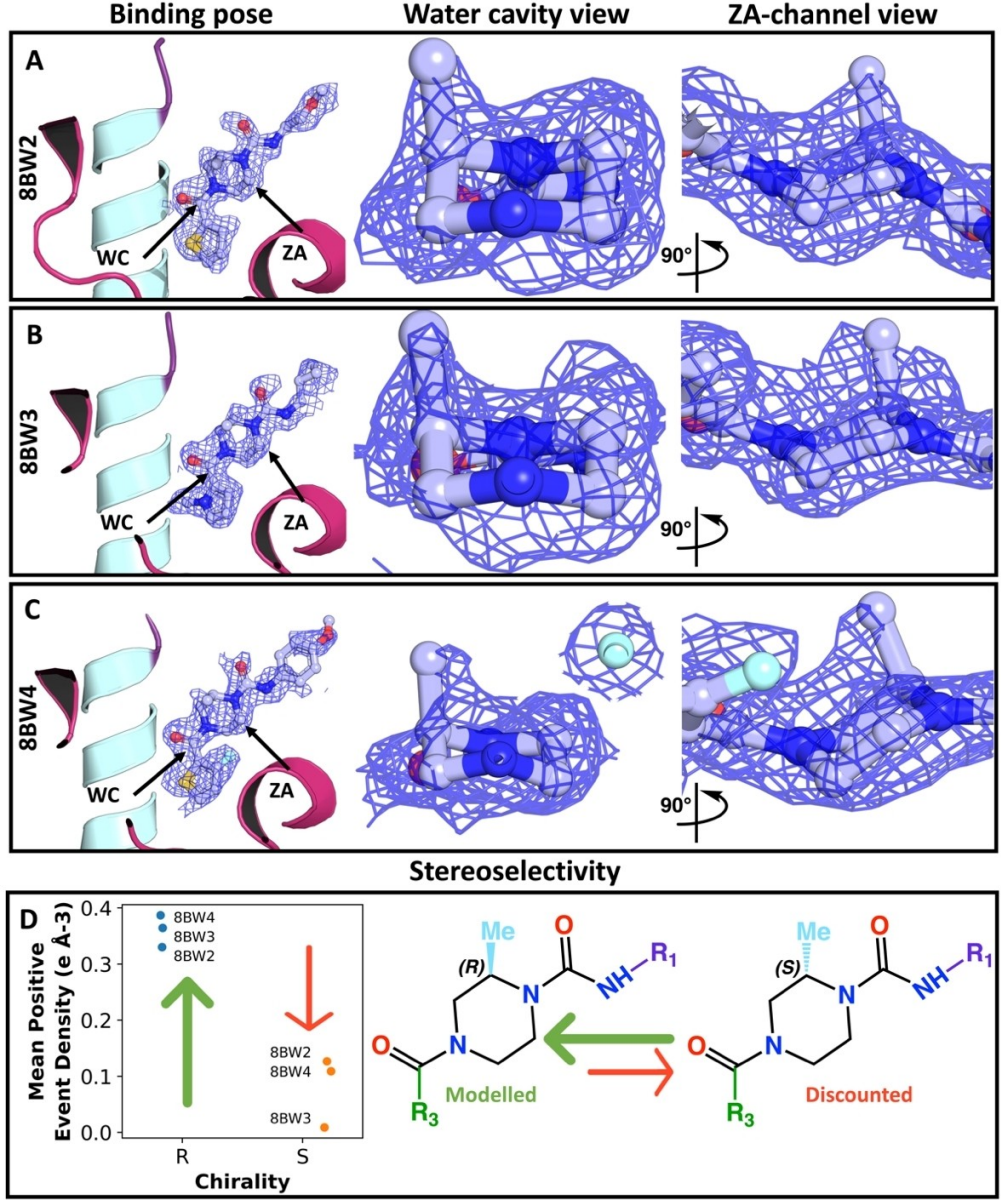
**Stereochemistry can be conclusively assigned from even low‐confidence electron density by combining similar compounds**. Panels A to C show the PanDDA event maps for the divers. The first column, labelled as the binding pose, presents the overall event density for binding. The second column shows the density of the methylpiperazine moiety as observed from the water cavity (WC). The third column depicts the same moiety′s density from the ZA‐channel (ZA) perspective, with viewpoints marked by arrows in the binding pose column. Panel D demonstrates the two potential chiralities, with the green arrow specifying the chirality modelled in the binding site. Panel D also illustrates the mean positive density values computed along the carbon‐carbon bond of piperazine‐methyl. These values are aligned with the PanDDA event map for both (R)‐ and (S)‐ enantiomers after fitting and refinement, thus supporting the final modelled structure.

The synthesis of the three diving products made use of racemic building blocks (Figure 2), resulting in CRMs containing a mixture of enantiomers, and the quality control protocol identified that products with the expected mass were present in the mixture. PanDDA event maps, instead of traditional electron density maps, were used to fit the compounds. The position of the methyl group around the piperazine ring was ambiguous and required further inspection (Figure 6) because its stereochemistry was unknown.

These maps showed a consistent protrusion at the same location, suggesting the presence of an additional group (Figure 6). Other possible methyl locations do not have a similar protrusion and positioning the group there would result in a clash with the protein and/or the compound itself. At the identified position, the methyl group interacts with the binding site through hydrophobic interactions (Figure 4, Suppl info. 11.1, S180).

Individually, none of the diving products could be unambiguously assigned to either *(R)‐* or (S)‐ enantiomer: all relevant variations in electron density were no higher than the standard deviation observed in the immediate vicinity of the protein model. In contrast, taken together, the electron densities for the three repeat observations offered consistent support for the *(R)‐* enantiomer, revealed by a paired sample T‐test at the 1% level (Figure 6D).

One driver for the pose change might be entropic gain via water displacement.^[71]^ Indeed, previous modelling efforts suggested the PHIP(2) water network to be relatively unstable compared with other bromodomains, with a positive Gibbs binding free energy.^[72]^ Moreover, the presence of a “magic methyl” group can significantly improve binding affinity in drug discovery.^[73]^ Overall, these results indicate that the protein binding site defined by this crystal system is favourable for the methyl group oriented in an “up” (*R*)‐fashion and located on the same side as the compound′s *cis‐*oriented carbonyl oxygens (Figure 6). This approach thus directly generates the statistical power required to unambiguously assign the stereoselectivity, that would otherwise have required costly resynthesis and the extensive delays of repeat experiments.

**Figure 7:**
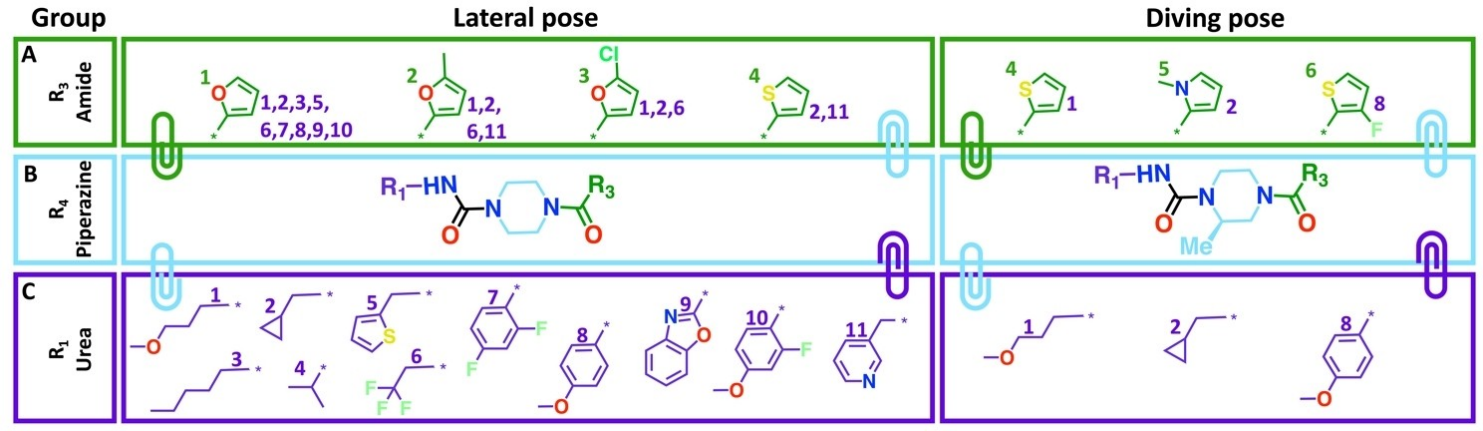
**Distinct combintations of R‐groups lead to crystallographic binding and changes in binding pose**. The different groups causing crystal binding for each pose are shown in the panels. The numbers next to the group for the amide (A) and urea expansions (C) highlight the observed combination, around the central piperazine scaffold (B), between those groups.

Overall, the crystallographic results identify which vectors can be successfully exploited, thereby providing key crystallographic SAR information around fragment F709 (Figure 7). Several modifications with confirmed successful synthesis consistently failed to show up as crystallography binding events, including: replacing the piperazine a with diazepane or dimethyl‐piperazine moiety; replacing the furan with a 6‐membered ring or tri‐heterocyclic ring; and the use a sulphonamide functional group on furan, in Iter 4.2 (Figure 2). Some of these may be false negatives, as soaking of CRMs, and high‐throughput methodologies in general, maximises data quantity over quality; yet it was the thorough sampling of the chemical space that revealed the change of binding pose paired with protein conformational changes, and that would not have been identified by classical or guided docking methods.

Such chemical exploration would not have been possible without the use of multi‐step chemistry, thus highlighting the importance of streamlined automated arrays to rapidly access more elaborated chemical space.

### Crystallographic Hit Exhibits On‐Scale Biochemically and Biophysically Binding

**Figure 8:**
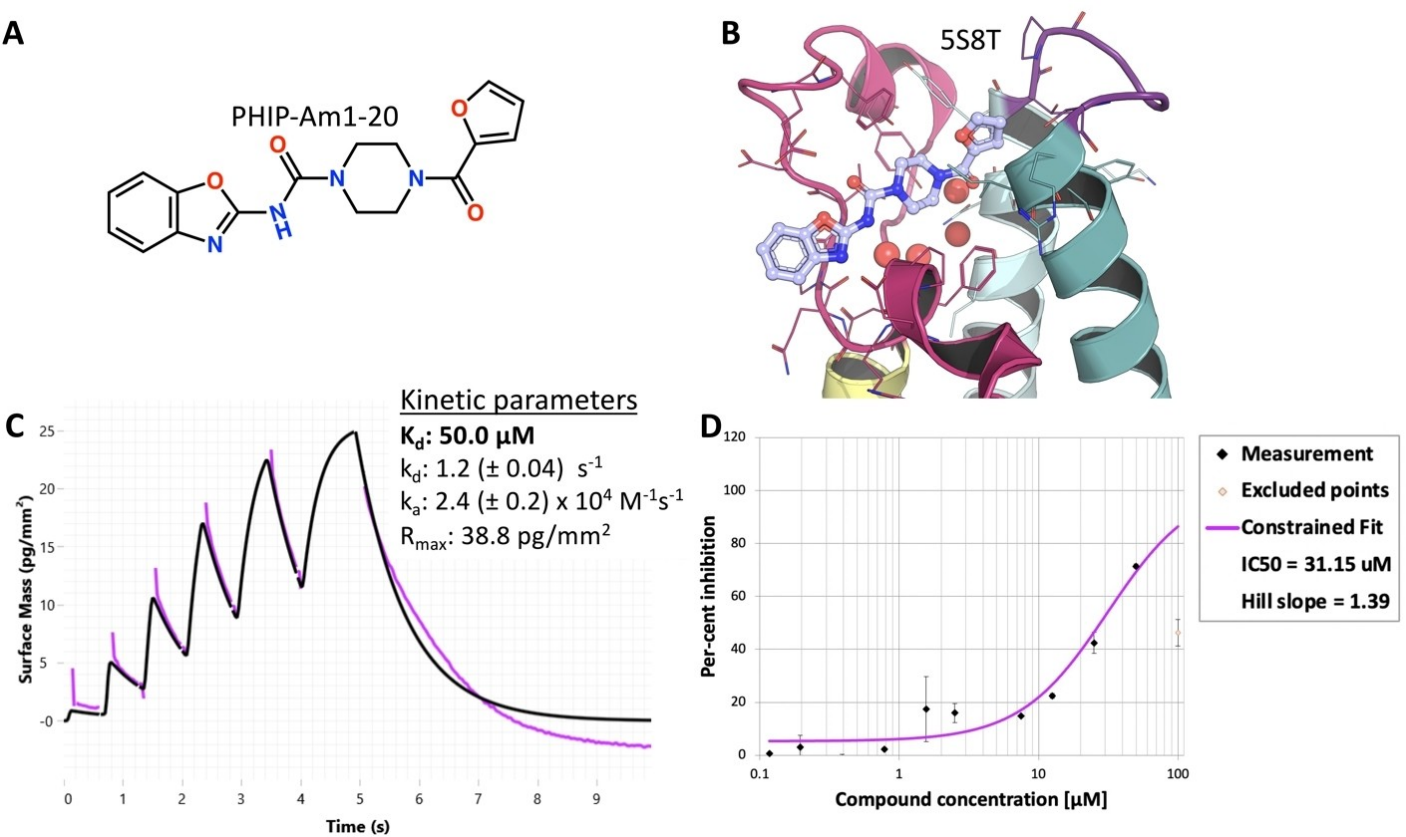
**Structural and assay binding of reaction product PHIP‐AM1‐20**. Panels A and B show the chemical structure and crystallographic binding pose of PHIP‐AM1‐20, respectively. The PDB code of the bound complex is written above B. Panel C shows kinetic parameters determined from the interaction kinetic curves by global fitting using a 1 : 1 interaction kinetic model modelled with the WAVEcontrol software. Panel D shows the dose response from the AlphaScreening assay. For both C and D, raw data in black and fitted binding curves in purple.

Given that the starting template fragment F709 did not yield any detectable signal in solution‐based biochemical assays, the elaborated crystallographic hits needed confirmation of activity and/or affinity via orthogonal methods. For this purpose, we ordered the pure hit compounds from Enamine Ltd or carried out a resynthesis in‐house (spectral data for 4 analogues resynthesised in house are in Suppl. Info. 9.1, S177). These were evaluated with an Amplified Luminescence Proximity Homogeneous Assay (AlphaScreen™)^[74]^ for biochemical activity; and by time‐resolved grating‐coupled interferometry‐based biosensor assay, using a pulsed injection Scheme (waveRAPID®),^[75]^ to estimate affinity and interaction kinetic rate constants (Figure 8).

Compound PHIP‐AM1‐20, from Iter 1 (Figure 2, Suppl info. 10, S179), had a measurable effect in both assays with a K_d_ and IC_50_ of 50.03 μM and 31.15 μM corresponding to ligand efficacies of 2.00 and 1.25 μM/heavy atom for the kinetic analysis and alpha‐screen, respectively (Figure 8). The binding event appears to be a reversible one‐step 1 : 1 interaction. This represents a 2‐ to 3‐fold increase in binding affinity when compared to the best binder obtained by Cox et al., (2016), which were elaborations around the first DSI‐poised fragment binders identified against PHIP(2).^[14]^ Overall, on‐scale potency was achieved starting from an undetectable fragment.

## Conclusions

This exploratory experiment generates extensive SAR data within a single DMTA step, that appears able to accelerate the overall hit‐to‐lead process. By combining crude array synthesis and X‐ray crystallographic structural determination of the resulting molecules, our conceptual fragment to drug‐like molecule exercise dramatical shrinks time, solvent usage, costs, energy, and many synthetic, extraction, work‐up and purification bottlenecks. A crude fragment screen of the DSiP library at Diamond vs PHIP(2), led to a piperazinyl hit F709, whose structural elaboration prompted several thousand multi‐step, robotic‐driven reactions. The repertoire can be iteratively expanded and a single DMTA cycle allows immediate SAR decision‐making. To reduce crude compound analysis time, a semi‐automated LCMS analysis tool *MSCheck* supersedes the previous cumbersome manually evaluated LCMS chromatogram methods, representing further significant time saving.^[69]^ CRMs were submitted to XChem for X‐Ray analysis at high‐throughput levels and without compromising crystal integrity, bypassing purification steps and enabling the identification of crystal binders that map the crystallographic SAR landscape around PHIP(2) defined by our enumeration. Notable highlights include the identification of a hitherto unidentified binding pose that displaces all 4 conserved bromodomain waters, and a 3‐fold gain in activity of one of the crystallographic binders in a biochemical assay. This binding‐site purification of actives (B‐SPA) technique has shown promise in generating SAR information economically from large scale crystallographic readouts of fragment elaborations in CRMs and identifying reaction products with on‐scale activity. Our findings also suggest that B‐SPA could be a valuable tool in further streamlining drug discovery workflows by circumventing costly and polluting purification steps.

This method has proven to be effective at generating large amounts of crystallographic data yet lacks an equally high‐throughput and automated means of validating those hits via assays and has yet to bypass the more time‐consuming, resource‐heavy, in‐house or outsourced compound resynthesis to reconfirm hits. However, efforts have been made in measuring K_d_ values from CRMs, which may combine efficiently with the novel pulsed injection schemes implemented for the time‐resolved kinetic analysis using grating‐coupled interferometry.[^76^, ^77^] Automated methods^[78]^ will also be needed to systematically analyse and rationalise the data resulting from these increasingly large high‐throughput crystallographic screenings.

This case study serves as an exemplar on how to generate crystallography‐driven SAR data quickly with minimal bottlenecks, caveated by the fact that improved binding interactions are not necessarily commensurate with increased activity, nor are they a substitute for biological activity derived from an assay. Future efforts and workflows will benefit from regular STOP/GO decisions informed by holistic structural and biological data input to modify the direction of the research and maximise the chances of success.

## Funding

EPSRC (EP/P026990/1) is thanked for funding (JS, SHH, FvD). Wellcome Institutional Strategic Support Fund (ISSF) is thanked for funding this work at Sussex (JS, SHH). The Diamond Light Source is also thanked for funding (COL0108 ‐ HG). The SGC is a registered charity (number 1097737) that receives funds from AbbVie, Bayer Pharma AG, Boehringer Ingelheim, Canada Foundation for Innovation, Eshelman Institute for Innovation, Genome Canada, Innovative Medicines Initiative (EU/EFPIA) [ULTRA‐DD grant no. 115766], Janssen, Merck KgaA Darmstadt Germany, MSD, Novartis Pharma AG, Ontario Ministry of Economic Development and Innovation, Pfizer, FAPDF, CAPES, CNPq, São Paulo Research Foundation‐FAPESP, Takeda, and Wellcome [106169/ZZ14/Z].

## Data Access and Availability

Supporting data uploaded to Zendo (10.5281/zenodo.7586212) and includes: a summary of X‐ray and LCMS results for the reactions executed on the OpenTrons, output reports and summaries from MSCheck (semi‐automated LCMS analyzer tool) and the Python scripts used to execute single and multistep chemistry on the OpenTrons.

## Author Contribution

AA, SH‐H, AB, TK: experimental design, robotics and synthesis of compounds. HG, TK, LK, SH−H: protein production and crystallization. HG, AA, CA, TK, CW, WJB; X‐Ray analysis and modelling. LK, EAF, JB, OF: assays. WT, SH−H, AA: post‐synthetic and quality control analysis. JS, FvD, PCB; project conceptualisation, oversight and funding acquisition. All authors contributed to data analysis, curation, drafting and proofreading the final manuscript.

## Conflict of Interests

The authors declare no conflict of interest.

1

## Supporting information

As a service to our authors and readers, this journal provides supporting information supplied by the authors. Such materials are peer reviewed and may be re‐organized for online delivery, but are not copy‐edited or typeset. Technical support issues arising from supporting information (other than missing files) should be addressed to the authors.

Supporting Information

## Data Availability

The data that support the findings of this study are openly available in Zenodo at https://zenodo.org/records/7586212, reference number 7586212.
