# Supplementary material for: Binding‐Site Purification of Actives (B‐SPA) Enables Efficient Large‐Scale Progression of Fragment Hits by Combining Multi‐Step Array Synthesis With HT Crystallography
Source: Angew Chem Int Ed Engl. 2025 Mar 18;64(16):e202424373. doi: 10.1002/anie.202424373 (PMC12001203; doi:10.1002/anie.202424373)

Angewandte Chemie
Eine Zeitschrift der Gesellschaft Deutscher Chemiker

Supporting Information

Binding-Site Purification of Actives (B-SPA) Enables Efficient Large-Scale Progression of Fragment Hits by Combining Multi-Step Array Synthesis With HT Crystallography

H. Grosjean, A. Aimon, S. Hassell-Hart, W. Thompson, L. Koekemoer, J. Bennett, A. Bradley, C. Anderson, C. Wild, W. J. Bradshaw, E. A. FitzGerald, T. Krojer, O. Fedorov, P. C. Biggin, J. Spencer, F. von Delft**

SUPPLEMENTARY INFORMATION

Binding-site purification of actives (B-SPA) enables efficient large-scale progression of fragment hits combining multi-step array synthesis with HT crystallography

Harold Grosjean[#1,2], Anthony Aimon[#1,3], Storm Hassell-Hart[#4], Warren Thompson[#1,3], Lizbé Koekemoer[5,6], James Bennett[5,6], Anthony Bradley[6], Cameron Anderson[2], Conor Wild[1], William J Bradshaw[6], Edward A. FitzGerald[7], Tobias Krojer[6], Oleg Fedorov[6], Philip C. Biggin[2], John Spencer*[4,8] and Frank von Delft*[1,3,5,6,9]

[#] Joint first authors

* Corresponding authors

Affiliation:

1. Diamond Light Source Ltd, Harwell Science and Innovation Campus, OX11 0QX, Didcot, UK
2. Structural Bioinformatics and Computational Biochemistry, Department of Biochemistry, University of Oxford, South Parks Road, OX1 3QU, Oxford, UK
3. Research Complex at Harwell, Harwell Science and Innovation Campus, OX11 0FA, Didcot, UK
4. Department of Chemistry, School of Life Sciences, University of Sussex, Falmer, BN1 9QJ
5. Centre for Medicines Discovery, University of Oxford, Old Road Campus, Roosevelt Drive, OX3 7DQ, Headington, UK
6. Structural Genomics Consortium, University of Oxford, Old Road Campus, Roosevelt Drive, OX3 7DQ, Headington, UK
7. Creoptix AG, Zugerstrasse 76, 8820 Wädenswil, Switzerland
8. Sussex Drug Discovery Centre (SDDC), School of Life Sciences, University of Sussex, Falmer, BN1 9QJ, UK
9. Department of Biochemistry, University of Johannesburg, Auckland Park 2006, South Africa

1 MATERIALS AND METHODS S3

1.1 QUALITY CONTROL S3
1.2 PROTEIN EXPRESSION, PURIFICATION, AND CRYSTALLIZATION S3
1.3 XCHEM SCREENING OF CRUDE REACTION MIXTURES, MODEL BUILDING, REFINEMENT AND ELECTRON DENSITY ANALYSIS S3
1.4 CHEMICAL SIMILARITY CALCULATIONS S4
1.5 INTERACTION KINETIC ANALYSIS – GCI S4
1.6 ALPHA-SCREEN ASSAY S4
1.7 HIGH-THROUGHPUT LCMS ANALYSIS USING MSCHECK S5
1.7.1 MSCHECK REPORTS AND COMMON EXAMPLES OF TRUE AND FALSE POSITIVE AND NEGATIVE REACTION SUCCESSES IDENTIFIED BY MSCHECK AND HUMAN-ANALYSIS..................... **S5**
1.8 REFERENCES FOR EXPERIMENTAL METHODS S9

2 DEVELOPMENT OF A LIQUID-HANDLER APPLICABLE UREA SYNTHESIS (IT 1+2)........................922

3 DEVELOPMENT OF A MULTIPLE STEP LIQUID-HANDLER APPLICABLE ELABORATED UREA SYNTHESIS (IT 2 +3) S22

4 DEVELOPMENT OF A MULTIPLE STEP LIQUID-HANDLER APPLICABLE ELABORATED SULPHONAMIDE UREA SYNTHESIS (IT 4) S29

5 DEVELOPMENT OF A MULTIPLE STEP LIQUID-HANDLER APPLICABLE ELABORATED UREA SYNTHESIS (IT 3.5 AND 4.5) S35

6 SYNTHESIS OF PURE HIT COMPOUNDS AS POSITIVE CONTROLS AND FOR FURTHER TESTING S37
7

7 ROBOTIC SYNTHESIS – EXPERIMENTAL AND ROBOTIC PROTOCOLS S400

7.1 ITERATION 1.0 AND 1.1 UREA REACTION S402
7.2 ITERATION 2.0 COUPLING REACTION S422
7.3 ITERATION 3.5 TRIPLE REACTION SEQUENCE S444
7.4 ITERATION 4.2 FIVE REACTIONS – SULPHONAMIDE SYNTHESES S566
7.5 ITERATION 3.0 .... S69
7.6 ITERATION 4.0 .... S733

8 COMPOUNDS SYNTHESISED AND SYNTHESIS QC RESULTS 80S79

8.1 ITERATION 1.0 .... S81
8.2 ITERATION 1.1 .... S87
8.3 ITERATION 2.0 .... S933
8.4 ITERATION 3.0 ....S1000
8.5 ITERATION 3.5 ....S1255
8.6 ITERATION 4.2 ....S16969

9 SPECTRA S1777

9.1 NMR S1777

10 2D-STRUCTURES OF REACTION PRODUCTS BOUND IN COCRYSTALS S17979

11 3D-STRUCTURES OF COMPOUNDS BOUND IN COCRYSTALS S1800

11.1 STARTING FRAGMENT AND DIVING REACTION PRODUCTS S1800
11.2 LATERALLY BOUND REACTION PRODUCTS S1811
11.3 STARTING MATERIALS S1833

1 Materials and methods

1.1 Quality control

LC–MS data was collected using an Agilent 1260/6120 SQ MS system (G1978B Multimode Source, G4225A Degasser, G1312B Binary Pump, G1316A (TCC) Oven, G1315D (DAD) Detector, G4260B ELSD Detector) connected to a G6120 LCMS Single Quad MS System. The column used was a Supelco Analytical Ascentis C18 (5cm x 2.1mm) with a particle size of 3.0 µm, and the column was held at 40 °C throughout. Mobile phase A was 0.1% formic acid in water, and mobile phase B was acetonitrile. A flow rate of 0.5 mL/min was used throughout, and the gradient was as follows: 5% B at 0.0 min; linear transition to 95% B until 3.0 min; held at 95% B until 3.5 min; linear transition back to 5% B until 4.5 min; and held at 5% B until 5.00 min for equilibration. An injection volume of 1 µL was used and the injection-to-injection cycle time for each sample was 5.7 min. Mass spectra were collected using multimode electron spray ionisation (ESI) and atmospheric pressure chemical ionization (APCI) over the m/z range of 100−600 in positive and negative ion modes. The source conditions were as follows: gas temperature: 250 °C; vaporizer temperature: 200 °C; nebulizer pressure: 35 psig; capillary voltage: 4 kV; and Vcharge: 2 kV.

1.2 Protein expression, purification, and crystallization

Proteins were expressed, purified and crystallized as previously reported.[1] Briefly, BL21 cells holding a vector coding for PHIP(2) were grown for 6 hours before expression was induced overnight with IPTG. The cultures were lyzed then centrifuged before the proteins contained in the supernatant were purified via nickel affinity chromatography and the resulting solution desalted. The his-tag was cleaved with TEV protease and the solution run again with nickel affinity chromatography. Proteins were concentrated by 20-fold and applied onto size exclusion chromatography where protein containing fractions were collected, concentrated to 15 mg/mL and flash frozen. PHIP(2) was crystallized in space group C2 at 4 °C by vapour diffusion in 230 nL sitting drops, formed by mixing 100 nL protein in wash buffer with 100 nL reservoir buffer (20% PEG8000 and 40 mM potassium phosphate) and 30 nL seeds of the same composition than reservoir.

1.3 XChem screening of crude reaction mixtures, model building, refinement and electron density analysis

The XChem screening was also done similarly to previously described.[1] Overall, Crystals suitable for screening were located with TexRank.[2] These were soaked with crude reaction mixtures using an ECHO acoustic liquid handler dispenser.[3] The crystals were incubated for 2 hours at 20°C and harvested with a SHIFTER[4] before being plunged into liquid nitrogen and shot at the i04-1 beamline (The Diamond Light Source, Harwell, UK). The XChemXplorer was used for crystallographic workflow management and paralleling.[5] Molecular replacements and initial refinements were performed with DIMPLE.[6] Ligands were fitted in Coot[7] and the structures refined with Buster[8] and deposited on the protein data bank (PDB) with deposition ID: G_1002190. Crystallographic factors and statistics are also available via the deposition ID.

Racemates are modelled into PanDDA event maps, and as such conventional crystallographic metrics of the goodness of fit of a single atom such as the B-factor[9] or EDIA[10] are not easily applicable due to the lack of reflections against which to refine and the sharp changes in electron density around atoms induced by mean map subtraction, respectively. The strength of the electron density around

the methyl group was calculated to quantify crystallographic support for each of the enantiomers. This was done by integrating the positive electron density in a 0.5Å radius about the methyl carbon by the Monte Carlo method[11] using 10000 samples, then dropping those with an electron density per unit Å of less than zero and taking the arithmetic mean. This was performed with a small program implemented in Python, available from the XChem Github: https://github.com/xchem/sample_bond_ed.

1.4 Chemical similarity calculations

Pairwise Tanimoto distances were calculated from the chemical fingerprints of each binding reaction product smile string with RDKit (v2022.03.3). The resulting values were subtracted to 1 yielding a similarity matrix where values close to 0 and 1 indicate high and low degrees of chemical similarity, respectively. Single linkage hierarchical clustering was applied onto the chemical similarity matrix using Euclidean distance with SciPy (v1.7.3).

1.5 Interaction Kinetic Analysis – GCI

Pulsed single-concentration surface-based biophysical measurements of binding kinetics were performed using a Creoptix® WAVE system (Creoptix, AG).[12] PHIP(2) was immobilized on a 4PCH biosensor surface using amine coupling. Briefly, a sensor chip was conditioned using injections of borate buffer (10 mM sodium tetraborate pH 9, 1 M NaCl). The sensor chip was activated using 1:1 mixture of 400 mM EDC/100 mM NHS for 420 s at 10 µL/min (Xantec). PHIP(2) was diluted to 30 µg/mL in sodium acetate buffer (10 mM pH 5.0) and injected over the active surface with a final protein immobilisation level of 5000 surface mass (pg/mm^2) corresponding to an injection time of 1800 s and a flow rate of 10 µL/min. After immobilisation, the surface was deactivated with ethanolamine-HCl (1.0 M pH 8.5) for 420 s.

Kinetic analysis for PHIP(2) and compounds was performed using a pulsed injection scheme (waveRAPID®) at 25°C with a 5s association and 20s dissociation at a flow rate of 200 µl/min and top concentration of 100 µM for each compound. Blank samples of the running buffer, (20mM HEPES, pH 7.5, 50 mM NaCl and 0.5% ethylene glycol), were injected during the measurements every fifth cycle. Compounds were applied to the immobilised surface and a reference channel. Data analysis and visualisation were performed using the WAVEcontrol software 4.5.13 (correction applied: X and Y offset; DMSO calibration; and double referencing). Kinetic parameters were calculated using the Direct Kinetics fitting engine with 1:1 kinetic binding model.

1.6 Alpha-screen assay

Assays were performed as described previously[13] with minor modifications from the manufacturer's protocol (PerkinElmer, USA). All reagents were diluted in 25 mM HEPES, 100 mM NaCl, 0.1 % BSA, pH 7.4 supplemented with 0.05 % CHAPS and allowed to equilibrate to room temperature prior to addition to plates. A 11-point 1:2.0 serial dilutions of the ligands were prepared on low-volume 384-well plates (ProxiPlateTM-384 Plus, PerkinElmer, USA), using LabCyte Echo liquid handler. Plates were filled with 12 µL/well with a mix of the assay buffer, biotinylated peptide [H-YSGRGKacGGKacGLGKacGGAKacRHRK(Biotin)- OH] and His-tagged protein to achieve final assay concentrations of 50 nM. Plates were sealed and incubated for a further 30 minutes, before the addition of 8 µl of the mixture of streptavidin-coated donor beads (12.5 µg/ml) and nickel chelate acceptor beads (12.5 µg/ml) under low light conditions. Plates were foil-sealed to protect from light, incubated at room temperature for 60 minutes and read on a PHERAstar FS plate reader (BMG Labtech, Germany) using an AlphaScreen 680 excitation/570 emission filter set. IC_{50} values were calculated in Prism 5 (GraphPad Software, USA) after normalization against corresponding DMSO controls and are given as the final concentration of compound in the 20 µl reaction volume.

1.7 High-throughput LCMS analysis using MSCheck

MSCheck is an open-source pip installable python package which utilises .mzML files, the open and generic XML format for mass spectra, converted from vendor files using ProteoWizard.[14,15] *MSCheck* searches for different parent ion matches (eg. M+H, M+Na) within a configurable M+H+± tolerance. Peaks in the total ion chromatogram (TIC) are initially identified and the peak's full width at half maximum (FWHM) calculated using Scipy's signal peak analysis algorithms (*find_peaks* and *peak_widths* functions respectively). The mass spectrum patterns, retrieved as discrete data points in the TIC , are analyzed around the peak above the FWHM height by searching for the sum of the parent mass of the target molecule and ion plus/minus the tolerance set. *MSCheck* generates a report in an .svg format that summaries the ion matches, and allows the results to be visually assessed rapidly for a large number of compounds.

1.7.1 MSCheck reports and common examples of true and false positive and negative reaction successes identified by MSCheck and human-analysis

Example 1: MSCheck true positive reaction success

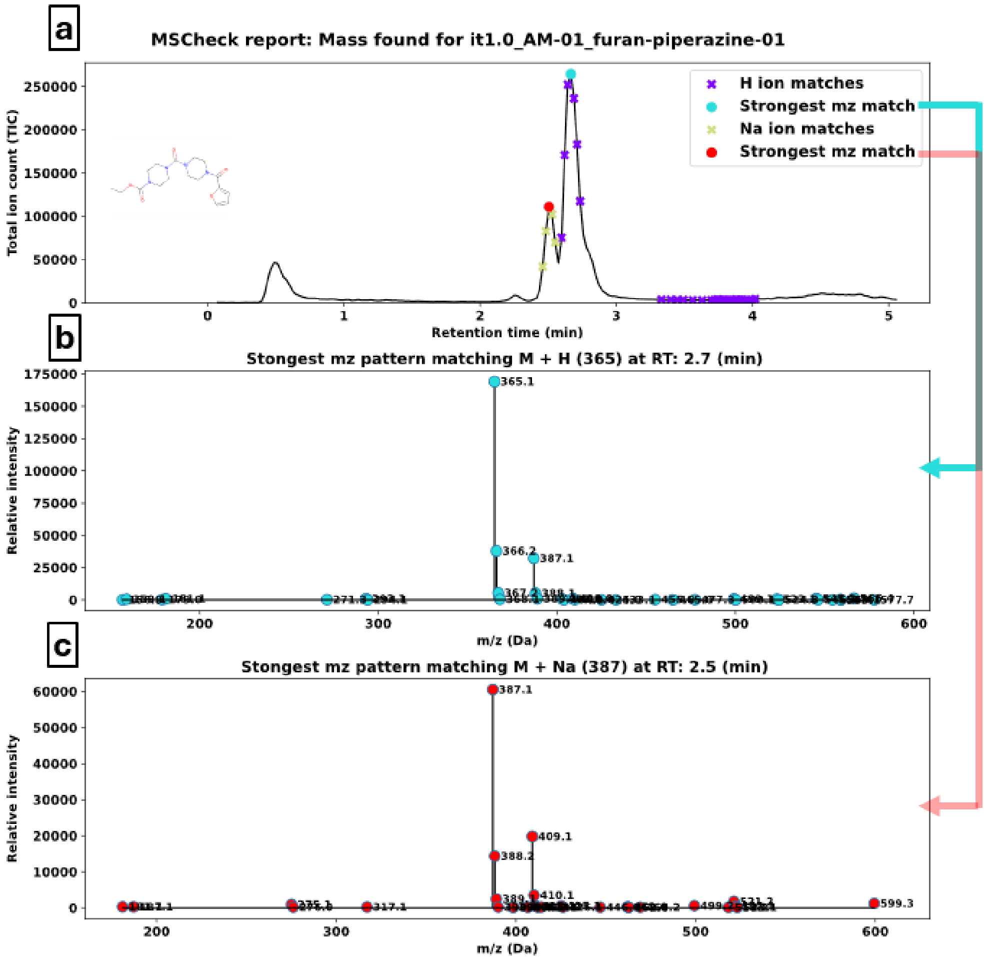

(a) The Total Ion Chromatogram for the first amide coupling compound made for Iteration 1.0. The color-coded legend indicates what M+ ions matched the expected compound, with the cross-markers indicating where the ion matches were found and the circular-markers indicating where the

strongest match was found. (b) Mass spectrum for the strongest M+H match and (C) Mass spectrum for the strongest M+Na match.

Example 2: MSCheck false positive reaction success that mis-matches human-analysis with less-prominent ion signals in the mass spectrum.

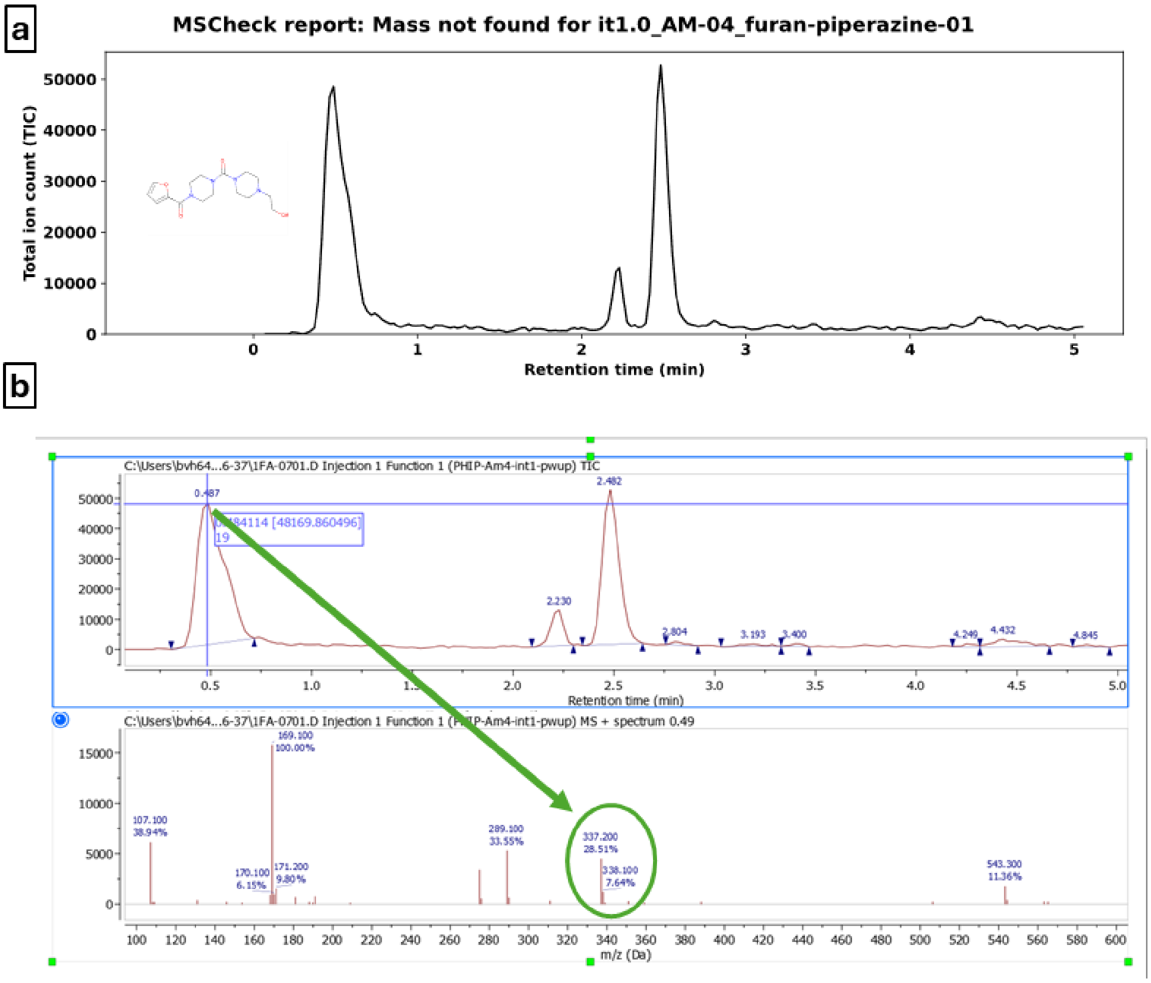

(a) *MSCheck* report indicating that no matching signals in the mass patterns were found and (b) Human-analysis found a relatively prominent signal matching the expected M+H ion signal match for the compound in the mass spectrum.

Example 3: MSCheck true positive reaction success where human-analysis failed to locate the ion signal match

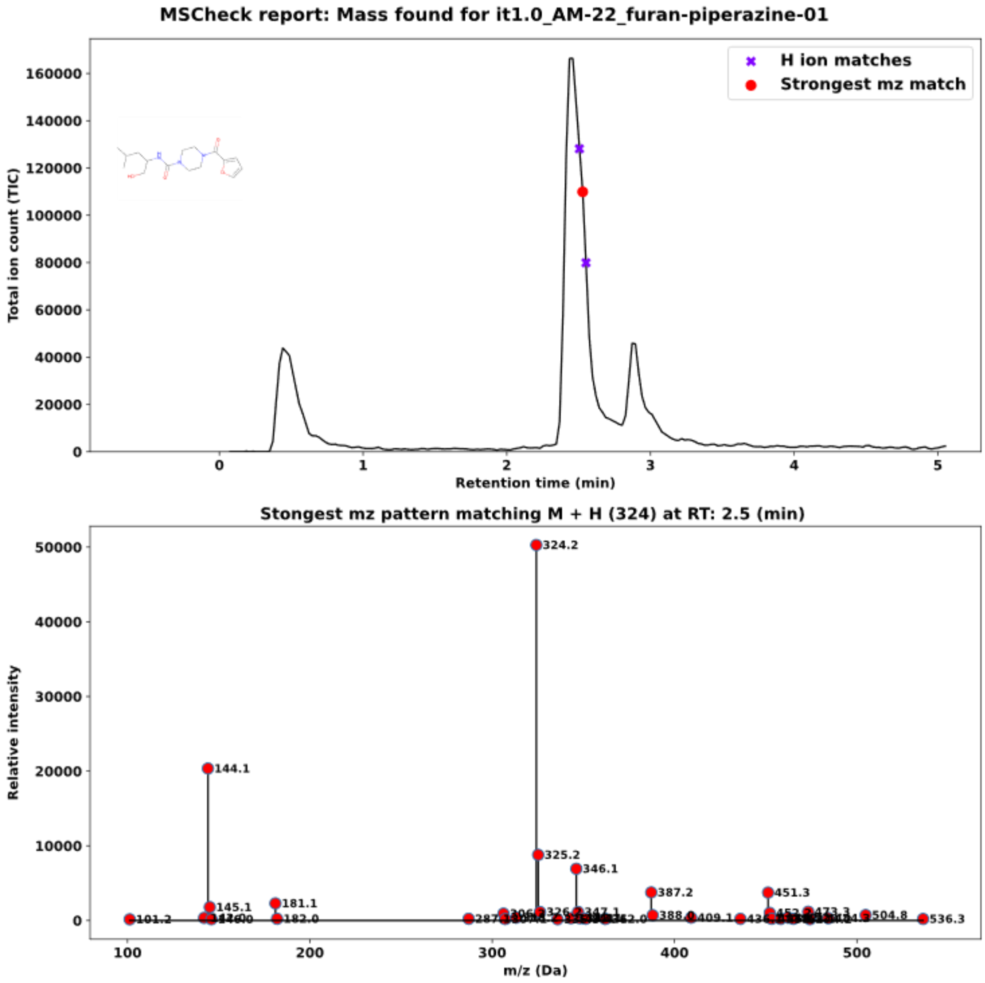

Example 4: MSCheck false positive reaction success with matching ion signal in noise

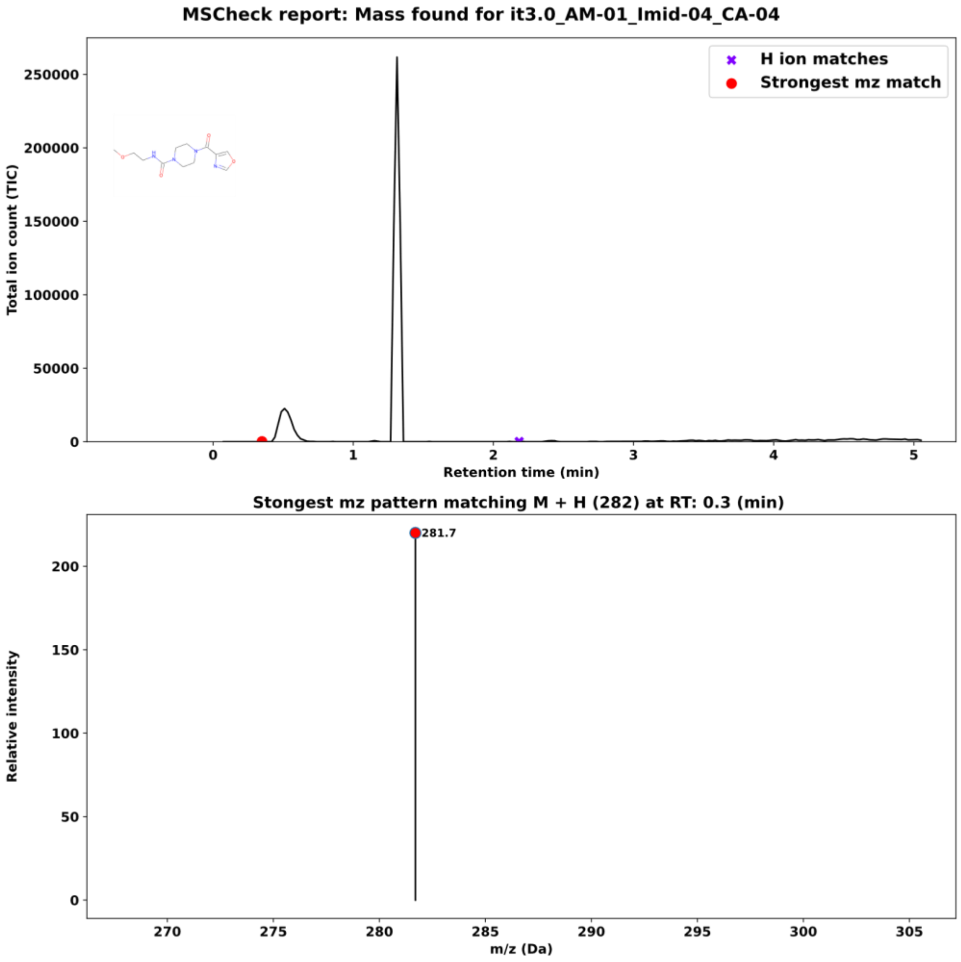

1.8 References for experimental methods

[1] H. Grosjean, M. Işık, A. Aimon, D. Mobley, J. Chodera, F. von Delft, P. C. Biggin, *J Comput Aided Mol Des* **2022**, *36*, 291–311.
[2] J. T. Ng, C. Dekker, M. Kroemer, M. Osborne, F. von Delft, *Acta Crystallogr D Biol Crystallogr* **2014**, *70*, 2702–2718.
[3] P. M. Collins, J. T. Ng, R. Talon, K. Nekrosiute, T. Krojer, A. Douangamath, J. Brandao-Neto, N. Wright, N. M. Pearce, F. von Delft, *Acta Crystallogr D Struct Biol* **2017**, *73*, 246–255.
[4] N. D. Wright, P. Collins, L. Koekemoer, T. Krojer, R. Talon, E. Nelson, M. Ye, R. Nowak, J. Newman, J. T. Ng, N. Mitrovich, H. Wiggers, F. von Delft, *Acta Crystallogr D Struct Biol* **2021**, *77*, 62–74.
[5] T. Krojer, R. Talon, N. Pearce, P. Collins, A. Douangamath, J. Brandao-Neto, A. Dias, B. Marsden, F. von Delft, *Acta Crystallogr D Struct Biol* **2017**, *73*, 267–278.
[6] M. Wojdyr, R. Keegan, G. Winter, A. Ashton, *Acta Crystallogr A Found Crystallogr* **2013**, *69*, s299–s299.
[7] P. Emsley, K. Cowtan, *Acta Crystallogr D Biol Crystallogr* **2004**, *60*, 2126–2132.
[8] O. S. Smart, T. O. Womack, C. Flensburg, P. Keller, W. Paciorek, A. Sharff, C. Vonrhein, G. Bricogne, *Acta Crystallogr D Biol Crystallogr* **2012**, *68*, 368–380.
[9] I. J. Tickle, *Acta Crystallogr D Biol Crystallogr* **2012**, *68*, 454–467.
[10] A. Meyder, E. Nittinger, G. Lange, R. Klein, M. Rarey, *J. Chem. Inf. Model.* **2017**, *57*, 2437–2447.
[11] J. Geweke, in *Handbook of Computational Economics*, Elsevier, **1996**, pp. 731–800.
[12] Ö. Kartal, F. Andres, M. P. Lai, R. Nehme, K. Cottier, *SLAS Discovery* **2021**, *26*, 995–1003.
[13] M. Philpott, J. Yang, T. Tumber, O. Fedorov, S. Uttarkar, P. Filippakopoulos, S. Picaud, T. Keates, I. Felletar, A. Ciulli, S. Knapp, T. D. Heightman, *Mol Biosyst* **2011**, *7*, 2899–2908.
[14] P. G. A. Pedrioli, J. K. Eng, R. Hubley, M. Vogelzang, E. W. Deutsch, B. Raught, B. Pratt, E. Nilsson, R. H. Angeletti, R. Apweiler, K. Cheung, C. E. Costello, H. Hermjakob, S. Huang, R. K. Julian, E. Kapp, M. E. McComb, S. G. Oliver, G. Omenn, N. W. Paton, R. Simpson, R. Smith, C. F. Taylor, W. Zhu, R. Aebersold, *Nat Biotechnol* **2004**, *22*, 1459–1466.
[15] M. C. Chambers, B. Maclean, R. Burke, D. Amodei, D. L. Ruderman, S. Neumann, L. Gatto, B. Fischer, B. Pratt, J. Egertson, K. Hoff, D. Kessner, N. Tasman, N. Shulman, B. Frewen, T. A. Baker, M.-Y. Brusniak, C. Paulse, D. Creasy, L. Flashner, K. Kani, C. Moulding, S. L. Seymour, L. M. Nuwaysir, B. Lefebvre, F. Kuhlmann, J. Roark, P. Rainer, S. Detlev, T. Hemenway, A. Huhmer, J. Langridge, B. Connolly, T. Chadick, K. Holly, J. Eckels, E. W. Deutsch, R. L. Moritz, J. E. Katz, D. B. Agus, M. MacCoss, D. L. Tabb, P. Mallick, *Nat Biotechnol* **2012**, *30*, 918–920.

2 Development of a Liquid-Handler Applicable Urea Synthesis (It 1+2)

4-Nitrophenyl morpholine-4-carboxylate

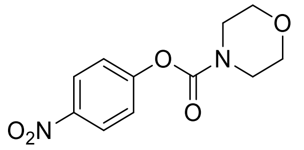

To a mixture of morpholine (87 µL, 1.50 mmol), triethylamine (278 µL, 2.00 mmol), and DCM (4 mL) was added 4-nitrochloroformate (202 mg, 1.00 mmol) and the resulting mixture stirred at ambient

temperature for 16 h. To the mixture was added DCM (10 mL) and water (10 mL). The resulting biphasic mixture was separated, and the resulting organic phase washed with 1M aqueous HCl (10 mL) and brine (10 mL), dried over anhydrous $MgSO_4$, filtered and concentrated under reduced pressure to give 4-nitrophenyl morpholine-4-carboxylate as a white solid (182 mg, 72%). ^1H NMR (600 MHz, d_6–DMSO): δ = 8.30-8.26 (2H, m), 7.48-7.44 (2H, m), 3.68-3.64 (4H, m), 3.63-3.58 (2H, m), 3.47-3.42 (2H, m).

Attempted Synthesis of (4-(Furan-2-carbonyl)piperazin-1-yl)(morpholino) methanone *via* Displacement of the Nitrophenol

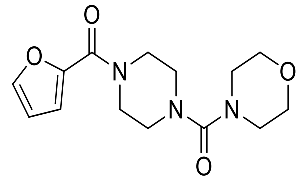

A mixture of furan-2-yl(piperazin-1-yl)methanone (18 mg, 0.10 mmol), 4-nitrophenyl morpholine-4-carboxylate (25 mg, 0.10 mmol), potassium carbonate (20 mg, 0.15 mmol), and acetonitrile (1 mL) was heated to 70 °C and stirred for 16 h. LCMS and TLC analysis showed no reaction had occurred and therefore the reaction was terminated at this stage.

Attempted One-Pot Synthesis of (4-(Furan-2-carbonyl)piperazin-1-yl)(morpholino) methanone

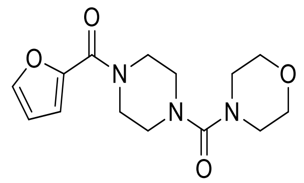

To a mixture of furan-2-yl(piperazin-1-yl)methanone (162 mg, 0.90 mmol), triethylamine (188 µL, 1.35 mmol), and anhydrous THF (4 mL) was added CDI (161 mg, 0.99 mmol) and the resulting mixture stirred for 16 h at ambient temperature. To the mixture was added morpholine (87 µL, 0.99 mmol) and the resulting mixture stirred for 24 h. LCMS analysis at this stage showed ~1% desired urea and therefore the reaction was terminated at this stage.

Attempted One-Pot Synthesis of *N*-Cyclopropyl-4-(furan-2-carbonyl)piperazine-1-carboxamide at 70 °C

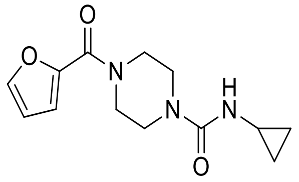

To a mixture of furan-2-yl(piperazin-1-yl)methanone (162 mg, 0.90 mmol), triethylamine (188 µL, 1.35 mmol), and anhydrous THF (4 mL) was added CDI (161 mg, 0.99 mmol) and the resulting mixture stirred for 3 h at ambient temperature. To the mixture was added cyclopropylamine (69 µL, 0.99 mmol) and the resulting mixture heated to 70 °C. LCMS after 16 h showed no product and therefore the reaction was terminated at this stage.

Attempted One-Pot Synthesis of 1-(4-(Furan-2-carbonyl)piperazine-1-carbonyl)-3-methyl-1*H*-imidazol-3-ium iodide

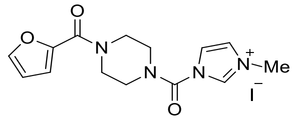

To a mixture of furan-2-yl(piperazin-1-yl)methanone (162 mg, 0.90 mmol), triethylamine (188 µL, 1.35 mmol), and anhydrous THF (4 mL) was added CDI (161 mg, 0.99 mmol) and the resulting mixture stirred for 3 h at ambient temperature. To the mixture was added methyl iodide (224 µL, 3.60 mmol) and the resulting mixture stirred for 16 h at ambient temperature. LCMS analysis at this stage showed no formation of the methylated species, with only the intermediate observed. The reaction was therefore concluded at this stage.

Testing the Telescoped Synthesis of (4-(Furan-2-carbonyl)piperazin-1-yl)(morpholino) methanone

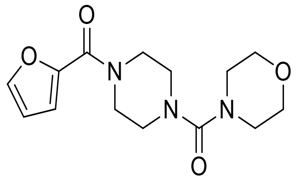

To a mixture of furan-2-yl(piperazin-1-yl)methanone (162 mg, 0.90 mmol), triethylamine (188 µL, 1.35 mmol), and anhydrous THF (4 mL) was added CDI (161 mg, 0.99 mmol) and the resulting mixture stirred for 3 h at ambient temperature. To the mixture was added DCM (5 mL) and water (5 mL) and the resulting mixture filtered through a hydrophobic frit. The organic phase was concentrated under reduced pressure to give a yellow gum (230 mg) which was carried into the next stage without any further purification.

The resulting residue was dissolved in acetonitrile (5 mL) and to the mixture was added methyl iodide (224 µL, 3.60 mmol). The mixture was stirred for 16 h at ambient temperature and concentrated under reduced pressure to give a yellow solid that was carried forward into the next stage without purification.

The resulting solid was dissolved in DCM (5 mL) and to the mixture was added morpholine (87 µL, 0.99 mmol). The mixture was stirred for 16 h at ambient temperature. To the mixture was added 2M aqueous HCl (5 mL) and the resulting mixture filtered through a hydrophobic frit. The organic phase was concentrated under reduced pressure to give (4-(furan-2-carbonyl)piperazin-1-yl)(morpholino) methanone as a pale yellow solid (106 mg, 40%). ^{1}H NMR (600 MHz, d_6–DMSO): δ = 7.85 (1H, m), 7.01 (1H, d, J = 3.4 Hz), 6.63 (1H, dd, J = 1.7, 3.4 Hz), 3.81-3.59 (4H, m), 3.58-3.54 (4H, m), 3.25-3.21 (4H, m), 3.18-3.14 (4H, m).

Testing the Telescoped Synthesis of Dimorpholinomethanone

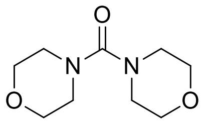

To a mixture of morpholine (79 µL, 0.90 mmol), triethylamine (188 µL, 1.35 mmol), and anhydrous THF (4 mL) was added CDI (161 mg, 0.99 mmol) and the resulting mixture stirred for 3 h at ambient temperature. To the mixture was added DCM (5 mL) and water (5 mL) and the resulting mixture filtered through a hydrophobic frit. The organic phase was concentrated under reduced pressure to give a yellow solid (66 mg) which was carried into the next stage without any further purification.

The resulting residue was dissolved in acetonitrile (5 mL) and to the mixture was added methyl iodide (224 µL, 3.60 mmol). The mixture was stirred for 16 h at ambient temperature and concentrated under

reduced pressure to give a yellow solid that was carried forward into the next stage without purification.

The resulting solid was dissolved in DCM (5 mL) and to the mixture was added morpholine (87 µL, 0.99 mmol). The mixture was stirred for 16 h at ambient temperature. To the mixture was added 2M aqueous HCl (5 mL) and the resulting mixture filtered through a hydrophobic frit. The organic phase was concentrated under reduced pressure to give dimorpholinomethanone as a white solid (39 mg, 22%). ^{1}H NMR (600 MHz, d_6–DMSO): δ = 3.57-3.53 (4H, m), 3.15-3.12 (4H, m).

Testing the Telescoped Synthesis of *N*-(4-Methoxyphenyl)morpholine-4-carboxamide

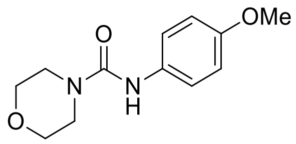

To a mixture of morpholine (79 µL, 0.90 mmol), triethylamine (188 µL, 1.35 mmol), and anhydrous THF (4 mL) was added CDI (161 mg, 0.99 mmol) and the resulting mixture stirred for 3 h at ambient temperature. To the mixture was added DCM (5 mL) and water (5 mL) and the resulting mixture filtered through a hydrophobic frit. The organic phase was concentrated under reduced pressure to give a yellow solid (67 mg) which was carried into the next stage without any further purification.

The resulting residue was dissolved in acetonitrile (5 mL) and to the mixture was added methyl iodide (224 µL, 3.60 mmol). The mixture was stirred for 16 h at ambient temperature and concentrated under reduced pressure to give a yellow solid that was carried forward into the next stage without purification.

The resulting solid was dissolved in DCM (5 mL) and to the mixture was added *p*-aniside (122 mg, 0.99 mmol). The mixture was stirred for 16 h at ambient temperature. To the mixture was added 2M aqueous HCl (5 mL) and the resulting mixture filtered through a hydrophobic frit. The organic phase was concentrated under reduced pressure to give *N*-(4-methoxyphenyl)morpholine-4-carboxamide as an off-white solid (38 mg, 18%). ^{1}H NMR (600 MHz, d_6–DMSO): δ = 8.35 (1H, s), 7.35-7.31 (2H, m), 6.84-6.80 (2H, m), 3.70 (3H, s), 3.59 (4H, t, J = 4.8 Hz), 3.39 (4H, J = 4.8 Hz).

Testing the Telescoped Synthesis of *N*-(4-Methoxyphenyl)morpholine-4-carboxamide (Reversed Order of Addition Steps)

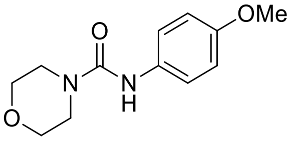

To a mixture of *p*-aniside (111 mg, 0.90 mmol), triethylamine (188 µL, 1.35 mmol), and anhydrous THF (4 mL) was added CDI (161 mg, 0.99 mmol) and the resulting mixture stirred for 3 h at ambient temperature. To the mixture was added DCM (5 mL) and water (5 mL) and the resulting mixture filtered through a hydrophobic frit. The organic phase was concentrated under reduced pressure to give a brown gum that was carried forward into the next stage without purification.

The resulting residue was dissolved in acetonitrile (5 mL) and to the mixture was added methyl iodide (224 µL, 3.60 mmol). The mixture was stirred for 16 h at ambient temperature and concentrated under reduced pressure and carried forward into the next stage without purification.

The resulting solid was dissolved in DCM (5 mL) and to the mixture was added morpholine (87 µL, 0.99 mmol). The mixture was stirred for 16 h at ambient temperature. To the mixture was added 2M aqueous HCl (5 mL) and the resulting mixture filtered through a hydrophobic frit. The organic phase was concentrated under reduced pressure to give *N*-(4-methoxyphenyl)morpholine-4-carboxamide as an off-white solid (98 mg, 46%). Data in agreement with that reported above.

Testing the Telescoped Synthesis of Dimorpholinomethanone Under the Liquid-Handling Model Conditions

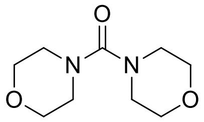

To a PFE reaction vessel was added mixture of morpholine (10 µL, 0.113 mmol), triethylamine (24 µL, 0.169 mmol), CDI (0.124 mmol, 20 mg), and anhydrous THF (0.5 mL). The vessel was covered and left at ambient temperature for 16 h (<u>not stirred</u>). The solvent was removed by blowing down under a stream of argon to give a yellow gum which was used in the next stage without further manipulation.

To the resulting residue in the PFE reaction vessel was added MeI (28 µL, 0.452 mmol) and MeCN (0.63 mL). The vessel was covered and left at ambient temperature for 16 h (<u>not stirred</u>). The solvent was removed by blowing down under a stream of argon to give a yellow gum which was used in the next stage without further manipulation.

To the resulting residue in the PFE reaction vessel was added morpholine (11 µL, 0.124 mmol) and DCM (0.63 mL). The vessel was covered and left at ambient temperature for 16 h (not stirred). The solvent was removed by blowing down under a stream of argon. LCMS and NMR analysis showed the presence of the imidazole intermediate with no product formation; therefore, the reaction was terminated at this stage.

Testing the Telescoped Synthesis of Dimorpholinomethanone Under the Liquid-Handling Model Conditions with An Addition Work-up Stage After the 1st Step

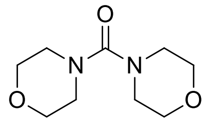

To a PFE reaction vessel was added mixture of morpholine (10 µL, 0.113 mmol), triethylamine (24 µL, 0.169 mmol), CDI (0.124 mmol, 20 mg), and anhydrous THF (0.5 mL). The vessel was covered and left at ambient temperature for 16 h (not stirred). To the mixture was added DCM (1 mL) and water (1 mL). The resulting biphasic mixture was separated, and the organic phase concentrated under reduced pressure to give an off-white solid (19 mg) which was used in the next stage without further manipulation.

To a PFE reaction vessel was added the crude from the first stage, MeI (28 µL, 0.452 mmol) and MeCN (0.63 mL). The vessel was covered and left at ambient temperature for 16 h (not stirred). The solvent was removed by blowing down under a stream of argon to give a yellow gum which was used in the next stage without further manipulation.

To the resulting residue in the PFE reaction vessel was added morpholine (11 µL, 0.124 mmol) and DCM (0.63 mL). The vessel was covered and left at ambient temperature for 16 h (not stirred). To the mixture was added DCM (1 mL) and 2M aqueous HCl (1 mL). The resulting biphasic mixture was separated and the organic phase concentrated under reduced pressure to give an off-white solid (5 mg). NMR analysis showed the major component was the desired product, with lower-level imidazole and morpholine impurities observed.

(1*H*-Imidazol-1-yl)(morpholino)methanone

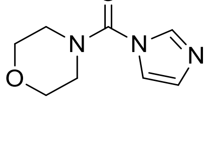

To a mixture of CDI (1.61 g, 9.90 mmol) and anhydrous THF (20 mL) was added morpholine (0.79 mL, 9.00 mmol) and triethylamine (1.88 mL, 13.50 mmol). The resulting mixture was a stirred at ambient temperature for 72 h. To the mixture was added ethyl acetate (50 mL) and water (50 mL). The resulting biphasic mixture was separated, and the aqueous phase extracted with ethyl acetate (3 x 50 mL). The combined organic extracts were washed with brine (50 mL), dried over anhydrous Na_2SO_4, filtered, and concentrated under reduced pressure to give a white solid. The resulting solid was purified by automated flash column chromatography (*n*-Hexane/EtOAc, 100:0 – 0:100 then 0-35% MeOH, 30 g SiO_2). The appropriate fractions were combined and concentrated under reduced pressure to give (1*H*-imidazol-1-yl)(morpholino)methanone as a white solid (336 mg, 21%). ^{1}H NMR (600 MHz, d_6–DMSO): δ = 8.04 (1H, m), 7.48 (1H, m), 7.03 (1H, m), 3.65 (4H, dd, J = 5.7, 4.1 Hz), 3.52-3.47 (4H, m).

(1*H*-Imidazol-1-yl)(morpholino)methanone (Base Free Conditions)

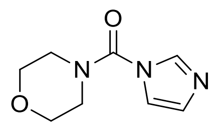

To a mixture of CDI (4.82 g, 29.7 mmol) and DCM (50 mL) was added dropwise morpholine (2.36 mL mL, 27.0 mmol). The resulting mixture was stirred at ambient temperature for 16 h. To the mixture was added water (150 mL) and the resulting biphasic mixture was separated. The aqueous phase was extracted with DCM (3 x 150 mL) and the combined organic extracts washed with brine (150 mL), dried over anhydrous $MgSO_4$, filtered, and concentrated under reduced pressure to give (1*H*-imidazol-1-yl)(morpholino)methanone as a white solid (1.32 g, 82%). Data in agreement with that reported above.

Testing the Telescoped Synthesis of Dimorpholinomethanone Under the Liquid-Handling Model Conditions From (1*H*-Imidazol-1-yl)(morpholino)methanone

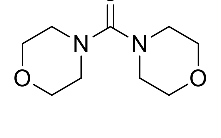

To a PFE reaction vessel was added (1*H*-imidazol-1-yl)(morpholino)methanone (20 mg, 0.113 mmol), MeI (28 µL, 0.452 mmol) and MeCN (0.63 mL). The vessel was covered and left at ambient temperature for 16 h (<u>not stirred</u>). The solvent was removed by blowing down under a stream of argon to give a yellow gum which was used in the next stage without further manipulation.

To the resulting residue in the PFE reaction vessel was added morpholine (11 µL, 0.124 mmol) and DCM (0.63 mL). The vessel was covered and left at ambient temperature for 16 h (not stirred). To the mixture was added DCM (1 mL) and 2M aqueous HCl (1 mL). The resulting biphasic mixture was separated, and the organic phase concentrated under reduced pressure to give an off-white solid (9 mg). NMR analysis showed the major component was the desired product, with lower level imidazole and morpholine derived impurities observed.

Solvent Screening for the Formation of *N*-(4-Methoxyphenyl)morpholine-4-carboxamide

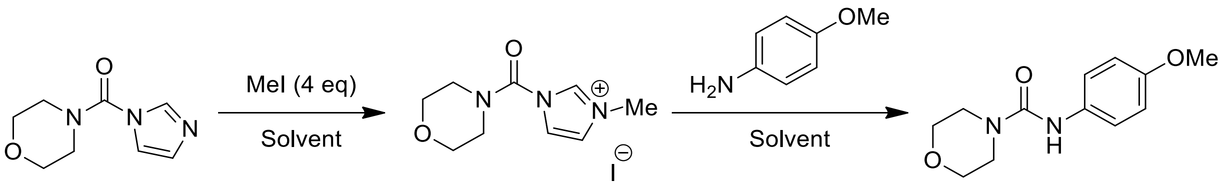

General Protocol:

To a PFE reaction vessel was added (1*H*-imidazol-1-yl)(morpholino)methanone (20 mg, 0.113 mmol), MeI (28 µL, 0.452 mmol), and solvent (0.5 mL). The vessel was covered and left at ambient temperature for 16 h (not stirred). The solvent was removed by blowing down under a stream of argon to give a yellow gum which was used in the next stage without further manipulation.

To the resulting residue in the PFE reaction vessel was added *p*-aniside (15 mg, 0.124 mmol) and solvent (0.5 mL). The vessel was covered and left at ambient temperature for 72 h (not stirred). At this stage the reactions were analysed by LCMS.

To the reactions which showed evidence of the product formation was added DCM (1 mL) and 2M aqueous HCl (1 mL). The resulting biphasic mixture was passed through a hydrophobic frit and concentrated under reduced pressure. The resulting residues were analysed by NMR to estimate the purity of the sample *via* integration of the product and the *p*-aniside signals.

Solvent	LCMS mass observed?	Mass Recovery	NMR % Purity
MeCN	Y	10 mg	52
DCM	Y	12 mg	80
DMF	N	N/A	N/A
EtOAc	N	N/A	N/A
Toluene	N	N/A	N/A

(4-(1*H*-Imidazole-1-carbonyl)piperazin-1-yl)(furan-2-yl)methanone

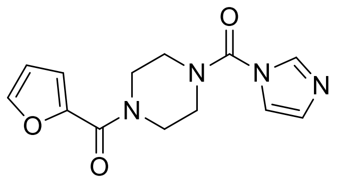

To a mixture of CDI (4.82 g, 29.7 mmol) and DCM (50 mL) was added furan-2-yl(piperazin-1-yl)methanone (4.87 g, 27.0 mmol). The resulting mixture was stirred at ambient temperature for 16 h. To the mixture was added water (150 mL) and the resulting biphasic mixture was separated. The aqueous phase was extracted with DCM (3 x 150 mL) and the combined organic extracts washed with brine (150 mL), dried over anhydrous $MgSO_4$, filtered, and concentrated under reduced pressure to give (4-(1*H*-imidazole-1-carbonyl)piperazin-1-yl)(furan-2-yl)methanone as a white solid (6.83 g, 92%). ^{1}H NMR (600 MHz, d_6–DMSO): δ = 8.07 (1H, s), 7.86 (1H, d, *J* = 1.8 Hz), 7.51 (1H, m), 7.07-7.03 (2H, m), 6.65 (1H, dd, *J* = 3.5, 1.8 Hz), 3.92-3.72 (4H, m), 3.65-3.55 (4H, m).

Nucleophile Screening for the Formation of (4-(1*H*-Imidazole-1-carbonyl)piperazin-1-yl)(furan-2-yl)methanone Derived Ureas

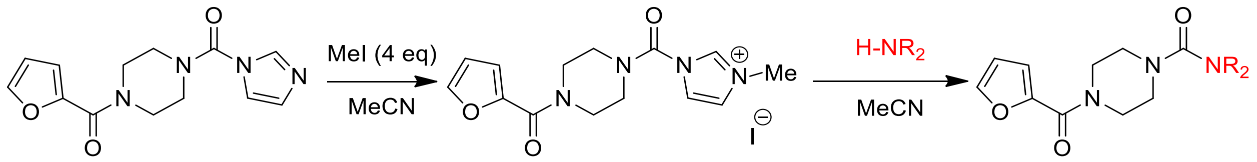

General Protocol:

To a PFE reaction vessel was added (4-(1*H*-imidazole-1-carbonyl)piperazin-1-yl)(furan-2-yl)methanone (31 mg, 0.113 mmol), MeI (28 µL, 0.452 mmol), and acetonitrile (0.5 mL). The vessel was covered and left at ambient temperature for 16 h (not stirred). The solvent was removed by blowing down under a stream of argon and the resulting residue was used in the next stage without further manipulation.

To the resulting residue in the PFE reaction vessel was added H-NR_2 (1.10 eq, 0.124 mmol) and acetonitrile (0.5 mL). The vessel was covered and left at ambient temperature for 72 h (not stirred). To the reaction was added DCM (1 mL) and 2M aqueous HCl (1 mL). The resulting biphasic mixture

was passed through a hydrophobic frit and concentrated under reduced pressure. The resulting residues were analysed by NMR to estimate the purity of the sample.

H-NR$_2$	Starting Material Mass	Crude Mass (mg)	NMR Purity (%)
p-Aniside	15 mg	5	90
NH$_2$Me (2M in THF)	62 µL	1	60
HNMe$_2$ (2M in THF)	62 µL	31	70
4-Nitroaniline	17 mg	3	>10

Nucleophile Screening for the Formation of (4-(1*H*-Imidazole-1-carbonyl)piperazin-1-yl)(furan-2-yl)methanone Derived Ureas Solvent Swap to DCM After Step 1

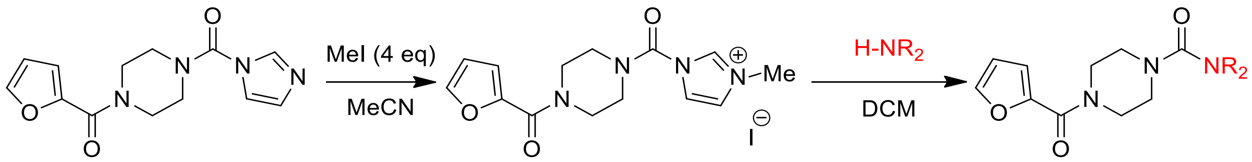

General Protocol:

To a PFE reaction vessel was added (4-(1*H*-imidazole-1-carbonyl)piperazin-1-yl)(furan-2-yl)methanone (31 mg, 0.113 mmol), MeI (28 µL, 0.452 mmol), and acetonitrile (0.5 mL). The vessel was covered and left at ambient temperature for 16 h (not stirred). The solvent was removed by blowing down under a stream of argon and the resulting residue was used in the next stage without further manipulation.

To the resulting residue in the PFE reaction vessel was added H-NR$_2$ (1.10 eq, 0.124 mmol) and DCM (0.5 mL). The vessel was covered and left at ambient temperature for 72 h (not stirred). LCMS analysis at this stage showed:

H-NR$_2$	Starting Material	LCMS (%)
p-Aniside	15 mg	25
NH$_2$Me (2M in THF)	62 µL	0
HNMe$_2$ (2M in THF)	62 µL	41
4-Nitroaniline	17 mg	0

Nucleophile Screening for the Formation of (4-(1*H*-Imidazole-1-carbonyl)piperazin-1-yl)(furan-2-yl)methanone Derived Ureas Solvent Swap to DMF and Addition of Base After Step 1

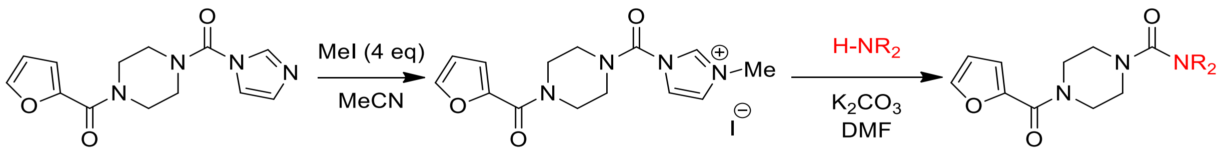

General Protocol:

To a PFE reaction vessel was added (4-(1*H*-imidazole-1-carbonyl)piperazin-1-yl)(furan-2-yl)methanone (31 mg, 0.113 mmol), MeI (28 µL, 0.452 mmol), and acetonitrile (0.5 mL). The vessel was covered and left at ambient temperature for 16 h (not stirred). The solvent was removed by blowing down under a stream of argon and the resulting residue was used in the next stage without further manipulation.

To the resulting residue in the PFE reaction vessel was added H-NR_2 (1.10 eq, 0.124 mmol), potassium carbonate (23 mg, 0.170 mmol), and DMF (0.5 mL). The vessel was covered and left at ambient temperature for 72 h (not stirred). LCMS analysis at this stage showed:

H-NR_2	Starting Material	Mass by LCMS?
p-Aniside	15 mg	Y
NH_2Me (2M in THF)	62 µL	N
$HNMe_2$ (2M in THF)	62 µL	Y
4-Nitroaniline	17 mg	N

To the successful reactions was added DCM (1 mL) and 2M aqueous HCl (1 mL). The resulting biphasic mixture was passed through a hydrophobic frit and concentrated under reduced pressure. The resulting residues were analysed by NMR to estimate the purity of the samples *via* integration of the furan protons in product and by-product:

p-Aniside = Brown gum (23 mg) – 68% purity by NMR.

$NHMe_2$ = yellow gum (18 mg) – 63% purity by NMR.

Base Screening For Formation of (4-(1*H*-Imidazole-1-carbonyl)piperazin-1-yl)(furan-2-yl)methanone Derived Ureas

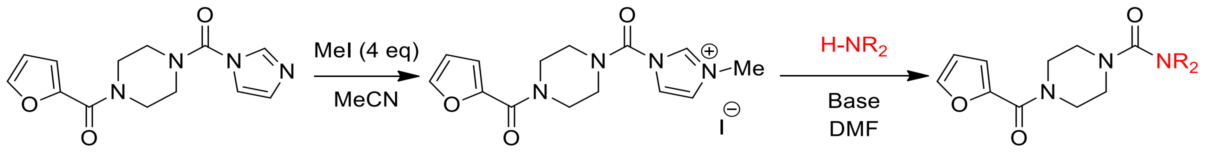

General Protocol:

To a PFE reaction vessel was added (4-(1*H*-imidazole-1-carbonyl)piperazin-1-yl)(furan-2-yl)methanone (31 mg, 0.113 mmol), MeI (28 µL, 0.452 mmol), and acetonitrile (0.5 mL). The vessel was covered and

left at ambient temperature for 16 h (not stirred). The solvent was removed by blowing down under a stream of argon and the resulting residue was used in the next stage without further manipulation.

To the resulting residue in the PFE reaction vessel was added *p*-aniside (15 mg, 0.124 mmol), Base (1.5 eq), and DMF (0.5 mL). The vessel was covered and left at ambient temperature for 72 h (not stirred). To the reactions was added DCM (1 mL) and 2M aqueous HCl (1 mL). The resulting biphasic mixture was passed through a hydrophobic frit and concentrated under reduced pressure. The resulting residues were analysed by LCMS/NMR to estimate the purity of the samples:

Base	Mass/Volume	Mass Recovery (mg)	NMR % Purity
K_2CO_3	23 mg	17	88
NEt_3	24 µL	20	85
$K_3PO_4.H_2O$	39 mg	0	-

Nucleophile Screening for the Formation of (1*H*-Imidazol-1-yl)(morpholino)methanone Derived Ureas Under the Optimised Reaction Conditions

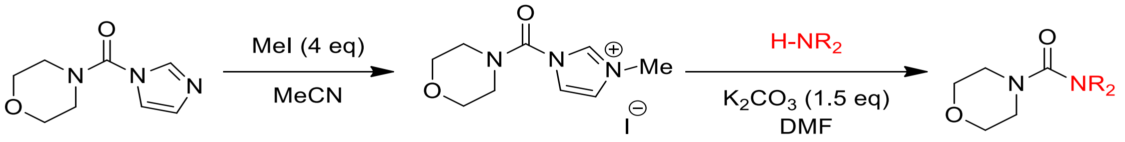

General Protocol:

To a PFE reaction vessel was added (1*H*-imidazol-1-yl)(morpholino)methanone (20 mg, 0.113 mmol), MeI (28 µL, 0.452 mmol), and MeCN (0.5 mL). The vessel was covered and left at ambient temperature for 16 h (not stirred). The solvent was removed by blowing down under a stream of argon to give a yellow gum which was used in the next stage without further manipulation.

To the resulting residue in the PFE reaction vessel was added H-NR_2 (1.10 eq, 0.124 mmol), potassium carbonate (23 mg, 0.170 mmol), and DMF (0.5 mL). The vessel was covered and left at ambient temperature for 72 h (not stirred). The vessel was covered and left at ambient temperature for 72 h (not stirred). To the reactions was added DCM (1 mL) and 2M aqueous HCl (1 mL). The resulting biphasic mixture was passed through a hydrophobic frit and concentrated under reduced pressure. The resulting residues were analysed by LCMS/NMR to estimate the purity of the samples:

Base	Mass/Volume	Mass Recovery (mg)	Product by LCMS?	NMR % Purity
Piperidine	12 µL	27	Y	>80%
Cyclopropylamine	9 µL	22	Y	>80%
$HNMe_2$ (2M in THF)	62 µL	26	Y	>90%

3 Development of a Multiple Step Liquid-Handler Applicable Elaborated Urea Synthesis (It 2 +3)

(4-Benzoylpiperazin-1-yl)(furan-2-yl)methanone – Evaluation of Amide Coupling Conditions and Work-Up Protocols

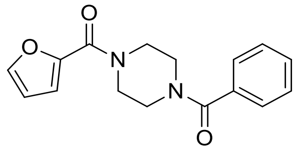

General Protocol:

To a mixture of furan-2-yl(piperazin-1-yl)methanone (45 mg, 0.250 mmol), benzonic acid (32 mg, 0.263 mmol), DIPEA (109 µL, 0.625 mmol), and DMA (1 mL) was added T3P (50% in EtOAc) (178 µL, 0.300 mmol). The resulting mixture was stirred for 16 h at ambient temperature.

Work up 1 (NH_4Cl):

To the mixture was added DCM (5 mL) and saturated aqueous NH_4Cl solution (5 mL). The resulting mixture was separated, and the organic phase concentrated under reduced pressure to give a yellow gum (72 mg). NMR analysis showed a mixture of the product, DMA, and lower-level unknown impurities.

Work up 2 ($NaHCO_3$):

To the mixture was added DCM (5 mL) and saturated aqueous $NaHCO_3$ solution (5 mL). The resulting mixture was separated, and the organic phase concentrated under reduced pressure to give a yellow gum (72 mg). NMR analysis showed a mixture of the product and DMA, with no other impurities.

(4-Benzoylpiperazin-1-yl)(furan-2-yl)methanone – Testing Amide Coupling Solvents

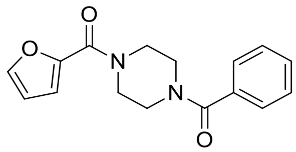

General Protocol:

To a mixture of furan-2-yl(piperazin-1-yl)methanone (45 mg, 0.250 mmol), benzonic acid (32 mg, 0.263 mmol), DIPEA (109 µL, 0.625 mmol), and solvent (1 mL) was added T3P (50% in EtOAc) (178 µL, 0.300 mmol). The resulting mixture was stirred for 16 h at ambient temperature. To the mixture was added

DCM (5 mL) and saturated aqueous NH_4Cl solution (5 mL). The resulting mixture was separated, and the organic phase concentrated under reduced pressure to give:

THF – 55 mg of a yellow gum. NMR analysis showed a mixture of the product and other lower level unknown impurities.

1,4-dioxane - ~100 mg of white solid. NMR analysis showed a mixture of the product and other unknown impurities.

Testing the Synthesis of (4-Benzoylpiperazin-1-yl)(furan-2-yl)methanone Under the Liquid-Handling Conditions

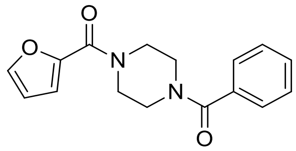

To a PFE reaction vessel was added furan-2-yl(piperazin-1-yl)methanone (6 mg, 0.036 mmol), benzonic acid (5 mg, 0.038 mmol), DIPEA (16 µL, 0.090 mmol), and DMA (0.5 mL). To the mixture was added T3P (50% in EtOAc) (26 µL, 0.043 mmol). The resulting mixture was stirred for 16 h at ambient temperature. The resulting mixture was covered and left at ambient temperature for 16 h (not stirred). To the resulting mixture was added DCM (1 mL) and saturated aqueous $NaHCO_3$ (1 mL). The resulting biphasic mixture was separated, and the organic phase concentrated by blowing down under a stream of argon to give a cloudy gum. NMR analysis showed the product, in addition to low levels of impurities and some residual DMA.

***tert*-Butyl 4-(1*H*-imidazole-1-carbonyl)piperazine-1-carboxylate**

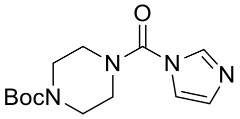

To a mixture of CDI (985 mg, 5.91 mmol) and DCM (10 mL) was added *tert*-butyl piperazine-1-carboxylate (1.00 g, 5.37 mmol). The resulting mixture was stirred at ambient temperature for 16 h. To the mixture was added water (25 mL) and the resulting biphasic mixture was separated. The aqueous phase was extracted with DCM (3 x 25 mL) and the combined organic extracts washed with brine (50 mL), dried over anhydrous $MgSO_4$, filtered, and concentrated under reduced pressure to give *tert*-butyl 4-(1*H*-imidazole-1-carbonyl)piperazine-1-carboxylate as a white solid (1.50 g, 99%). ^{1}H NMR (600 MHz, d_6–DMSO): δ = 8.03 (1H, s), 7.47 (1H, m), 3.51-3.46 (4H, m), 3.46-3.41 (4H, m).

(1*H*-Imidazol-1-yl)(piperazin-1-yl)methanone

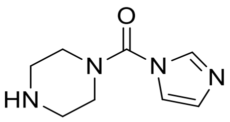

To a mixture of *tert*-butyl 4-(1*H*-imidazole-1-carbonyl)piperazine-1-carboxylate (400 mg, 1.426 mmol) and DCM (2 ml) was added dropwise TFA (2 mL). The resulting mixture was stirred for 16 h at ambient temperature. The resulting mixture was concentrated under reduced pressure. To the residue was added ethyl acetate (5 mL) and saturated aqueous Na_2CO_3 (2 mL). The biphasic mixture was separated, and the aqueous phase extracted with ethyl acetate (5 mL). The combined organic extracts were dried over anhydrous $MgSO_4$, filtered, and concentrated under reduced pressure to give (1*H*-imidazol-1-yl)(piperazin-1-yl)methanone as a yellow gum (156 mg, 61%). ^{1}H NMR (600 MHz, d_6–DMSO): δ = 8.02 (1H, s), 7.47 (1H, s), 7.02 (1H, s), 3.63 (4H, t, J = 5.2 Hz), 3.14 (4H, t, J = 5.2 Hz).

(4-(1*H*-Imidazole-1-carbonyl)piperazin-1-yl)(phenyl)methanone

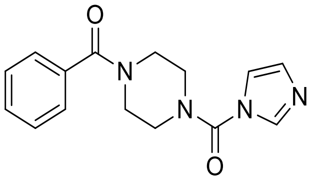

To a mixture of (1*H*-imidazol-1-yl)(piperazin-1-yl)methanone (128 mg, 0.713 mmol) and DCM (2 mL) was added benzoyl chloride (83 µL, 0.713 mmol) and triethylamine (333 µL, 2.317 mmol). The resulting mixture was stirred for 72 h. To the mixture was added water (10 mL) and ethyl acetate (10 mL). The resulting biphasic mixture was separated, and the aqueous phase extracted with ethyl acetate (3 x 10 mL). The combined organic extracts were washed with brine (20 mL), dried over anhydrous $MgSO_4$, filtered, and concentrated under reduced pressure to give (4-(1*H*-imidazole-1-carbonyl)piperazin-1-yl)(phenyl)methanone as a white solid (81 mg, 40%). ^{1}H NMR (600 MHz, d_6–DMSO): δ = 8.03 (1H, s), 7.48-7.38 (6H, m), 7.02 (1H, s), 3.81-3.36 (8H, m).

1-(4-Benzoylpiperazine-1-carbonyl)-3-methyl-1*H*-imidazol-3-ium iodide

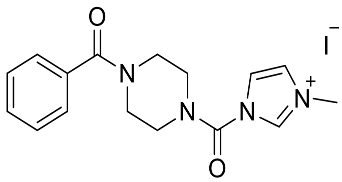

To a mixture of (4-(1*H*-imidazole-1-carbonyl)piperazin-1-yl)(phenyl)methanone (79 mg, 0.278 mmol) and acetonitrile (1 mL) was added MeI (87 µL, 1.39 mmol). The resulting mixture was stirred for 16 h at ambient temperature. The reaction was concentrated under reduced pressure to give a yellow solid of sufficient purity to be used in the subsequent reaction without further manipulation.

***N*-Benzyl-4-(furan-2-carbonyl)piperazine-1-carboxamide**

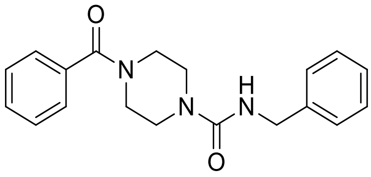

To a mixture of 1-(4-benzoylpiperazine-1-carbonyl)-3-methyl-1*H*-imidazol-3-ium iodide (371 mg, 0.89 mmol), potassium carbonate (185 mg, 1.34 mmol), and 1,4-dioxane (4 mL) was added *N*-benzylamine (107 µL, 0.98 mmol). The resulting mixture was stirred for 16 h at ambient temperature. To the reaction was added DCM (20 mL) and 2M aqueous HCl (20 mL). The biphasic mixture was separated and the organic phase washed with brine (20 mL), dried over anhydrous $MgSO_4$, filtered, and concentrated under reduced pressure to give *N*-benzyl-4-(furan-2-carbonyl)piperazine-1-carboxamide as a yellow gum (130 mg) of sufficient purity for testing (NMR analysis showed some residual dioxane as the only major impurity).

(1*H*-imidazol-1-yl)(piperazin-1-yl)methanone dihydrochloride

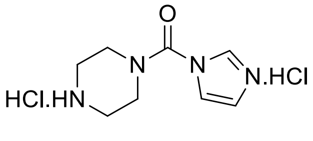

To a mixture of *tert*-butyl 4-(1*H*-imidazole-1-carbonyl)piperazine-1-carboxylate (500 mg, 1.78 mmol) and 1,4-dioxane (2 mL) was added 4M HCl (in 1,4-dioxane) (2 mL). The resulting mixture was stirred for 16 h at ambient temperature. The resulting mixture was concentrated under reduced pressure to give (1*H*-imidazol-1-yl)(piperazin-1-yl)methanone dihydrochloride as a white solid (445 mg, 99%). ^1H

NMR (600 MHz, d_6–DMSO): δ = 10.16 (1H, s), 9.57 (1H, m), 8.04 (1H, m), 7.79 (1H, m), 3.81-3.75 (2H, m), 3.52-3.42 (4H, m), 3.25-3.19 (2H, m).

Testing the Synthesis of (1*H*-imidazol-1-yl)(piperazin-1-yl)methanone dihydrochloride Under the Liquid-Handling Conditions

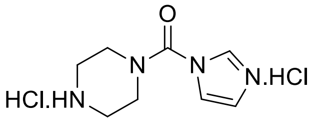

To a mixture of *tert*-butyl 4-(1*H*-imidazole-1-carbonyl)piperazine-1-carboxylate (10 mg, 0.036 mmol) and 1,4-dioxane (0.25 mL) in a PFE reaction vessel was added 4M HCl (in 1,4-dioxane) (0.25 mL). The resulting mixture was covered and left at ambient temperature for 16 h (not stirred). The solvent was removed by blowing down under a stream of argon to give a white solid. NMR analysis confirmed the presence of the product in sufficient purity to be used in the subsequent reaction without any further manipulation.

Testing the Synthesis of (4-(1*H*-Imidazole-1-carbonyl)piperazin-1-yl)(phenyl)methanone Under the Liquid-Handling Conditions

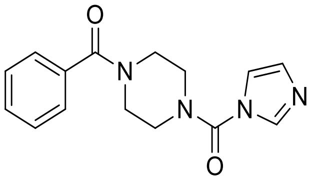

To a PFE reaction vessel was added a mixture of crude (1*H*-imidazol-1-yl)(piperazin-1-yl)methanone (9 mg, 0.036 mmol) and DMA (0.5 mL). To the mixture was added benzoic acid (5 mg, 0.038 mmol), T3P (50% in EtOAc) (26 µL, 0.043 mmol), and DIPEA (16 µL, 0.090 mmol). The resulting mixture was covered and left at ambient temperature for 16 h (not stirred). To the resulting mixture was added DCM (1 mL) and saturated aqueous $NaHCO_3$ (1 mL). The resulting biphasic mixture was separated, and the organic phase concentrated by blowing down under a stream of argon to give a cloudy gum. NMR analysis showed the product, in addition to low levels of impurities and some residual DMA.

Telescoped Complete Analogue Synthesis – Step 1, Synthesis of (1*H*-imidazol-1-yl)(piperazin-1-yl)methanone dihydrochloride Under the Model Liquid Handler Conditions

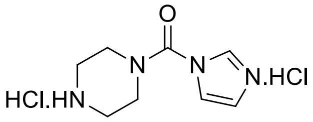

To a PFE reaction vessel was added *tert*-butyl 4-(1*H*-imidazole-1-carbonyl)piperazine-1-carboxylate (10 mg, 0.036 mmol) and 1,4-dioxane (0.25 mL). To the mixture was added 4M HCl (in 1,4-dioxane) (0.25 mL) and the resulting mixture was covered and left at ambient temperature for 16 h (not stirred). The reaction mixture was concentrated by blowing down under argon to give (1*H*-imidazol-1-yl)(piperazin-1-yl)methanone dihydrochloride as a white solid of sufficient purity to be utilised in the next stage without further purification.

Telescoped Complete Analogue Synthesis – Step 1, Synthesis of (4-(1*H*-Imidazole-1-carbonyl)piperazin-1-yl)(phenyl)methanone Under the Model Liquid Handler Conditions

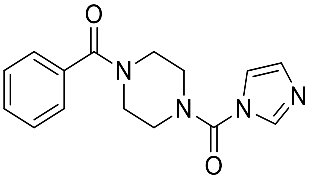

To the PFE reaction vessel containing crude (1*H*-imidazol-1-yl)(piperazin-1-yl)methanone dihydrochloride (0.036 mmol) was added benzoic acid (5 mg, 0.038 mmol), T3P (50% in EtOAc) (26 µL, 0.043 mmol), and DIPEA (16 µL, 0.090 mmol). The resulting mixture was covered and left at ambient temperature for 16 h (not stirred). To the resulting mixture was added DCM (1 mL) and saturated aqueous $NaHCO_3$ (1 mL). The resulting biphasic mixture was separated, the organic phase was transferred to a PFE vessel, and concentrated by blowing down under a stream of argon to give crude (4-(1*H*-imidazole-1-carbonyl)piperazin-1-yl)(phenyl)methanone a colourless gum. LCMS showed over 80% purity at this stage and the resulting residue was carried forward into the next stage without further purification.

Telescoped Complete Analogue Synthesis – Step 2, Synthesis of 1-(4-Benzoylpiperazine-1-carbonyl)-3-methyl-1*H*-imidazol-3-ium iodide Under the Model Liquid Handler Conditions

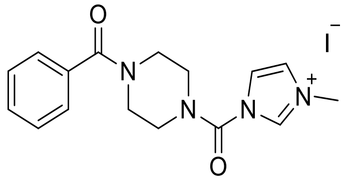

To the PFE reaction vessel containing crude (4-(1*H*-imidazole-1-carbonyl)piperazin-1-yl)(phenyl)methanone (0.036 mmol) was added acetonitrile (0.5 mL) and MeI (11 µL, 0.180 mmol). The resulting mixture was covered and left at ambient temperature for 16 h (not stirred). The resulting mixture was concentrated by blowing down under a stream of argon to give crude 1-(4-benzoylpiperazine-1-carbonyl)-3-methyl-1*H*-imidazol-3-ium iodide which was utilised in the subsequent steps without purification.

Telescoped Complete Analogue Synthesis – Step 3, Synthesis of *N*-Benzyl-4-(furan-2-carbonyl)piperazine-1-carboxamide Under the Model Liquid Handler Conditions

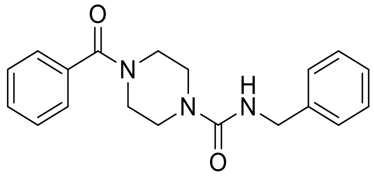

To the PFE reaction vessel containing crude 1-(4-benzoylpiperazine-1-carbonyl)-3-methyl-1*H*-imidazol-3-ium iodide (0.036 mmol) was added potassium carbonate (185 mg, 1.34 mmol), and 1,4-dioxane (4 mL). To the resulting mixture was added *N*-benzylamine (107 µL, 0.98 mmol). The resulting mixture was covered and left at ambient temperature for 16 h (not stirred). To the resulting mixture was added DCM (1 mL) and saturated aqueous $NaHCO_3$ (1 mL). The resulting biphasic mixture was separated, and the organic phase concentrated by blowing down under a stream of argon to give a cloudy gum. LCMS analysis at this stage showed the desired product as the major product (~85% purity) with unknown minor products.

4 Development of a Multiple Step Liquid-Handler Applicable Elaborated Sulphonamide Urea Synthesis (It 4)

1-(4-(*tert*-Butoxycarbonyl)piperazine-1-carbonyl)-3-methyl-1*H*-imidazol-3-ium iodide

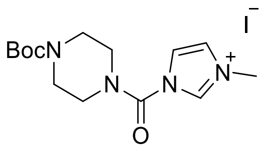

To a mixture of *tert*-butyl 4-(1*H*-imidazole-1-carbonyl)piperazine-1-carboxylate (250 mg, 0.891 mmol) and MeCN (2.5 mL) was added MeI (278 µL, 4.45 mmol). The resulting mixture was stirred at ambient temperature for 16 h. The resulting mixture was concentrated under reduced pressure to give 1-(4-(*tert*-butoxycarbonyl)piperazine-1-carbonyl)-3-methyl-1*H*-imidazol-3-ium iodide as a yellow solid (391 mg, 99%). ^{1}H NMR (600 MHz, d_6–DMSO): δ = 9.54 (1H, s), 8.00 (1H, m), 7.85 (1H, m), 3.91 (3H, s), 3.58-3.41 (8H, m), 1.42 (9H, s).

tert-Butyl 4-(piperidine-1-carbonyl)piperazine-1-carboxylate

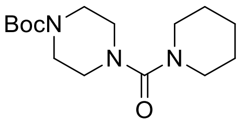

To a mixture of 1-(4-(*tert*-butoxycarbonyl)piperazine-1-carbonyl)-3-methyl-1*H*-imidazol-3-ium iodide (100 mg, 0.237 mmol), potassium carbonate (49 mg, 0.355 mmol), and DMF (1 mL) was added piperidine (26 µL, 0.261 mmol). The resulting mixture was stirred at ambient temperature for 16 h. To the mixture was added DCM (5 mL) and 2M aqueous HCl (5 mL). The resulting biphasic mixture was passed through a hydrophobic frit and the organic phase concentrated under reduced pressure to give *tert*-butyl 4-(piperidine-1-carbonyl)piperazine-1-carboxylate as a white solid (71 mg, 100%). ^{1}H NMR (600 MHz, d_6–DMSO): δ = 3.33-3.27 (4H, m), 3.11 (4H, t, J = 5.4 Hz), 3.08-3.03 (4H, m), 1.55-1.50 (2H, m), 1.49-1.43 (4H, m), 1.40 (9H, s).

tert-Butyl 4-(benzylcarbamoyl)piperazine-1-carboxylate

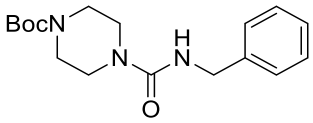

To a mixture of 1-(4-(*tert*-butoxycarbonyl)piperazine-1-carbonyl)-3-methyl-1*H*-imidazol-3-ium iodide (200 mg, 0.474 mmol), potassium carbonate (98 mg, 0.711 mmol), and DMF (2 mL) was added *N*-benzyl amine (57 µL, 0.522 mmol). The resulting mixture was stirred at ambient temperature for 16 h. To the mixture was added DCM (5 mL) and 2M aqueous HCl (5 mL). The resulting biphasic mixture was passed through a hydrophobic frit and the organic phase concentrated under reduced pressure to give *tert*-butyl 4-(benzylcarbamoyl)piperazine-1-carboxylate as a yellow solid (155 mg, 93%). ^{1}H NMR (600 MHz, d_6–DMSO): δ = 7.31-7.27 (2H, m), 7.26-7.23 (2H, m), 7.20 (1H, m), 7.12 (1H, m), 4.24 (2H, d, J = 5.7 Hz), 3.31-3.27 (8H, m), 1.41 (9H, s).

Piperazin-1-yl(piperidin-1-yl)methanone

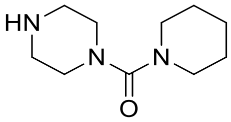

To a mixture of *tert*-butyl 4-(piperidine-1-carbonyl)piperazine-1-carboxylate (66 mg, 0.22 mmol) and DCM (1 mL) was added TFA (1 mL) and the resulting mixture was stirred for 16 h at ambient temperature. The reaction mixture was concentrated under reduced pressure to give a yellow gum of sufficient purity to be utilised in the subsequent reactions without further manipulation.

_N_-Benzylpiperazine-1-carboxamide hydrochloride

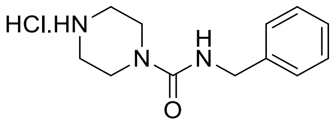

To a mixture of *tert*-butyl 4-(benzylcarbamoyl)piperazine-1-carboxylate (180 mg, 0.563 mmol) and 1,4-dioxane (1 mL) was added 4M HCl (in 1,4-dioxane) (1 mL). The resulting mixture was stirred at ambient temperature for 16 h. The reaction mixture was concentrated under reduced pressure to give *N*-benzylpiperazine-1-carboxamide hydrochloride as a yellow solid (133 mg, 92%). ^{1}H NMR (600 MHz, d_6–DMSO): δ = 9.19 (2H, s), 7.35-7.28 (3H, m), 7.27-7.27 (2H, m), 7.21 (1H, m), 4.24 (2H, d, J = 5.7 Hz), 3.59-3.53 (4H, m), 3.42-3.34 (4H, m).

Methyl 5-(piperidin-1-ylsulfonyl)furan-2-carboxylate

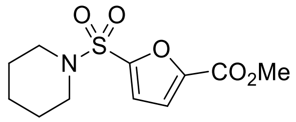

A mixture of methyl 5-(chlorosulfonyl)furan-2-carboxylate (225 mg, 1.00 mmol) and anhydrous THF (3 mL) was cooled to 0 °C. To the mixture was added dropwise piperidine (207 µL, 2.10 mmol) and the reaction warmed to ambient temperature and stirred for 24 h. To the mixture was added ethyl acetate (10 mL) and saturated aqueous $NaHCO_3$ solution (10 mL). The resulting biphasic mixture was separated and the aqueous phase extracted with ethyl acetate (3 x 10 mL). The combined organic extracts were washed with brine (20 mL), dried over anhydrous $MgSO_4$, filtered, and concentrated under reduced pressure to give methyl 5-(piperidin-1-ylsulfonyl)furan-2-carboxylate as an orange solid (222 mg, 81%). ^1H NMR (600 MHz, d_6–DMSO): δ = 7.48 (1H, d, J = 3.7 Hz), 7.32 (1H, d, J = 3.7 Hz), 3.87 (3H, s), 3.14-3.10 (4H, m), 1.56-1.51 (4H, m), 1.47-1.41 (2H, m).

5-(Piperidin-1-ylsulfonyl)furan-2-carboxylic acid

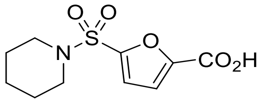

A mixture of methyl 5-(piperidin-1-ylsulfonyl)furan-2-carboxylate (100 mg, 0.366 mmol), LiOH.H_2O (18 mg, 0.439 mmol), and THF/H_2O (1:1) (3 mL) was stirred at ambient temperature for 16 h. The resulting mixture was concentrated under reduced pressure to give 5-(piperidin-1-ylsulfonyl)furan-2-carboxylic acid as an orange gum of sufficient purity to be utilised in the subsequent reactions without further manipulation.

***N*-Benzyl-4-(5-(piperidin-1-ylsulfonyl)furan-2-carbonyl)piperazine-1-carboxamide**

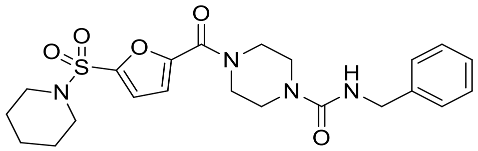

To a mixture of *N*-benzylpiperazine-1-carboxamide hydrochloride (25 mg, 0.094 mmol), 5-(piperidin-1-ylsulfonyl)furan-2-carboxylic acid (25 mg, 0.099 mmol), DIPEA (57 µL, 0.329 mmol), and DMA (1.5 mL) was added T3P (50% in EtOAc) (67 µL, 0.113 mmol). The resulting mixture was stirred at ambient temperature for 16 h. To the mixture was added DCM (2.5 mL) and saturated aqueous $NaHCO_3$ (2.5

mL). The resulting mixture was passed through a hydrophobic frit and concentrated under reduced pressure to give *N*-benzyl-4-(5-(piperidin-1-ylsulfonyl)furan-2-carbonyl)piperazine-1-carboxamide as a orange gum (30 mg). NMR analysis showed sufficient purity for screening, with low level unidentified impurities and some residual DMA.

Testing the Synthesis of Synthesis of Methyl 5-(morpholinosulfonyl)furan-2-carboxylate Under the Model Liquid Handler Conditions

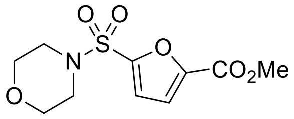

To a PFE reaction vessel was added methyl 5-(chlorosulfonyl)furan-2-carboxylate (22 mg, 0.10 mmol), morpholine (18 µL, 0.21 mmol), and THF (0.3 mL). The resulting mixture was covered and left at ambient temperature for 16 h (<u>not stirred</u>). To the resulting mixture was added DCM (0.5 mL) and saturated aqueous $NaHCO_3$ solution (0.5 mL). The mixture was separated, and the organic phase concentrated under a stream of argon to give a yellow solid. NMR analysis showed the desired product with trace levels of unknown impurities with sufficient purity to be used in subsequent steps if required.

Testing the Synthesis of Synthesis of Methyl 5-(morpholinosulfonyl)furan-2-carboxylate Under the Model Liquid Handler Conditions – Solvent Swap to DMA

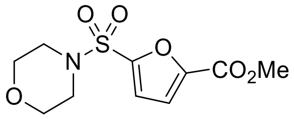

To a PFE reaction vessel was added methyl 5-(chlorosulfonyl)furan-2-carboxylate (22 mg, 0.10 mmol), morpholine (18 µL, 0.21 mmol), and DMA (0.3 mL). The resulting mixture was covered and left at ambient temperature for 16 h (<u>not stirred</u>). LCMS analysis at this stage showed complete conversion to the desired sulphonamide. The reaction was concluded at this stage.

Testing the Synthesis of Synthesis of 5-(Piperidin-1-ylsulfonyl)furan-2-carboxylic acid Under the Model Liquid Handler Conditions

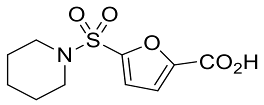

To a PFE reaction vessel was added a mixture of methyl 5-(piperidin-1-ylsulfonyl)furan-2-carboxylate (27 mg, 0.10 mmol), LiOH.H_2O (5 mg, 0.12 mmol), and THF/H_2O (1:1) (1 mL). The resulting mixture was covered and left at ambient temperature for 16 h (not stirred). The mixture was concentrated under a stream of argon to give a white solid. LCMS analysis of the solid showed complete hydrolysis of the ester.

Testing the Synthesis of *tert*-Butyl 4-(benzylcarbamoyl)piperazine-1-carboxylate Under the Model Liquid Handler Conditions

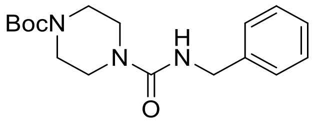

To a PFE reaction vessel was added 1-(4-(*tert*-butoxycarbonyl)piperazine-1-carbonyl)-3-methyl-1*H*-imidazol-3-ium iodide (26 mg, 0.062 mmol), potassium carbonate (13 mg, 0.093 mmol), *N*-benzyl amine (7 µL, 0.068 mmol), and DMF (0.5 mL). The resulting mixture was covered and left at ambient temperature for 16 h (not stirred). To the resulting mixture was added DCM (0.5 mL) and 2M aqueous HCl (0.5 mL). The mixture was separated, and the organic phase concentrated under a stream of argon. The resulting residue was of sufficient purity to be used in subsequent steps if required.

Sulphonamide Formation Solvent Screening Under Liquid Handling Conditions

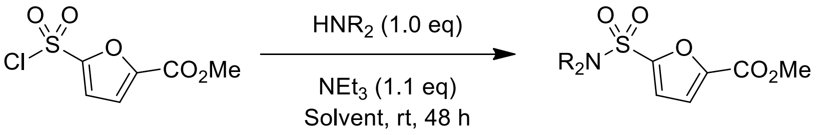

General Protocol:

To a PFE reaction vessel was added 5-(chlorosulfonyl)furan-2-carboxylate solution in solvent (0.33 M) (300 µL, 0.10 mmol) and triethylamine (15.3 µL, 0.11 mmol). To the vessel was added amine (0.10

mmol) and the resulting mixture was covered and left at ambient temperature for 48 h (not stirred). The resulting mixture was concentrated under a stream of argon and the resulting residue analysed by LCMS:

	Urea (% purity by LCMS)		
Solvent	Cyclopropylamine	Aniline	Morpholine
THF	76%	79%	46%
DMA	82%	70%	70%
1,4-Dioxane	79%	70%	70%
Toluene	66%	58%	74%
MeOH	0%	0%	0%

Hydrolysis Molarity Screening Under Liquid Handling Conditions

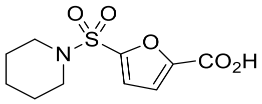

General Protocol:

To a PFE reaction vessel was added methyl 5-(piperidin-1-ylsulfonyl)furan-2-carboxylate solution in 1,4-dioxane (0.33 M) (150 µL, 0.05 mmol). To the mixture was LiOH solution (**X** M) (150 µL) and the resulting mixture was covered and left at ambient temperature (not stirred). The reaction mixtures were assessed by LCMS after 4 h and 16 h:

LiOH Molarity	% Hydrolysis after 4 h	% Hydrolysis after 16 h
1.00	95	99
0.50	82	97
0.10	11	25
0.05	4	11

5 Development of a Multiple Step Liquid-Handler Applicable Elaborated Urea Synthesis (It 3.5 and 4.5)

Solubility Assessment of 1-(4-(*tert*-Butoxycarbonyl)piperazine-1-carbonyl)-3-methyl-1*H*-imidazol-3-ium iodide

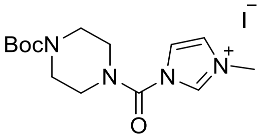

Testing the solubility of 1-(4-(*tert*-butoxycarbonyl)piperazine-1-carbonyl)-3-methyl-1*H*-imidazol-3-ium iodide in a variety of solvents under different conditions.

To 1-(4-(*tert*-butoxycarbonyl)piperazine-1-carbonyl)-3-methyl-1*H*-imidazol-3-ium iodide (20 mg, 0.047 mmol) was added solvent (150 µL). The resulting mixture was either; a) stirred at ambient temperature, b) sonicated, or c) heated to reflux and the solubility recorded:

Solvent	Stirred Solubility	Sonicated Solubility	Heated Solubility
DMA	low	low	complete
THF	none	none	none
1,4-Dioxane	trace	low	low
EtOAc	none	trace	trace
DCM	none	none	none
Toluene	none	none	none
MeCN	low	low	complete

Determination of the Approximate Maximum Molarity 1-(4-(*tert*-Butoxycarbonyl)piperazine-1-carbonyl)-3-methyl-1*H*-imidazol-3-ium iodide in MeCN, DMA, and DMSO

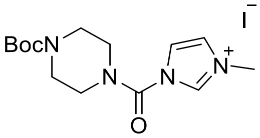

To 1-(4-(*tert*-butoxycarbonyl)piperazine-1-carbonyl)-3-methyl-1*H*-imidazol-3-ium iodide (20 mg, 0.047 mmol) was added sequentially solvent (150 µL) until complete dissolution was observed:

DMA = 6 x 150 µL = 900 µL = **0.052 M** solubility

MeCN = 10 x 150 µL = 1500 µL = **0.031 M** solubility

DMSO = 1 x 150 µL = 150 µL > **0.31 M** solubility

Testing the Synthesis of *tert*-butyl 4-(morpholine-4-carbonyl)piperazine-1-carboxylate in d_6-DMSO Under the Liquid Handling Conditions

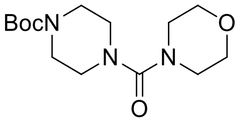

To a PFE reaction vessel containing a mixture of 1-(4-(*tert*-butoxycarbonyl)piperazine-1-carbonyl)-3-methyl-1*H*-imidazol-3-ium iodide (42 mg, 0.10 mmol), potassium carbonate (21 mg, 0.15 mmol), and d_6-DMSO (300 µL). To the mixture was added morpholine (8.80 µL, 0.10 mmol) and the resulting mixture was covered and left at ambient temperature (not stirred) for 16 h. The resulting mixture was filtered and analysed by NMR which showed complete conversion had occurred.

Testing the Synthesis of *tert*-butyl 4-(morpholine-4-carbonyl)piperazine-1-carboxylate in DMSO Under the Liquid Handling Conditions

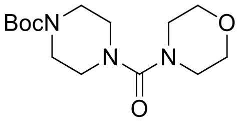

To a PFE reaction vessel containing a mixture of 1-(4-(*tert*-butoxycarbonyl)piperazine-1-carbonyl)-3-methyl-1*H*-imidazol-3-ium iodide (42 mg, 0.10 mmol), potassium carbonate (21 mg, 0.15 mmol), and DMSO (300 µL). To the mixture was added morpholine (8.80 µL, 0.10 mmol) and the resulting mixture was covered and left at ambient temperature (not stirred) for 16 h. To the resulting mixture was added DCM (600 µL) and 2M aqueous HCl (600 µL). The resulting biphasic mixture was separated, and the organic phase was concentrated under a stream of argon to give a pale yellow solid. NMR analysis showed no remaining aromatic signals and a single Boc signal consistent with the product and therefore the solid was utilised in subsequent reactions without further manipulation.

Testing the Synthesis of morpholino(piperazin-1-yl)methanone hydrochloride Under the Liquid Handling Conditions

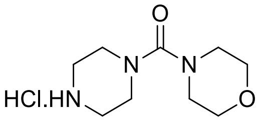

To a PFE reaction vessel was added a solution of crude *tert*-butyl 4-(morpholine-4-carbonyl)piperazine-1-carboxylate (0.10 mmol) in 1,4-dioxane (300 µL) followed by 4M HCl (in 1,4-

dioxane) (300 µL). The resulting mixture was covered and left at ambient temperature (not stirred) for 16 h. The resulting mixture was concentrated under a stream of argon to give an orange oil. NMR analysis showed the desired product with trace levels of impurities and therefore this material could be utilised in subsequent reactions without any purification.

6 Synthesis of Pure Hit Compounds as Positive Controls and For Further Testing

1-(4-(Furan-2-carbonyl)piperazine-1-carbonyl)-3-methyl-1*H*-imidazol-3-ium iodide

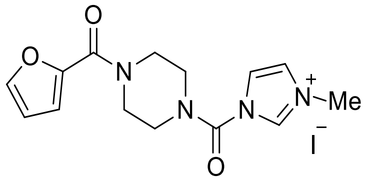

To a mixture of (4-(1*H*-imidazole-1-carbonyl)piperazin-1-yl)(furan-2-yl)methanone (1.35 g, 4.92 mmol) and acetonitrile (25 mL) was added methyl iodide (1.53 mL, 24.60 mmol) and the resulting mixture was stirred for 16 h at ambient temperature. The resulting mixture was concentrated under reduced pressure to give an off-white solid which was used in the subsequent stages without any further purification.

General Procedure for the formation of ureas from 1-(4-(furan-2-carbonyl)piperazine-1-carbonyl)-3-methyl-1*H*-imidazol-3-ium iodide

A mixture of 1-(4-(furan-2-carbonyl)piperazine-1-carbonyl)-3-methyl-1*H*-imidazol-3-ium iodide (512 mg, 1.23 mmol), amine (1.35 mmol), potassium carbonate (257 mg, 1.85 mmol), and DMF (5 mL) was stirred for 16 h at ambient temperature. To the mixture was added 2M aqueous HCl (5 mL) and DCM (10 mL). The resulting biphasic mixture was passed through a hydrophobic frit and the organic phase concentrated under reduced pressure to give the crude urea.

4-(Furan-2-carbonyl)-*N*-(2-methoxyethyl)piperazine-1-carboxamide

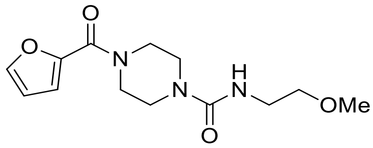

Preformed under the general protocol with 2-methoxyethanamine (118 µL, 1.35 mmol) to give the crude as a yellow solid (218 mg). The resulting solid was purified by automated flash column chromatography (*n*-hexane/EtOAc, 100:0 – 0:100, 30 g SiO_2). The appropriate fractions were combined and concentrated under reduced pressure to give 4-(furan-2-carbonyl)-*N*-(2-methoxyethyl)piperazine-1-carboxamide as a yellow gum (145 mg, 42%). ^{1}H NMR (600 MHz, d_6–DMSO): δ = 7.84 (1H, m), 7.01 (1H, d, J = 3.6 Hz), 6.65-6.60 (2H, m), 3.70-3.56 (4H, m), 3.39-3.31 (8H, m), 3.23 (3H, s), 3.19 (2H, q, J = 5.9 Hz). ^{13}C NMR (151 MHz, d_6–DMSO): δ = 158.4, 157.2, 146.9, 144.8, 115.7, 111.3, 71.6, 71.2, 57.9, 43.4, 39.8.

***N*-(Cyclopropylmethyl)-4-(furan-2-carbonyl)piperazine-1-carboxamide**

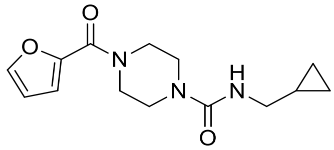

Preformed under the general protocol with cyclopropylmethanamine (117 µL, 1.35 mmol) to give the crude as a yellow solid (214 mg). The resulting solid was purified by automated flash column chromatography (*n*-hexane/EtOAc, 100:0 – 0:100, 30 g SiO_2). The appropriate fractions were combined and concentrated under reduced pressure to give *N*-(cyclopropylmethyl)-4-(furan-2-carbonyl)piperazine-1-carboxamide as an off-white solid (202 mg, 59%). ^{1}H NMR (600 MHz, d_6–DMSO): δ = 7.84 (1H, m), 7.01 (1H, dd, J = 3.5, 0.8 Hz), 6.66-6.61 (2H, m), 3.70-3.57 (4H, m), 3.40-3.35 (4H, m), 2.91 (2H, dd, J = 6.7, 5.4 Hz), 0.93 (1H, m), 0.39-0.35 (2H, m), 0.16-0.13 (2H, m).

4-(Furan-2-carbonyl)-*N*-(thiophen-2-ylmethyl)piperazine-1-carboxamide

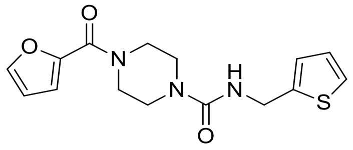

Preformed under the general protocol with thiophen-2-ylmethanamine (139 µL, 1.35 mmol) to give the crude as a brown solid (340 mg). The resulting solid was purified by automated flash column chromatography (*n*-hexane/EtOAc, 100:0 – 85:15, 30 g SiO_2). The appropriate fractions were combined and concentrated under reduced pressure to give 4-(furan-2-carbonyl)-*N*-(thiophen-2-ylmethyl)piperazine-1-carboxamide (243 mg, 62%). ^{1}H NMR (600 MHz, d_6–DMSO): δ = 7.84 (1H, m), 7.34 (1H, dd, J = 4.7, 1.7 Hz), 7.26 (1H, m), 7.01 (1H, dd, J = 3.5, 0.8 Hz), 6.95-6.92 (2H, m), 6.63 (1H, dd, J = 3.5, 1.8 Hz), 4.40 (2H, d, J = 5.7 Hz), 3.70-3.57 (4H, m), 3.43-3.38 (4H, m).

4-(Furan-2-carbonyl)-*N*-(2,2,2-trifluoroethyl)piperazine-1-carboxamide

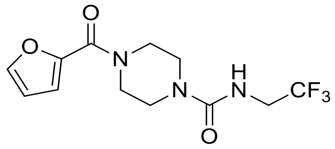

Preformed under the general protocol with 2,2,2-trifluoroethanamine (108 µL, 1.35 mmol) to give the crude as a yellow oil (290 mg). The resulting solid was purified by automated flash column chromatography (*n*-hexane/EtOAc, 100:0 – 85:15, 30 g SiO_2). The appropriate fractions were combined and concentrated under reduced pressure to give 4-(furan-2-carbonyl)-*N*-(2,2,2-trifluoroethyl)piperazine-1-carboxamide as a light yellow solid (214 mg, 57%). ^{1}H NMR (600 MHz, d_6–DMSO): δ = 7.85 (1H, m), 7.23 (1H, t, J = 6.2 Hz), 7.02 (1H, dd, J = 3.5, 0.9 Hz), 6.63 (1H, dd, J = 3.5, 1.7 Hz), 3.84 (2H, dq, J = 9.8, 6.1 Hz), 3.73-3.58 (4H, m), 3.46-3.40 (4H, m).

7 Robotic synthesis – experimental and robotic protocols

Python scripts uses to execute the OpenTrons – see sections titled “Robotic steps protocols” – can be found at 10.5281/zenodo.7586212

Consumables list

24-Rack Fluidx 24 rack – 4mL glass capped vials with barcode starting material stock solution

2ml-96-well plate: 2mL square, round bottom 96-well, PP. Used as reaction rack and work-up (Starlab S1896-2800)

96-well plate: 96-well plate, PP, 320ul (Starlab S1833-9600) used for QC plates

12- and 4-trough: 12-, 4-well trough, PP, Starlab, were used for the transfer larger quantities (10 – 40 ml) of building blocks.

The reaction racks were sealed using plastic mats in between operations, or aluminium seals. (Starlab E2896-1801 and B1494-1124)

384-plate: Labcyte 384PP plate used as screening plates (XChem) (Labcyte P-05525)

SPE DRY (Biotage) equipped with a 96-head

Opentrons OT1 (1mL eppendorf single channel and 300 uL 8-channel D-Lab)

LCMS Agilent 1260 Infinity series, with a 6120 Agilent MS quadripole and ELSD (1260)

7.1 Iteration 1.0 and 1.1 Urea reaction

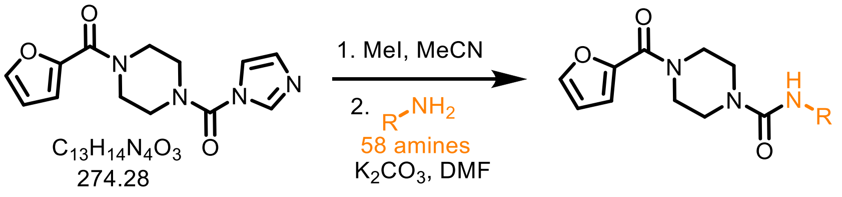

Iteration 1.0

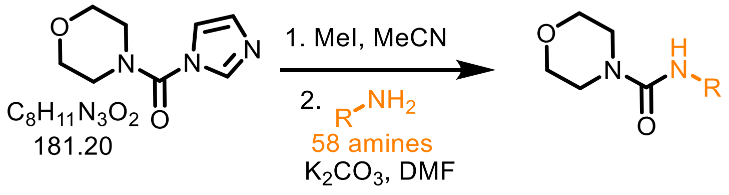

Iteration 1.1

Scheme S1 – Urea synthesis for iterations 1.0 and 1.1

Experimental details

Iteration 1.0 - Imidazole 1((4-(1H-imidazole-1-carbonyl)piperazin-1-yl)(furan-2-yl)methanone) was dissolved in MeCN (500 mM) and dispensed (0.06 mmol, 120uL) in 58 wells of two 96-well reaction racks (StarLab 2mL). Iodomethane (15 eq, 56uL) was added in each well and reaction rack was

covered with a plastic mat. After 24 hours the mixture was concentrated (SPE-Dry, heated nitrogen 96-head dispenser) and K_2CO_3 (>1.5 eq, >12.4 mg) was added. 58 amines in DMF (1.1 eq, 0.067 mmol, 132 uL from a 500mM stock) were dispensed in each of the mixture containing wells followed by the addition of a further volume of DMF (132 uL). The mixture was concentrated after 24 hours (SPE-dry) and partitioned in DCM (300uL) and an aqueous solution (3M HCl, 300uL). The organic extracts were transferred (250uL) to a 96-well plate (Starlab, 300uL) and the DCM removed overnight. DMSO was added (189uL, 250 mM) to dissolve the crude reaction mixtures and make a stock plate used to prepare one QC plate (LCMS) and two screening plates (in dmso and ethylene glycol).

*15uL were taken off the reaction mixture before workup for QC check.

Iteration 1.1 - (1H-imidazol-1-yl)(morpholino)methanone was dissolved in MeCN (1000 mM) and dispensed (0.06 mmol, 60uL) in 58 wells of two 96-well reaction racks (StarLab 2mL). The rest of the procedure was identical to iteration 1.0.

Robotic steps protocols

01_Urea_stocksolution_intermediate_t1 #combined
02_Urea_stocksolution_Intermediate1_transfer_t1.py # separate
03_Urea_MeCN_MeI_addition_intermediate1&2.py # combined
04_Urea_stocksolution_amines.py
05_Urea_amine_addition_int1&2.py # combined
06_Urea_QC_96to96_multichannel.py # separate
07a_Urea_workup.py & 07b_Urea_more_mixing_wu.py # separate
08_pWUpBottomPhaseTransfer_96to96_multi300.py # separate
09_pWUpScreenSolventDispensing_troughTo96_multi300.py # separate
10_pWUpQC_Screen_96to96_96to384_troughTo96_multi300_int1.py # separate
11_pWUpQC_Screen__96to384__multi300_int1.py # separate
12_pWUpQC_AddEG_troughTo96_multi300_int1.py # separate

Robotic steps comment

Step 1 - Stock solution for piperazine and morpholine building blocks (MeCN as solvent)
The building blocks, powders in a 4- trough were dissolved to the required concentration and transferred to storage vials (24-rack)

Step 2 - transfer of piperazine and morpholine building blocks in the reaction wells (2 × 2ml-96-well plates)

Step 3 - Addition of MeCN and MeI in both 2ml-96-well

Step 4 - Stock solution amines (DMF used as solvent) in storage vials (24-rack)

à MeCN removal in 2ml-96-well plates (SPE DRY)

à Manual addition of K_2CO_3 powder

Step 5 - Addition of a amines to both imidazolium salts in 2ml-96-well plates

Step 6 - Transfer of a reaction mixture aliquot to 96-well plate after 1.5 h.

à DMF removal in 2ml-96-well plates (SPE DRY)

Step 7 - Reaction workup (organic/aqueous)
Addition of an aqueous solution (3M HCl), then organic solvent (DCM). The heterogeneous mixture is mixed using repeated $_{aspiration}$/dispense with the multichannel pipette.

Step 8 - post workup bottom phase transfer to 96-well plate. The organic phase is taken out of the biphasic mixture and dispensed in 96-well plates.

à DCM removal in 96-well plates (SPE DRY)

Step 9 - post workup solvent dispensing (DMSO) to residual crude mixtures in 96-well plates

Step 10 - post workup transfer to 96-well plates (LCMS) and 384PP-screening plate. The same 384 plate is used for both intermediate (using A C E.. for pipearazine containing mixtures and B D F..for morpholine containing mixtures.)

Step 11 – second post workup transfer of an aliquot to a 384PP-screening plate. This screen plate is then concentrated and EG is added.

à DMSO removal in 96-well plates (SPE DRY)

Step 12 - post workup addition of EG in 384-screening plate

All crude reaction mixtures are ready to be screened in 2 plates (DMSO and EG)

7.2 Iteration 2.0 Coupling reaction

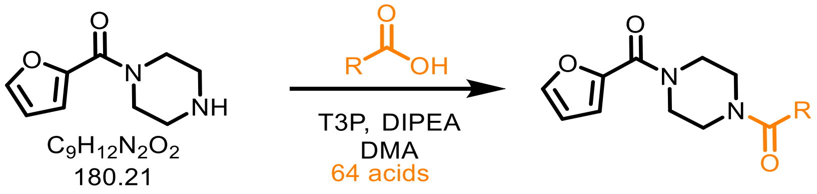

Iteration 2.0

Scheme S2 –Amidation reaction for iteration 2.0

Experimental details

Iteration 2.0 – T3P(50% in EtOAc, 47 uL, 0.07 mmol, 1.2 eq) and DIPEA (43uL, 0.27 mmol, 4.5 eq) were added in a 2mL-96-well-plate (9 rows). Next, 64 acids in DMA (0.5 M, 126uL, 0.06 mmol, 1.05 eq) were added to the plate followed by furan-2-yl(piperazin-1-yl)methanone in DMA (1.5 M, 40uL, 0.06 mmol, 1.0 eq) and the reaction rack was covered with a plastic mat. The mixture was concentrated after 24 hours (SPE-dry) and partitioned in DCM (300uL) and an aqueous solution (satNH_4Cl-H_2O, 1:1, 300uL). The organic extracts were transferred (250uL) to a 96-well plate (Starlab, 300uL) and the DCM removed overnight. EG was added (240 uL, 200 mM) to dissolve the crude reaction mixtures and make a stock plate used to prepare QC plate (LCMS) and screening plate.

*15uL were taken off the reaction mixture before workup for QC check.

Robotic steps protocols

01_Coupling_SC1000_mainReactant_stocksolution_vialStorage
02_Coupling_SC1000_reactants_stocksolution_trough_vialstorage.py
03_Coupling_MC300_base_couplingAgent_troughTo96.py

04_Coupling_SC1000_reactants_addition_storage_96.py
05_Coupling_SC1000_mainReactant_transfer_96.py
06_coupling_QC_96to96_multichannel.py
07_workup_96.py
08_pWUpBottomPhaseTransfer_96to96_multi300.py
09_ADD_EG_trough_96.py
10_pWUpQC_Screen_96to96_96to384_troughTo96_multi300.py

Robotic steps comment

Step 1 - Stock solution for the main reactant, furan-piperazine. Reactant is in a 4-well trough and DMA added. Once added, and diluted, the solution is transferred to storage vials (24-rack) capped after transfer.

Step 2 - Stock solution for the other reactants, here 64 acids. Acids are already in vials in 24-racks and DMA is added.

Step 3 - Addition of T3P and base. Reagents are in a 12-trough and dispensed in a 96-reaction rack.

Step 4 - Addition of the carboxylic acids in 24-racks to the T3P/base in 96-well reaction rack.

Step 5 - Addition of the furan-piperazine in 12-trough to 96-well reaction rack.

Step 6 - Pre workup QC - 15ul of each reaction mixture transfered to a small 96-well plate for LCMS

Step 7 - Work-up - aqueous solution and organic solvent added to 96-reaction rack and mixing using repeated aspiration/dispense.

Step 8 - Post work up extraction of organic phase (bottom phase), from 96-reaction rack to small 96-well rack

Step 9 - Add ethylene glycol (screening solvent) to small 96-well plate

Step 10 - transfer to QC (20ul) and screen plate.

7.3 Iteration 3.5 Triple reaction sequence

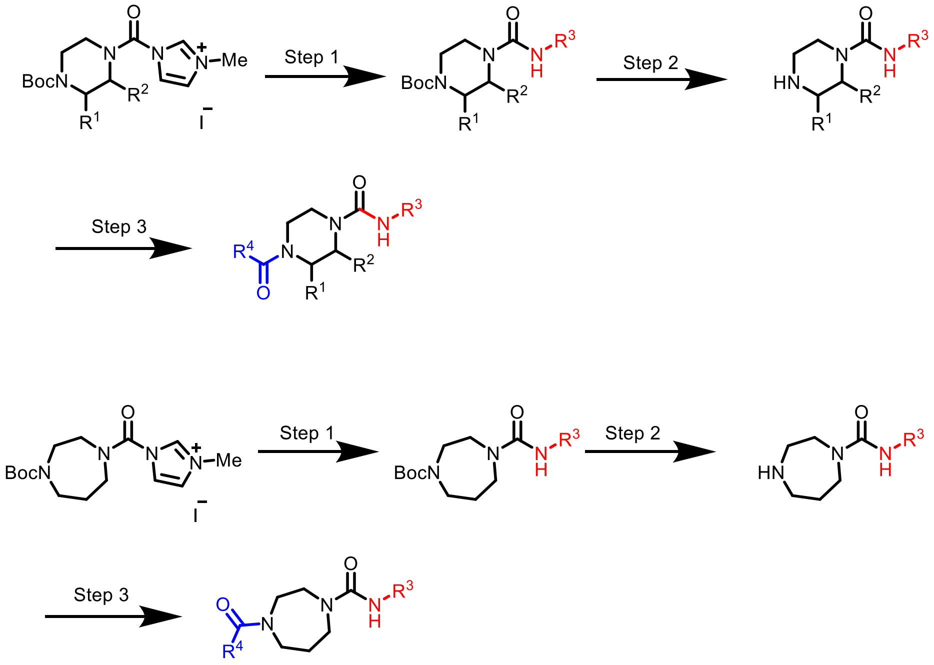

Iteration 3.5

Scheme S3 – Urea, boc-deprotection and amidation reactions for iteration 3.5

Experimental details

Iteration 3.5 – 8 amines (263 uL, 0.131 mmol, 1.05 eq, 500 mM in DMA) were dispensed in four 2ml 96-well reaction plates, each amine transferred across the 8 wells of a column containing K_2CO_3 (25.9mg, 0.187 mmol, 1.5 eq). Next 4 Imidazolium salts (250 uL, 0.125, 1.00 eq, 500mM in DMSO[a]) were dispensed, one per plate and across the 64 amine containing wells. The reaction plates were sealed and stored overnight at rt. The reaction mixtures were concentrated after 24 hours (SPE-dry) and partitioned in DCM (300uL) and an aqueous solution (3M aqueous HCl or 50% brine solution[b], 300uL). The organic extracts were transferred (260uL) to a 96-well plate (Starlab, 300uL) and each aqueous phase was extracted a second time with DCM (300uL). The organic extracts (260uL) were added to the 96-well plate containing the first extracts and the DCM was removed overnight. 1,4-Dioxane (1200 uL) was added to each crude residue and upon complete dilution, each crude mixture was dispensed (290 uL, 0.030 mmol) in 4 wells, across 12 reaction plates, 3 plates per imidazole (e.g. series). 4M HCl in 1,4-dioxane (200 µL, excess) was added to each reaction wells to each of the three-reaction plates. The reaction plates were sealed and stored at ambient temperature for 16 h. The resulting mixtures were concentrated under reduced pressure using the blowdown apparatus. DMA (500uL) was added to dilute the crude residues before the addition of 32 acids (52 uL, 0.031 mmol, 1.04 eq, 600mM in DMA) in each reaction well followed by T3P (22.1 ul, 1.2 eq, 50% in EtOAc) and DIPEA (24.3 uL, 4.5 eq). The 12 reaction plates containing 1024 amide coupling reactions were sealed and stored at ambient temperature for 72 hours. The reaction mixtures were concentrated partitioned in DCM (300uL) and an aqueous saturated solution of $NaHCO_3$ (300uL). The organic extracts were transferred (210uL) to a 96-well plate (Starlab, 300uL) and each aqueouse phase was extracted a second time with DCM (300uL). The organic extracts (260uL) were added to the 96-well plate containing the first extracts and the DCM was removed overnight. MeOH (155 µL) was added and an aliquot (15 uL) of each solution was transferred to 96-well low volume plates for LCMS analysis. MeOH

was then removed (SPE-Dry) and EG (140ul, 200 mM) added. An aliquot (30 uL) of the 1024 reactions was then transferred to 384PP screening plates.

[a]Imidazolium 1 stock was 250mM hence 500uL was added instead.

[b]Brine was added instead of HCl for ureas contining basic sites

Robotic steps protocols

01_1_P1000s_stockSolutions_trough_or_vialRack_acids.py
01_2_P1000s_stockSolutions_trough_or_vialRack _amines.py
01_3_P1000s_stockSolutions_trough_or_vialRack_imidazoliums.py
01_4_P1000s_stockSolutions_trough_or_vialRack_MoreDMSO.py
02_P300m_reactant1_troughTo96rack.py
03_1_P300m_reactant2addition_troughTo96rack.py
03_2_P300m_reactant2addition_troughTo96rack.py
03_3_P300m_reactant2addition_troughTo96rack.py
03_4_P300m_reactant2addition_troughTo96rack_Imid1only.py
04_2_1_ureas_workup_96_mix.py
04_2_2_ureas_workup_96_mix_exothermic.py
04_3_pWupBottomPhaseTransfer_96to96_multi300.py
04_4_ureas_workup_96_extration2_addDCM.py
04_5_ureas_workup_96_mix_extraction2.py
04_6_pWupBottomPhaseTransfer_96to96_multi300 .py
05_1_P300m_urea_dioxane_addition_and_mix_troughTo96.py
05_2_P300m_OPTIONAL_urea_more_mix_troughTo96.py
06_it3-5-P300m_split_96_to_3_96_four_row_identical.py
07_1_P300m_HCl-dioxane_reagent4_troughTo96_perplate_plate_1.py
07_2_P300m_HCl-dioxane_reagent4_troughTo96_perplate_plate_2.py
07_3_P300m_HCl-dioxane_reagent4_troughTo96_perplate_plate_3.py
08_1_it3-5-P300m_add_solvent_in_3_plates.py
08_2_it3-5-P1000s_reactant2_addition_on_3_plate.py
08_3_it3-5-P300m_add_reagent_1_and_2_in_3_plates.py
09_1_3.5_workup_all_in_one.py
09_2_3.5__workup_96_mix_exothermic_plate1.py
09_3_3.5_workup_BottomPhaseTransfer_96to96_plate1.py
09_4_3.5_workup_96_extration_addDCM_all_in_one.py
09_5_3.5_workup_96_mix_even_shorter_plate_1.py
09_6_3.5_workup_BottomPhaseTransfer_96to96_plate1.py
10_3.5_add_MeOH_all_in_one.py
11_3.5_96_short_mix_plate_1.py
12_3.5_prepare_QC_plate_addMeCN_waterQC_all_in_one.py
13_3.5_transfer_15_to_QC_plate_1.py
14_3.5_add_EG_all_in_one.py
15_3.5_transfer_30_to_384PP_screen_plate_1.py

Robotic steps

Protocol 01-1 – Preparation of Carboxylic Acid Stock Solutions for Step 3
Preparation of 600 mM solutions in DMA of the carboxylic acids to be used in Step 3 (**Scheme S3**) and Step 3 (**Scheme S4**). Use of the liquid handling platform to add DMA (Location C2, Well A4) to known amounts of the carboxylic acids (Location A1 Opentrons Deck) using the 1000 µL single channel pipette.
Protocol was successful, two pauses to refill the solvent reservoir.

Protocol 01-2 – Preparation of Amine/Aniline Stock Solutions for Step 1
Preparation of 500 mM solutions in DMA of the amines/anilines to be used in Step 1. Use of the liquid handling platform to add DMA (Location C2, Well A4) to individual troughs for the amine/aniline stock solutions (Location C1, Rows 1-8) using the 1000 µL single channel pipette.
To avoid any potential issues with volatility the liquid amines were added after the addition of DMA and the plate sealed and stored until required.
Protocol was successful apart from amine 4, the presence of commas in the name resulted in an incorrect amount of DMA being added. This was manually corrected and amended on the spreadsheet.
Protocol 01-2 was repeated for Series 1b, due to the failure of Series 1 (unsubstituted piperazine core, *Cf Protocol 04-02 – Mixing the Organic and Aqueous Phases*)

Protocol: 3.5.2_01_2_P1000s_stockSolutions_trough_or_vialRack_amines.py - OK

Protocol 01-3 – Preparation of Imidazolium Salt Stock Solutions
Preparation of 500 mM solutions of the four different imidazolium salts in DMSO to be used in Step 1. Use of the liquid handling platform to add DMSO (Location C2, Well A4) to pre-weighed samples of the four different imidazolium salts (Location C2) using the 1000 µL single channel pipette.
Protocol was successful with no issues. The unsubstituted imidazolium salt (R1 = R2 = H) was only partially soluble, therefore an additional solvent addition protocol was required (see below).

Protocol 01-3 was repeated for Series 1b, due to the failure of Series 1 (unsubstituted piperazine core, *Cf Protocol 04-02 – Mixing the Organic and Aqueous Phases*).

Protocol: 3.5.2_01_2_P1000s_stockSolutions_trough_or_vialRack_imidazolium.py.

Run 1: Incorrect protocol was run adding 12 mL of solvent instead of 36.8 mL. The csv file was amended, and the protocol rerun to add the remaining solvent (24.8 mL).

Protocol 01-4 – Addition of More Solvent to the Piperazine Imidazolium Salt (R1=R2=H)
Repeat of the previous protocol with just the piperazine imidazole to produce a homogeneous solution. Addition of DMSO (Location C2, Well A4) to the partially soluble piperazine DMSO solution (Location C1, Well A2) to produce a 250 mM solution (a 100 mg test indicated solubility at this molarity). Protocol was an amended/repeat of the previous protocol, with the volume added to the other salts were set as zero on the csv file. Procedure was successful with complete solubility.

NOTE: Due to poorer solubility, concentration for Imidazole 1 (series 1, 250 mM) stock solution was half of the other 3 Imidazole stock solutions series (2 to 4, 500 mM)

Protocols 02 – Transfer of the Amine Stock Solutions to the 96-well reaction plates

Transfer of the eight amine stock solutions (Location E2) to one reaction plate (one amine per row, eight wells per amine) containing the potassium carbonate (Location D1) with the 8-channel 300 µL pipette. This protocol is repeated three times for each imidazolium salts resulting in the preparation of four plates containing eight amines in eight rows.

Run 1. Protocol successful. One channel (channel 3) was found to take up a slightly lower volume than the others, therefore the pipette was recalibrated after this stage.

Run 2. Protocol was successful. No issues after recalibration of the pipette.

Run 3. Protocol was successful.

Run 4. Protocol was stopped for recalibration after the first amine, concerns that due to the low levels of remaining amine stock solution the pipette was too high to take a sufficient volume. Run 4 repeated was successful.

Protocol 02 was repeated for Series 1b, due to the failure of Series 1 (unsubstituted piperazine core, *Cf Protocol 04-02 – Mixing the Organic and Aqueous Phases*).

Protocol 3.5.2_02_P300m_reactant1_troughTo96rack.py

Run 1: protocol successful

Protocols 03 – Transfer of the Imidazolium Salt to the Reaction Plate

Addition of the imidazolium stock solutions to one reaction plate containing the amine and potassium carbonate with the 8-channel 300 µL pipette. After addition, the reaction plate was sealed and stored at ambient temperature for 16 h. The resulting reaction mixtures were concentrated under blowdown conditions (SPE-Dry) in preparation for the work-up protocols. The protocol war run four times, one plate per imidazolium salt. **At the end of that stage, 32 ureas reactions were setup, each plate containing 8 amines, one per row, and 1 imidazolium salt. Each reaction is divided between the 8 wells constituting a row.**

Run 1. Series 2 - Protocol was successful, the other stock solutions in the troughs were covered to prevent any cross contamination from any dripping as the robotic arm moved.

Run 2. Series 3 - Protocol was successful

Run 3. Series 4 - Protocol was successful

Run 4. Series 1 - Due to the previous solubility issues double the volume of stock reagent was added to retain the correct molarity. (Protocol *_Imid1only variant*)

Protocol 03 was repeated for Series 1b, due to the failure of Series 1 (unsubstituted piperazine core, *Cf Protocol 04-02 – Mixing the Organic and Aqueous Phases*).

Protocol 3.5.2_03_P300m_reactant2addition_troughTo96rack.py

Run 1: protocol successful

Protocols 04 – Workup

Workup was accomplished using six consecutive protocols per series

After transfer, the combined DCM extracts (one plate per series) were concentrated using blowdown conditions (SPE-dry) in preparation for step 2.

Protocol 04-01 – Addition of DCM and the Aqueous Phase to the Reaction Plate

Addition of DCM (300uL) and either 3M aqueous HCl or 50% brine solution (300uL) depending on the expected ureas (e.g. picolyl-derived urea with a basic site was worked up using brine) using the 8-channel 300 µL pipette.

Series 4. Protocol was successful.

Series 2. Protocol was successful.

Series 3. Protocol was successful.

Series 1. Protocol was successful.

Series 1_bis. Protocol used: 3.5.2_04_1_a_Ureas_workup_96_addition_orga_and_aqueous_base_or_neutral.py - Protocol was successful with no excessive reaction between the acid and the undissolved base. Solid was still observed in some of the wells and therefore a slow mixing protocol was employed (See below) to prevent excessive bubbling and cross contamination.

Protocol 04-02 – Mixing the Organic and Aqueous Phases

Mixing the organic and aqueous phases through a series of dispensing and aspiration (300 uL each) using the 8-channel 300 µL pipette.

Series 4. Protocol was successful, however, the mixing appeared insufficient and therefore was repeated after lowering the height of the pipette (to ensure a mixture of both phases was taken up). Repeat was successful with excellent mixing of both phases of the work-up media.

Series 2. Protocol was successful.

Series 3. Protocol was successful.

Series 1. Protocol was started and stopped four times due to vigorous reaction upon mixing and concerns that this would lead to cross contamination between rows. Lower volumes aspiration, pause between aspiration and dispense were investigated but ultimately this series was terminated and started again with a modified work/up protocol. It was suspected that the amount of solid K_2CO_3 added was higher than for the three other series.

Series 1_bis. 3.5.2_04_2_Ureas_workup_96_mix_exothermic.py. Slow mixing of the DCM and aqueous phases. No excessive reaction/bubbling was observed. Some solids remained in the reaction wells and therefore an additional 300 µL of the appropriate aqueous media was added using protocol 3.5.2_04_1_b_Ureas_workup_96_addition_more_acid_aqueous.py. No excessive bubbling was observed. This time the mixing occurred at two different height, according to the following protocol: 3.5.2_04_2_test_Ureas_workup_96_mix_two_heights.py. All remaining solids were dissolved.

Protocol 04-03 – Removal of the Bottom DCM Phase

Removal of the bottom DCM phase (260 µL) and transfer to the reaction plate for Step 2 (Boc deprotection) using the 8-channel 300 µL pipette.

Series 4. Protocol was successful with no visible transfer of the aqueous phase to the reaction plate.

Series 2. Protocol was successful.

Series 3. Protocol was successful.

Series 1_bis. Protocol 3.5.2_04_4_Ureas_BottomPhaseTransfer_96to96_multi300.py was successful with minor water ransfer in some transfers.

Protocol 04-04 – Addition of a Further 300 µL of DCM to the remaining aqueous mixture

Addition of a further 300 µL of DCM to the reaction plate from step 1 containing the aqueous phase after the first extraction.

Series 4. Protocol was successful.

Series 2. Protocol was successful.

Series 3. Protocol was successful.

Series 1_bis. Protocol 3.5.2_04_5_Ureas_workup_96_extration_addDCM.py was successful

Protocol 04-05 – Mixing Protocol for the Second Extraction

Repeat of the mixing the organic and aqueous phases through a series of dispensing and aspiration with the 8-channel 300 µL pipette.

Series 4. Protocol was successful.

Series 2. Protocol was successful.

Series 3. Protocol was successful.

Series 1_bis. Protocol 3.5.2_04_6_Ureas_workup_96_mix_even_shorter.py was successful

Protocol 04-06 – Removal of the Bottom DCM Phase for the Second Extraction

Removal of the bottom DCM phase (300 µL, then 260 uL) and transfer to the reaction plate for Step 2 (Boc deprotection) with the 8-channel 300 µL pipette.

Series 4. Protocol was successful but may have been minimal water transfer in a few instances. Will be adjusted to 260 µL in the other three series.

Series 2. Protocol was successful. 260 uL aspiration meant no aqeous solution was transferred.

Series 3. Protocol was successful.

Series 1_bis. Protocol 3.5.2_04_7_Ureas_second_BottomPhaseTransfer_96to96_multi300.py was successful

Protocols 05

The aim of the robotic protocols in 05 is to dilute the crude residual mixtures from step 1 in MeCN to be able to transfer them in several reaction plates used to set up both reaction 2 and 3.

Experimentation using an initial volume of **600 uL** 1,4-Dioxane and various mixing strategies showed that for full dissolution fo the residues, **1200 uL** 1,4-dioxane was necessary and the following protocols required.

Protocol 05-01 – Addition of 1,4-Dioxane and mix to dissolve the crude ureas

1200 uL were added using the 8-channel 300 µL pipette in the mixture-containing rows of each reaction plate. Each solution was then mixed 10 times, by successive aspiration/dispense of 300 uL.

Series 4. Addition and mixing were done separately to find the optimal conditions. The protocol was successful, and no residues remained undissolved in the bottom of the well.

Series 2. The protocol was successful, however, during the mixing stage the pipette did not go low enough to agitate the solid residues.

Series 3. The protocol was successful at the lower mixing height (1.5 mm from the base), sonication to completely dissolve any remaining residues.

Series 1_bis. Protocol 3.5.2_05_1_P300m_urea_dioxane_addition_and_mix_troughTo96.py was successful. the resulting mixtures were sonicated to ensure complete dissolution of the residues.

Protocol 05-02 – Optional mixing of the solutions through a series of 300 µL aspiration and dispensing

Series 4. Not necessary

Series 2. Three additional mixing procedures were required, by lowering the pipette tip mixing position.

Series 3. Not necessary

Series 1_bis. Not necessary

Protocol 06 – Addition of the Boc-Ureas solutions in 1,4-Dioxane to 3 96-well reaction plates

Slightly more complex transfer. Per series, the eight crude ureas are dipensed in 256 wells contained in three 96-well plates. At the end of protocols 05, 12 reaction plates will contain the amount required of 32 ureas to start step 2 and 3. The pattern is the following. A quarter of each source row (290 uL) is taken out of and dispensed in four consecutive destination rows. Hence eight ureas are scattered across 32 rows and 3 96-well plates.

Series 4. Slight issues with one channel of the pipette sticking occasionally. The pipette was taken apart after the protocol and greased.

Series 2. Pipette calibration issue on the first run, stopped after the first three ureas for recalibration. Second run was ok; the pipette tips were not replaced for the first three ureas which had already been dispensed.

Series 3. Protocol successful

Series 1. Protocol 3.5.2_05_3_P300m_split_96_to_3_96_four_row_identical.py was successful, some isolated instances when a channel from the pipette stuck during the transfer. These wells were manually topped up and the pipette re-greased after this stage.

Protocol 07 - Addition of 4M HCl in 1,4-Dioxane to the reaction plates

Addition of 4M HCl in 1,4-dioxane (200 µL) to each of the three reaction plate. After addition, the reaction vessels were sealed and stored at ambient temperature for 16 h. The resulting mixtures were

concentrated under reduced pressure using the blowdown apparatus in preparation for the final step (step 3).

Series 4. Plate 3; Insufficient HCl solution, added 100 µL manually to row 6.

Series 3. Plate 2; Protocol paused half-way to refill the acid containing reservoir.

Series 1_bis. Protocol 3.5.2_06_1_P300m_HCl-dioxane_reagent4_troughTo96_perplate_plate_1.py (*_2, *_3) were successful

Protocol 8.1 – Addition of DMA to the Reaction Plates for the Amide Coupling

DMA is added to the residual mixtures contained in each of the three reaction plates, using the 300ul 8-channel pipette. This is the first step for the final coupling reaction to the reaction plates.

Series 4. Protocol successful

Series 2. Protocol was successful, had to be paused several times to refill the DMA reservoir. For future protocols, a large 1-well trough was created.

Series 3. Protocol was successful, utilising a larger trough to remove the need to pause the protocol to refill the DMA.

Series 1_bis. Protocol was successful

Protocol 8.2 – Addition of Acid stock solution to the Reaction Plates for Amide Coupling

Per series, 8 crude amines diluted in DMA are sitting across the three reaction plates, 4 rows per amine. Using the single channel 1000uL pipette 32 acids are dispensed eight times, forming a matric of 296 unique mixtures per series.

Series 4. Run 1. Copy paste error in the code for the destination wells, however only one acid was dispensed incorrectly into a blank location. Protocol was stopped after 4 minutes and the code corrected. Run 2. Four of the acids were found to be suspensions/insoluble (acids 6, 10, 18, and 32). Acids 6 and 10 the solid blocked the pipette and therefore no solution was dispensed (added manually). Sonication and manual agitation of acids 18 and 32 was able to create a slurry that the liquid handling apparatus was able to dispense. The issues with insolubility for these four reactions may have impacted the amount of the acid dispensed into these four reactions.

Series 1_bis, 2, 3. Protocols were successful. The four acids that were previously found to be suspensions/insoluble (acids 6, 10, 18, and 32) were omitted from the protocol and added manually.

Protocol 8.3 – Addition of T3P Solution and DIPEA for the Amide Coupling

Addition of 22.1 uL T3P (50% in EtOAc) and 24.3 uL DIPEA to each of the three reaction plates using the 8-channel 300 µL. After the additions, the 296 reaction plates were sealed and stored at ambient temperature for 72 h. The resulting mixtures were concentrated under reduced pressure in preparation for the subsequent work-up stage.

Series 4. Protocol successful

Series 1_bis, 2 and 3. Some issues due to the viscous nature of the T3P solution. Some dripping was observed which meant that no T3P was added on the last row. This was added manually after the automated addition.

Protocol 9.1 – Work Up – Addition of DCM and Aqueous $NaHCO_3$

Addition of DCM (300uL) and saturated aqueous $NaHCO_3$ (300 uL) solutions to the 296 crude residues for each series. using the 8-channel 300 µL.

Series 2. One pipette channel not transferring the required amount therefore the protocol was terminated after the addition of the aqueous phase. DCM was added in a separate protocol once pipette was serviced (grease added). The protocol was amended to transfer only the DCM.

Series 3. Maintenance of multichannel pipette required. Occasional DCM dripping lowering the actual amount of DCM in the wells.

Series 1_bis. Maintenance of multichannel pipette required. Occasional DCM dripping lowering the actual amount of DCM in the wells.

Series 4. Protocol ok.

Protocol 9.2 – Work Up – mixing heterogeneous mixtures.

DCM and $NaHCO_3$ phases were mixed by successive aspiration/dispense using the 300uL 8-channel

Series 2. Plate 1,2,3 - Protocol ok

Series 3. Plate 1,2,3 - Protocol ok. Faster mix than series 2 as no solids or exothermic reactions had been observed.

Series 1_bis. Plate 1,2,3 - Protocol ok

Series 4. Protocol ok.

Protocol 9.3 – Work Up – Removal of the DCM Phase

For each of the 3 plates of the series, the DCM phase (150, 180, 200, 220 uL) is taken out of the reaction plate and dispense in a destination 2ml 96-well storage plate.

Series 2. -plate 1 - Lower volume of DCM (150uL instead of 260) taken than previous work ups to ensure no aqueous contamination of the samples. Protocol ok.

-plate 2. Increased volume from plate 1 (220uL), one or two isolated instances when aqueous may have been taken up, will decrease the volume in the following work ups.

-plate 3. Increased volume from plate 1 (180 uL), protocol successful.

Series 3. -plate 1,2,3 – DCM volume set to 200uL. Protocol ok.

Series 1_bis. -plate 1,2,3 – DCM volume set to 210uL. Protocol ok.

Series 4. -plate 1 – protocol ok

-plate 2 – Run 1. recalibration needed. Run 2. Protocol used was taking out 250ul instead of 210. Row D of source plate has very little DCM left and some aqueous solution was transferred too. It might be that partition between phases was not full.

-plate 3 – protocol ok

Protocol 9.4 – Work Up – Addition of a further 300 µL of DCM for Second Extraction

Addition of a further 300 µL of DCM to the reaction plates containing the aqueous phase after the first extraction.

Series 2. -plate 1,2,3 – Protocol successful.

Series 3. -plate 1,2,3 – Protocol successful.

Series 1_bis. -plate 1,2,3 – Protocol successful.

Series 4. Run1. One tip fell and cancelled the run. Run2. Protocol change added a loop error and added another 300uL DCM in first row of the plate 1. Protocol stopped. Run 3. Successful, however pipette needs maintenance.

Protocol 9.5 – Work Up – Second Mixing

Mixing of the aqueous and organic phases in the work-up of step 3 for the second extraction, with the 8-channel 300 µL, through series of aspiration and dispensing.

Series 2. -plate 1,2,3 – Error in the code, therefore, conducted manually and will be rectified in the subsequent series

Series 3. -plate 1,2,3 – Protocol successful

Series 1_bis. -plate 1,2,3 – Protocol successful

Series 4. -plate 1,2,3 – Protocol successful

Protocol 9.6 –Work Up – Removal of the Bottom DCM Phase after Second Extraction

Removal of the bottom DCM phase (250 µL) and transfer to a separate plate with the 8-channel 300 µL pipette. After this stage, the combined DCM extracts were concentrated using the blowdown apparatus.

Series 2. -plate 1 – Increased volume relative to the first extraction to maximise the product recovery, no aqueous contamination was observed.

-plate 2 – Protocol ok.

-plate 3 – Protocol run was for 12 rows instead of 8. Stopped before the end of the protocol.

Series 3. -plate 1 – Protocol ok.

-plate 2 – 270 uL was taken out but there were a few instances of a drop of aqueous phase contamination.

-plate 3 – Protocol successful

Series 1_bis. -plate 1 – Protocol ok.

-plate 2 – Protocol successful

-plate 3 – One tip fell during the run but the robot could be paused in time.

Series 4. -plate 1 – Some aqueous solution taken out.

-plate 2 – Protocol successful

-plate 3 – Volume of DCM down to 210uL but some aqueous was still taken. Unfortunately there was a long period of time (3 h) between the addition of DCM, mix of the phases and the extraction, whicch reduced the volume of DCM in the wells.

Protocol 10 – Addition of MeCN/MeOH to Enable LCMS and Transfer to the Screening Plate

Addition of MeCN (155 µL) to the storage plates containing the residual crude mixtures after work-up and concentration, using the 8-channel 300 µL pipette.

Series 2. Run1.Protocol was successful, however, despite sonication some insolubility was observed. Subsequent series will be tested with methanol instead of MeCN. MeCN was removed. Run 2. This time with MeOH. Protocol successful

Series 1_bis. MeOH superior to MeCN as almost complete solubility was observed. This will be utilised in the remaining series.

Series 3. Protocol successful.

Series 4. Run1. Run stopped because of an unusual noise on arm thread of OT1. Run 2 started after robot maintenance. Run 2 OK.

Protocol 11 – Short Mixing Protocol after the MeOH Addition

Short mixing protocol using the 8-channel 300 µL pipette.

Series 1_bis. Plate 1,2,3 - All in solution after the mixing stage.

Series 2. Protocol successful

Series 3. Protocol successful.

Series 4. Wrong protocol used for plate 2. 15uL of the first row discarded. Protocol successful in run2 and for plate 1 and 3

Protocol 12 – Preparation of the LCMS Plates by Addition of Water (20 µL) and MeCN (40 µL)

Addition of water (20 µL) and MeCN (40 µL) to three 96-well low volume QC plates for the final LCMS analysis using the 8-channel 300 µL pipette.

Series 1_bis. Protocol successful

Series 2. Protocol successful

Series 3. Protocol successful.

Series 4. Protocol successful.

Protocol 13 – Transfer of the Final Product Reaction Mixture in MeOH (15 µL) to the LCMS/QC Plate

Transfer of an aliquot of the final product mixtures dissolved in methanol (15 µL) to the pre-prepared LCMS plate (See above – Protocol 12). After this addition, the QC plate was analysed by LCMS to provide an indication of the final purity estimation of the samples. The remaining methanol from the final product mixture was removed using the blowdown apparatus to enable the addition of the screening solvent (See below).

Series 1_bis. Protocol successful

Series 2. Protocol successful

Series 3. Protocol successful

Series 4. Protocol successful

Protocol 14 – Addition of Ethylene Glycol to 96-well storage plates.

Addition of ethylene glycol (140 µL) to the screening plates with the using the 8-channel 300 µL pipette, after concentration on the blowdown apparatus to remove the methanol.

The resulting samples were stored at 0 °C until transfer to echo plates.

Series 1_bis. Protocol ok, some dripping of the solvent was observed, potentially due to the surface tension of ethylene glycol. Plunger speed was decreased from 700 to 200 for the subsequent series.

Series 2. Protocol successful, decreased plunger speed prevented dripping during the transfer.

Series 3. Protocol successful.

Series 4. Protocol successful.

Protocol 15 – Transfer to the 384-Well Echo Screening Plate

Transfer of an aliquot of the ethylene glycol solution (30 µL) to the 384-well echo plate for screening.

Series 1_bis. Coding error for the protocol used for the transfer of plate 3. Column 12 in screen plate has a mixture of 2 compounds in each wells. Protocol was stopped, and started again once the code corrected.

Series 2. Protocol successful.

Series 3. Protocol successful.

Series 4. Protocol successful.

7.4 Iteration 4.2 Five reactions – sulphonamide syntheses

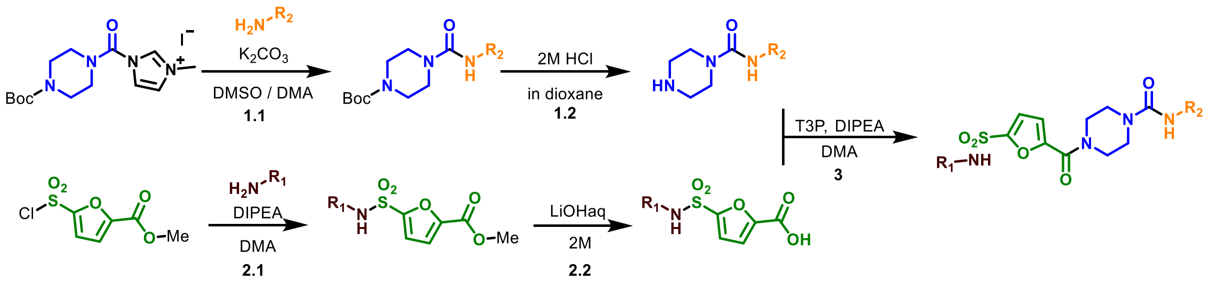

Scheme S4 – Parallel synthesis used for iteration 4.2

Experimental details

1.1 and 1.2 Urea syntheses and Boc deprotection

Eight amines (158 uL, 0.079 mmol, 1.05 eq, 500 mM in DMA) were dispensed in one 2ml 96-well reaction plates, each amine transferred across the 8 wells of a column already containing K_2CO_3 (15.5 mg, 0.113 mmol, 1.5 eq). Next the imidazolium salt (150 uL, 0.075 mmol, 1.00 eq, 250 mM in DMSO) was dispensed, one per plate and across the 64 amine containing wells. The reaction plate was sealed and stored overnight at rt. The reaction mixtures were concentrated (SPE-dry) and partitioned in DCM (300uL) and an aqueous solution (1M aqueous HCl or 50% brine solution[a], 600 uL). The organic extracts were transferred (260uL) to a 2mL 96-well plate and each aqueous phase was extracted a second time with DCM (300uL). The organic extracts (260uL) were added to the 96-well plate containing the first extracts and the DCM was removed overnight. 1,4-Dioxane (1200 uL) was added to each crude residue and upon complete dilution, each crude mixture was dispensed (395 uL, 0.025 mmol) in 3 wells, across 2 reaction plates. 4M HCl in 1,4-dioxane (200 µL, excess) was added to each well of the two reaction

plates. The reaction plates were sealed and stored at ambient temperature for 16 h. The resulting mixtures were concentrated under reduced pressure using the blowdown apparatus, then DMA was added (250 uL)

2.1 and 2.2 Sulphonamide syntheses and Ester hydrolysis

Methyl 5-(chlorosulfonyl)furan-2-carboxylate (600 uL, 0.3 mmol, 1, eq, 500 mM in DMA) was added in the first 20 wells of a 2mL 96-well reaction plate. To each well was dispensed one amine (600uL, 0.3 mmol, 1 eq, 500 mM in DMA, *cf 4-2_amines_sulfonamides.csv*) followed by DIPEA (125 uL, 0.718 mmol, 2.4 eq). The reaction plate was sealed and stored overnight at rt. The reaction mixtures were concentrated (SPE-dry) and partitioned in DCM (300uL) and an aqueous solution (1M aqueous HCl or 50% brine solution[a], 300uL). The organic extracts were transferred (260uL) to a 2mL 96-well plate (Starlab, 300uL) and each aqueous phase was extracted a second time with DCM (300uL). The organic extracts (260uL) were added to the 96-well plate containing the first extracts and the DCM was removed overnight. To the crude sulfonamide esters was added an aqueous solution of LiOH (2M, 500 uL, excess) and 1,4-dioxane (500 uL). The resulting mixtures were covered, sonicated to mix/dissolve the starting material, and stored at ambient temperature overnight. The reaction mixtures were then concentrated (SPE-Dry) and partitioned with DCM (600uL) and an aqueous solution (2M aqueous HCl or 50% brine solution[a], 600uL). After a mixing step, more acidic aqueous solution was added (3M aqueous HCl, 200 uL or 250 uL)[b] and both phases mixed. The organic extracts were transferred (560uL) to a 2mL 96-well plate and each aqueous phase was extracted a second time with DCM (600uL). The organic extracts (560uL) were added to the 96-well plate containing the first extracts and the DCM was removed overnight. After LCMS analysis of the crudes, DMA (600 uL, 500 mM) was added to the crudes residues and the solutions mixed to ensure full dilution.

3. Coupling between deprotected ureas and acid sulfonamides

To each deprotected urea (0.025 mmol, 1 eq) contained in two 96-well plates and diluted with DMA was added a solution of an acid sulfonamide (52.5 uL per reaction well, 0.026 mmol, 1.04 eq) using the single channel 1ml pipette. As an example, the sulfonamide acid solution contained in the first well (A1) of the source sulfonamide plate was transferred to A1, A4, A7, A10 in the first urea containing destination plate and A1, A4, A7, A10 of the second urea containing destination plate. 180 reaction mixtures were prepared, while ureas containing wells in col 3, 6, 9, 12 row E, F, G, H of each destination plate were discarded.

T3P (18 ul, 1.2 eq, 50% in EtOAc), then Et_3N (16 uL, 4.5 eq) were added. The 2 reaction plates containing 180 amide coupling reactions were sealed and stored at ambient temperature for 72 hours. The reaction mixtures were concentrated partitioned in DCM (300uL) and an aqueous saturated solution of $NaHCO_3$ (300uL). The organic extracts were transferred (210uL) to a 96-well plate (Starlab, 300uL) and each aqueouse phase was extracted a second time with DCM (300uL). The organic extracts (260uL) were added to the 96-well plate containing the first extracts and the DCM was removed overnight. MeOH (125 µL) was added and an aliquot (15 uL) of each solution was transferred to 96-well low volume plates for LCMS analysis. MeOH was then removed (SPE-Dry) and EG (120 ul, 183 mM) added. An aliquot (30 uL) of the 180 reactions was then transferred to 384PP screening plates.

[a]Brine was added instead of HCl for ureas contining basic sites

[b]250 for acids containing a basic site, 200 for the ones that did not.

Robotic steps protocols

Urea (1.1 and 1.2)

01.2_P1000s_stockSolutions_trough_or_vialRack_amines_urea.py
01.3_P1000s_stockSolutions_trough_or_vialRack_imidazolium.py
02_P300m_reactant1_troughTo96rack.py
03_P300m_reactant2addition_troughTo96rack.py
07_1_Ureas_workup_96_addition_orga_and_aqueous_base_or_neutral.py
07_2_Ureas_workup_96_mix_exothermic.py
07_4_Ureas_workup_96_mix_short.py
07_5_Ureas_pWUpBottomPhaseTransfer_96to96_multi300.py
07_6_Ureas_workup_96_extration2_addDCM.py
07_7_Ureas_workup_96_mix_even_shorter.py
07_8_Ureas_pWUpBottomPhaseTransfer_96to96_multi300.py
10_1_P300m_urea_dioxane_addition_and_mix_troughTo96.py
10_2_P300m_split_96_to_2_96_three_row_identical.py
10_3_P300m_HCl-dioxane_reagent_troughTo96_perplate_plate_1.py
10_4_P300m_HCl-dioxane_reagent_troughTo96_perplate_plate_2.py

Sulfonamide (2.1 and 2.2)
01.1_P1000s_stockSolutions_trough_or_vialRack_amines_sulfonamide.py
01.4_P1000s_stockSolutions_trough_or_vialRack_sulfonylCl.py
04_Sulfonamide_P1000s_SulfonylCl_troughto96.py
05_Sulfonamide_P1000s_aminesSO2Cl_addition_storage_96.py
06_Sulfonamide_P300m_base_troughTo96.py
08_1_P1000s_Sulfo_workup_96_addition_orga_and_aqueous_base_or_neutral.py
08_2_p300_Sulfo_workup_96__addDCM.py
08_3_Sulfo_workup_96_mix.py
08_4_Sulfo_pWUpBottomPhaseTransfer_96to96_multi300.py
08_5_p300_Sulfo_workup_96__addDCM.py
08_6_Sulfo_workup_96_mix.py
08_7_Sulfo_pWUpBottomPhaseTransfer_96to96_multi300.py
09_1_Sulfonamide_P300m_dioxane_LiOH_troughTo96.py
09_2_Sulfonamide_P300m_workup_96_mix_short_OPTIONAL.py
11_1_P1000s_Sulfo_workup_96_addition_orga_and_aqueous_acid_or_neutral.py
11_2_Sulfo_workup_96_mix_exothermic.py
11_3_P1000s_Sulfo_workup_96_addition_aqueous_acid.py
11_4_Sulfo_workup_96_mix_exothermic.py
11_5_Sulfo_pWUpBottomPhaseTransfer_96to96_multi300.py
11_6_Sulfo_workup_96_extration2_addDCM.py
11_7_Sulfo_workup_96_mix_even_shorter.py
11_8_Sulfo_pWUpBottomPhaseTransfer_96to96_multi300 .py

Combined (3)
12_1_Combined_P300m_add_solvent_in_2_plates.py
12_2_Combined_P300m_add_solvent_in_2_plates_plate2_row3_12.py
13_1_Sulfo_P300m_stock_solution_DMA_troughTo96_and_mixing.py
13_2_Combined_P1000s_reactant2_addition_on_2_plate.py
14_Combined_P300m_add_reagent_1_and_2_in_3_plates.py
15.1_p300_combined_workup_all_in_one.py
15.2.1_combined_workup_96_mix_even_shorter_plate_1.py

15.2.2_combined_workup_96_mix_even_shorter_plate_2.py
15.3.1_combined_workup_BottomPhaseTransfer_96to96_plate1.py
15.3.2_combined_workup_BottomPhaseTransfer_96to96_plate2.py
15.4_combined_workup_96_extration_addDCM_all_in_one.py
15.5.1_combined_workup_96_mix_even_shorter_plate_1.py
15.5.2_combined_workup_96_mix_even_shorter_plate_2.py
15.6.1_combined_workup_BottomPhaseTransfer_96to96_plate1.py
15.6.2_combined_workup_BottomPhaseTransfer_96to96_plate2.py
16_add_MeOH_all_in_one.py
17.1_96_short_mix_plate_1.py
17.2_96_short_mix_plate_2.py
18_prepare_QC_plate_addMeCN_waterQC_all_in_one.py
19.1_transfer_15_to_QC_plate_1.py
19.2_transfer_15_to_QC_plate_2.py
20_add_EG_all_in_one.py
21.1_96_short_mix_plate_1.py
21.2_96_short_mix_plate_2.py
22_transfer_30_to_384PP_screen_plate_1.py
22_transfer_30_to_384PP_screen_plate_2.py

Protocol 01.1 – Amines Stock solution for the sulfonamide reaction (2.1)

Dilution of 20 amines in DMA (500mM) in 24-4ml-vial rack, capped after addition of the solvent, using the 1mL single channel pipette.

Run 1. Calibration error on the amine containing rack and run stopped after 4 stock solutions.

Run 2. No tip for the first 4 transfers, then paused to add the tip. Dispensing height also adjusted for as initially too high. Protocol successful.

Protocol 01.2 – Amines Stock solution for the urea reaction (1.1)

Dilution of 8 amines in DMA (500mM) in 12-row trough, capped after addition of the solvent, using the 1mL single channel pipette.

Run 1. Protocol successful. Air-gap improves liquid transfers.

Protocol 01.3 – Imidazolium Stock solution for the urea reaction (1.1)

Dilution of the imidazolium salt in DMSO (250mM) in a 4-well trough, using the 1mL single channel pipette.

Run 1. Protocol successful- solution stirred manually and left for 1h to dissolve fully.

Protocol 02 – Addition of Amines to reaction rack (1.1)

Transfer of 8 amines (158 uL, 0.079 mmol, 1.05 eq) from the 12-well trough stock solution to a 2ml-96-well reaction rack already containing K_2CO_3 (15.5 mg, 0.113 mmol, 1.5 eq) in each reaction well using the 8-channel pipette. Each amine is transferred 8 times, one amine per column to fill the 8 first columns of the reaction plate.

Run 1. Protocol successful

Protocol 03 – Addition of Imidazolium salt to reaction rack (1.1)

Transfer of imidazolium salt (150uL, 0.600 mmol, 1.00 eq) from the 4-well trough stock solution to the 2ml-96-well reaction rack already containing K_2CO_3 and amines using the 8-channel pipette. The The same amount is transferred to each of the 64 reaction wells. The reaction plate was then sealed and stored overnight at rt. The reaction solvent mixture was then removed (SPE-dry).

Run 1. Protocol successful. There is an issue with dmso drops sometimes falling on the deck after transfer but it is not detrimental to the reactions and there is no risk of contamination (cf 3.5, transfer to 4 plates on the same deck)

Protocol 01.4 – Sulfonyl chloride stock solution (2.1)

Stock solution prepared just before reaction setup due to reactivity of the sulfonyl chloride. Dilution of the sufonyl chloride in DMA (500 mM) in a 12-well trough, using the 1mL single channel pipette.

Run 1. Protocol successful.

Protocol 04 – Addition of sulfonyl chloride to reaction rack (2.1)

Transfer of sulfonyl chloride (600 uL, 0.3 mmol, 1 eq, 500 mM in DMA) from the 12-well trough stock solution to the first 20 wells of 2ml-96-well reaction rack using the 1mL single channel pipette.

Run 1. Protocol successful although transferred to 21 wells insead of 20. Code was amended.

Protocol 05 - Addition of the amines to the sulfonylchloride containing wells

Transfer of the 20 amines (600 uL, 0.3 mmol, 1 eq, 500 mM in DMA) from the 24-rack stock solution to the first 20 wells of 2ml-96-well reaction rack containing the sulfonyl chloride using the 1mL single channel pipette.

Protocol 06 - Addition of the base to the sulfonyl chloride reactions

Transfer of DIPEA (125 uL, 2.4 eq) using the 1mL single channel pipette. The reaction rack was sealed and stored at rt overnight. The reaction solvent was then removed (SPE-dry)

Run 1. File amended in the csv before starting to allow for the fact its a 0.30 mmol reaction (not 0.60 mmol). But the robot still transfered 2.4 eq of DIPEA (125ul), most likely because the csv file was not saved.

Protocol 07.1 – Urea Workup – Addition of aqueous solution and DCM to the urea formation reactions (1.1)

Addition of DCM (300uL) and either 1M aqueous HCl or 50% brine solution (300uL) depending on the expected ureas (e.g. picolyl-derived urea with a basic site was worked up using brine) using the 8-channel 300 µL pipette.

Run 1. Protocol successful, potassium carbonate still observed at the bottom of the wells, therefore will use the slower mix protocol in the following stage.

Protocol 07.2 – Urea Workup – Mixing the organic and aqueous phases (1.1)

Mixing the organic and aqueous phases through a series of dispensing and aspiration (300 uL each) using the 8-channel 300 µL pipette.

Run 1. Exothermic mix protocol successful. pH checked not obvious therefore addition of another volume of HCl, and the quick mix script.

Protocol 07.3 – Urea Workup – Addition of more Aqueous solution to the reaction wells (1.1)

Addition of either 1M aqueous HCl or 50% brine solution (300uL) depending on the expected ureas (e.g. picolyl-derived urea with a basic site was worked up using brine) using the 8-channel 300 µL pipette.

Run 1. Protocol successful

Protocol 07.4 – Urea Workup – Mixing the organic and aqueous phases (1.1)

Mixing the organic and aqueous phases through a series of dispensing and aspiration (300 uL each) using the 8-channel 300 µL pipette.

Run 1. Protocol successful

Protocol 07.5 – Urea Workup – Removal of the bottom DCM phase (1.1)

Removal of the bottom DCM phase (260 µL) and transfer to a 2mL 96-well reaction plate for Step 1.2 (Boc deprotection) using the 8-channel 300 µL pipette.

Run 1. Protocol successful

Protocol 07.6 – Urea Workup – Addition of a further 300 µL of DCM to the remaining aqueous mixture (1.1)

Addition of a further 300 µL of DCM to the reaction plate from step 1.1 containing the aqueous phase after the first extraction.

Run 1. Protocol successful

Protocol 07.7 – Urea Workup – Mixing protocol for the second extraction (1.1)

Repeat of the mixing the organic and aqueous phases through a series of dispensing and aspiration with the 8-channel 300 µL pipette.

Run 1. Protocol successful

Protocol 07.8 – Urea Workup – Removal of the bottom DCM phase for the second extraction (1.1)

Removal of the bottom DCM phase (260 uL) and transfer to the reaction plate for Step 1.2 (Boc deprotection) with the 8-channel 300 µL pipette. The combined extracts were then concentrated (SPE-dry)

Run 1. Protocol successful

Protocol 08.1 – Sulfonamide Workup – Addition of aqueous solution and DCM to the sulfonamide formation reactions (2.1)

Addition of DCM (300uL) and either 1M aqueous HCl or 50% brine solution (300uL) depending on the expected ureas (e.g. picolyl-derived urea with a basic site was worked up using brine) using the single channel 1mL pipette on the crude residues.

Run 1. Aqueous addition ok, not DCM, stopped after 3 additions. The volume aspirated is too low for the single channel.

Protocol 08.2 – Sulfonamide Workup – Addition of DCM to the sulfonamide formation reactions (2.1)

Addition of DCM (300uL) to the sulfonamide reactions using the 8-channel 300 uL on the 96-well reaction rack.

Run 1. Protocol successful. 8-channel pipette handles small volumes of DCM better than the single channel 1 mL pipette.

Protocol 08.3 – Sulfonamide Workup – Mixing the organic and aqueous phases (2.1)

Mixing the organic and aqueous phases through a series of dispensing and aspiration (300 uL each) using the 8-channel 300 µL pipette.

Run 1. Protocol successful

Protocol 08.4 – Sulfonamide Workup – Transfer of the bottom DCM phase (2.1)

Removal of the bottom DCM phase (260 µL) and transfer to a 2mL 96-well reaction plate for Step 2.2 (hydrolysis) using the 8-channel 300 µL pipette.

Run 1. Protocol successful

Protocol 08.5 – Sulfonamide Workup – Addition of a further 300 µL of DCM to the remaining aqueous mixture (2.1)

Addition of a further 300 µL of DCM to the reaction plate from step 2.1 containing the aqueous phase after the first extraction.

Run 1. Protocol successful

Protocol 08.6 – Sulfonamide Workup – Mixing protocol for the second extraction (2.1)

Repeat of the mixing the organic and aqueous phases through a series of dispensing and aspiration with the 8-channel 300 µL pipette.

Run 1. Protocol successful

Protocol 08.7 – Sulfonamide Workup – Removal of the bottom DCM phase for the second extraction (2.1)

Removal of the bottom DCM phase (260 uL) and transfer to the reaction plate for Step 2.2 (hydrolysis) with the 8-channel 300 µL pipette. The combined extracts were then concentrated (SPE-dry)

Run 1. Protocol successful

Protocol 09.1 - addition of 1,4-dioxane and 2M LiOH to the sulfonamide esters (2.2)

To the crude residues from the sulfonamides formation reactions in a 2ml 96-well reaction plate was added 2M LiOH (500 uL) and 1,4-dioxane (500uL). The resulting mixtures were covered, sonicated to mix/dissolve the starting material, and stored at ambient temperature overnight before LCMS analysis.

Run 1. Protocol successful. However, sonication did not dissolve the substrates. Therefore, a mixing protocol was required. NOTE: add dioxane and dissolve the residues before LiOH addition.

Protocol 09.1 – mixing of the sulfonamide esters containing solution (2.2)

The crude residues from the sulfonamides formation reactions in a 2ml 96-well reaction plate was not fully solubilised in the Dioxane/LiOH solution, therefore a mixing stage was added to the protocol, using the 8-channel pipette

Runs. The protocol was run 3 times, with minor changes in mixing height, leading to almost full solubilisation of the 20 sulfonamide esters. The reaction plate was sealed and stored overnight at room temperature. The reaction mixtures were then concentrated (SPE-dry).

Protocol 10.1 - addition of dioxane to the crude ureas post work-up and mixing of the solutions (1.2)

To the crude residues from the urea formation reactions was added 1,4-dioxane (1200uL) using a p300 8-channel pipette. A mixing step ensured the residues were fully solubilised.

Run 1. Protocol successful

Protocol 10.2 - transfer of the Urea dioxane solutions to the reaction plates (Boc-deprotection) (1.2)

Each column containing eight solutions (1200 uL) of one crude urea were split in 3 columns of two 2ml 96-well reaction rack, using the 8-channel pipette. Ureas (395 uL) one to four (in columns 1 to 4) were transferred to the first plate, while Ureas five to eight (in columns 5 to 8) were transferred to the second plate.

Run 1. Protocol successful

Protocol 10.3 – addition of 4M HCl/dioxane on the ureas to remove the Boc group (1.2)

To the crude ureas in 1,4-dioxane was added 4M HCl-dioxane (200uL, excess). The two reaction plates were sealed and stored overnight. The reaction mixtures were then concentrated (SPE-dry)

Run 1. Protocol successful for both plates.

Protocol 11.1 – Sulfonamide Workup – addition of 2M HCl or Brine and DCM to the sulfonamide hydrolysis reaction mixtures after concentration. (2.2)

To the 20 crude sulfonamide acid residues was added DCM (600uL) and either 2M aqueous HCl or 50% brine solution (600uL) depending on the expected sulfonamide. (e.g. picolyl-derived sulfonamide with a basic site was worked up using brine) using the single channel 1mL pipette.

Run 1. Protocol successful – as a warning DCM needs to be transferred quickly because it consumes the air_gap

Protocol 11.2 – Sulfonamide Workup – Mixing the organic and aqueous phases (2.2)

Mixing the organic and aqueous phases through a series of dispensing and aspiration (300 uL each) using the 8-channel 300 µL pipette.

Run 1. Slow mixing at first to avoid any excess bubbling. Protocol successful. However, indicator paper suggested that the PH was >6, therefore more acid solution needed.

Protocol 11.3 – Sulfonamide Workup – Addition of 3M HCl to the reactions (2.2)

To the reaction solutions was added 3M aqueous HCl (200 uL if no basic site, 250 if basic site) to make sure the solution are neutralised/acidic.

Run 1. Protocol successful - indicator paper suggested that the pH was now 1 or below (still above 6 for the basic sites)

Protocol 11.4 – Sulfonamide Workup – Mixing the organic and aqueous phases (2.2)

Mixing the organic and aqueous phases through a series of dispensing and aspiration (300 uL each) using the 8-channel 300 µL pipette.

Run 1. Protocol successful

Protocol 11.5 – Sulfonamide Workup – Transfer of the bottom DCM phase (2.2)

Removal of the bottom DCM phase (560 µL) and transfer to a 2mL 96-well reaction plate for the couplinng step using the 8-channel 300 µL pipette.

Run 1. Protocol successful, minor contamination with aquous phase

Protocol 11.6 – Sulfonamide Workup – Addition of a further 600 µL of DCM to the remaining aqueous mixture (2.2)

Addition of a further 600 µL of DCM to the reaction plate from step 2.2 containing the aqueous phase after the first extraction, with the 8-channel pipette.

Run 1. Protocol successful

Protocol 11.7 – Sulfonamide Workup – Mixing protocol for the second extraction (2.2)

Repeat of the mixing the organic and aqueous phases through a series of dispensing and aspiration with the 8-channel 300 µL pipette.

Run 1. Short mix protocol successful

Protocol 11.8 – Sulfonamide Workup – Removal of the bottom DCM phase for the second extraction (2.2)

Removal of the bottom DCM phase (560 uL) and transfer to the reaction plate for the final coupling step with the 8-channel 300 µL pipette. The combined extracts were then concentrated (SPE-dry)

Run 1. Protocol successful

- LCMS/QC of the hydrolysis after the work-up showed that the hydrolysis reaction had been successful (first 2 acids, full QC of the products and the aqueous phase) still ongoing.

Protocol 12.1 – Add DMA to crude deprotected ureas containing wells (3)

To the wells containing the boc deprotected ureas residues was added DMA (250 mL) using the 8-channel pipette. The solutions were then mixed by successive aspiration/dispense actions (300 uL).

Run 1. Protocol changed, the second tip rack was moved to C2 (from E3) as the p1000 hit the tip rack in D3. Mixing and addition was fine, but the csv was wrong - had 2 rows not 12 in the second. Csv file was amended. Not completely dissolved in a lot of cases but this is anticipated to improve as the rest of the reagents are added.

Protocol 12.2 – Add DMA to crude deprotected ureas containing wells (3)

Due to a typo in the csv file (reaction conditions) DMA was only added to columns 1-2 in the second plate of ureas. Another protocol was run to add the DMA in the rest of the ureas containing wells.

Run 1. Protocol successful

Protocol 13.1 - addition of DMA to the sulfonamide acids to prepare stock solutions that can be added to the crude ureas (3)

To the 20 wells containing the sulfonamide acid residues was added DMA (600 mL) using the 8-channel pipette. The solutions were then mixed by successive aspiration/dispense actions (300 uL).

Run 1. Protocol successful. At the end of the protocol the plate was sonicated until dissolution of all acids.

Protocol 13.2 – Add sulfonamide acids solutions to crude ureas containing reaction plates. (2.2)

To the crude urea dissolved in DMA (250 uL) was added the sulfonamide acids solutions (52.5 uL per reaction well) using the single channel 1ml pipette. Each sulfonamide was transferred to one well containing one of the 8 ureas. As an example, the sulfonamide acid solution contained in the first well (A1) of the source sulfonamide plate was transferred to A1, A4, A7, A10 in the first urea containing destination plate and A1, A4, A7, A10 of the second urea containing destination plate. 180 reaction mixtures were prepared, while ureas containing wells in col 3, 6, 9, 12 row E, F, G, H of each destination plate were discarded.

Run 1. Protocol successful apart from urea 8 where the amount dispensed was too little (slight loss over the 8 consecutive transfers). This was corrected by manual addition.

Protocol 14– Addition of T3P Solution and DIPEA for the final Amide Coupling

Addition of 18 uL T3P (50% in EtOAc) and 16 uL Et_3N to each of the three reaction plates using the 8-channel 300 µL. After the additions, the reaction plates were sealed and stored at ambient temperature for 48 h. The resulting mixtures were concentrated under reduced pressure in preparation for the subsequent work-up stage.

Run 1. Protocol successful apart from known T3P issue (dripping meant that row 12 in both cases didn't get the correct amount of the T3P). It was added manually.

Protocol 15.1 – Coupling Workup – Addition of aqueous solution and DCM to the urea formation reactions (3)

Addition of DCM (300uL) and saturated aqueous $NaHCO_3$ solution (300uL) using the 8-channel 300 µL pipette.

Run 1. Protocol successful.

Protocol 15.2 – Coupling Workup – Mixing the organic and aqueous phases (3)

Mixing the organic and aqueous phases through a series of dispensing and aspiration (300 uL each) using the 8-channel 300 µL pipette.

Run 1. Quick mix protocol successful for both reaction plates

Protocol 15.3 – Coupling Workup – Removal of the bottom DCM phase (3)

Removal of the bottom DCM phase (210 µL) and transfer to a 2mL 96-well storage plate using the 8-channel 300 µL pipette.

Run 1. Protocol successful for both reaction plates

Protocol 15.4 – Coupling Workup – Addition of a further 300 µL of DCM to the remaining aqueous mixture (3)

Addition of a further 300 µL of DCM to the reaction plates containing the aqueous phase after the first extraction.

Run 1. Protocol successful

Protocol 15.5 – Coupling Workup – Mixing protocol for the second extraction (3)

Repeat of the mixing the organic and aqueous phases through a series of dispensing and aspiration with the 8-channel 300 µL pipette.

Run 1. Protocol successful for both reaction plates

Protocol 15.6 – Coupling Workup – Removal of the bottom DCM phase for the second extraction (3)

Removal of the bottom DCM phase (260 uL) and transfer to the reaction plate for Step 1.2 (Boc deprotection) with the 8-channel 300 µL pipette. The combined extracts were then concentrated (SPE-dry)

Run 1. Protocol successful for the first plate. For the second plate a tip fell four times, signs that maintenance (calibration) is due.

Protocol 16 – Addition of MeOH to Enable LCMS and Transfer to the Screening Plate

Addition of MeOH (125 µL) to the storage plates containing the residual crude mixtures after work-up and concentration, using the 8-channel 300 µL pipette.

Run 1. Protocol successful

Protocol 17 – Short Mixing Protocol after the MeOH Addition

Short mixing protocol using the 8-channel 300 µL pipette.

Run 1. Protocol successful for both plates

Protocol 18 – Preparation of the LCMS Plates by Addition of Water (20 µL) and MeCN (40 µL)

Addition of water (20 µL) and MeCN (40 µL) to three 96-well low volume QC plates for the final LCMS analysis using the 8-channel 300 µL pipette.

Run 1. Protocol successful

Protocol 19 – Transfer of the Final Product Reaction Mixture in MeOH (15 µL) to the LCMS/QC Plate

Transfer of an aliquot of the final product mixtures dissolved in methanol (15 µL) to the pre-prepared LCMS plate (See above – Protocol 18). After this addition, the QC plate was analysed by LCMS to provide an indication of the final purity estimation of the samples. The remaining methanol from the final product mixture was removed using the blowdown apparatus to enable the addition of the screening solvent (See below).

Run 1. Protocol successful

Protocol 20 – Addition of Ethylene Glycol to 96-well storage plates.

Addition of ethylene glycol (120 µL) to the screening plates with the using the 8-channel 300 µL pipette, after concentration on the blowdown apparatus to remove the methanol.

The resulting samples were stored at 0 °C until transfer to echo plates.

Run 1. Protocol successful

Protocol 21 – Short Mixing Protocol after EG Addition

Short mixing protocol using the 8-channel 300 µL pipette.

Run 1. Protocol successful for both plates. Best practice seems to be 1 min ultrasound, then mix, then ultrasound if necessary.

Protocol 22 – Transfer to the 384-Well Echo Screening Plate

Transfer of an aliquot of the ethylene glycol solution (30 µL) to the 384-well echo plate for screening.

Run 1. Stopped early to lower the dispensing height.

Run 2. Protocol successful

7.5 Iteration 3.0

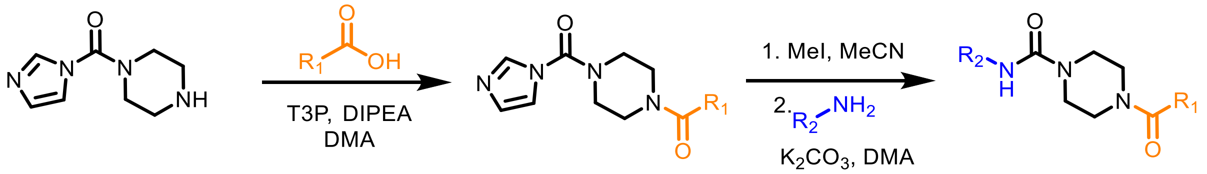

Scheme S5 – Amidation and urea reactions for iteration 3.0

Experimental details

Iteration 3.0 – To a 2 ml 96-well plate containing in the first 64 wells DIPEA (52.3 ul, 0.300 mmol, 2.5 eq) and T3P (85.7 ul, 0.144 mmol, 1.2 eq 50% in EtOAc) were added in each well a different carboxylic acid (252 ul, 0.126 mmol, 1.07 eq, 500 mM in DMA, *cf acids_list.csv*). The 64 coupling reactions were started with the addition of (1H-imidazol-1-yl)(piperazin-1-yl)methanone (121 ul, 0.118 mmol, 1.0 eq, 978 mM in DMA/DIPEA, *cf main_reactant.csv*). The reaction plate was sealed and stored for 24h at rt. The reaction mixtures were then concentrated (SPE-dry) and partitioned in DCM (300uL) and 1M aqueous solution (300uL). The organic extracts were transferred (260uL) to a 96-well plate (Starlab, 300uL) and each aqueous phase was extracted a second time with DCM (300uL). The organic extracts (260uL) were added to the 96-well plate containing the first extracts and the DCM was removed overnight. DMA (500uL) was then added followed by iodomethane (75 ul, 1.21 mmol, 10 eq). The reaction plate was sealed, and stored overnight at rt. The reactions were concentrated and the residues diluted in DMA (800 ul) with a mixing stage to ensure full dilution of the imidazolium salts. An aliquot (100ul, 0.015 mmol, 1.05 eq) of each row of the source plate containing the imidazolium

salt solution was transferred seven times to seven 2ml 96-well reaction plates leaving one aliquot in the original plate. Next K_2CO_3 (2.9 mg, 0.021 mmol, 1.5 eq) was added manually, then eight amines (43 ul, 0.014 mmol, 1.0 eq, 333 mM in DMA, *cf amines_for_urea_reaction.csv*) were transferred to each of the 512 wells containing the imidazolium salt (0.015 mmol, 1.05 eq) in DMA (100 ul), one amine per reaction plate. The reaction plates were sealed and stored for 24 h. DMA (100ul) was added to each reaction well and aliquot (15 ul) was transferred to low-profile 96-well plates for LCMS analysis. The reaction mixtures were concentrated (SPE-dry) partitioned in DCM (300uL) and an aqueous saturated solution of $NaHCO_3$ (300uL). The organic extracts were transferred (250 uL) to a 96-well plate (Starlab, 300uL) and concentrated overnight. Ethylene glycol (80 µL, 164 mM) was added and an aliquot (15 uL) of each solution was transferred to 96-well low volume plates for post work-up LCMS analysis. A second aliquot (30 uL) of the 512 reactions was then transferred to 384PP screening plates.

Protocols

01_SC1000_mainReactant_stocksolution_trough.py
02_SC1000_mainReactant_stocksolution_troughto24.py
03_MC300_base_reagent_troughTo96.py
04_SC1000_reactants_addition_storage_96.py
05_SC1000_mainReactant_transfer_96.py
06_dmf_iodo_addition.py
07_MC300_dma_addition.py
08_p300_mixing-protocol.py
09_1_MC300_dispense_96_3times96_ToRUNTWICE.py
09_2_MC300_dispense_96_3times96_ToRUNONCE.py
10_1_stock_solution_amine_SC1000_reactants_stocksolution_trough_vialstorage.py
10_2_SC1000_1amine_dispense_24To1times96_col.py
11_1_Ureas_MC300_add_DMA_troughTo96_RUNONCE.py
11_2_Ureas_MC300_add_DMA_troughTo96_RUNTWICE.py
12_QC_96to96_multichannel.py
13_1_Ureas_workup_96.py
13_2_pWUpBottomPhaseTransfer_96to96_multi300.py
14_ADDEG_trough_96.py
15_pWUpQC_Screen_96to96_96to384_troughTo96_multi300.py

Protocol 01 – Preparation of Imidazole stock solution
To (1H-imidazol-1-yl)(piperazin-1-yl)methanone (2.55 g, 10.1 mmol) in a 4-well trough was added DMA (6716 uL) and DIPEA (3509 uL, 20 mmol, 2.0 eq) to make a 978 mM stock solution using the 1mL single channel pipette.
Protocol was successful with the imidazole fully dissolved.

Protocol 02 – Transfer of Imidazole stock solution to storage vials
(1H-imidazol-1-yl)(piperazin-1-yl)methanone in DMA (978 mM) was transferred to 4 ml 24-vial FluidX racks using the 1000 ul single channel pipette. The maximum volume transferred per vial was set to 2000 ul.
Protocol was successful. However calibration of the 24-rack needed, and for small storage volume of solvent (10mL), it would have been better to use a 12-well trough.

Protocol 03 – Addition of T3P and DIPEA to a 2ml 96-well reaction plate

DIPEA (52.3 ul, 0.300 mmol, 2.5 eq) and T3P (85.7 ul, 0.144 mmol, 1.2 eq 50% in EtOAc) were added to the first 9 columns of a 2ml 96-well reaction plate using the 300ul 8-channel pipette.
Protocol was successful

Protocol 04 – Addition of carboxylic acids to the reaction plate
65 acids (252 ul, 0.126 mmol, 1.07 eq, 500 mM in DMA, *cf acids_list.csv*) stored in 24-vial racks were added to the reaction plate already containing DIPEA and T3P, one acid per well, using the 1000 ul single channel pipette.
Protocol was successful.

Protocol 05 – Addition of the main imidazole-amine reactant to the reaction plate
(1H-imidazol-1-yl)(piperazin-1-yl)methanone (121 ul, 0.118 mmol, 1.0 eq, 978 mM in DMA/DIPEA, *cf main_reactant.csv*) stored in 24-vial rack was added to the reaction plate already containing DIPEA, T3P, and the acids. The reactant was added to each of the 65 reaction wells using the 1000 ul single channel pipette. The reaction plate was sealed, and stored overnight at rt. The reactions were concentrated before the imidazolium salt formation.
Protocol was successful, although a change of colour in the last 6 reaction wells might indicate that some of the amine crashing out of solution.

No protocol – Urea worku
Due to time constraints and logistical issues (many series run in parallel) workup of the coupling reaction was done by manual pipetting. DCM (300uL) and saturated aqueous $NaHCO_3$ solution (300uL) were added to the wells using the 8-channel 300 µL pipette. After aspiration/dispense mixing cycles, the organic extracts (260 uL) were transferred to another 2 ml 96-well plate. More DCM (300 ul) was added and the two phases mixed. The organic extracts were combined and the solvent removed (SPE-dry).

Protocol 06 – Imidazolium salts formation
DMA (500uL) was added followed by iodomethane (75 ul, 1.21 mmol, 10 eq) using the 8-channel pipette. The reaction plate was sealed, and stored overnight at rt. The reactions were concentrated before the urea synthesis setup
The protocol was successful.

Protocol 07 – Addition of solvent (DMA) to the crude imidazolium salts.
DMA (800 ul) was added to the 64 imidazolium salts using the 8-channel pipette.
Protocol successful

Protocol 08 – Mixing of the imidazolium salt solutions before transfer to ura synthesis reaction plates.
Sequential aspiration (300 ul) and dispense (300 ul) using the 300ul 8-channel pipette.
Protocol successful

Protocol 09 – Transfer of imidazolium salt solutions to urea synthesis reaction plate
An aliquot (100ul) of each row of the source plate containing the imidazolium salt solution was transferred seven times to seven 2ml 96-well reaction plates to make seven copies of the original plate and have 8 plates availables for the urea synthesis with 8 different amines, one per plate. The liquid transferred was done using the 8-channel 300 ul pipette. K_2CO_3 (2.9 mg, 0.021 mmol, 1.5 eq) was added to each reaction well.

Protocol was successful although run 4 times.
3 copies were made at once on the deck, twice then a last run made the last copy.
A coding error would have transferred to the 10th row, but was prevented.

Protocol 10.1 – Stock solution preparation of eight amines for urea reactions.
To 8 amines in 4ml vials (24-vial racks) was added DMA using the 1 ml single-channel pipette. Once the stock solution prepared (333 mM), the vials were capped.
Protocol successful.

Protocol 10.2 – Transfer of eight amines to each of the eight urea synthesis reaction plates
One amine (43 ul, 0.014 mmol, 1.0 eq, 333 mM in DMA, *cf amines_for_urea_reaction.csv*) in storage vials was transferred to each of the 64 reaction wells, already containing the imidazolium salt (0.015 mmol, 1.05 eq) in DMA (100 ul) in one reaction plate. The protocol was repeated seven times using the 1ml single channel pipette, setting up 512 urea synthesis reactions on eight different 2ml 96-well plates. The reaction plates were sealed and stored for 24 h.
Run 1. Protocol successful although the protocol made the robot change tip for every transfer, and not pick up the amine from the vial in the C1 position of the 24-vial rack.
Run 2. Pipette did not pick enough solution in the D1 source vial (calibration tip too low) and did not transfer the amine in a few wells. This was manually rectified.
Run 3. After recalibration, protocol successful
Run 4. Not enough to start the last 2 reaction. Added manually
Run 5. After recalibration, protocol successful
Run 6-8. Protocol successful.

Protocol 11 – Add more DMA to reaction wells
Before taking an aliquot for LCMS analysis, DMA (100 ul) was added to each reaction well.
3 runs were necessary, 1 to preparing 2 plates and 2 preparing 3 plates. All runs were successful.

Protocol 12 – Pre-workup LCMS analysis sample preparation
Using the 300 ul 8-channel pipette MeCN (100ul) was added to a low-profile 96-well plate followed by the crude reaction mixtures (15 uL), one column at a time. The protocol was repeated 7 times, all runs successful. All reaction mixtures were subsequently concentrated (SPE-dry)

Protocol 13-1 – Urea Workup – Addition of aqueous solution and DCM to the crude residues

Addition of DCM (300uL) and saturated aqueous $NaHCO_3$ solution (300uL) using the 8-channel 300 µL pipette. Organic and aqueous phases were then mixed by a series of dispensing and aspiration (300 uL each) using the 8-channel 300 µL pipette.

Run 1. Fall of a tip which required code adjustements n the next run

Run 6. Fall of a tip. Protocol stopped and manual mixing of the last rows.

Run 8. On channel not aspirating as much as it should. Maintenance required

Run 2-5 and 7. Protocol successful

Protocol 13-2 – Urea Workup – Removal of the bottom DCM phase

Removal of the bottom DCM phase (250 µL) and transfer to a low-profile 96-well storage plate using the 8-channel 300 µL pipette. The organic extracts were then concentrated (SPE-dry)

Run 1. Protocol successful but loop iteration to correct in the protocol (two extra rows)

Run 2-8 Protocol successful

Protocol 14 – Add ethylene glycol to crude ureas

EG (80 ul) was added to each well containing a urea residue, using the 300 ul 8-channel pipette. The protocol (4 plates at a time) was executed twice and the resulting solution concetration was estimated at 164 mM.

Run 1. Destination plate not well calibrated but transfer successful

Run 2 Protocol successful

Protocol 15 – Transfer of crude reaction mixtures to Screen and QC plates

An aliquot (20 ul) of each crude ureas was transferred across 8 low-profile 96-well plate followed by the addition of MeCN (100ul) and water (20 ul). Another aliquot (30 ul) was transferred to two 384 PP echo-compatible Agilent plates, all transfers were done using the 8-channel pipette.

Run 1 and 2. Only the LCMS plates were prepared as the corresponding plates had already been manually prepared

Run 3-8. Protocol successful

7.6 Iteration 4.0

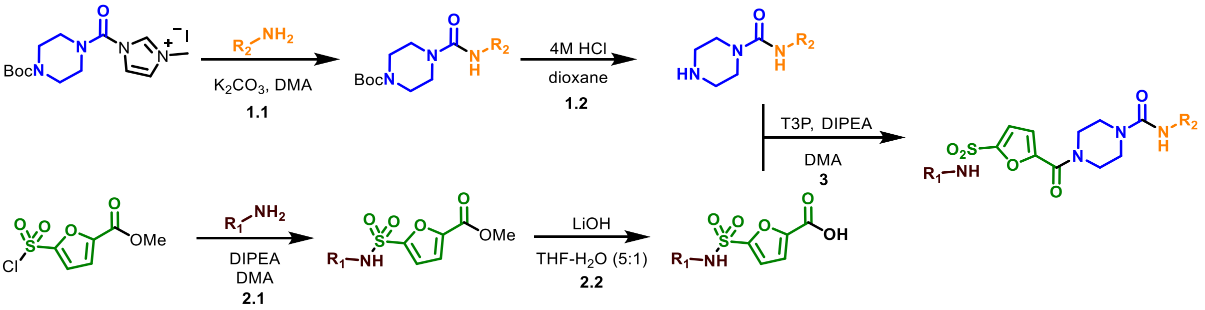

Scheme S6 – Parallel synthesis used for iteration 4.2

Experimental details

Urea syntheses and Boc deprotection

Ten amines (314 ul, 0.440 mmol, 1.10 eq, 1.4 M in DMA) were dispensed in the first 10 wells of a 2ml 96-well reaction plates, already containing K_2CO_3 ((83 mg, 0.60 mmol, 1.5 eq)) and 1-(4-(tert-butoxycarbonyl)piperazine-1-carbonyl)-3-methyl-1H-imidazol-3-ium iodide (1600 ul, 0.400 mmol, 1.0 eq, 250 mM in DMA). The reaction plate was sealed and stored overnight at rt. The reaction mixtures were concentrated (SPE-dry) and partitioned in DCM (500uL) and saturated aqueous NH_4Cl, an aqueous solution (500 uL). Aqueous HCl (2M, 500 ul) was then added to help phases separation and the organic extracts were transferred (500uL) to a 2mL 96-well plate. Each aqueous phases was extracted a second time with DCM (500uL). The organic extracts (450uL) were added to the 96-well plate containing the first extracts and the DCM was removed overnight. 4M HCl in 1,4-dioxane (300 µL, excess) was added to each reaction well. The reaction plates were sealed and stored at ambient temperature for 16 h. The resulting mixtures were concentrated under reduced pressure using the blowdown apparatus, then DMA was added (1700 uL)

Sulphonamide syntheses and Ester hydrolysis

Methyl 5-(chlorosulfonyl)furan-2-carboxylate (133 ul, 0.2 mmol, 1.0 eq, 1.5 M in DMA) was added in the first 20 wells of a 2mL 96-well reaction plate. To each well was dispensed one amine (420 ul, 0.210 mmol, 1.05 eq, 500 mM in DMA) followed by DIPEA (42 ul, 0.240 mmol, 1.20 eq). The reaction plate was sealed and stored overnight at rt. The reaction mixtures were concentrated (SPE-dry) and 1,4-dioxane (800 ul) was added before each crude solution was transferred to 10 wells of a 2ml 96-well reaction plate (80 ul, 0.0195 mmol), therefore preparing 200 reaction wells across 3 reaction plates. Hydrolysis of the methyl esters was eventually achieved using lithium hydroxide (*cf protocol 13 below for more details*) and the crude acids were concentrated (SPE-dry)

3. Coupling between deprotected ureas and acid sulfonamides

To each acid sulfonamide residue (0.02 mmol, 1 eq) contained in three 96-well plates DMA (100 ul), DIPEA (9 ul, 0.5 mmol, 2.5 eq) and T3P (14.3 ul, 0.24 mmol, 1.2 eq, 50% in EtOAc) were added. Next the 10 crude Boc-deprotected ureas (85 ul, 0.02 mmol, 1.0 eq) were added to the reaction wells. Each urea was transferred 20 times, along one column over the the 3 reaction plates (e.g urea 1 was transferred to column 1 of reaction plate 1, 2, 3; urea 2 to column 2 of plate 1, 2, 3 etc.). The 3 reaction plates containing 200 amide coupling reactions were sealed and stored at ambient temperature for 72 hours. The reaction mixtures were concentrated partitioned in DCM (300uL) and saturated aqueous $NaHCO_3$ solution (300uL) followed by a mixing stage. To improve phase separation and inorganic solubilisation, water was added (100 ul). Further mixing allowed the transfer of a fraction of the organic extracts to low-profile 96-well plates (100 ul). More DCM (250 ul) was added and after partition transferred to the plates containing the first organic extracts. DCM was removed overnight. EG (80 µL) was added and an aliquot (15 uL) of each solution was transferred to 96-well low volume plates for LCMS analysis. Upon LCMS analysis of all the reaction mixture, the sequence dd not yield any desired products. One main reason, aside for experimenting a five step reaction for the first time, was the unexpectedly difficult hydrolysis of the methyl ester, resulting on the accumulation of inorganic in the crude mixtures before the final coupling step.

Changes for the new iteration:

- Workup after sulfonamide formation.
- Use of dioxane and 2M aqueous LiOH for the hydrolysis and workup after the reaction
- Check pH during workup

- Improve building blocks solubility. Transfer at steps where the crudes are known to be fully soluble in a given solvent.

Protocols

Urea and Boc-deprotection
01_Urea_Coupling_SC1000_Imidazolium_stocksolution_trough.py
02_Urea_Coupling_SC1000_Imidazolium_stocksolution_troughto96_2ml.py
03_Urea_Coupling_SC1000_reactants_stocksolution_trough_vialstorage.py
04_UreaCoupling_SC1000_reactants_addition_storage_96.py
14_1_Ureas_workup_96.py
14_2_Ureas_pWUpBottomPhaseTransfer_96to96_multi300.py
15_Ureas_MC300_HCl-dioxane_reagent4_troughTo96.py
16_Ureas_MC300_add_DMA_troughTo96.py

Sulfonamide reaction and ester hydrolysis
05_Sulfonamide_SC1000_reactants_stocksolution_trough_vialstorage.py
06_Sulfonamide_SC1000_SulfonylCl_stocksolution_trough.py
07_Sulfonamide_SC1000_SulfonylCl_stocksolution_troughto96.py
08_Sulfonamide_SC1000_reactants_addition_storage_96.py
09_Sulfonamide_MC300_base_troughTo96.py
10_QC_96to96_multichannel.py
11_Sulphonamide_MC300_dioxane_add_troughTo96.py
12_Sulphonamide_MC300_dispense_3to10times96.py
13_Sulphonamide_MC300_LiOH_dioxane_troughTo96.py
13b_Sulphonamide_MC300_LiOH_dioxane_troughTo96.py

Combined
17_MC300_solvent_base_reagent_troughTo96.py
17bis_MC300_solvent_base_reagent_troughTo96.py
18_Urea_SC1000_dispense_96To3times96_col.py
19_QC_96to96_multichannel_one_plate.py
19b_QC_96to96_multichannel - add water.py
20_Ureas_workup_96.py
20b_Ureas_workup_lower_96 .py
20c_ADD_DCM_AGAIN_Ureas_workup_lower_96.py
21_pWUpBottomPhaseTransfer_96to96_multi300.py
21b_LOWER_pWUpBottomPhaseTransfer_96to96_multi300.py
21c_LOWER_pWUpBottomPhaseTransfer_96to96_multi300.py
22_ADDEG_trough__96.py
23_pWUpQC_AddEG_troughTo96_multi300_int2.py
23_pWUpScreenSolventDispensing_troughTo96_multi300.py
24_pWUpQC_Screen_96to96_96to384_troughTo96_multi300.py

Protocol 01 – Preparation of imidazolium salt stock solution
To 1-(4-(tert-butoxycarbonyl)piperazine-1-carbonyl)-3-methyl-1H-imidazol-3-ium iodide (2.9 g, 6.87 mmol) in a 4-well trough was added DMA (27.5 mL) to make a 250 mM stock solution using the 1mL single channel pipette.
Solubility of the imidazolium salt was an issue; ideally the stock solution was supposed to be 1.5M but the protocol had to be repeated 5 times to get most of the salt in solution.

Protocol 02 – Transfer of the imidazolium salt to reaction plate
Using the 1ml single channel pipette the imidazolium salt (1600 ul, 0.400 mmol, 1.0 eq, 250 mM in DMA) contained in the 4-well trough was transferred to the first 12 wells of a 2ml 96-well reaction plate.
The solution was manually stirred during the transfer of the imidazolium to the reaction plate to avoid solid formation. A coding error resulted in the preparation of 10 reaction wells +2 negative controls instead of the 12 originally planned. No manual correction was made and it was decided to start 10 urea syntheses instead of 12.

Protocol 03 – Preparation of amines stock solution for the urea synthesis
To 10 amines in 4ml vials (24-vial racks) was added DMA using the 1 ml single-channel pipette. Once the stock solution prepared (1.4 M), the vials were capped.
Protocol successful.

Protocol 04 – Transfer of amines to the reaction plate
A fraction of the 10 freshly prepared amine stock solutions (314 ul, 0.440 mmol, 1.10 eq, 1.4 M) was added to 10 of the 12 wells of a 2ml 96-well reaction plate already containing the imidazolium salt using the 1ml single-channel pipette. K_2CO_3 (83 mg, 0.60 mmol, 1.5 eq) was then manually added to each reaction and the reaction plate was sealed and stored for 24 h at rt. The reaction mixtures were then concentrated (SPE-dry)
Protocol was successful.

Protocol 05 – Preparation of amines stock solution for the sulfonamide synthesis
To 20 amines in 4ml vials (24-vial racks, *cf Amines_Sulfonamides.csv*) was added DMA using the 1 ml single-channel pipette. Once the stock solution prepared (500 mM), the vials were capped.
Protocol successful.

Protocol 06 – Sulfonyl chloride stock solution preparation
To methyl 5-(chlorosulfonyl)furan-2-carboxylate (2.0 g, 8.9 mmol, 1.0 eq) in a 12-well trough was added DMA (5.9 mL) to make a 1500 mM stock solution using the 1mL single channel pipette.
Protocol successful.

Protocol 07 – Transfer of the sulfonyl chloride to a reaction plate
Using the 1ml single channel pipette sulfonyl chloride (133 ul, 0.2 mmol, 1.0 eq, 1.5 M in DMA) contained in the 12-well trough was transferred to the first 22 wells of a 2ml 96-well reaction plate.
Protocol successful

Protocol 08 – Transfer of the amines to the reaction plate containing the sulfonyl chloride
Using the 1ml single channel pipette 20 amines (420 ul, 0.210 mmol, 1.05 eq, 500 mM in DMA) contained in a 24-vial rack were transferred to the first 20 wells of the 2ml 96-well reaction plate containing the sulfonyl chloride solution, one amine per well starting from C1 (A1 and B1 serving as negative reaction controls.)
Protocol run twice, robot collision with a capped vial.

Protocol 09 – Transfer of the reaction base to the sulfonamide reaction plate
Using the 300 ul 8-channel pipette DIPEA (42 ul, 0.240 mmol, 1.20 eq) was transferred to the first 3 columns of the 2ml 96-well reaction plate containing sulfonyl chloride and amine solution. The reaction plate was sealed and stored for 24 h at rt.

Protocol not successful because of loss of calibration that happens sometimes with OT1, with unknown cause. The liquid handling step was done manually, and the calibration file restored the day after using a backup.

Protocol 10 – Crude sulfonamide reaction aliquots for LCMS analysis
Using the 300 ul 8-channel pipette MeCN (100 ul) was transferred to the first 3 columns of the low-profile 96-well plate. An aliquot (15 ul) of each sulfonamide reaction mixture was then transferred to the plate. The reaction mixtures were then concentrated (SPE-dry)
Protocol successful although error with number of liquid transfer (4 rows instead of 3, corrected).

Protocol 11 – Addition of 1,4-dioxane to crude sulfonamides.
Using the 300 ul 8-channel pipette 1,4-dioxane (800 ul) was added to the crude sulfonamide residues.
Protocol successful.

Protocol 12 – Transfer of the crude sulfonamide solution to 200 reaction vessels
Using the 300 ul 8-channel pipette an fraction of each crude sulfonamide solution (80 ul, 0.0195 mmol) was transferred to 10 wells of a 2ml 96-well reaction plate, therefore preparing 200 reaction wells across 3 reaction plates.
Run 1. Pipette height needed to be adjusted as no solution was aspirated on the tenth transfer of the first row. The protocol was stopped and it is apparent that there is less volume.
Run 2. After pipette height adjustment, the protocol was successful for row 2 and 3. Sulfonamides 19 and 20 were however only partly soluble and clogging slightly the tip decreasing the accuracy of the dispensing for these two sulfonamides.

Protocol 13 – Methyl ester hydrolysis of the crude sulfonamides with Lithium hydroxide
$LiOH.H_2O$ (1mg, 0.024 mmol, 1.2 eq) was manually added to each reaction well. No conversion was observed.
More solvent was added (320 ul 1,4-dioxane, *cf 13_Sulphonamide_MC300_LiOH_dioxane_troughTo96.py*) with the same outcome. The reaction mixtures were concentrated. A lithium hydroxide solution (0.07 M in 1,4-dioxane) was then added (cf *13b_Sulphonamide_MC300_LiOH_dioxane_troughTo96.py*) with an equally poor conversion.
The reactions were concentrated and more 1,4-dioxane (320 ul) and 1M LiOH in 1,4-dioxane (80 ul) were added. After 24 h, LCMS analysis showed completion of the hydrolysis reaction. The reaction mixtures were concentrated (SPE-dry)

Protocol 14.1 – Urea workup – Add DCM and aqueous solutions
Addition of DCM (500uL) and saturated aqueous NH_4Cl solution (500uL) using the 8-channel 300 µL pipette. Organic and aqueous phases were then mixed by a series of dispensing and aspiration (300 uL each) using the 8-channel 300 µL pipette.

Run 1. Protocol successful but ammonium chloride not helping with phase separation and salt solubilisation.

Run 2. Protocol modified to add 500 ul of 2M HCl. Much better separation after phase mixing.

Protocol 14.2 – Urea Workup – Removal of the bottom DCM phase

Removal of the bottom DCM phase (500 µL) and transfer to 2ml 96-well plate using the 8-channel 300 µL pipette.

Run 1. 250ul instead of 500 DCM were transferred so the protocol was run twice. It was later amended to take out 450 ul at once.

Protocol 14.3 – Urea Workup – Second extraction

The same protocol as 14.1 was amended, this time to only add DCM (500ul)

Protocol 14.4 – Urea Workup – Removal of the bottom DCM phase

Organic extracts (450 ul) from second extraction were transferred to the plate containing the first extracts. The combined organics were then concetrated (SPE-dry)

Protocol 15 – Urea Boc deprotection – Addition of 4M HCl-1,4-dioxane

To the crude urea residues was added with the 8-channel pipette HCl in 1,4-dioxane (300 ul, excess). The reaction plate was sealed and stored overnight at rt. The mixtures were then concentrated (SPE-dry).
Protocol successful

Protocol 16 – Urea/Sulfonamide Coupling – DMA addition to Boc-deprotected ureas before transfer to coupling reaction plates

To the crude Boc-deprotected urea residues DMA (1700 ul) was added with the 8-channel pipette.
Protocol successful with the salts coming in solution.

Protocol 17 – Urea/Sulfonamide Coupling – T3P and DIPEA addition to crude acid sulfonamide residues

To the 200 reaction wells contained in three 96-well plate were added DMA (100 ul), DIPEA (9 ul, 0.5 mmol, 2.5 eq) and T3P (14.3 ul, 0.24 mmol, 1.2 eq, 50% in EtOAc)
Run 1. Protocol successful but there were concerns about the T3P dispensing. With the air gap a few samples appeared to not get a drop, probably due to the low volume and viscocity of the reagent. Run 2. Protocol amended to add 42 ul of T3P in solution with DMA (1/2 ratio).

Protocol 18 – Urea/Sulfonamide Coupling – Coupling reaction set-up with the addition of crude Boc-deprotected ureas to the DMA solution containing crude acid-sulfonamides, T3P and DIPEA

To the 200 reaction wells contained in three 96-well plate were added the 10 crude ureas (85 ul, 0.02 mmol, 1.0 eq). Each urea was transferred 20 times, along one column over the the 3 reaction plates (e.g urea 1 was transferred to column 1 of reaction plate 1, 2, 3; urea 2 to column 2 of plate 1, 2, 3 etc.)
The first run was stopped after the first row to amend the protocol and add an air gap between each transfer. Run 2 was successful. Less soluble urea 3 was not dispensed across sulfonamides 18-20.

Protocol 19 – Urea/Sulfonamide Pre Workup QC – Transfer of reaction aliquot to 96-well plate for QC analysis

After 24 h an aliquot (15 ul) of each of the 200 amide coupling reactions was transferred to low-profile 96-well plate for pre-workup LCMS analysis, using the 8-channel pipette. The reaction mixtures were then concentrated (SPE-dry)

Protocol successful, with addition of MeCN (100 ul) first, then the aliquot

Protocol 20/21 – Urea/Sulfonamide Workup QC – Addition of saturated aquous $NaHCO_3$ and DCM, mix and transfer of organic extracts.

Addition of DCM (300uL) and saturated aqueous $NaHCO_3$ solution (300uL) using the 8-channel 300 µL pipette, followed by a mixing stage. To improve phase separation and inorganic solubilisation, water was added (100 ul). Further mixing allowed the transfer of a fraction of the organic extracts to low-profile 96-well plates (100 ul). More DCM (250 ul) was added and after partition transferred to the plates containing the first organic extracts.

Protocol 20,b,c, and 21,b,c were used to performs the steps.

Protocol 22 – Addition of ethylene glycol

Addition of ethylene glycol (80 µL) to the storage plates containing the residual crude mixtures after work-up and concentration, using the 8-channel 300 µL pipette.

Run 1. Protocol successful

Protocol 15 – Transfer of crude reaction mixtures to Screen and QC plates

An aliquot (20 ul) of each crude ureas was transferred across three low-profile 96-well plates followed by the addition of MeCN (100ul) and water (20 ul). Another aliquot (30 ul) was transferred to one 384 PP echo-compatible Agilent plates, all transfers were done using the 8-channel pipette.

Upon LCMS analysis of all the reaction mixture, the sequence dd not yield any desired products. One main reason, aside for experimenting a five step reaction for the first time, was the unexpectedly difficult hydrolysis of the methyl ester, resulting on the accumulation of inorganic in the crude mixtures before the final coupling step.

8 Compounds synthesised and synthesis QC results

This section visually summaries the success of the products attempted for the R_1 (Urea), R_2 (Amide), R_3 (Amide), R_4 (Piperazine) and R_5 (Sulfonamide) starting material cores (**Figure S1**) used for each iteration of robotic chemistry. A pale green background indicates trace success, a green background success and red background a failure with the cores shown with a white background.

A table summary of the QC data is also available on Zenodo as X-ray and reaction QC data summary.csv.

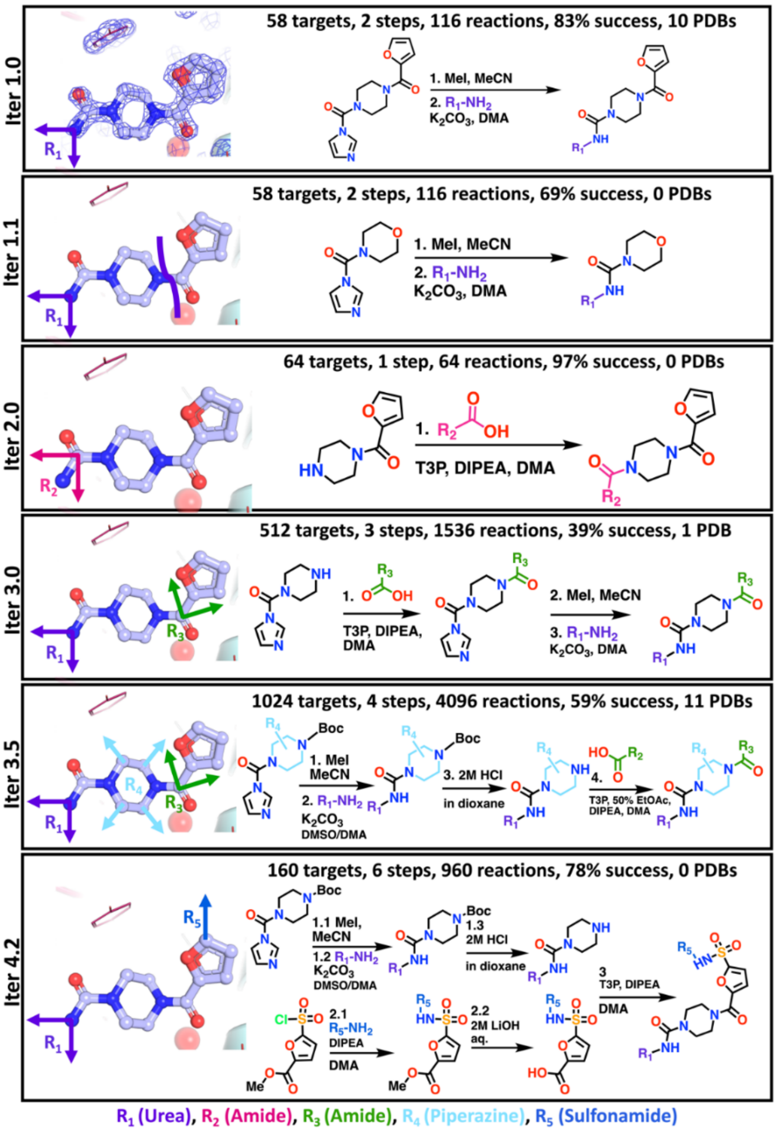

__Figure S1__: Each panel shows an iteration ("Iter") of synthetic routes and conditions, as well as (along the top): the number of fragment growth targets; number of steps performed on the robot; total number of reactions; success rates; and number of product-bound 3D protein structures (detailed below). Success rates were calculated as the fraction of CRMs for which the mass expected from the respective product could be detected in the LCMS-based quality control pipeline. The various expansion vectors are coloured like the respective functional groups listed below the final panel; and reactants are oriented to correspond to the original fragment on the left.

8.1 Iteration 1.0

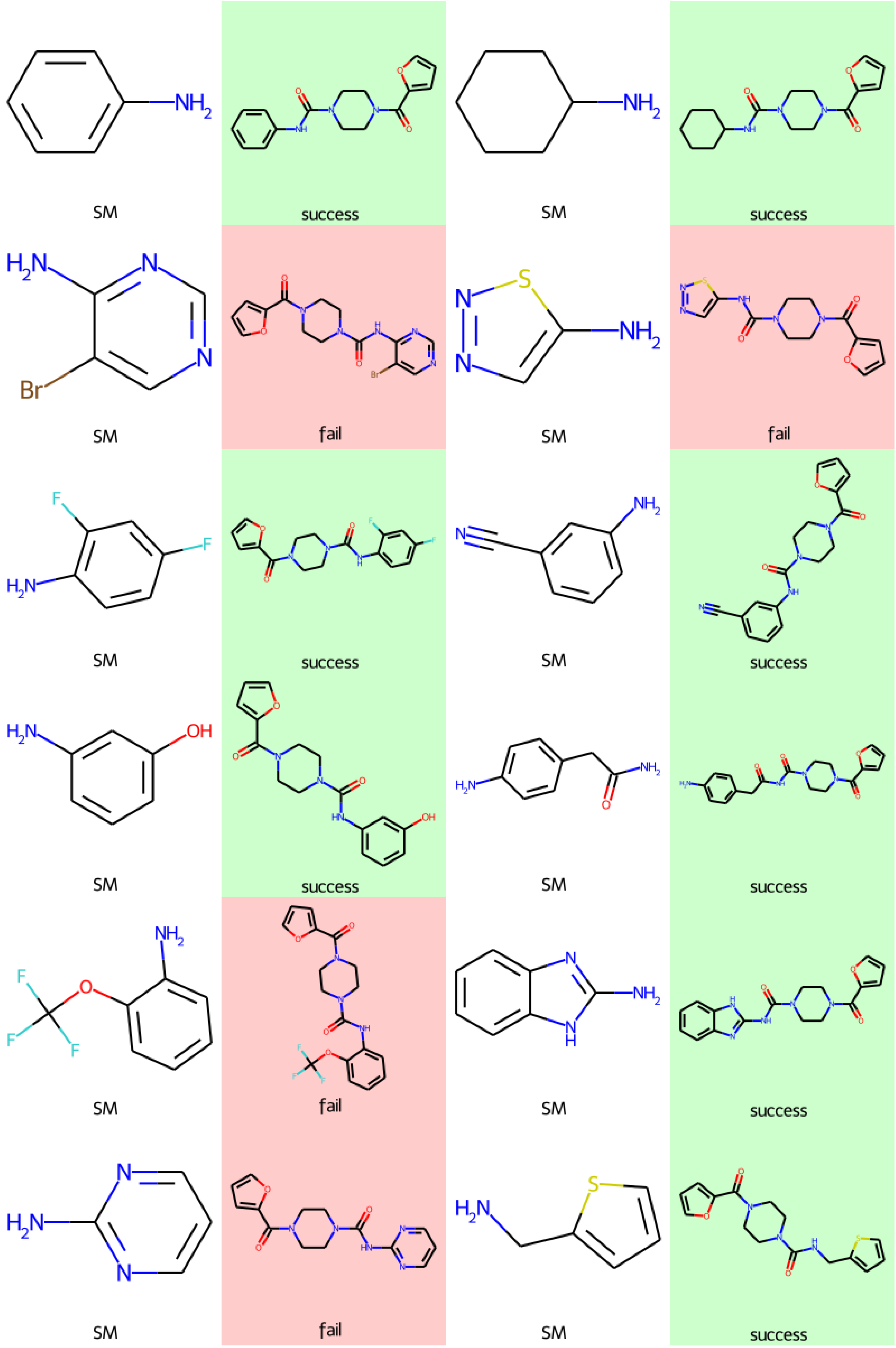
SM
success
SM
success
SM
fail
SM
fail
SM
success
SM
success
SM
success
SM
success
SM
fail
SM
success
SM
fail
SM
success

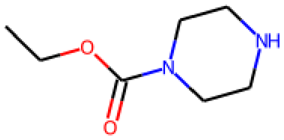

SM

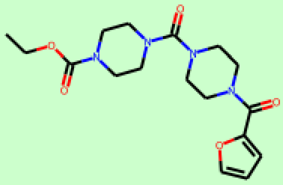

success

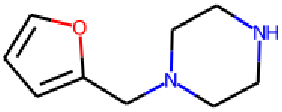

SM

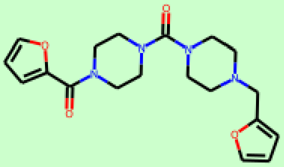

success

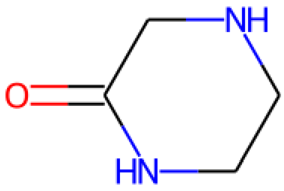

SM

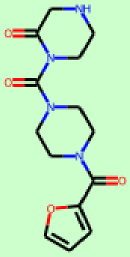

success

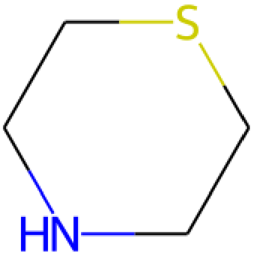

SM

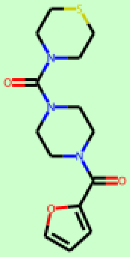

success

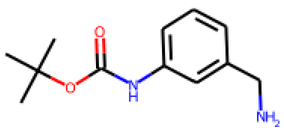

SM

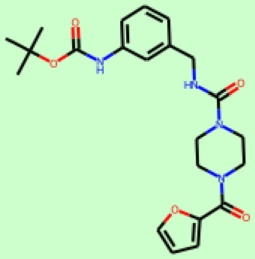

success

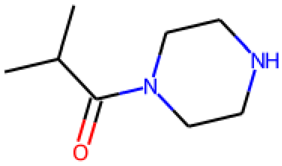

SM

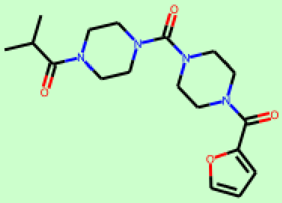

success

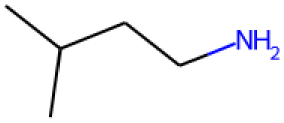

SM

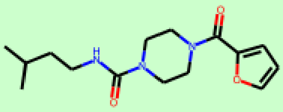

success

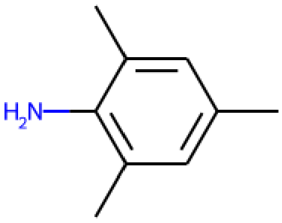

SM

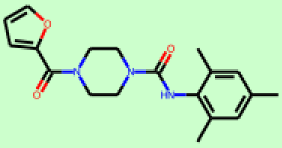

success

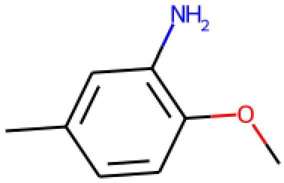

SM

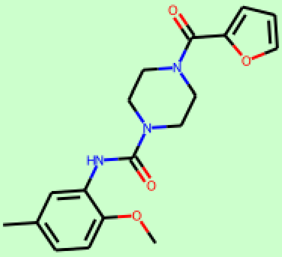

success

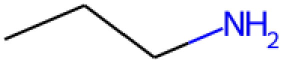

SM

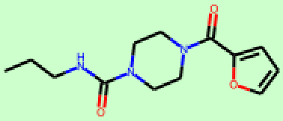

success

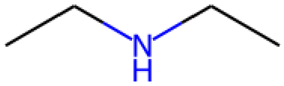

SM

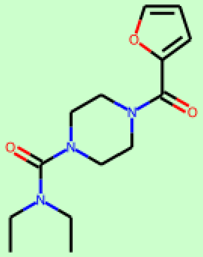

success

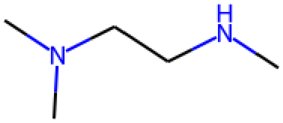

SM

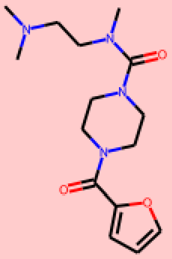

fail

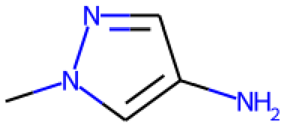
SM

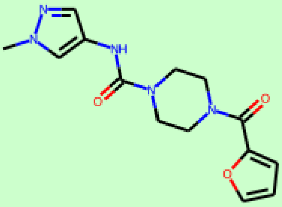
success

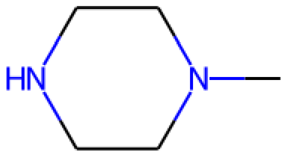
SM

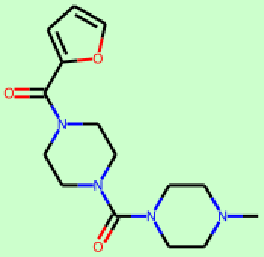
success

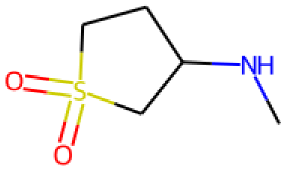
SM

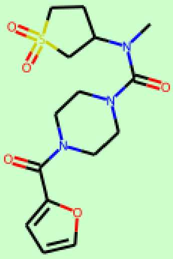
success

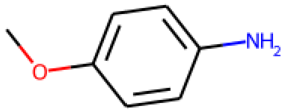
SM

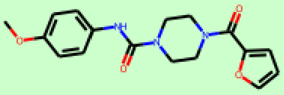
success

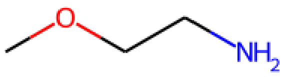
SM

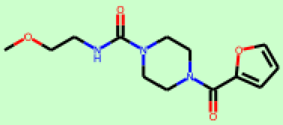
success

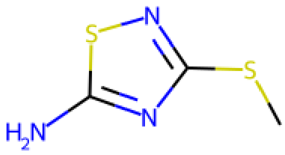
SM

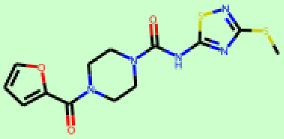
success

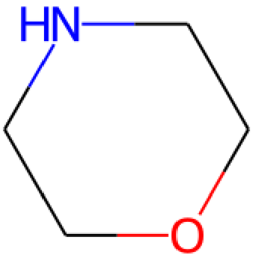
SM

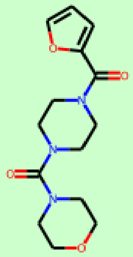
success

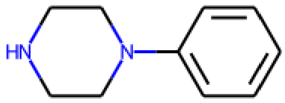
SM

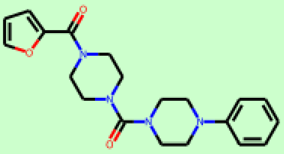
success

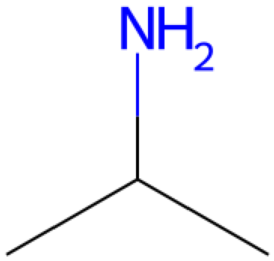
SM

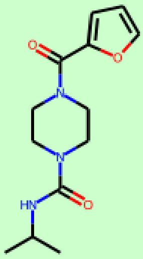
success

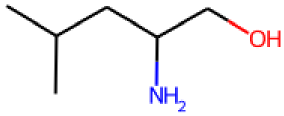
SM

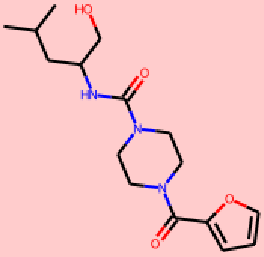
fail

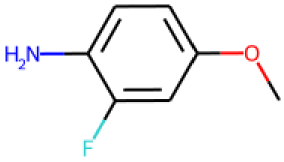
SM

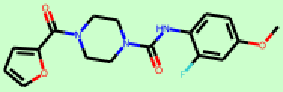
success

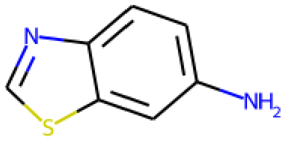
SM

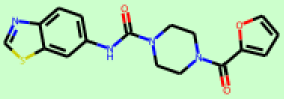
success

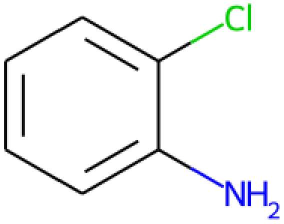

SM

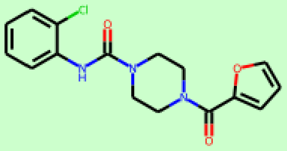

success

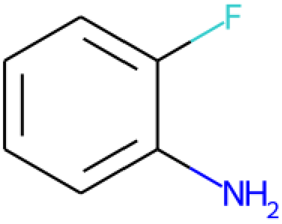

SM

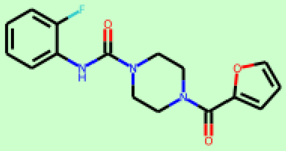

success

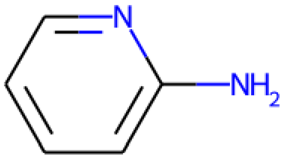

SM

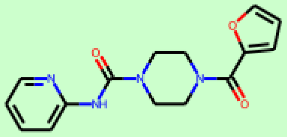

success

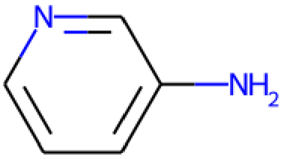

SM

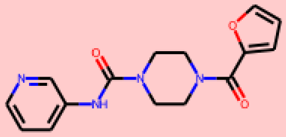

fail

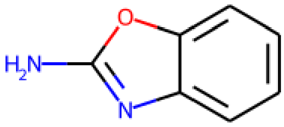

SM

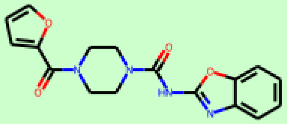

success

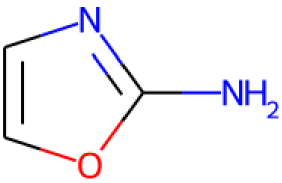

SM

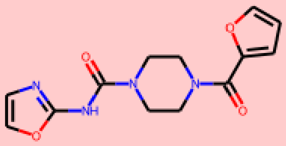

fail

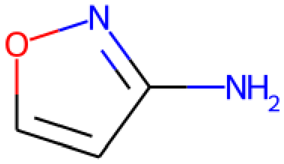

SM

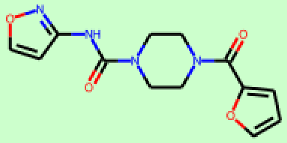

success

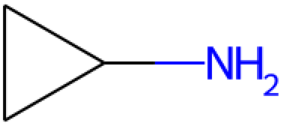

SM

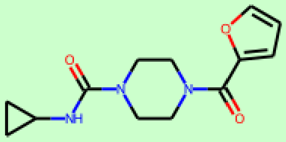

success

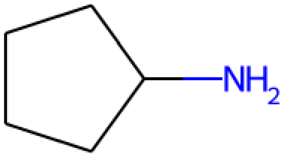

SM

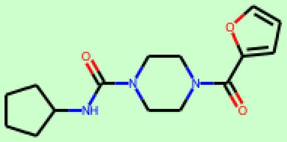

success

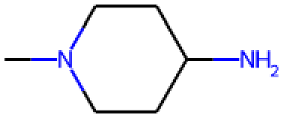

SM

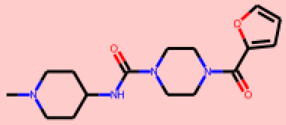

fail

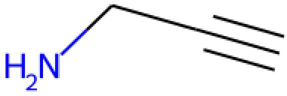

SM

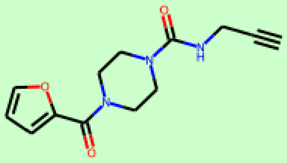

success

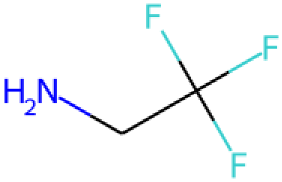

SM

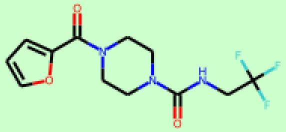

success

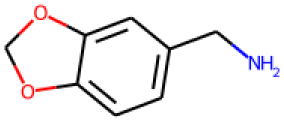
SM

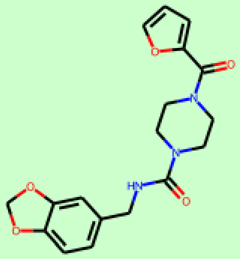
success

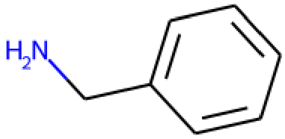
SM

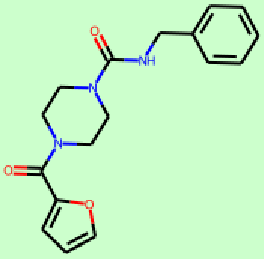
success

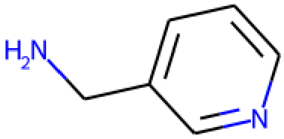
SM

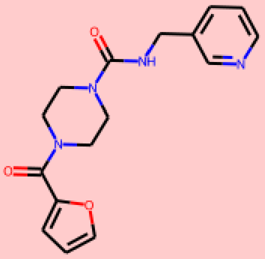
fail

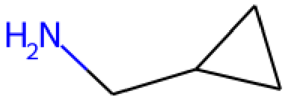
SM

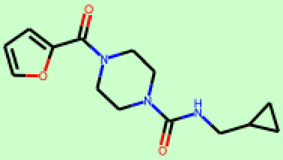
success

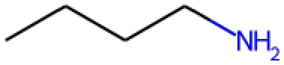
SM

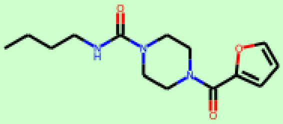
success

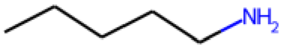
SM

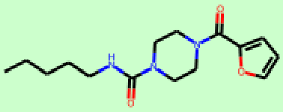
success

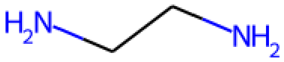
SM

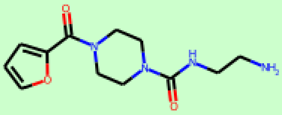
success

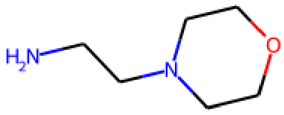
SM

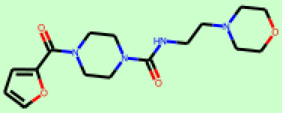
success

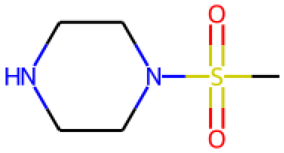
SM

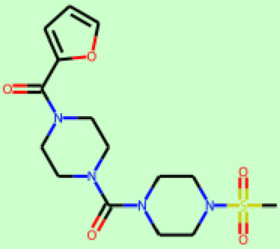
success

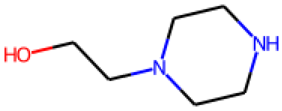
SM

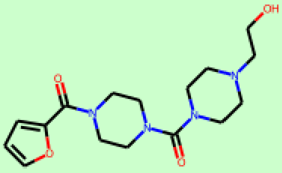
success

8.2 Iteration 1.1

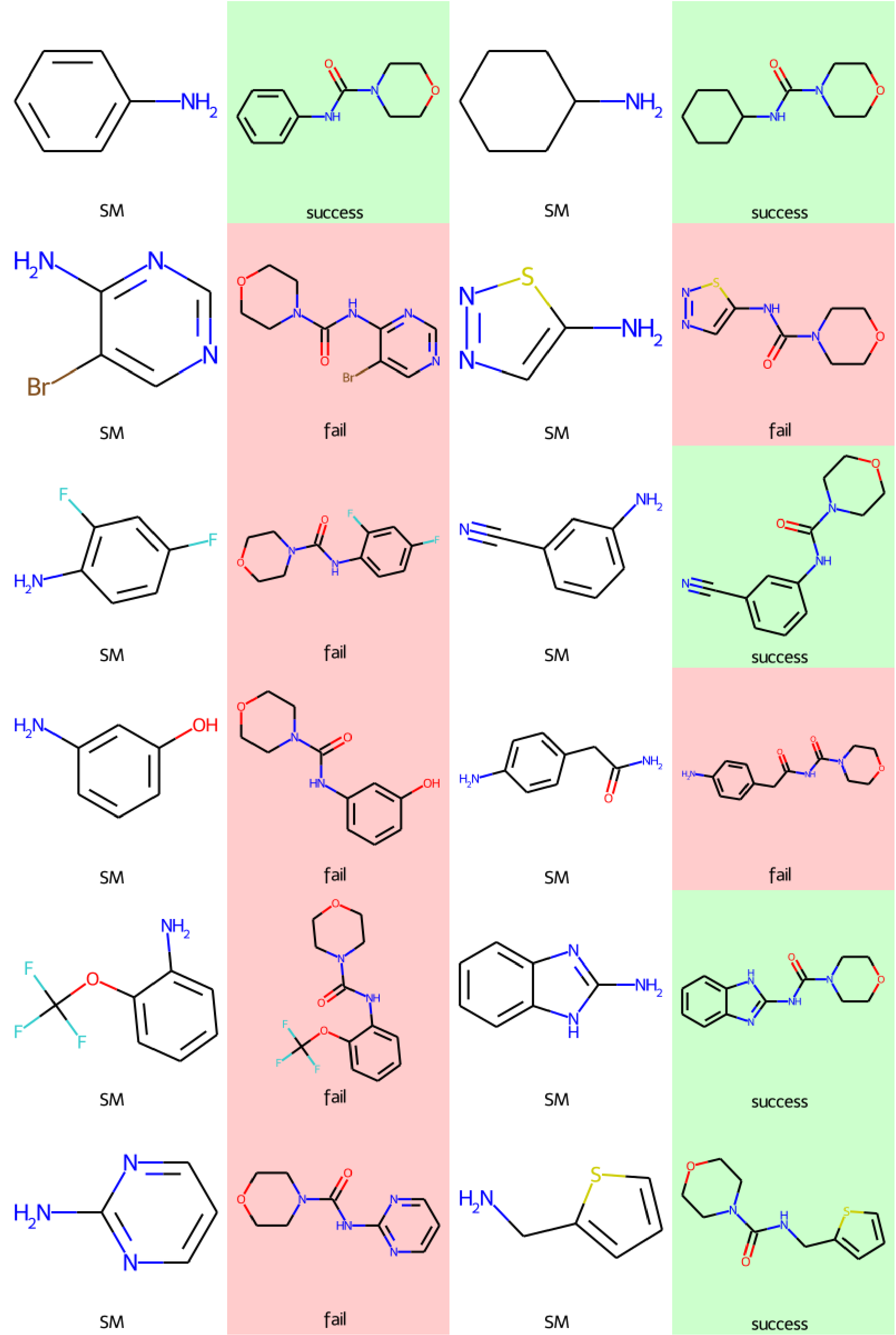
SM
success
SM
success
SM
fail
SM
fail
SM
fail
SM
success
SM
fail
SM
fail
SM
fail
SM
success
SM
fail
SM
success

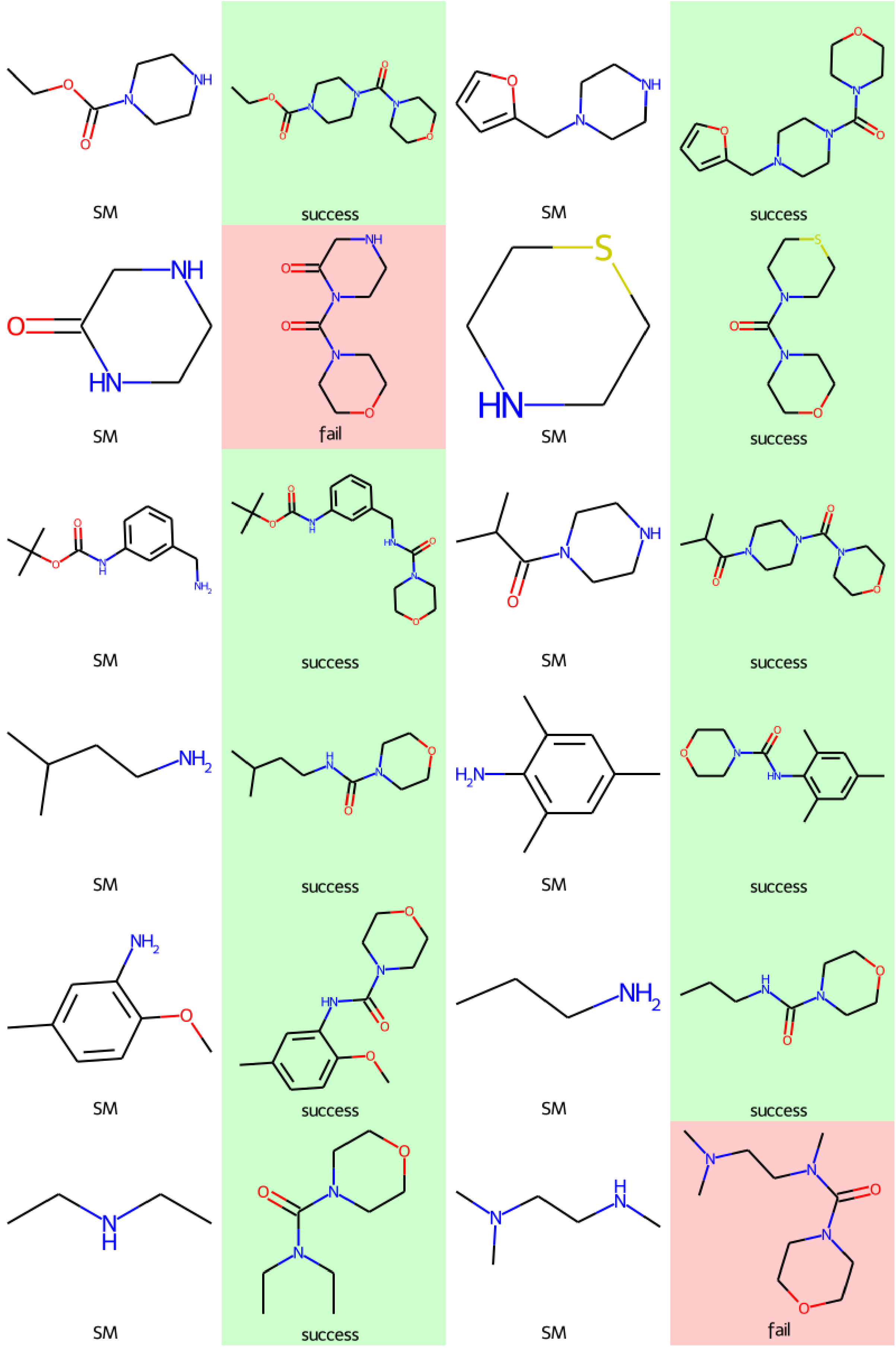
SM
success
SM
success
SM
fail
SM
success
SM
success
SM
success
SM
success
SM
success
SM
success
SM
success
SM
success
SM
fail

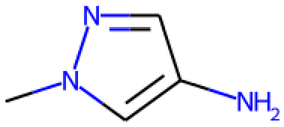

SM

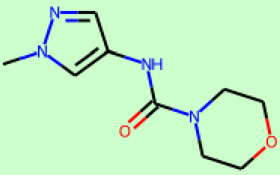

success

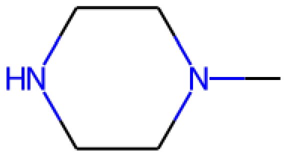

SM

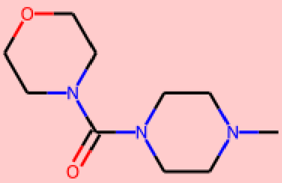

fail

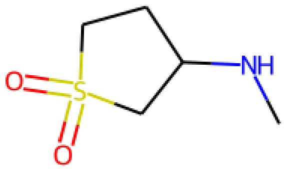

SM

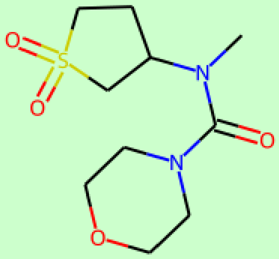

success

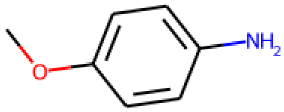

SM

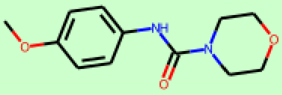

success

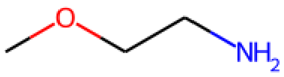

SM

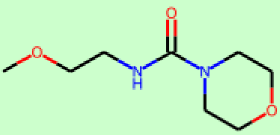

success

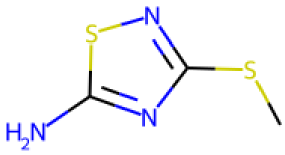

SM

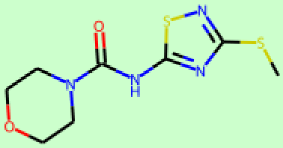

success

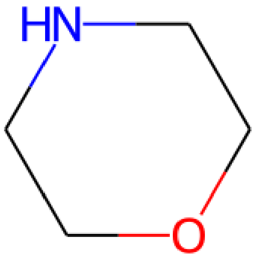

SM

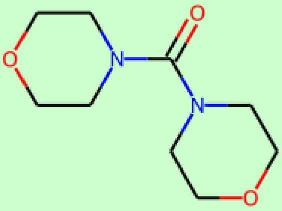

success

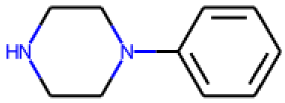

SM

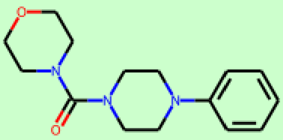

success

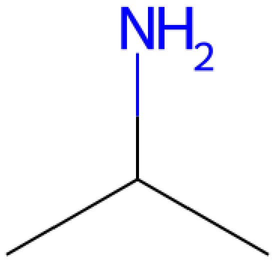

SM

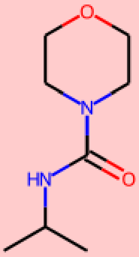

fail

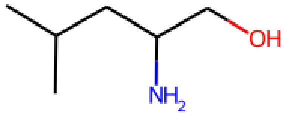

SM

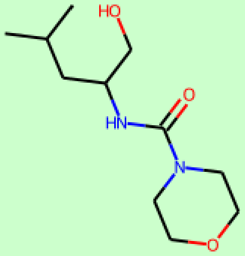

success

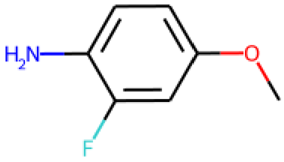

SM

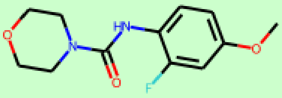

success

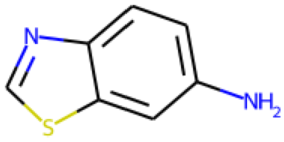

SM

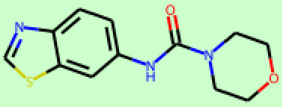

success

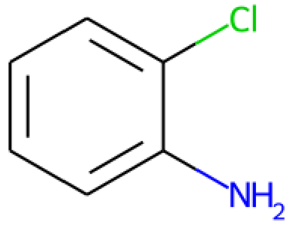

SM

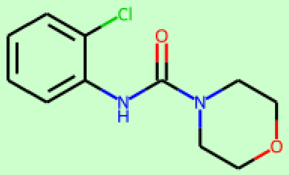

success

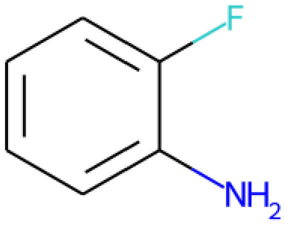

SM

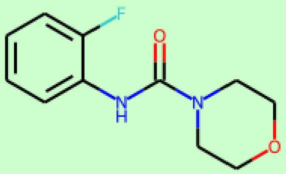

success

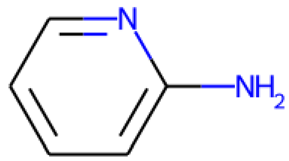

SM

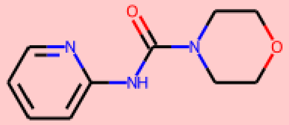

fail

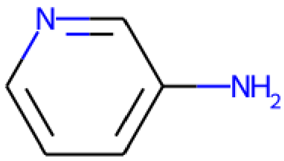

SM

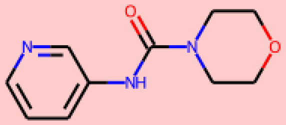

fail

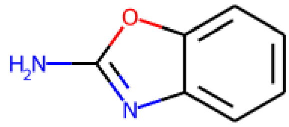

SM

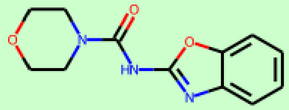

success

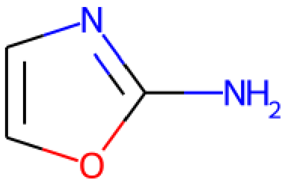

SM

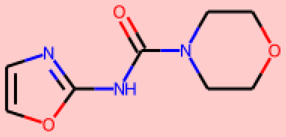

fail

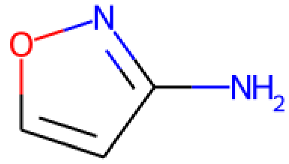

SM

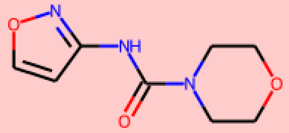

fail

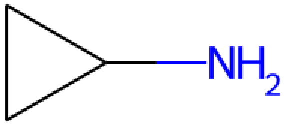

SM

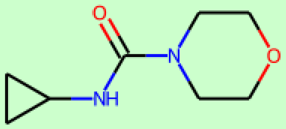

success

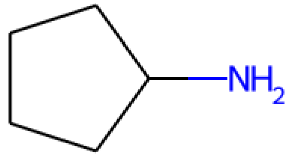

SM

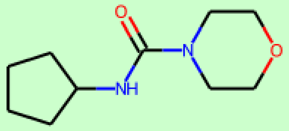

success

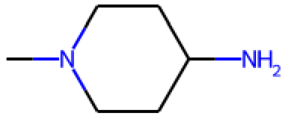

SM

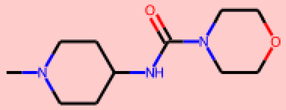

fail

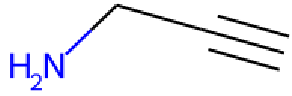

SM

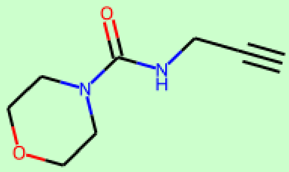

success

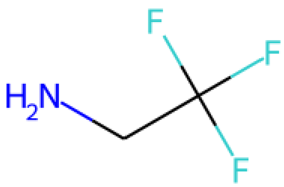

SM

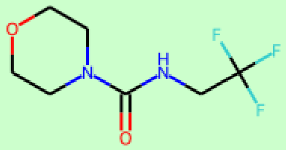

success

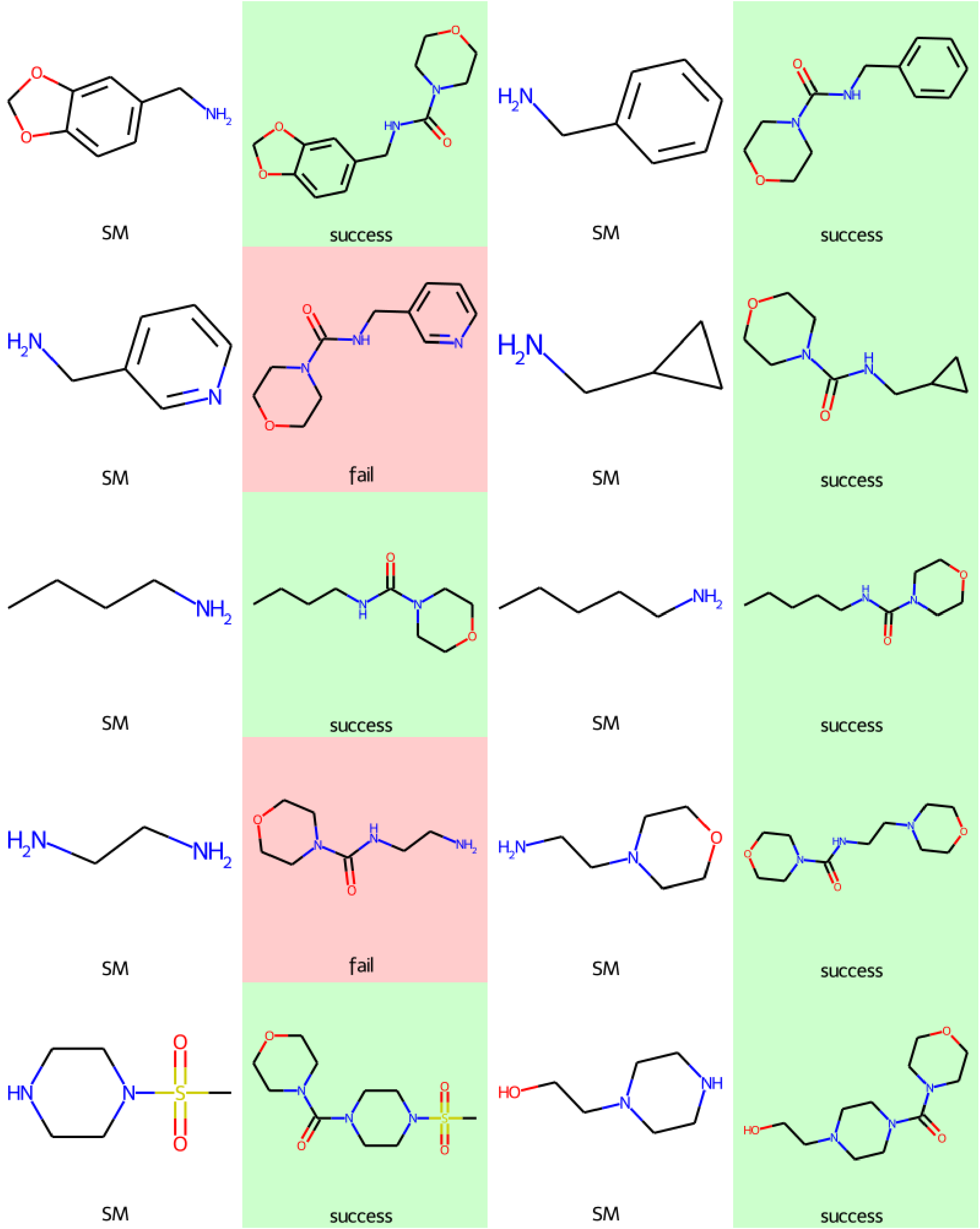
SM
success
SM
success
SM
fail
SM
success
SM
success
SM
success
SM
fail
SM
success
SM
success
SM
success

8.3 Iteration 2.0

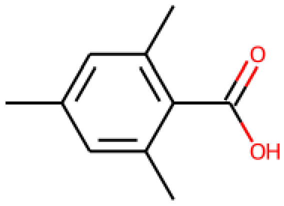

SM

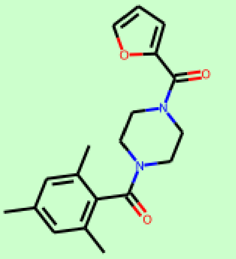

success

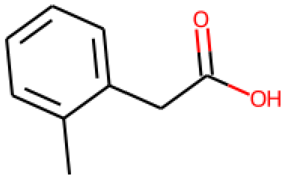

SM

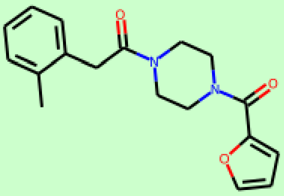

success

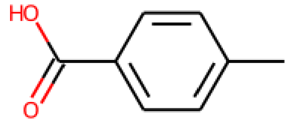

SM

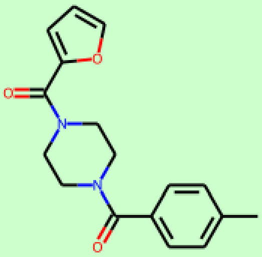

success

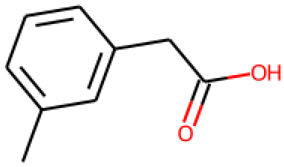

SM

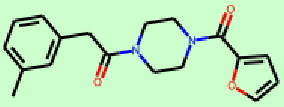

success

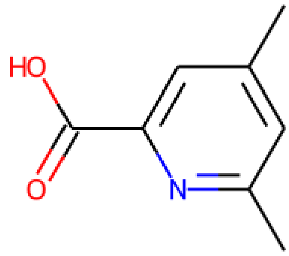

SM

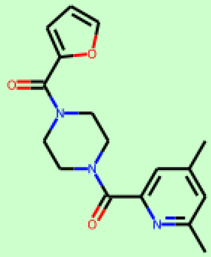

success

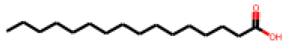

SM

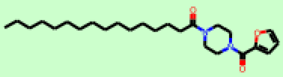

success

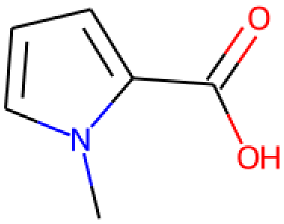

SM

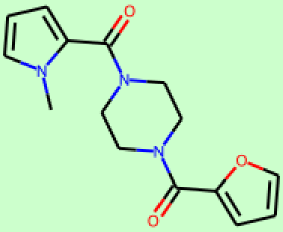

success

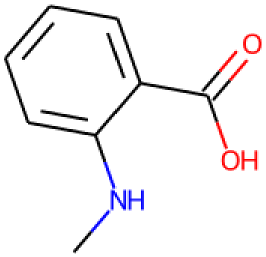

SM

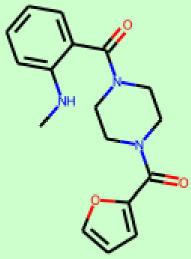

success

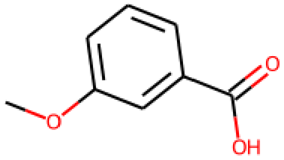

SM

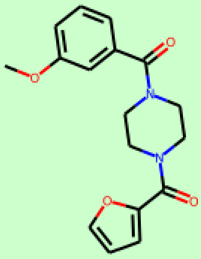

success

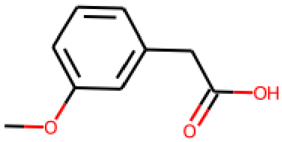

SM

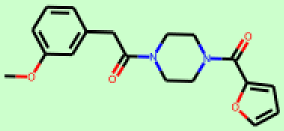

success

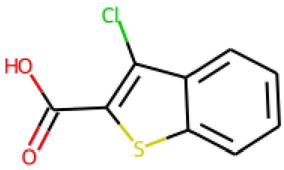

SM

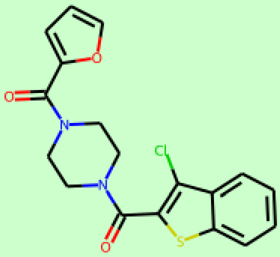

success

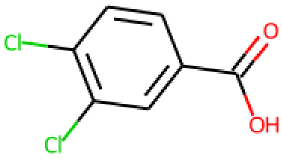

SM

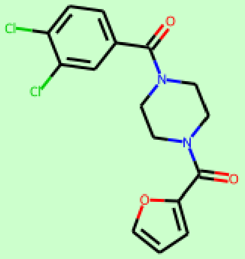

success

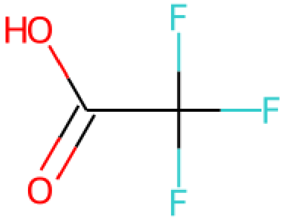
SM

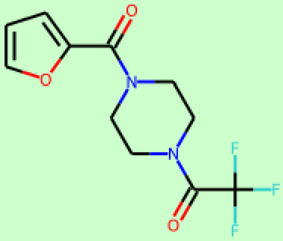
success

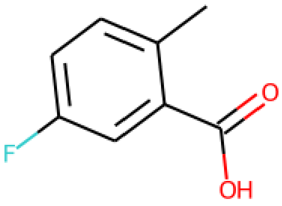
SM

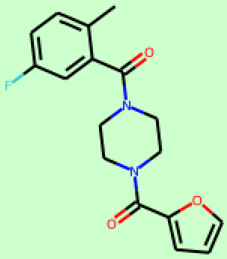
success

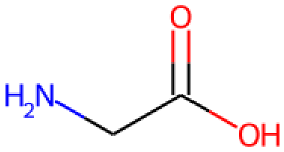
SM

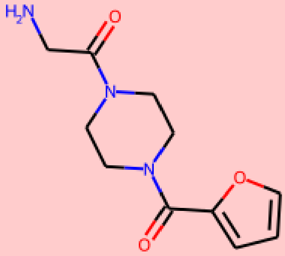
fail

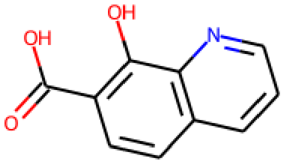
SM

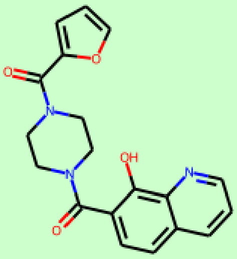
success

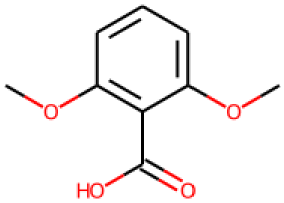
SM

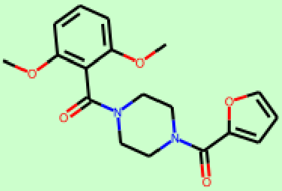
success

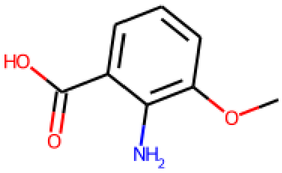
SM

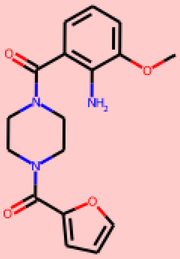
fail

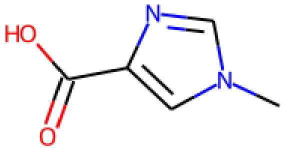
SM

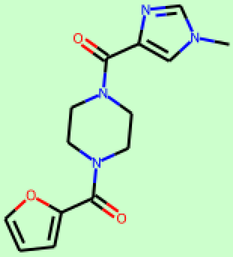
success

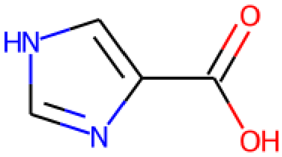
SM

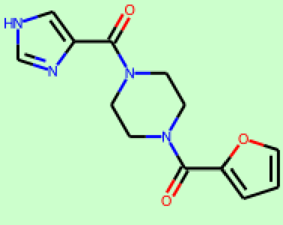
success

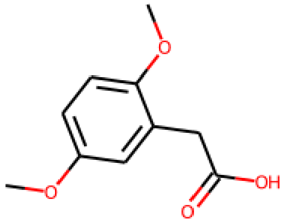
SM

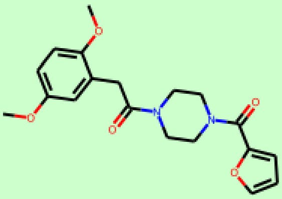
success

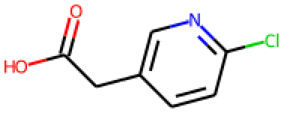
SM

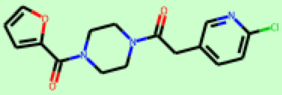
success

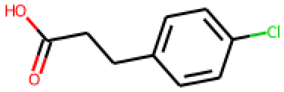
SM

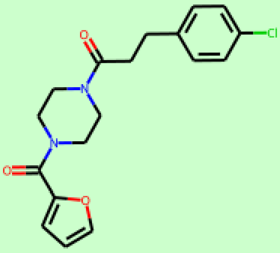
success

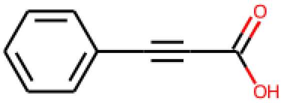
SM

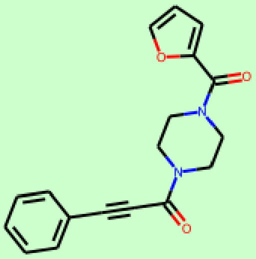
success

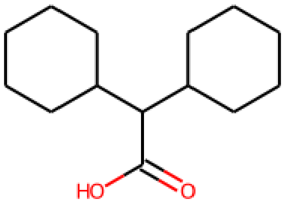

SM

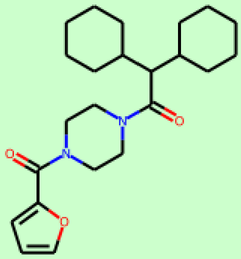

success

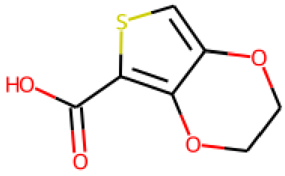

SM

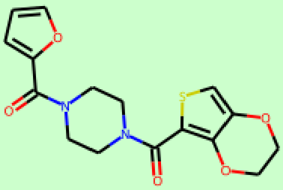

success

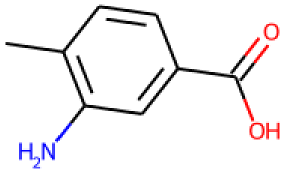

SM

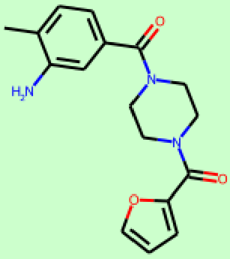

success

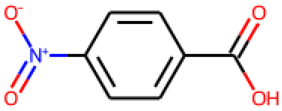

SM

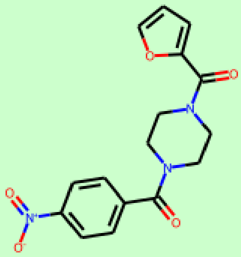

success

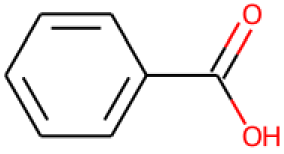

SM

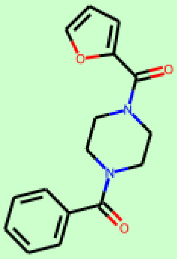

success

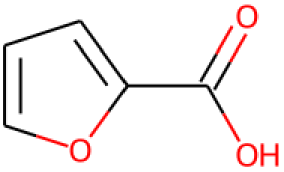

SM

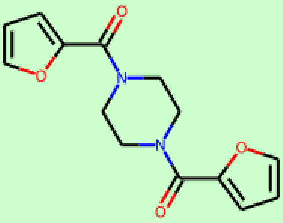

success

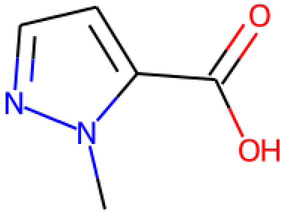

SM

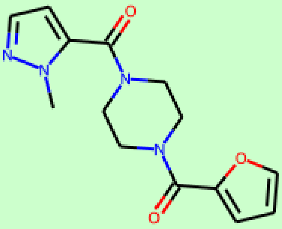

success

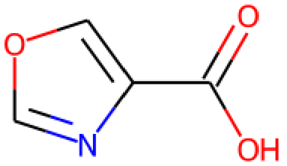

SM

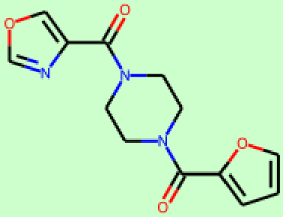

success

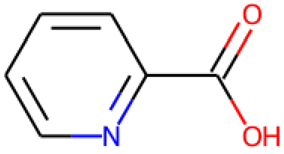

SM

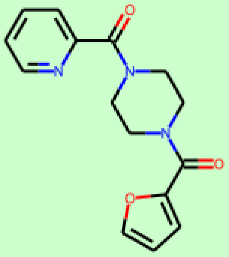

success

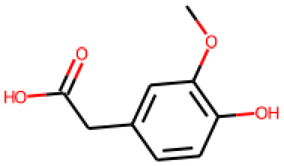

SM

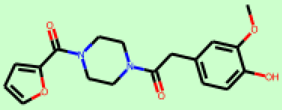

success

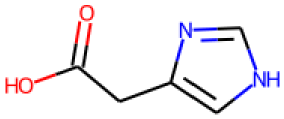

SM

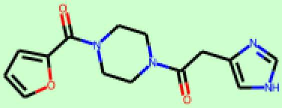

success

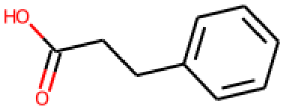

SM

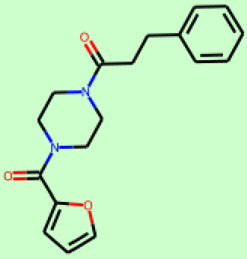

success

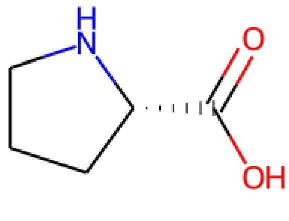

SM

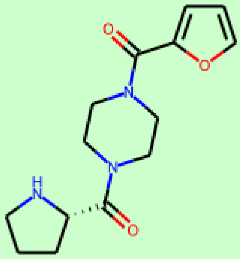

success

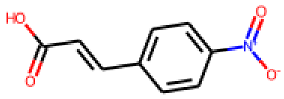

SM

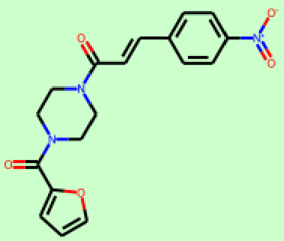

success

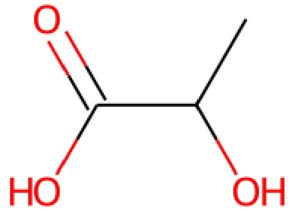

SM

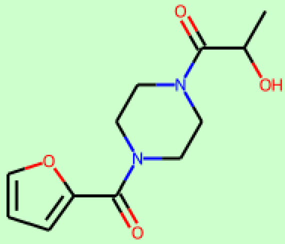

success

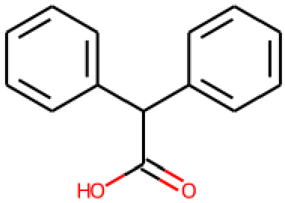

SM

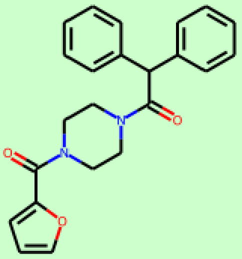

success

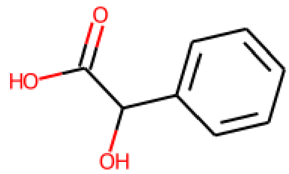

SM

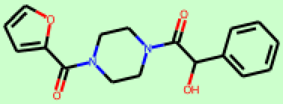

success

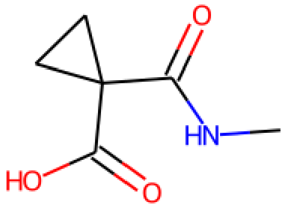

SM

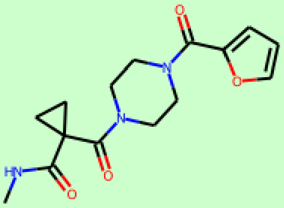

success

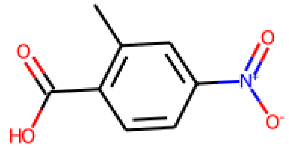

SM

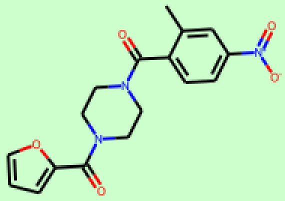

success

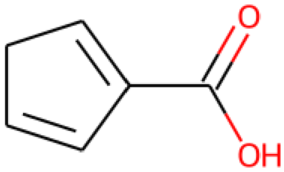

SM

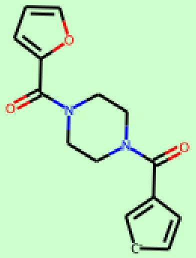

success

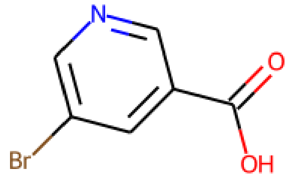

SM

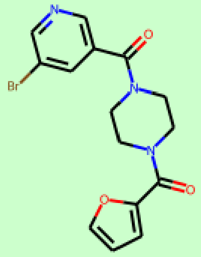

success

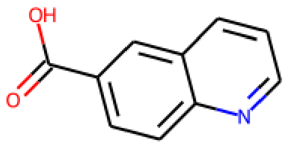

SM

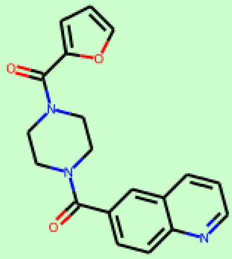

success

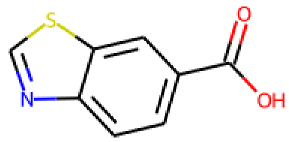

SM

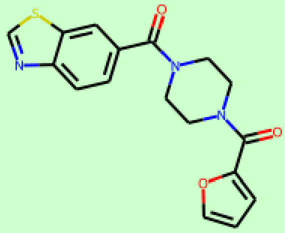

success

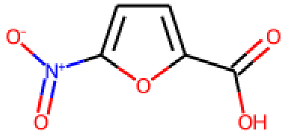

SM

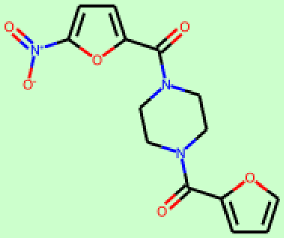

success

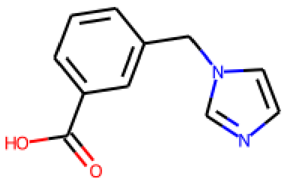

SM

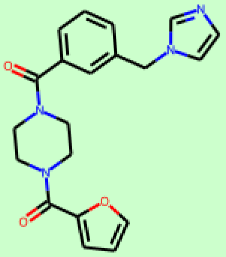

success

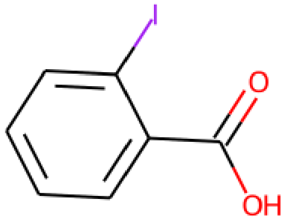

SM

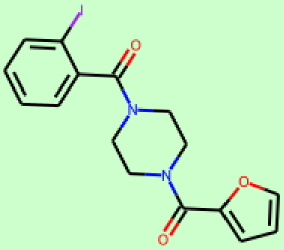

success

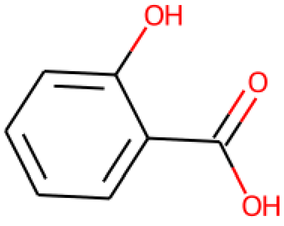

SM

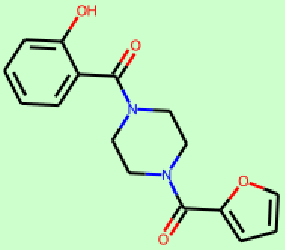

success

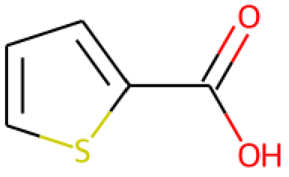

SM

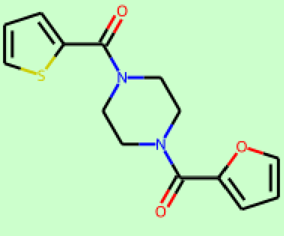

success

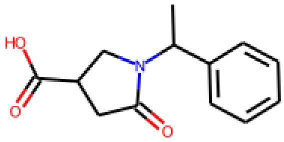

SM

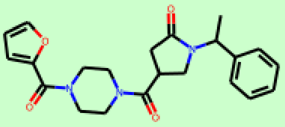

success

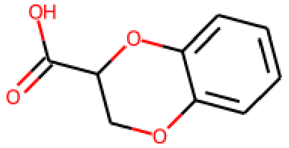

SM

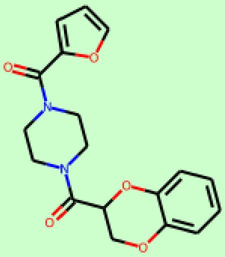

success

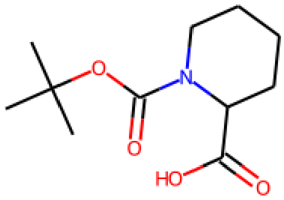

SM

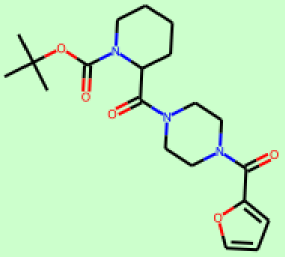

success

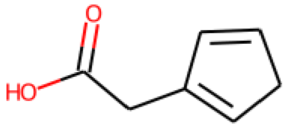

SM

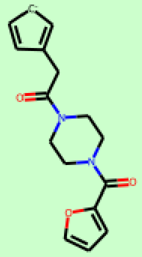

success

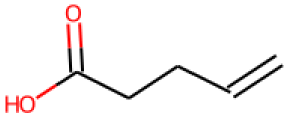

SM

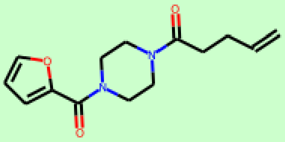

success

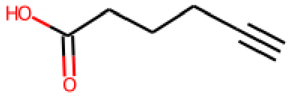

SM

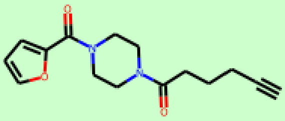

success

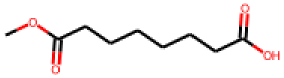

SM

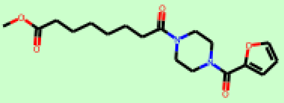

success

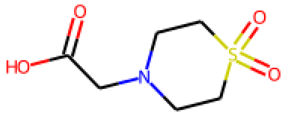

SM

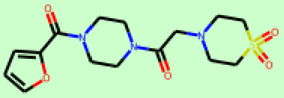

success

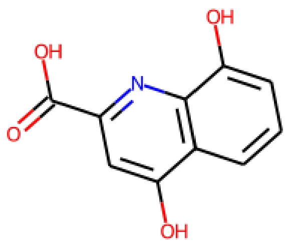

SM

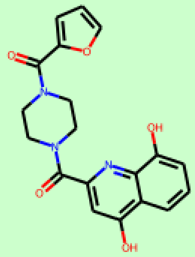

success

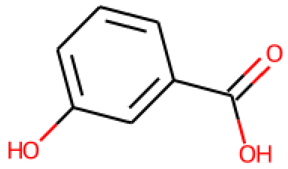

SM

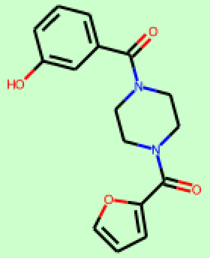

success

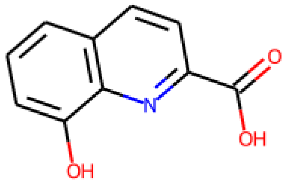

SM

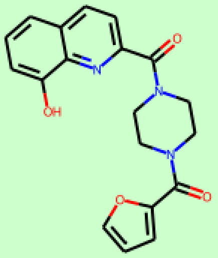

success

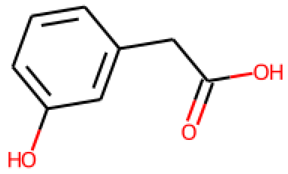

SM

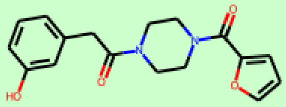

success

8.4 **Iteration 3.0**

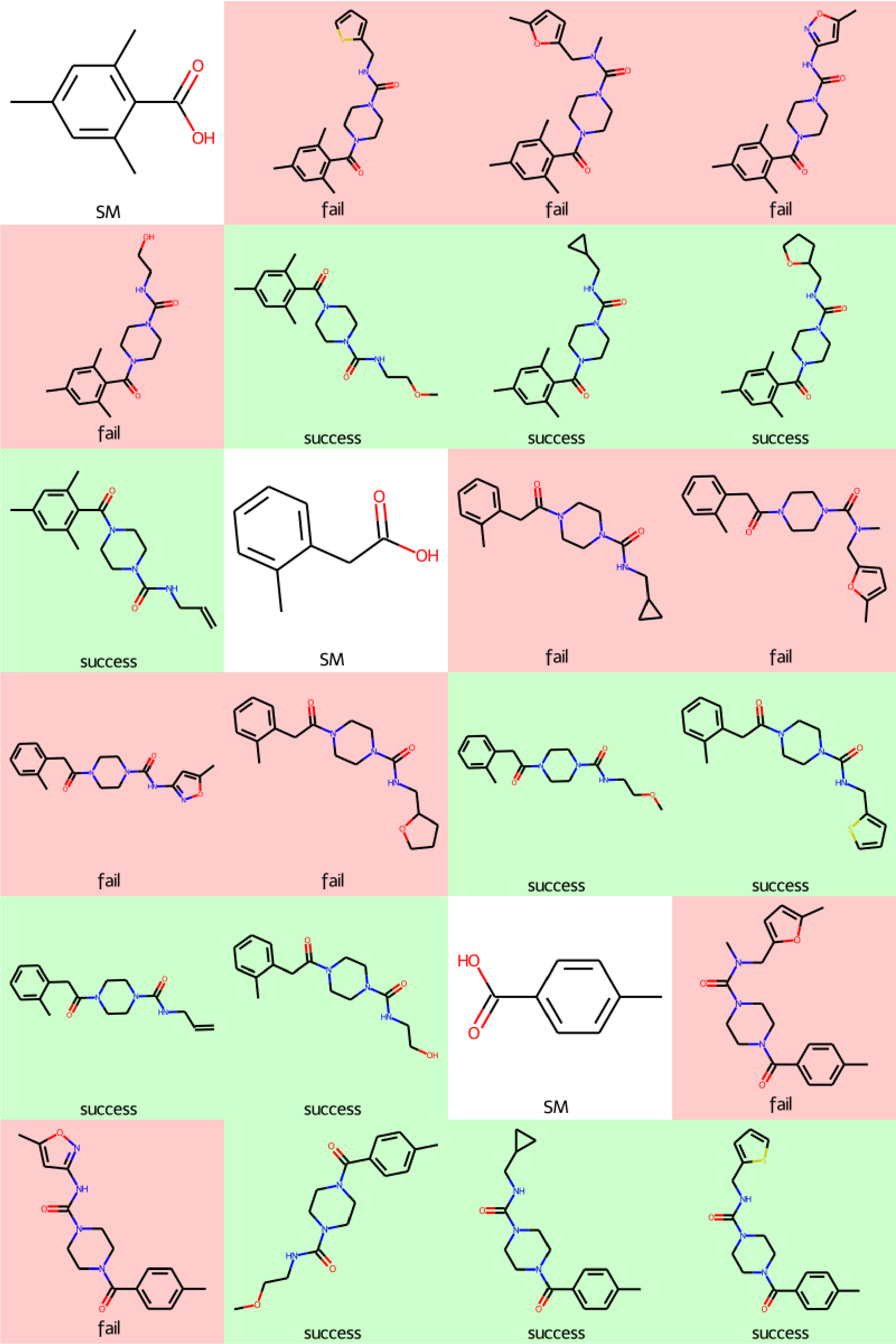
SM
fail
fail
fail
fail
success
success
success
success
SM
fail
fail
fail
fail
success
success
success
success
SM
fail
fail
success
success
success

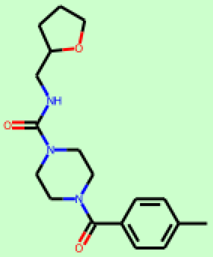
success
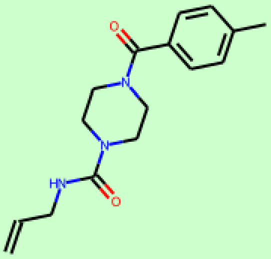
success
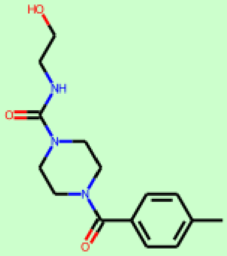
success
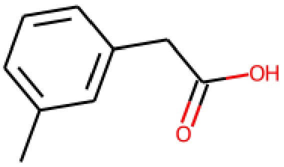
SM

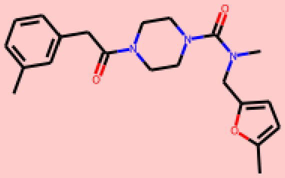
fail
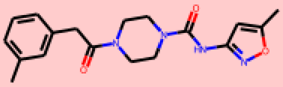
fail
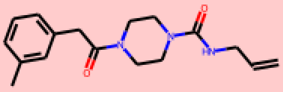
fail
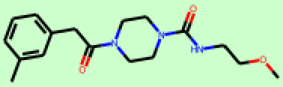
success

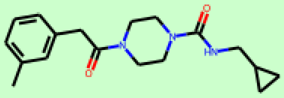
success
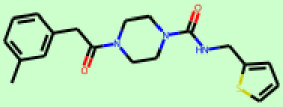
success
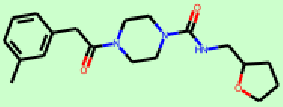
success
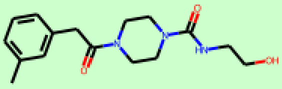
success

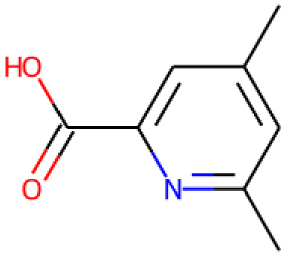
SM
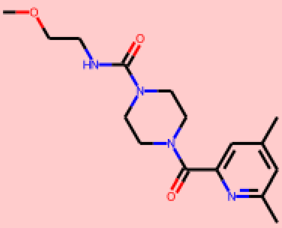
fail
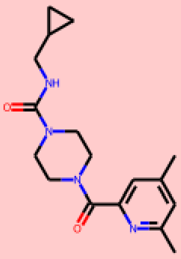
fail
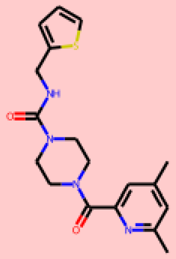
fail

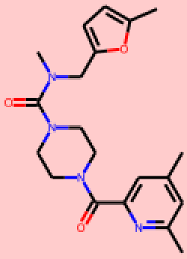
fail
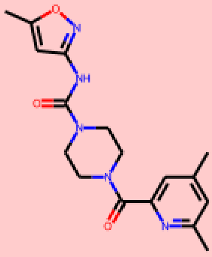
fail
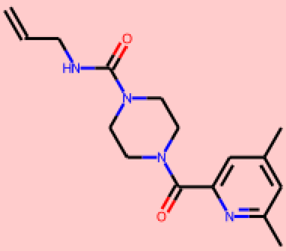
fail
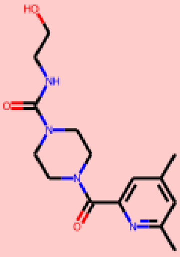
fail

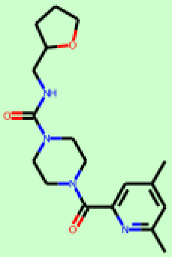
success
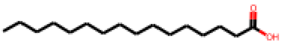
SM
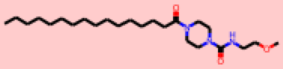
fail
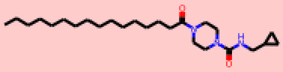
fail

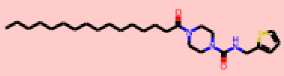
fail
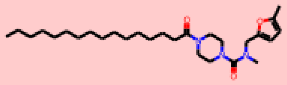
fail
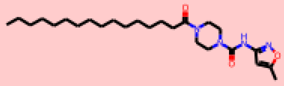
fail
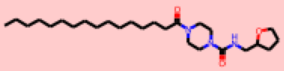
fail
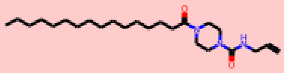
fail
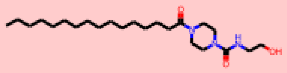
fail
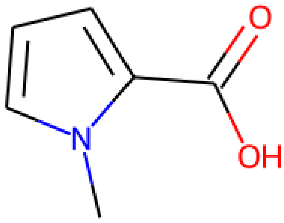
SM
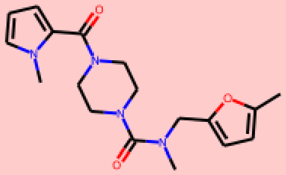
fail
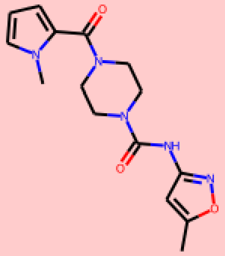
fail
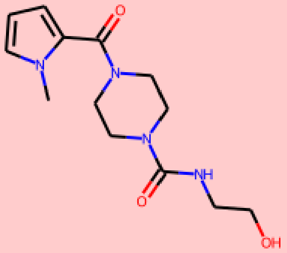
fail
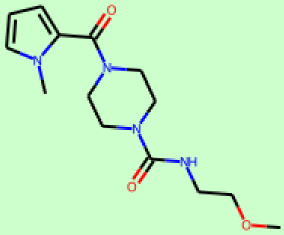
success
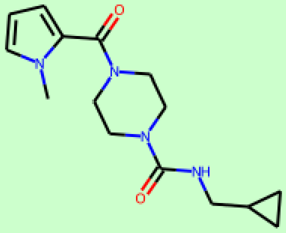
success
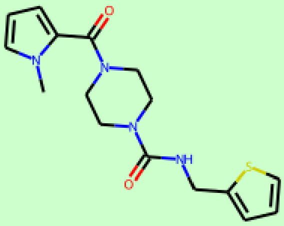
success
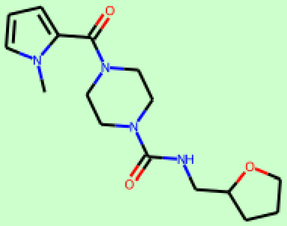
success
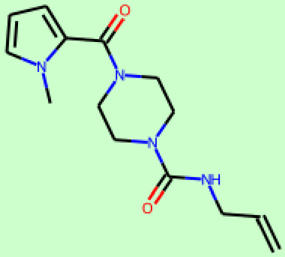
success
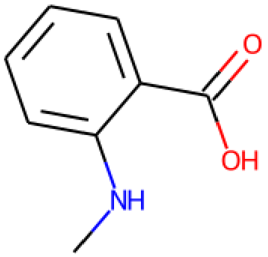
SM
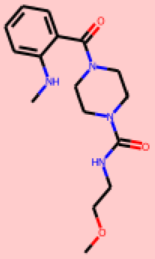
fail
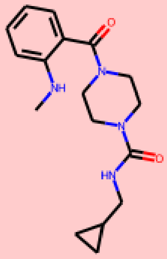
fail
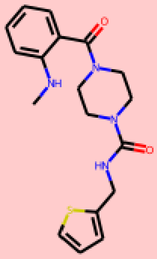
fail
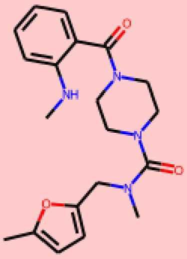
fail
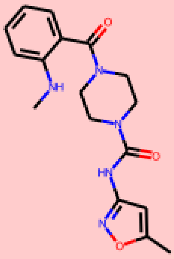
fail
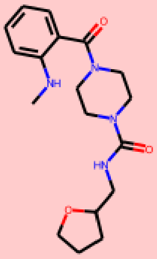
fail
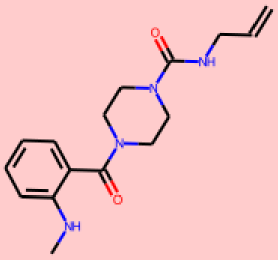
fail
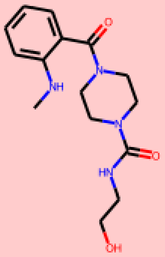
fail

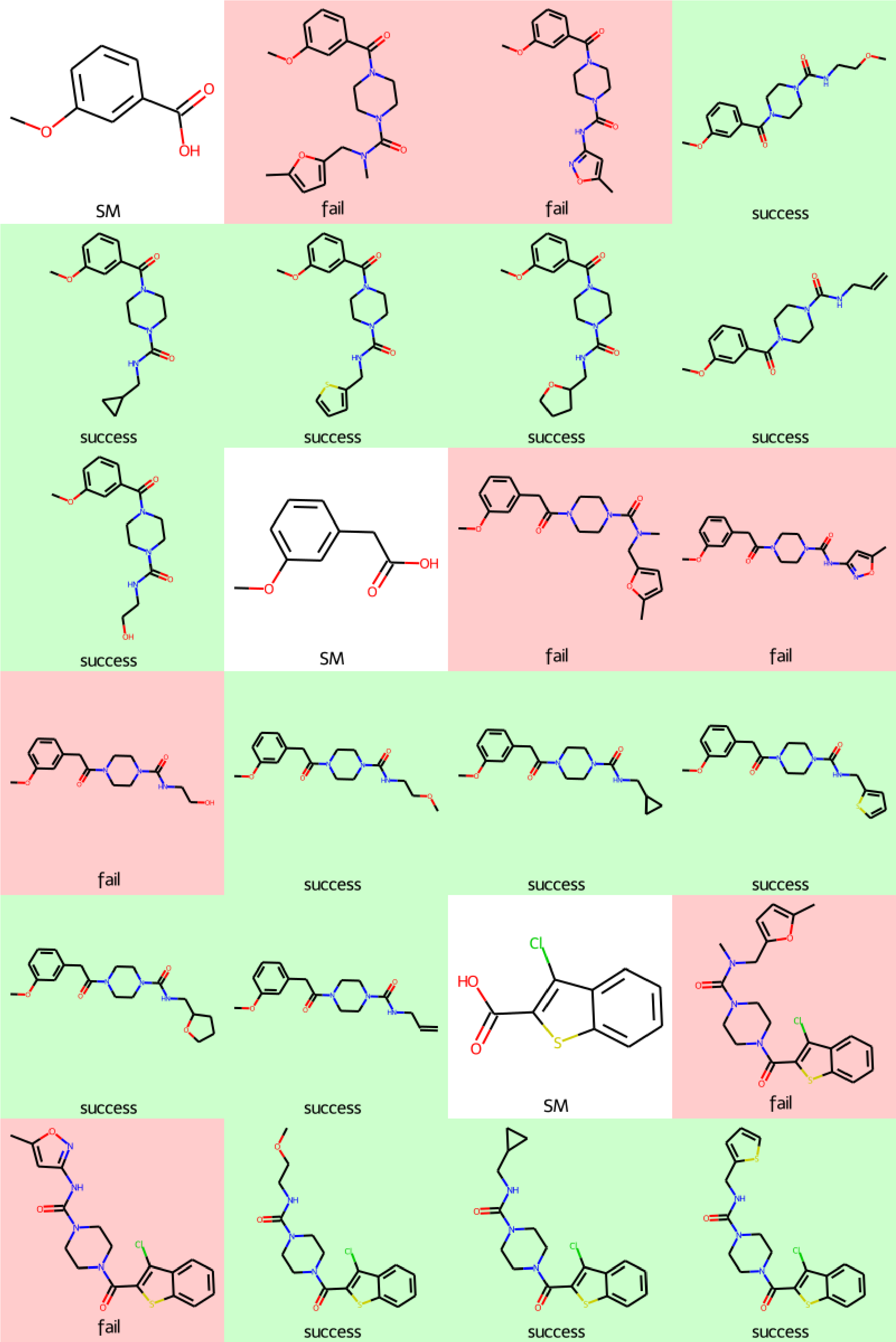
SM
fail
fail
success
success
success
success
success
success
SM
fail
fail
fail
success
success
success
success
success
SM
fail
fail
success
success
success

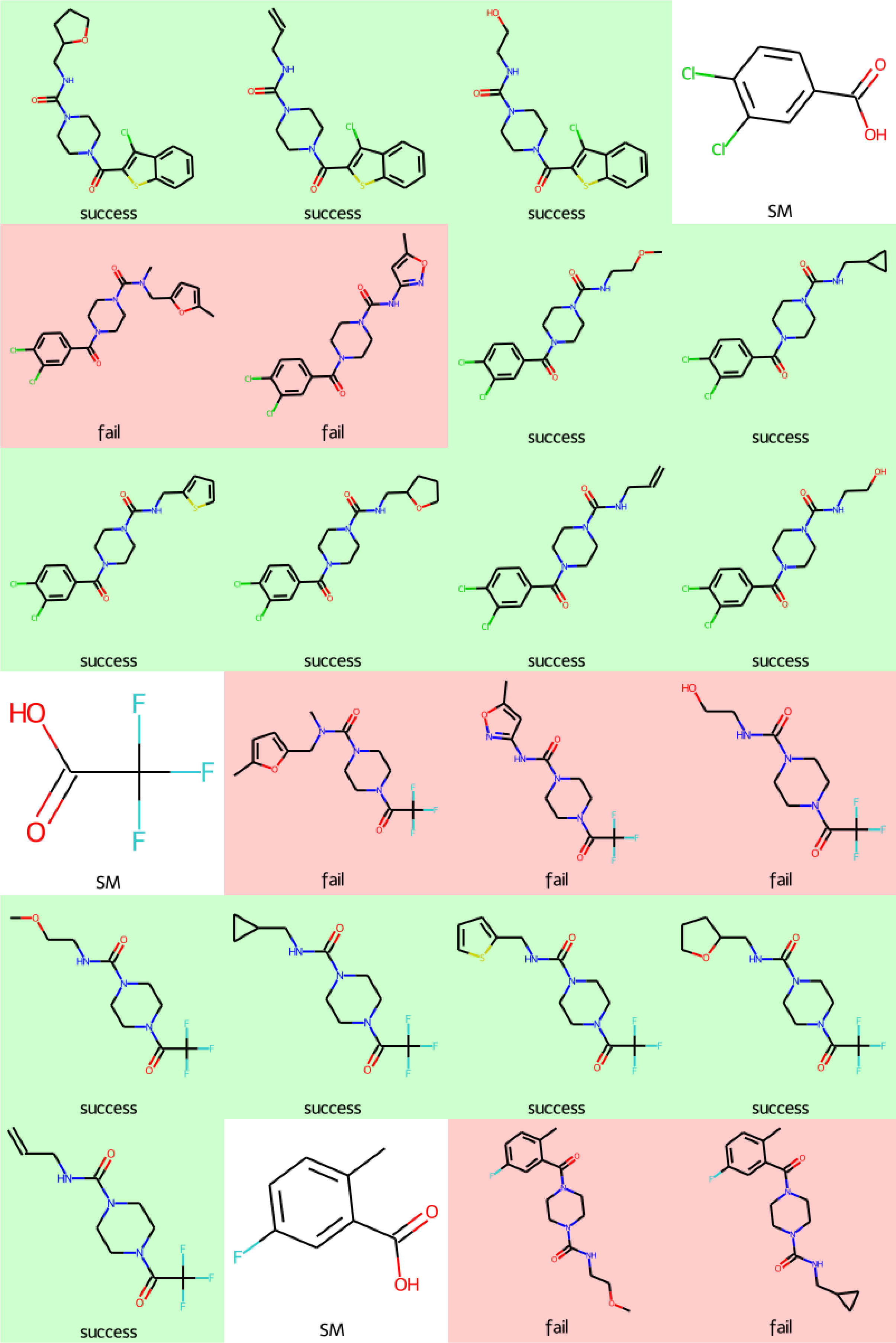
success
success
success
SM
fail
fail
success
success
success
success
success
success
SM
fail
fail
fail
success
success
success
success
success
SM
fail
fail

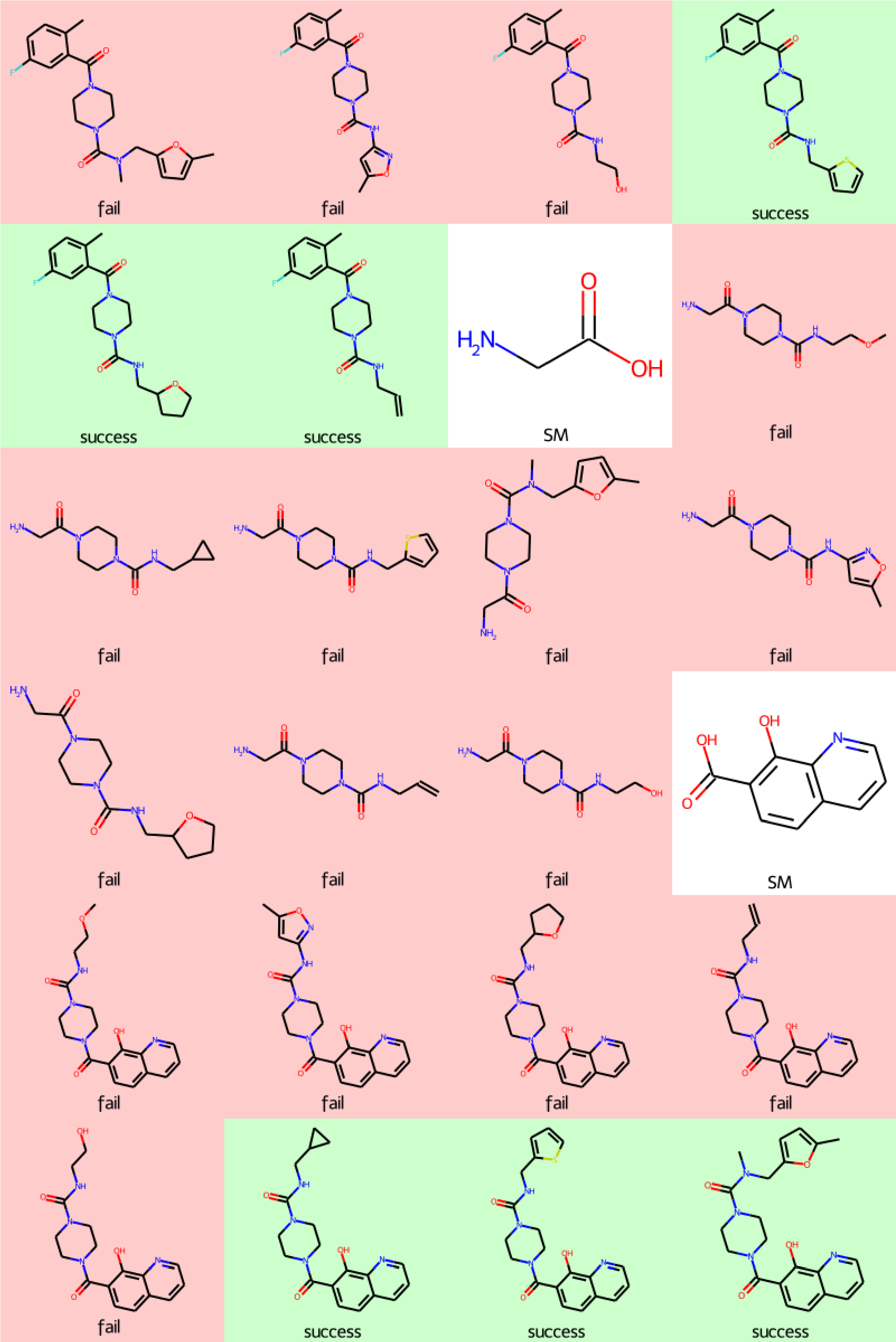
fail
fail
fail
success
success
success
SM
fail
fail
fail
fail
fail
fail
fail
fail
SM
fail
fail
fail
fail
fail
success
success
success

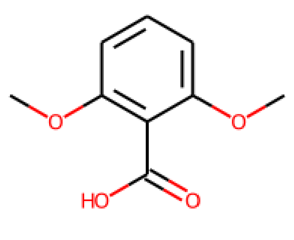
SM
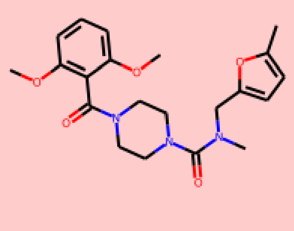
fail
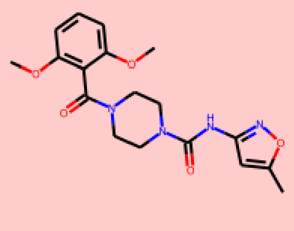
fail
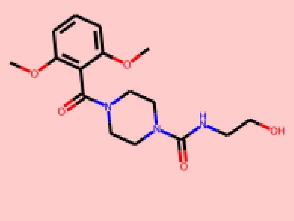
fail
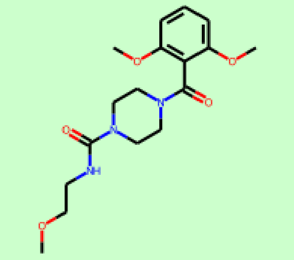
success
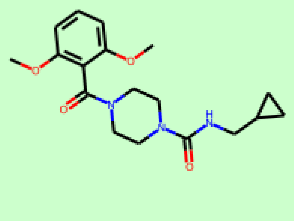
success
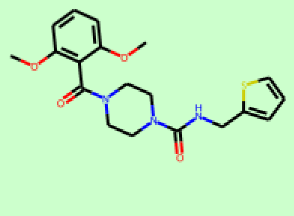
success
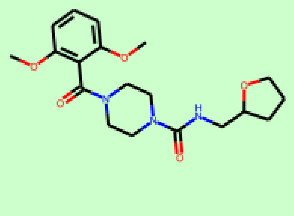
success
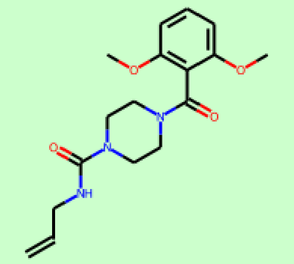
success
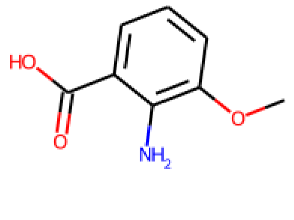
SM
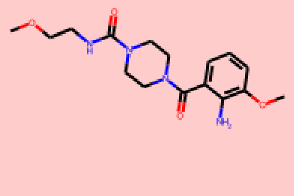
fail
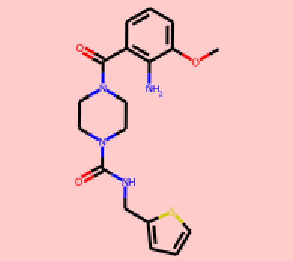
fail
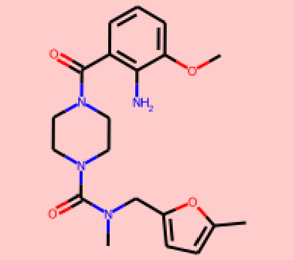
fail
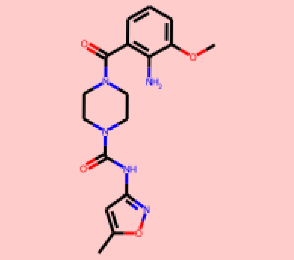
fail
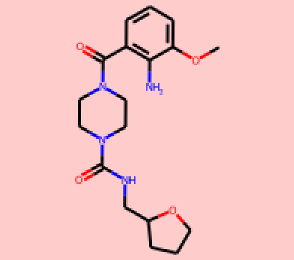
fail
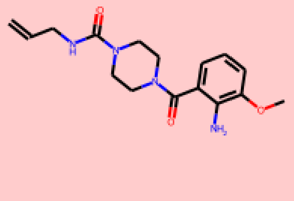
fail
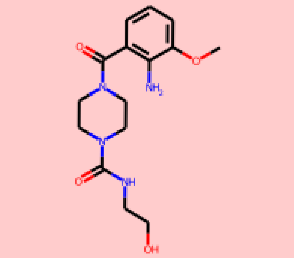
fail
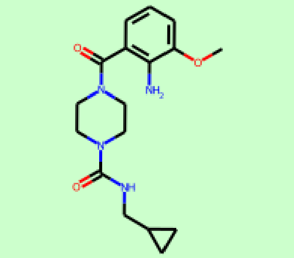
success
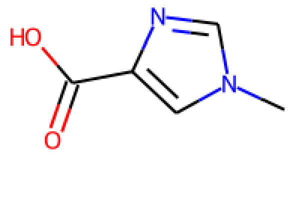
SM
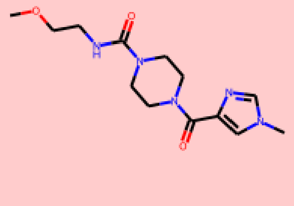
fail
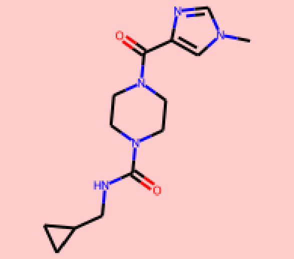
fail
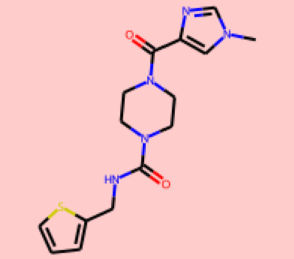
fail
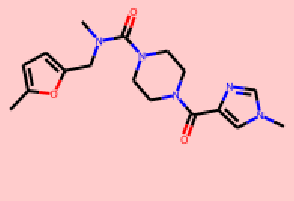
fail
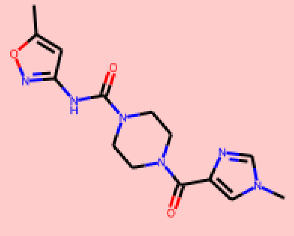
fail

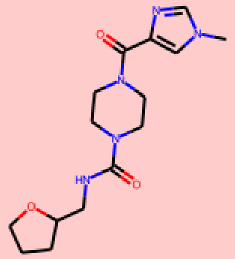

fail

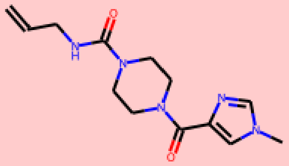

fail

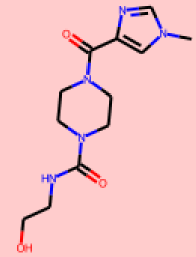

fail

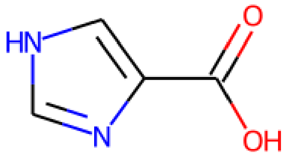

SM

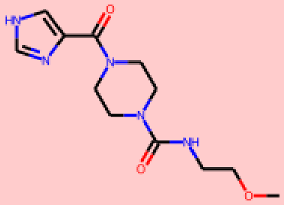

fail

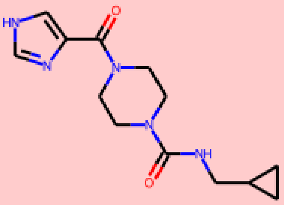

fail

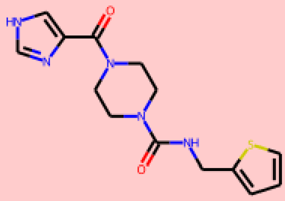

fail

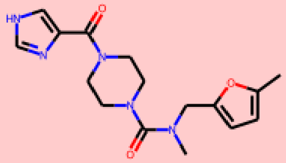

fail

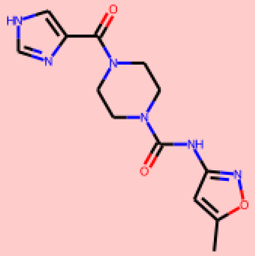

fail

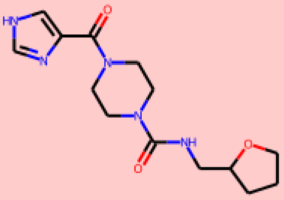

fail

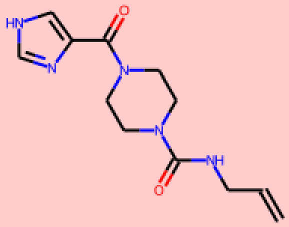

fail

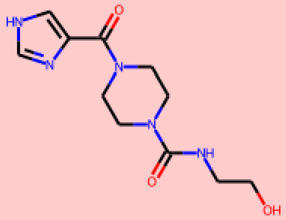

fail

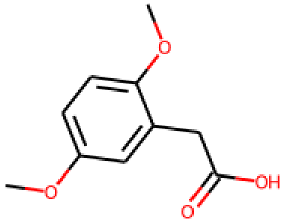

SM

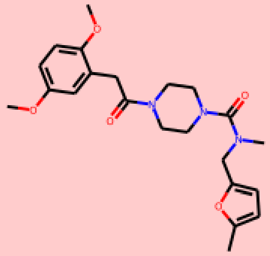

fail

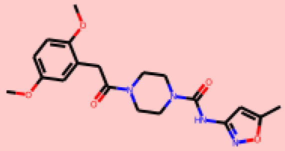

fail

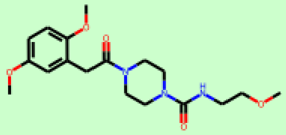

success

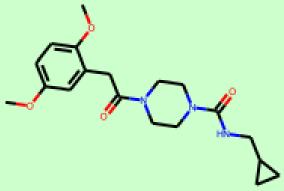

success

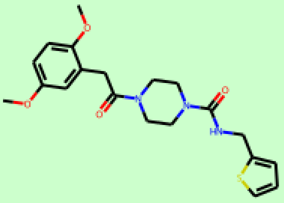

success

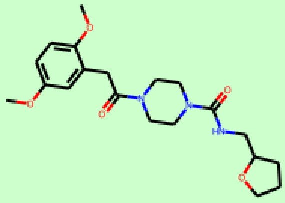

success

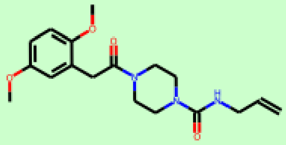

success

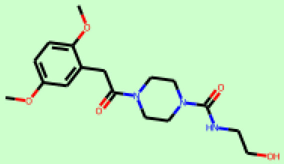

success

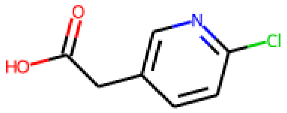

SM

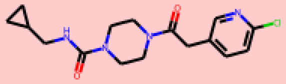

fail

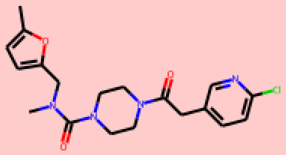

fail

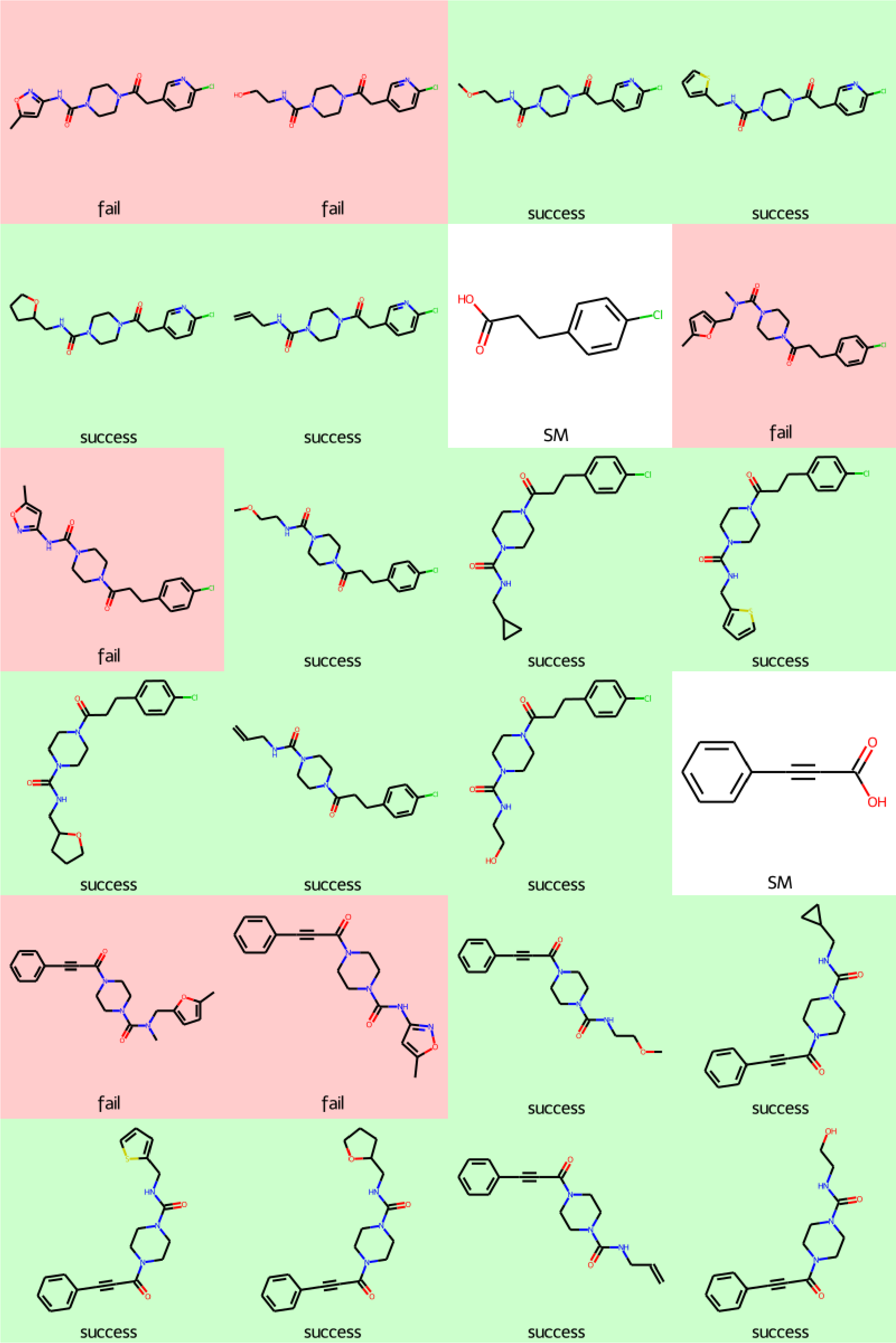
fail
fail
success
success
success
success
SM
fail
fail
success
success
success
success
success
success
SM
fail
fail
success
success
success
success
success
success

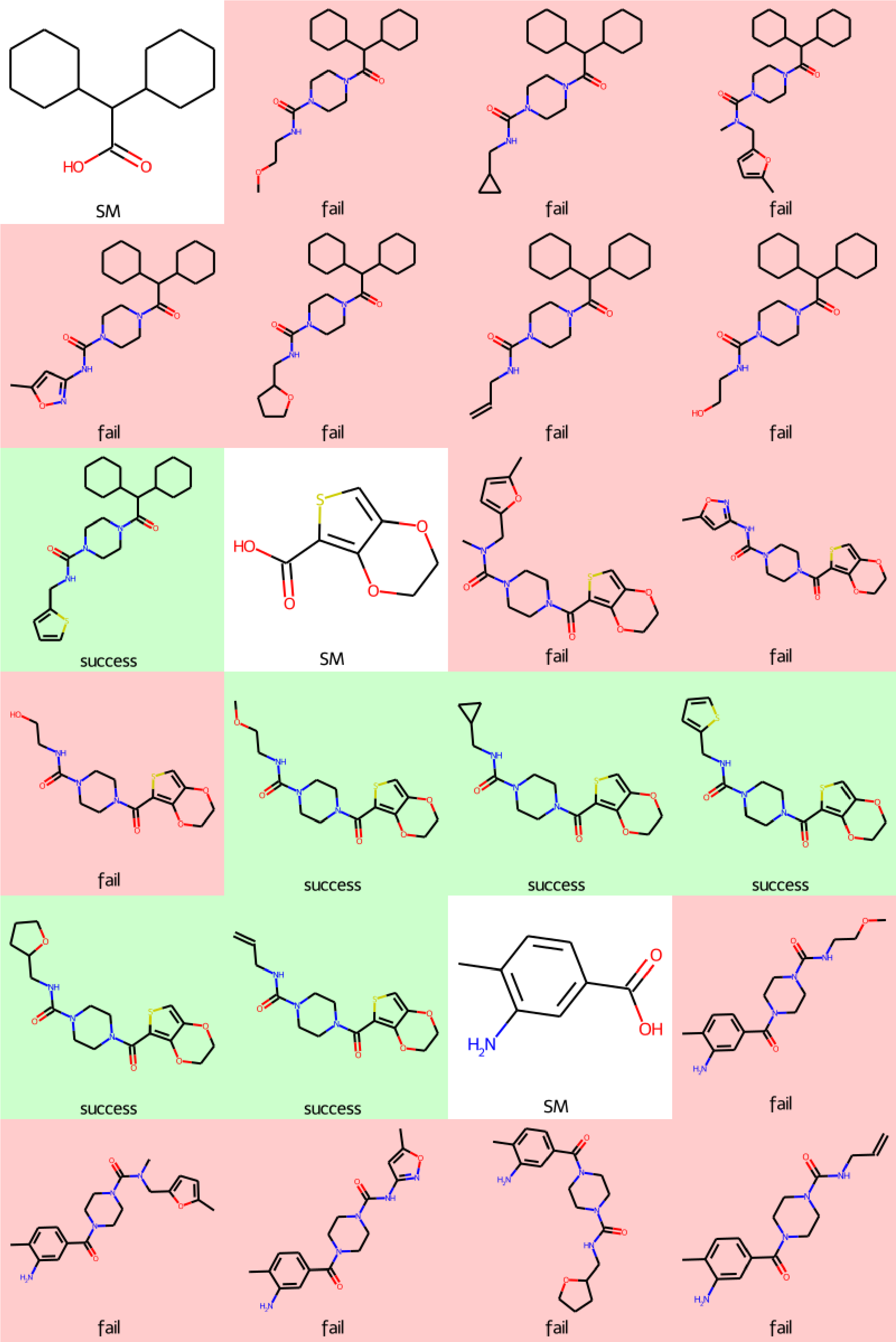
SM
fail
fail
fail
fail
fail
fail
fail
success
SM
fail
fail
fail
success
success
success
success
success
SM
fail
fail
fail
fail
fail

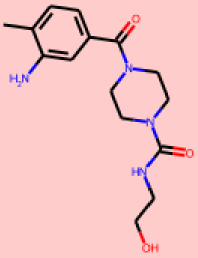

fail

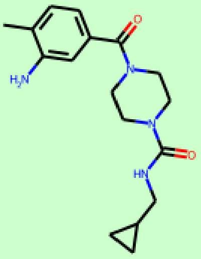

success

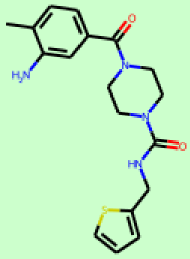

success

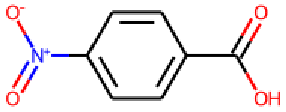

SM

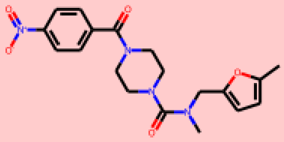

fail

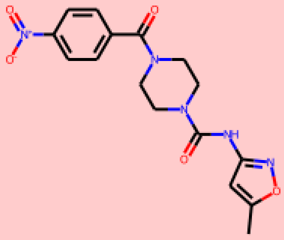

fail

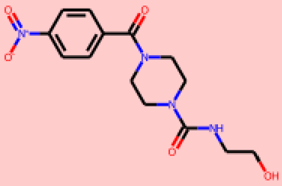

fail

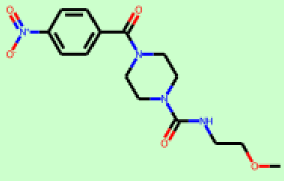

success

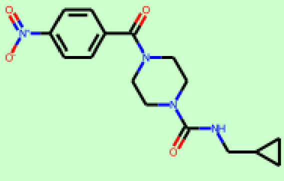

success

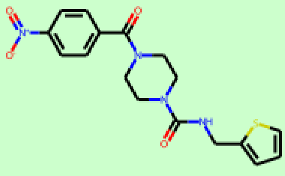

success

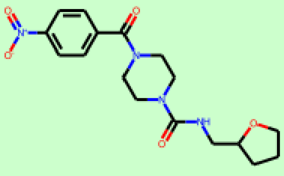

success

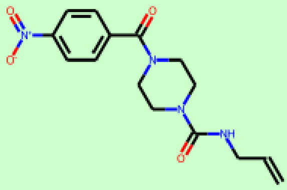

success

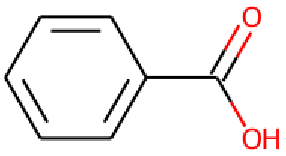

SM

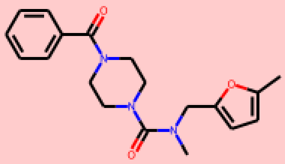

fail

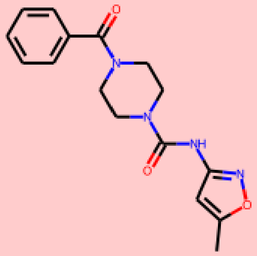

fail

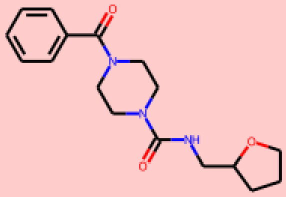

fail

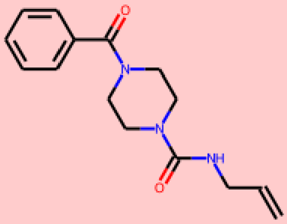

fail

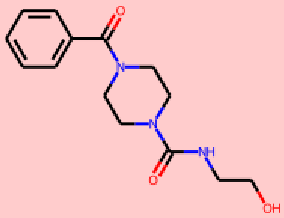

fail

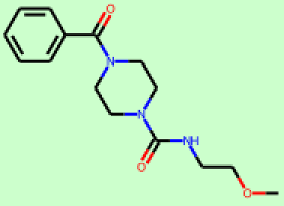

success

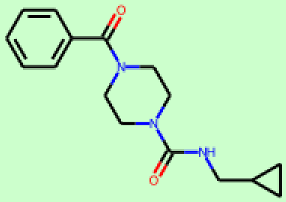

success

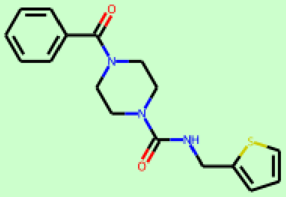

success

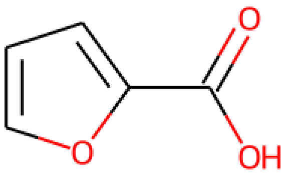

SM

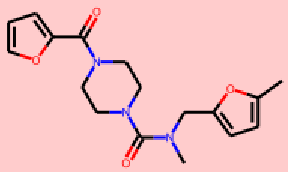

fail

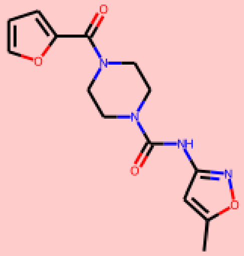

fail

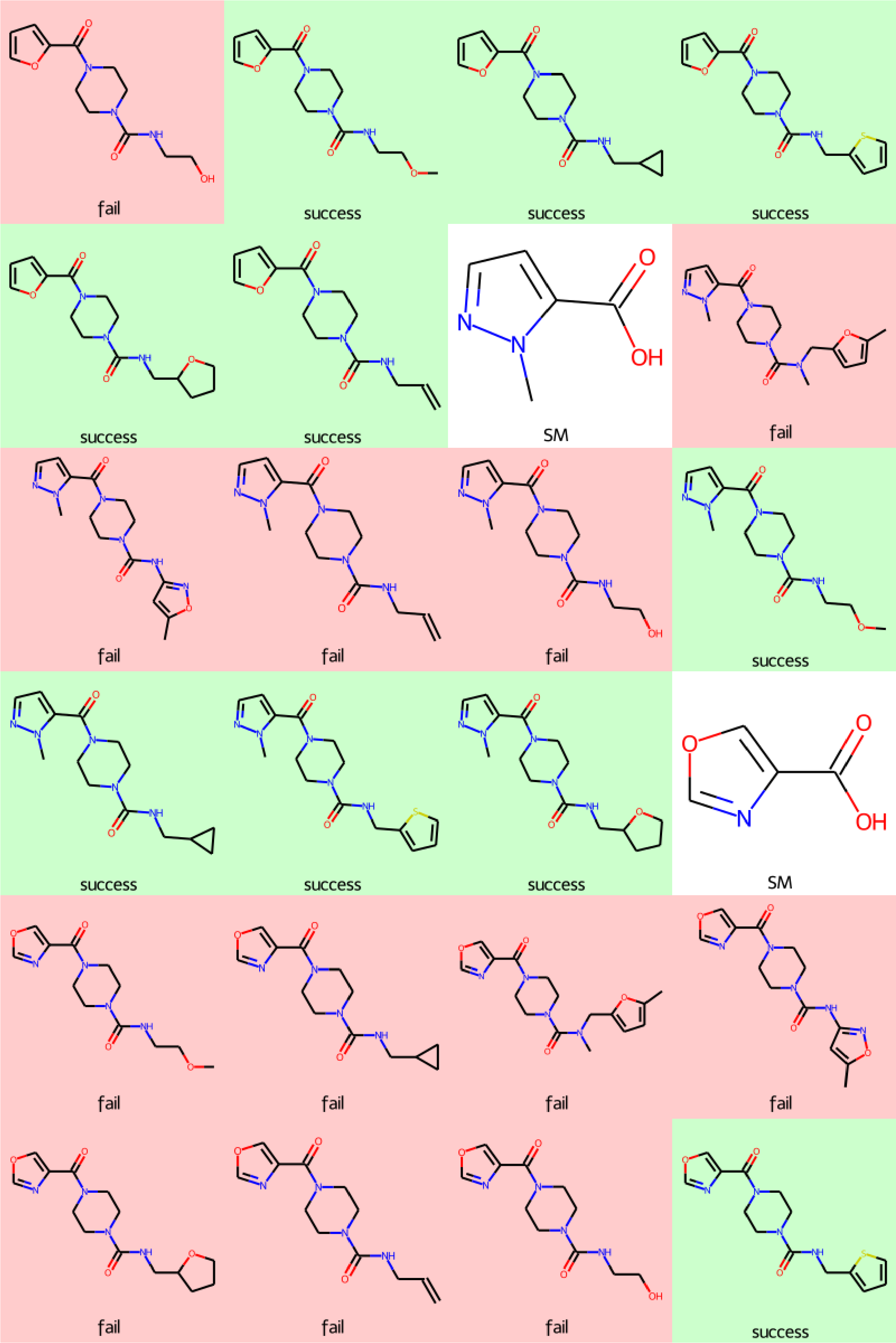
fail
success
success
success
success
success
SM
fail
fail
fail
fail
success
success
success
success
SM
fail
fail
fail
fail
fail
fail
fail
success

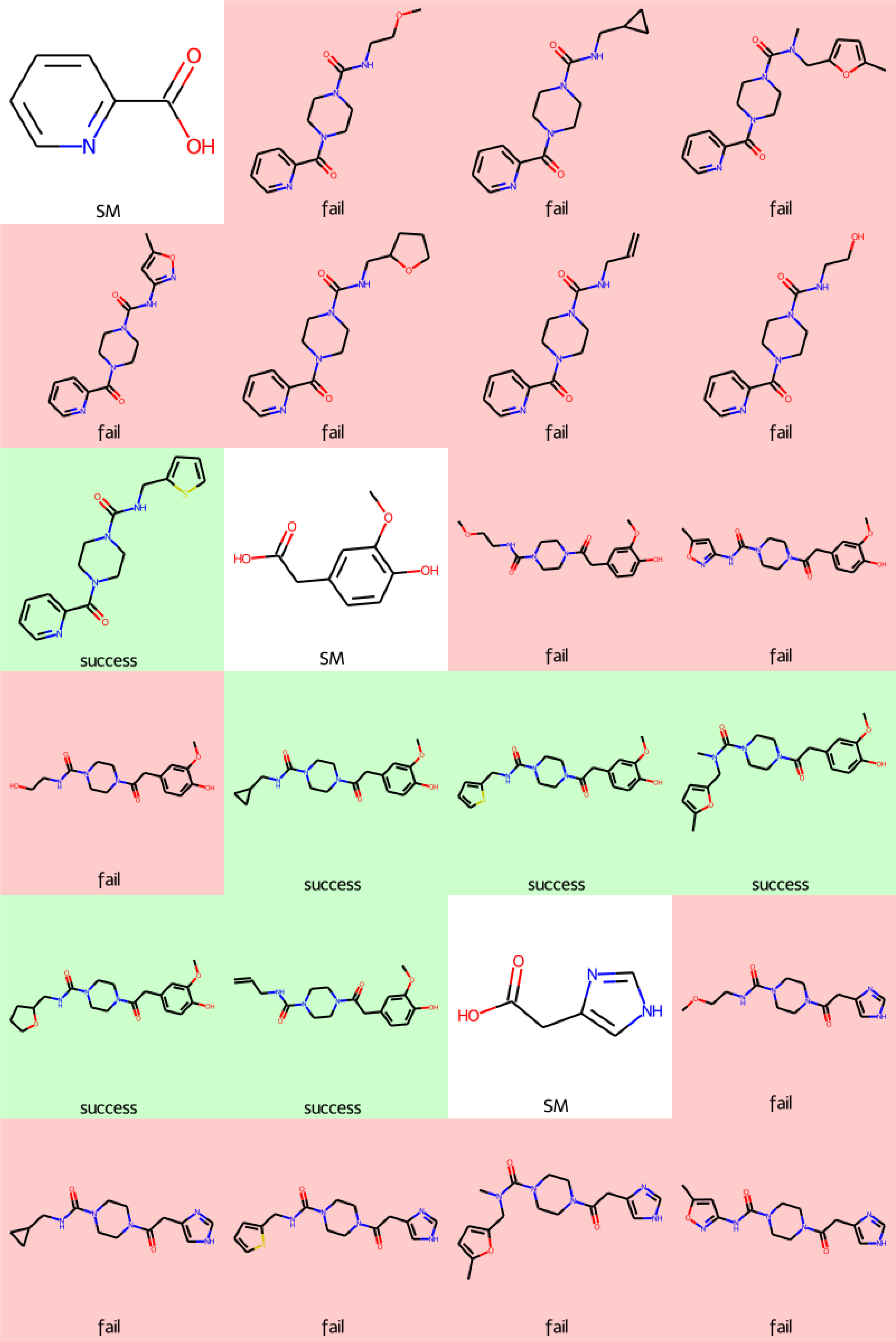
SM
fail
fail
fail
fail
fail
fail
fail
success
SM
fail
fail
fail
success
success
success
success
success
SM
fail
fail
fail
fail
fail

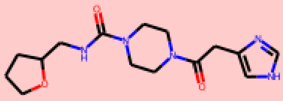
fail
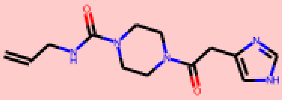
fail
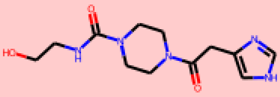
fail
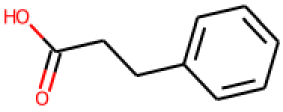
SM
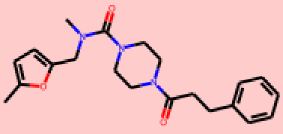
fail
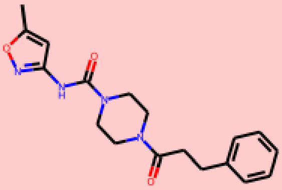
fail
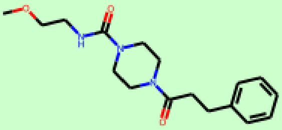
success
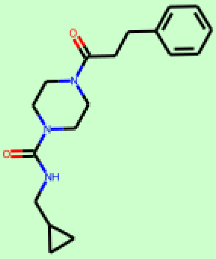
success
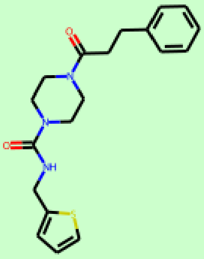
success
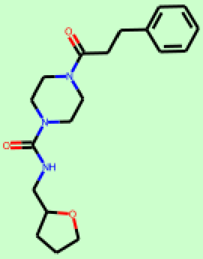
success
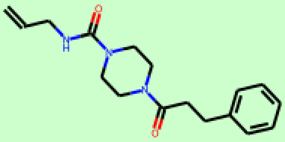
success
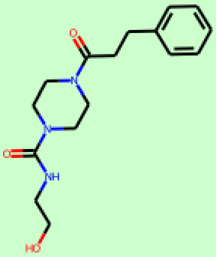
success
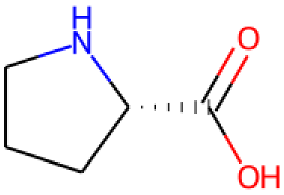
SM
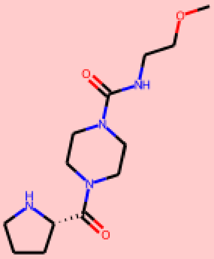
fail
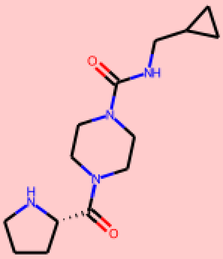
fail
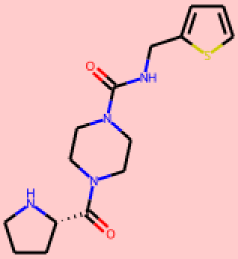
fail
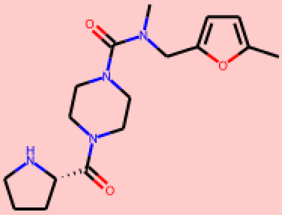
fail
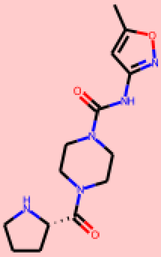
fail
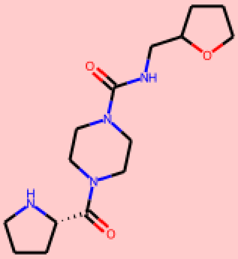
fail
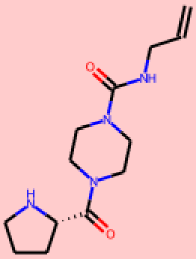
fail
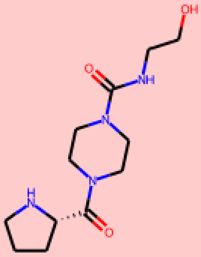
fail
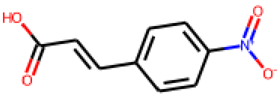
SM
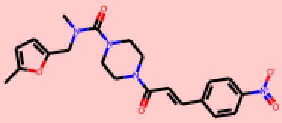
fail
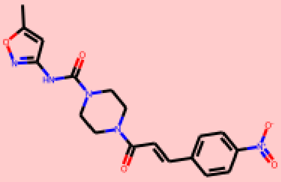
fail

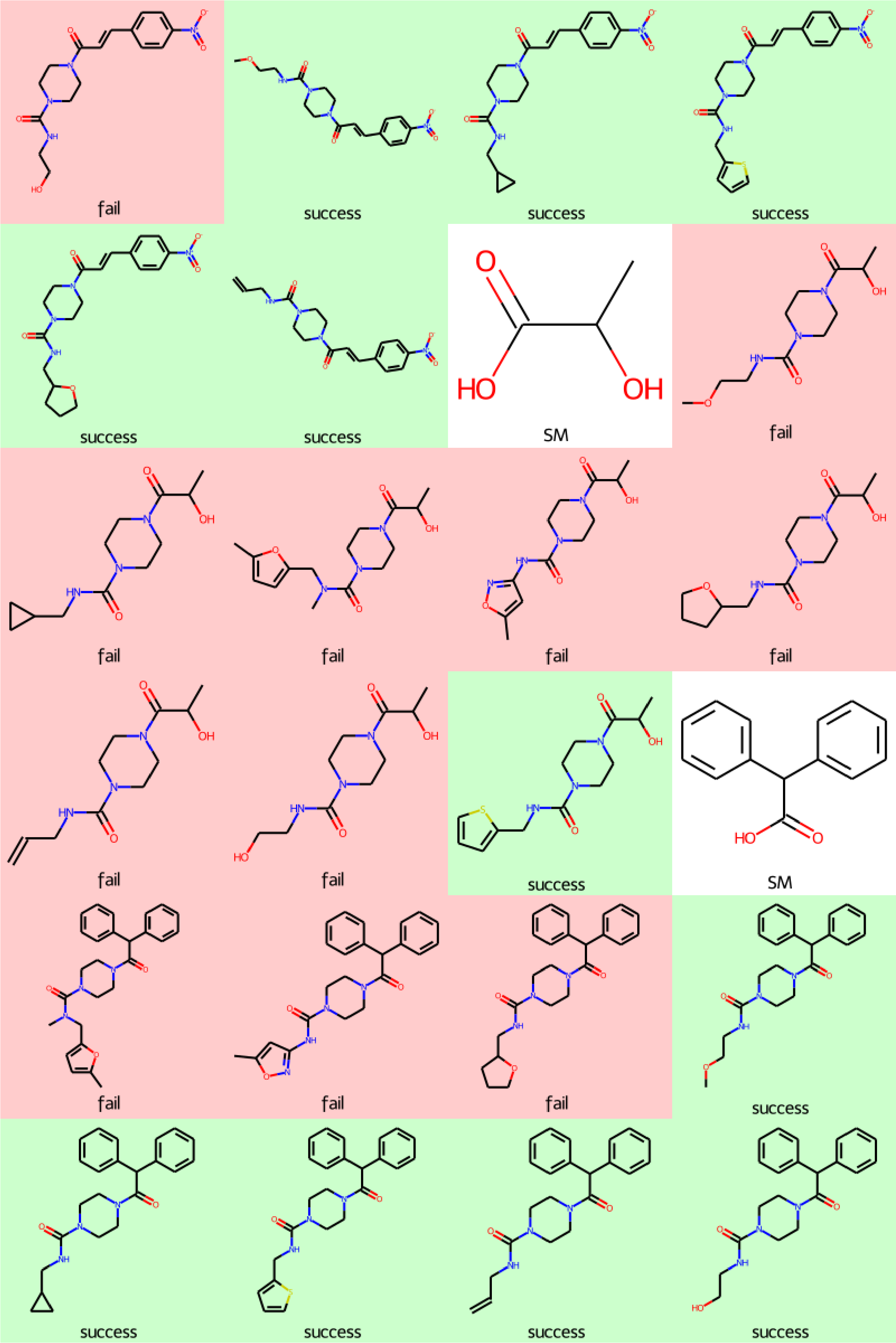

fail
success
success
success
success
success
SM
fail
fail
fail
fail
fail
fail
fail
success
SM
fail
fail
fail
success
success
success
success
success

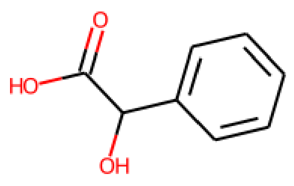

SM

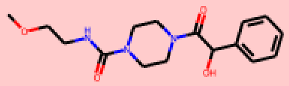

fail

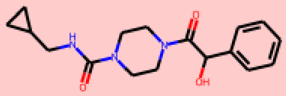

fail

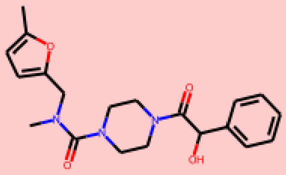

fail

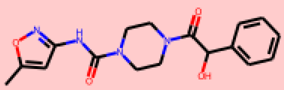

fail

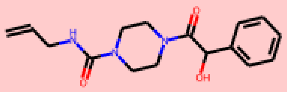

fail

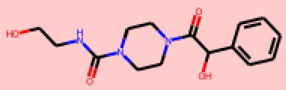

fail

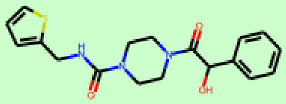

success

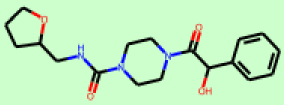

success

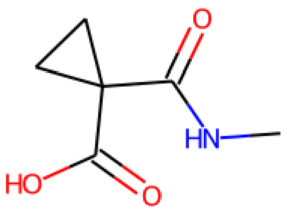

SM

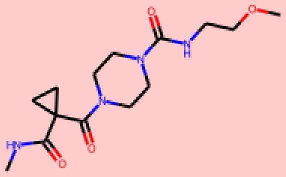

fail

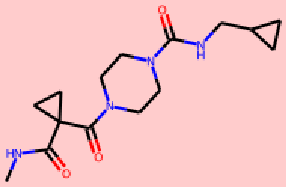

fail

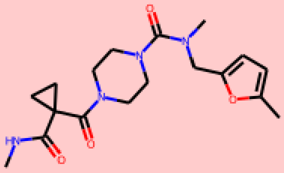

fail

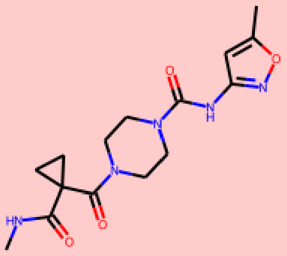

fail

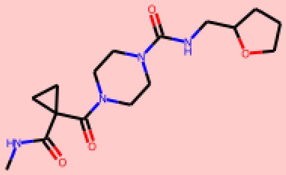

fail

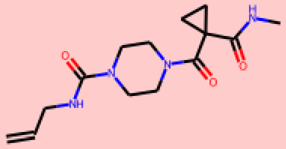

fail

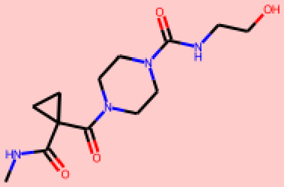

fail

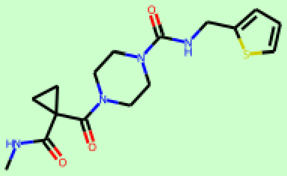

success

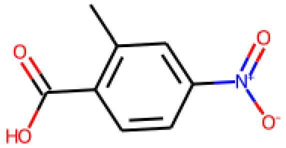

SM

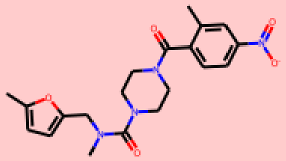

fail

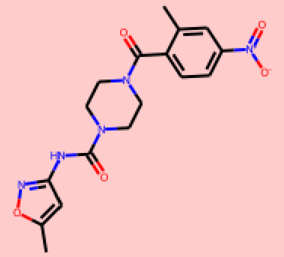

fail

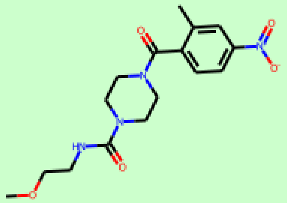

success

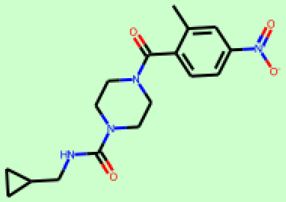

success

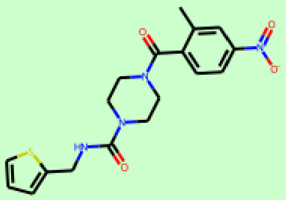

success

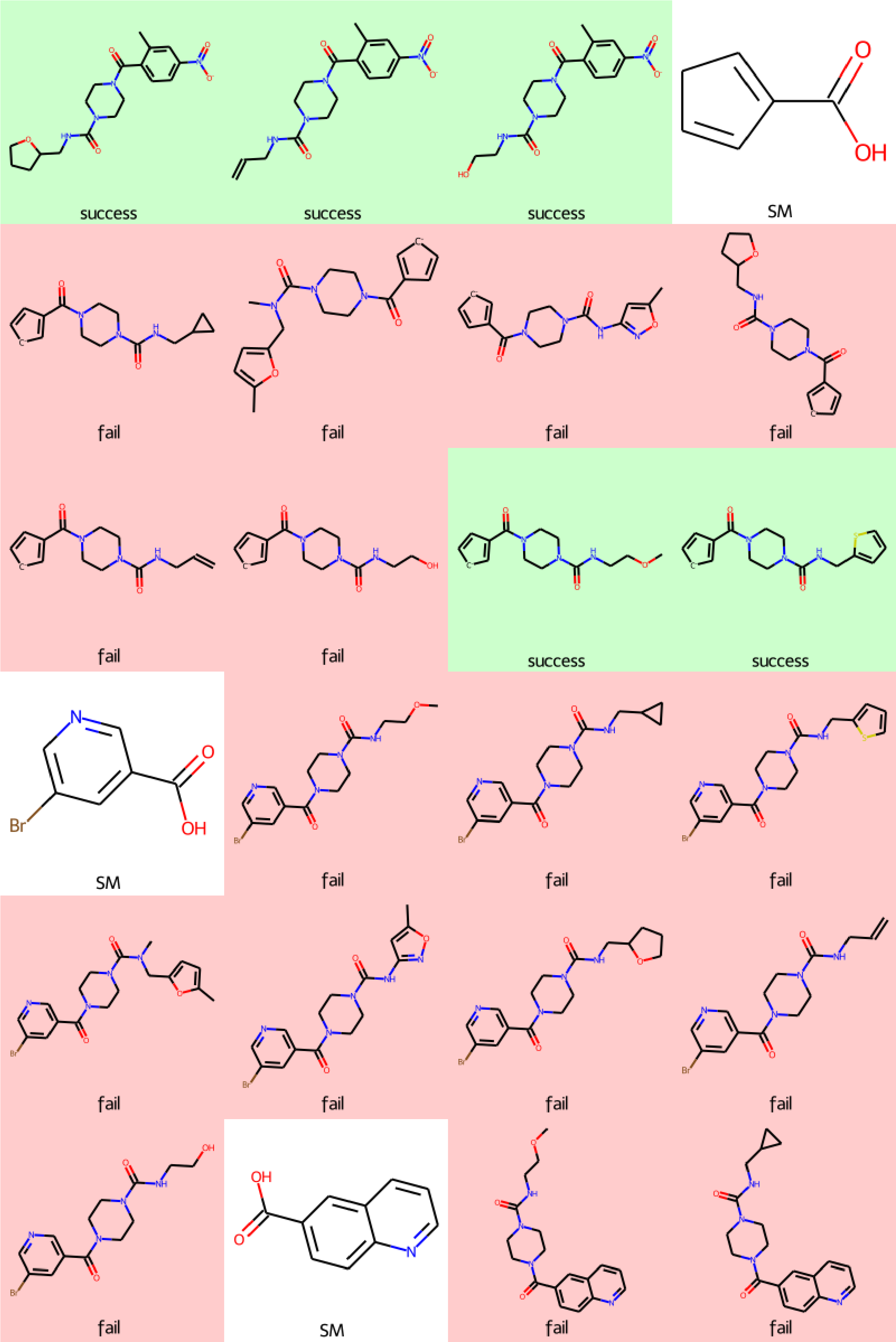
success
success
success
SM
fail
fail
fail
fail
fail
fail
success
success
SM
fail
fail
fail
fail
fail
fail
fail
fail
SM
fail
fail

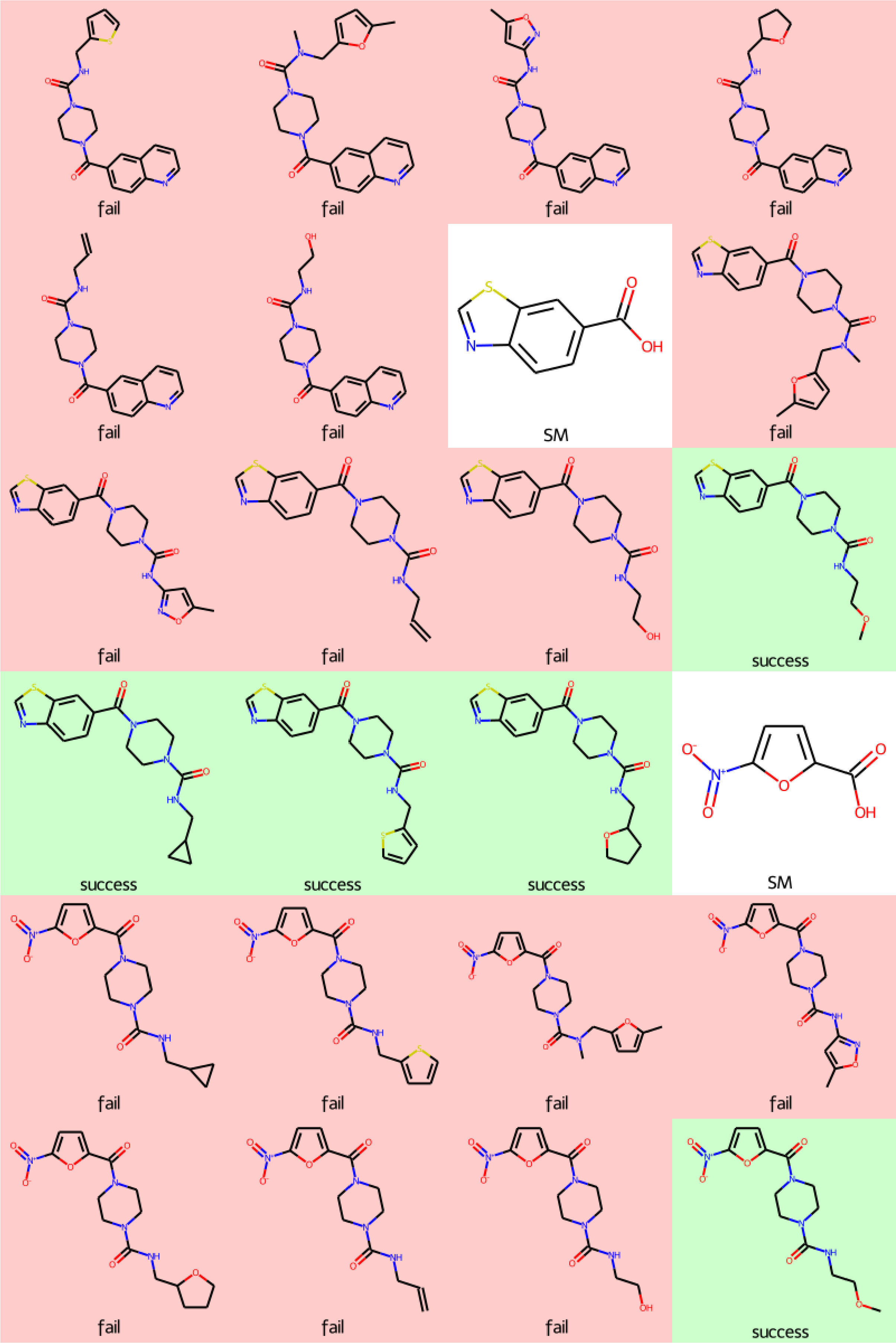
fail
fail
fail
fail
fail
fail
SM
fail
fail
fail
fail
success
success
success
success
SM
fail
fail
fail
fail
fail
fail
fail
success

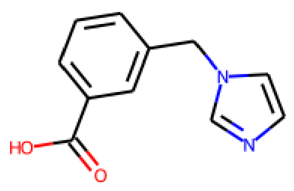
SM

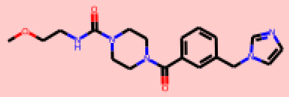
fail

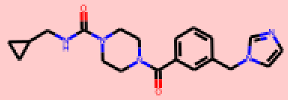
fail

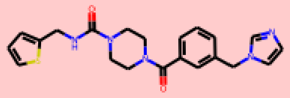
fail

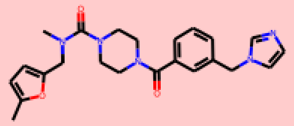
fail

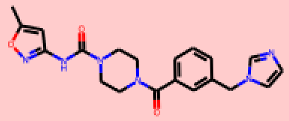
fail

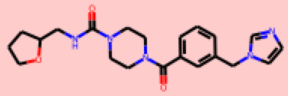
fail

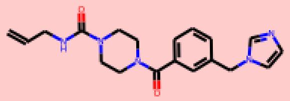
fail

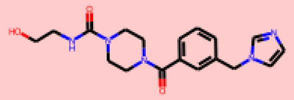
fail

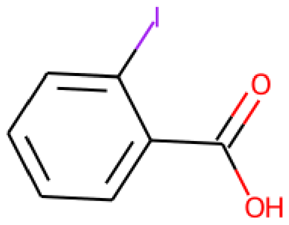
SM

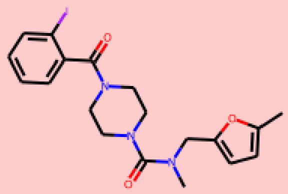
fail

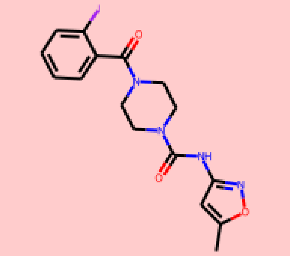
fail

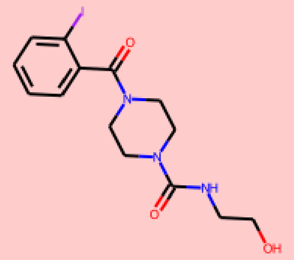
fail

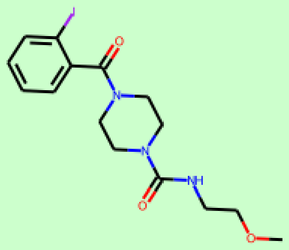
success

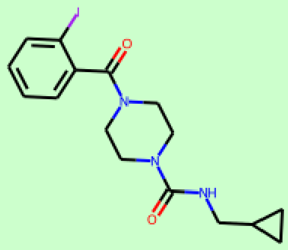
success

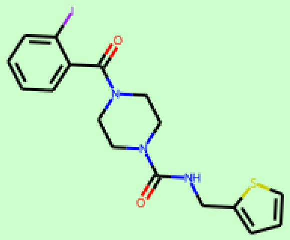
success

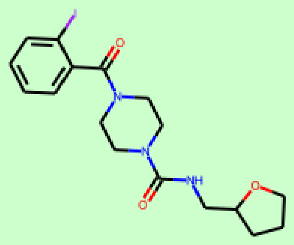
success

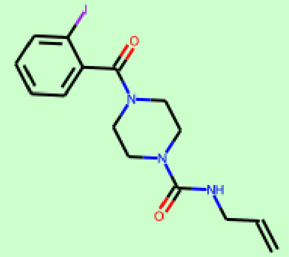
success

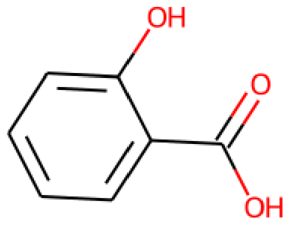
SM

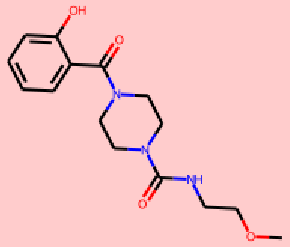
fail

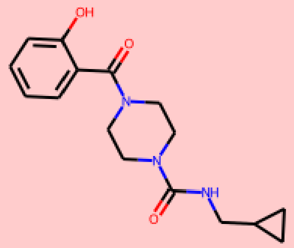
fail

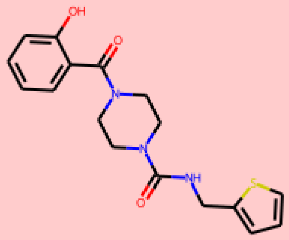
fail

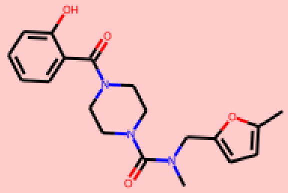
fail

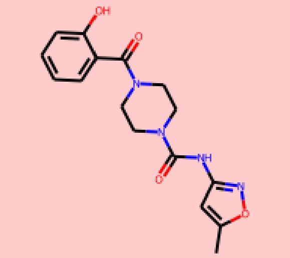
fail

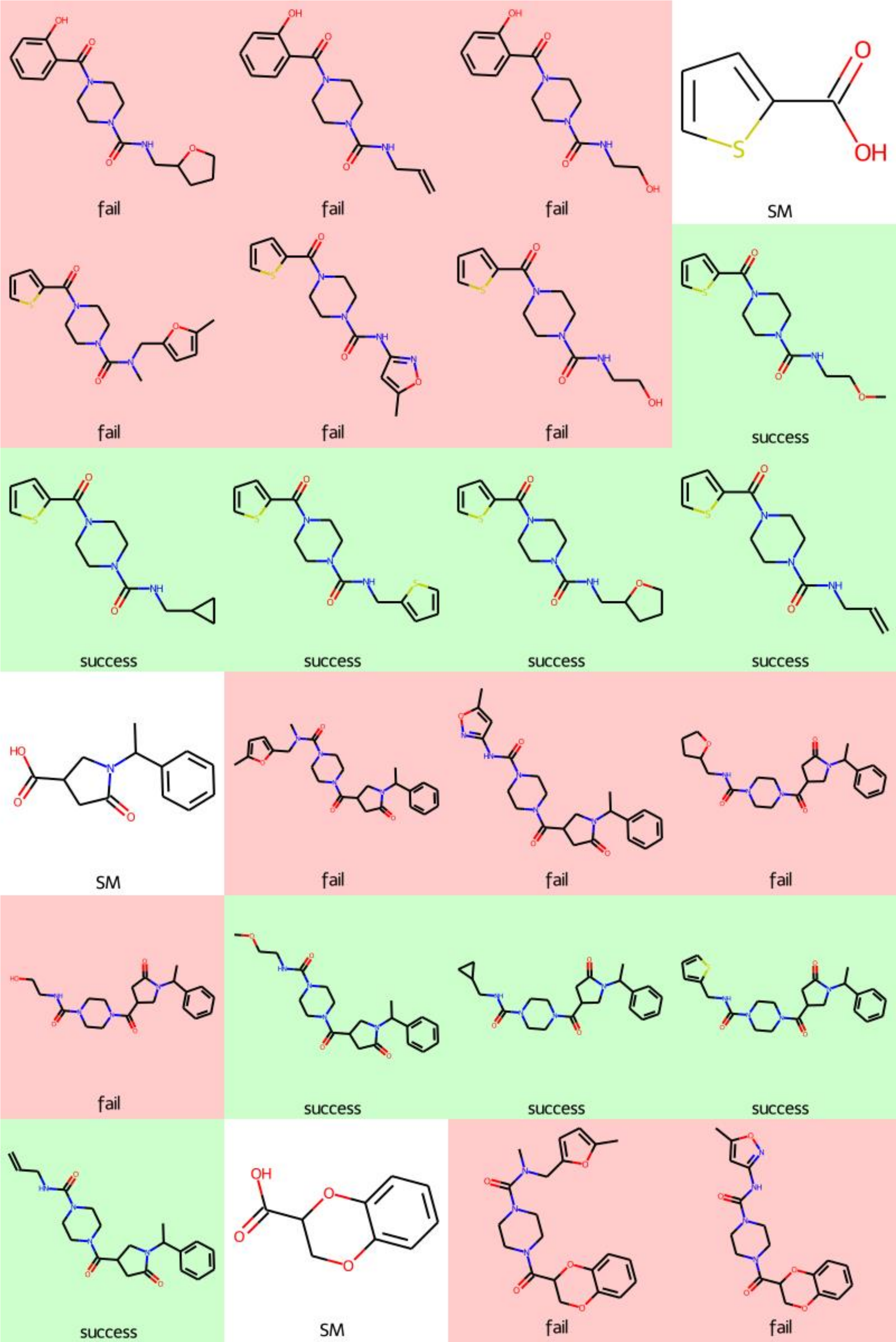
fail
fail
fail
SM
fail
fail
fail
success
success
success
success
success
SM
fail
fail
fail
fail
success
success
success
success
SM
fail
fail

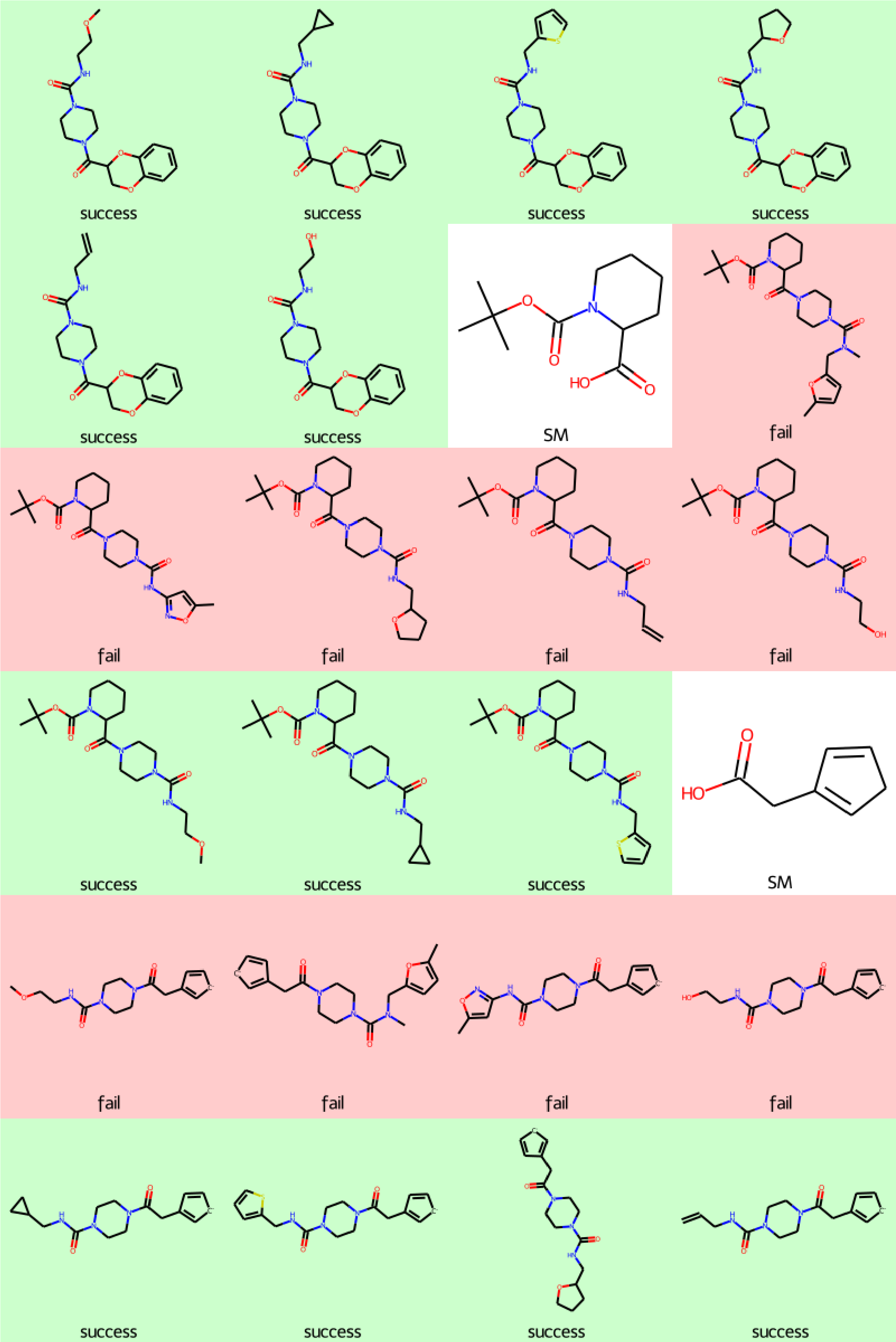
success
success
success
success
success
success
SM
fail
fail
fail
fail
fail
success
success
success
SM
fail
fail
fail
fail
success
success
success
success

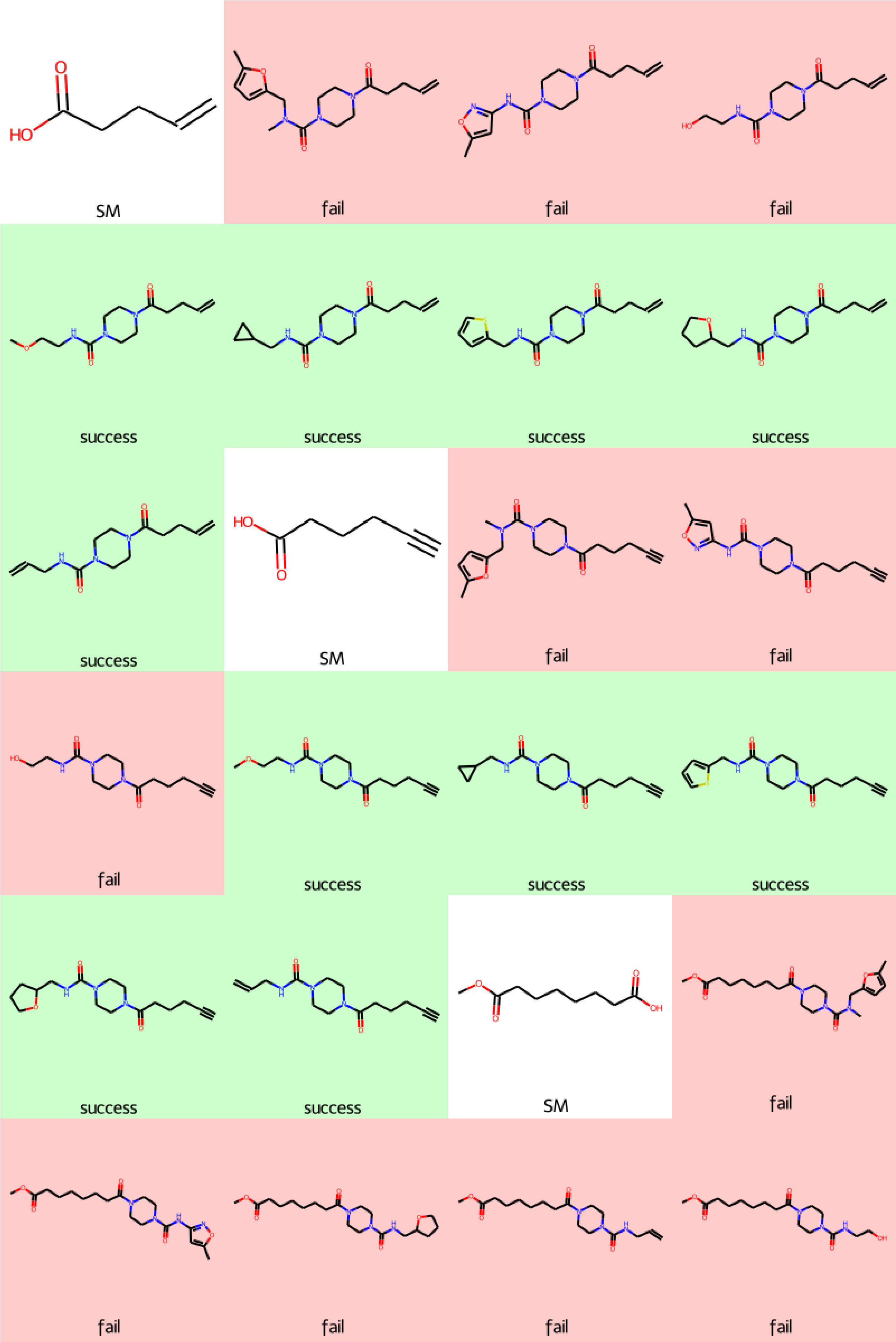
SM
fail
fail
fail
success
success
success
success
success
SM
fail
fail
fail
success
success
success
success
success
SM
fail
fail
fail
fail
fail

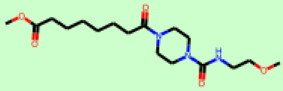

success

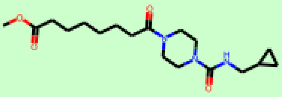

success

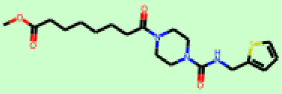

success

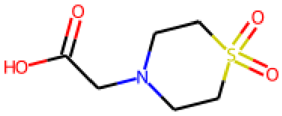

SM

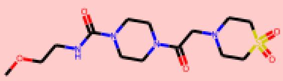

fail

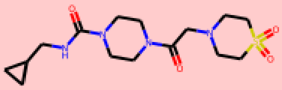

fail

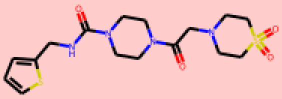

fail

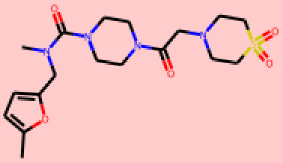

fail

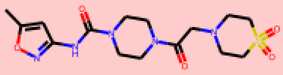

fail

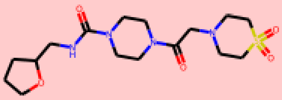

fail

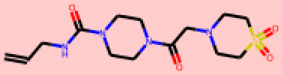

fail

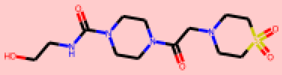

fail

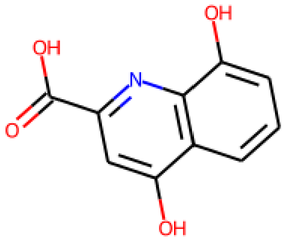

SM

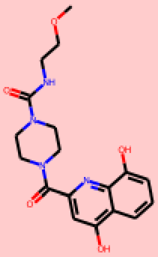

fail

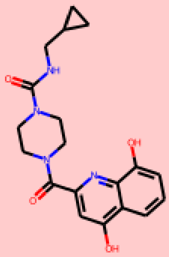

fail

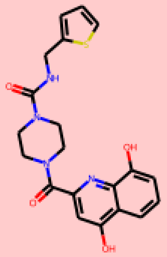

fail

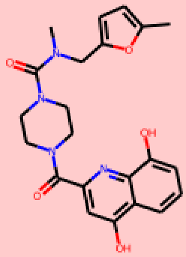

fail

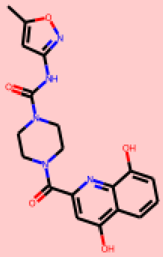

fail

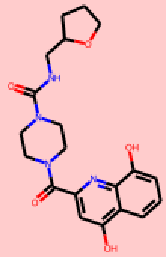

fail

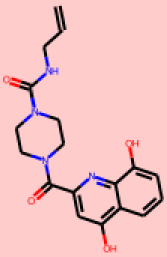

fail

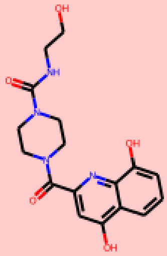

fail

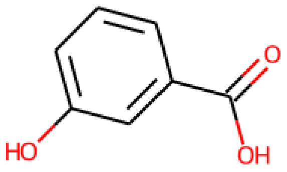

SM

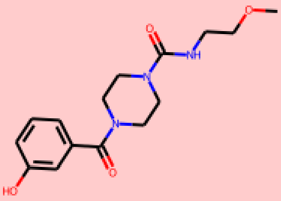

fail

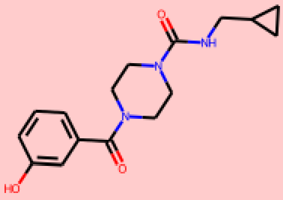

fail

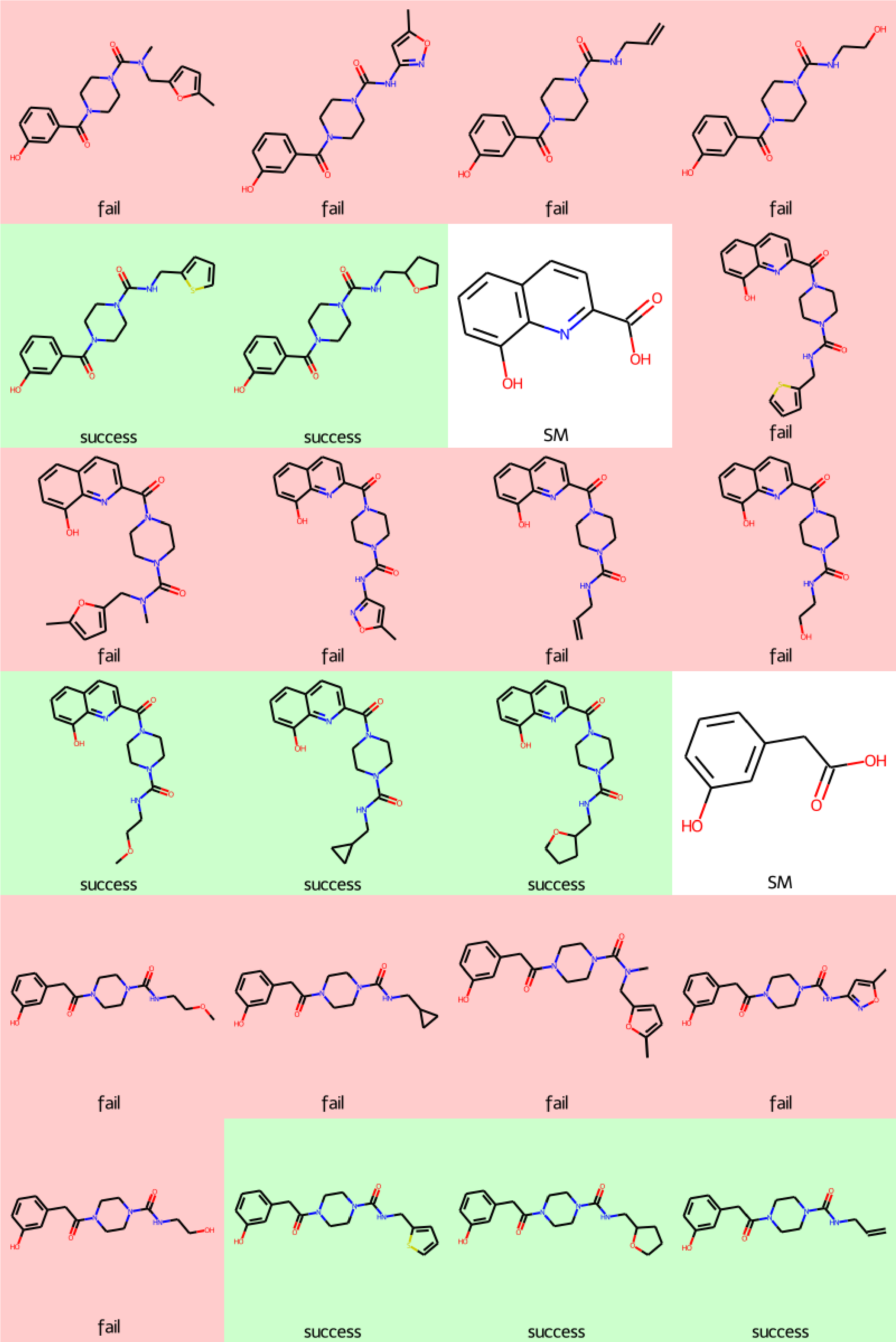
fail
fail
fail
fail
success
success
SM
fail
fail
fail
fail
fail
success
success
success
SM
fail
fail
fail
fail
fail
success
success
success

8.5 Iteration 3.5

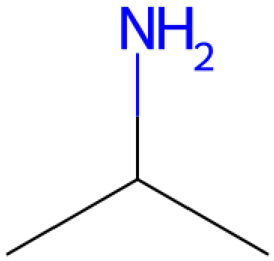

SM

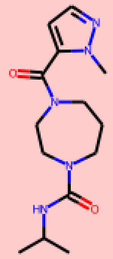

fail

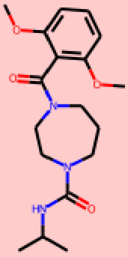

fail

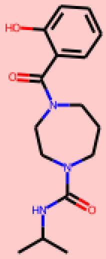

fail

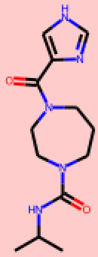

fail

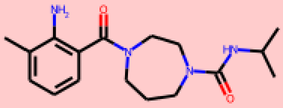

fail

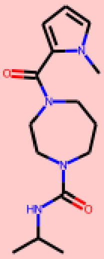

fail

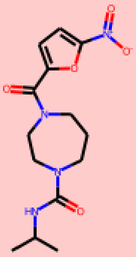

fail

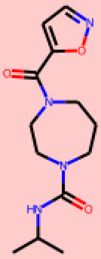

fail

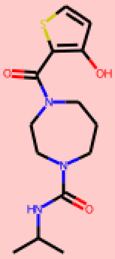

fail

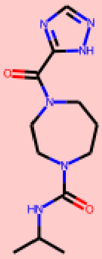

fail

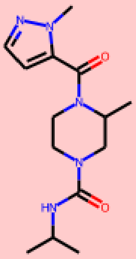

fail

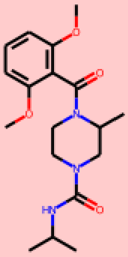

fail

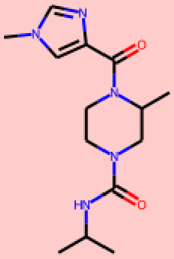

fail

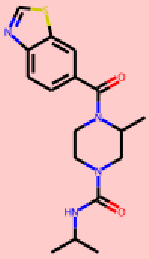

fail

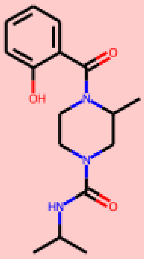

fail

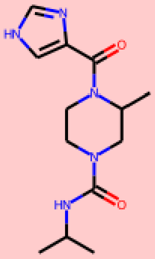

fail

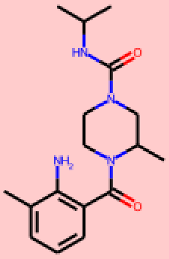

fail

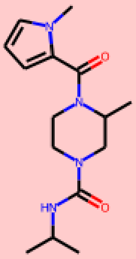

fail

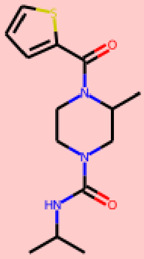

fail

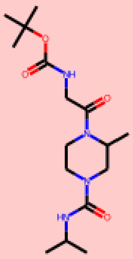

fail

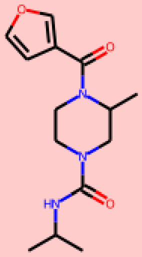

fail

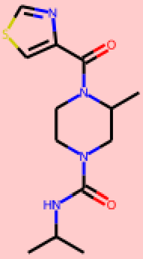

fail

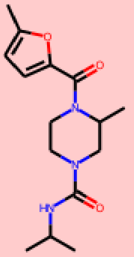

fail

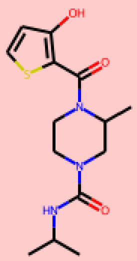
fail
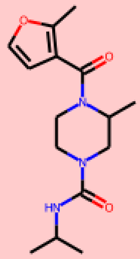
fail
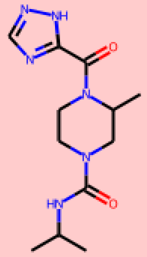
fail
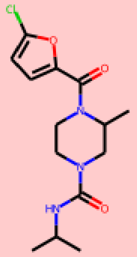
fail
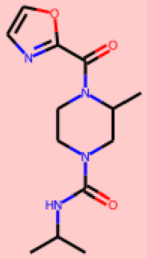
fail
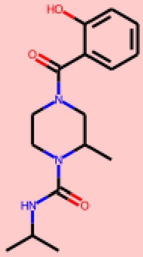
fail
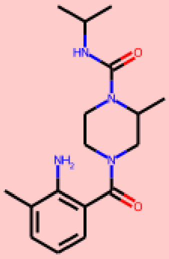
fail
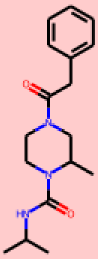
fail
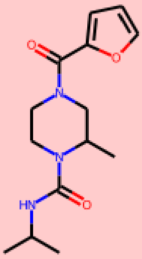
fail
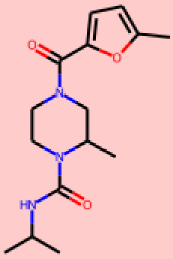
fail
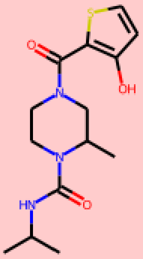
fail
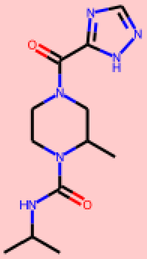
fail
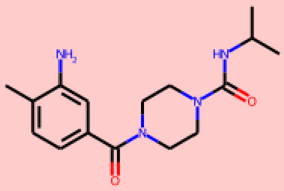
fail
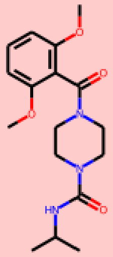
fail
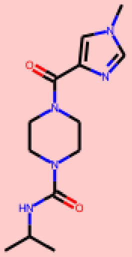
fail
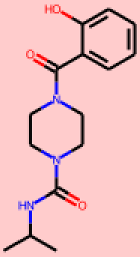
fail
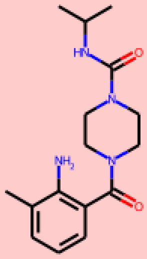
fail
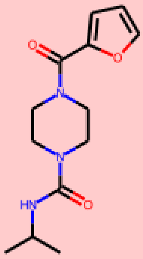
fail
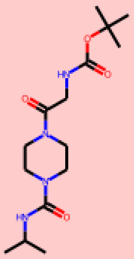
fail
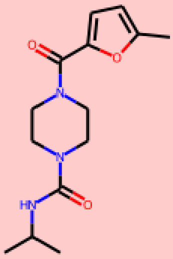
fail
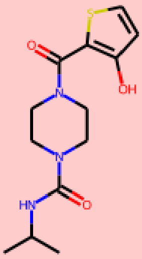
fail
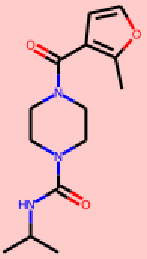
fail
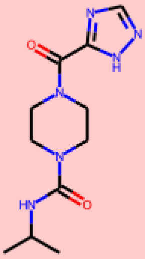
fail
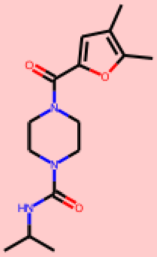
fail

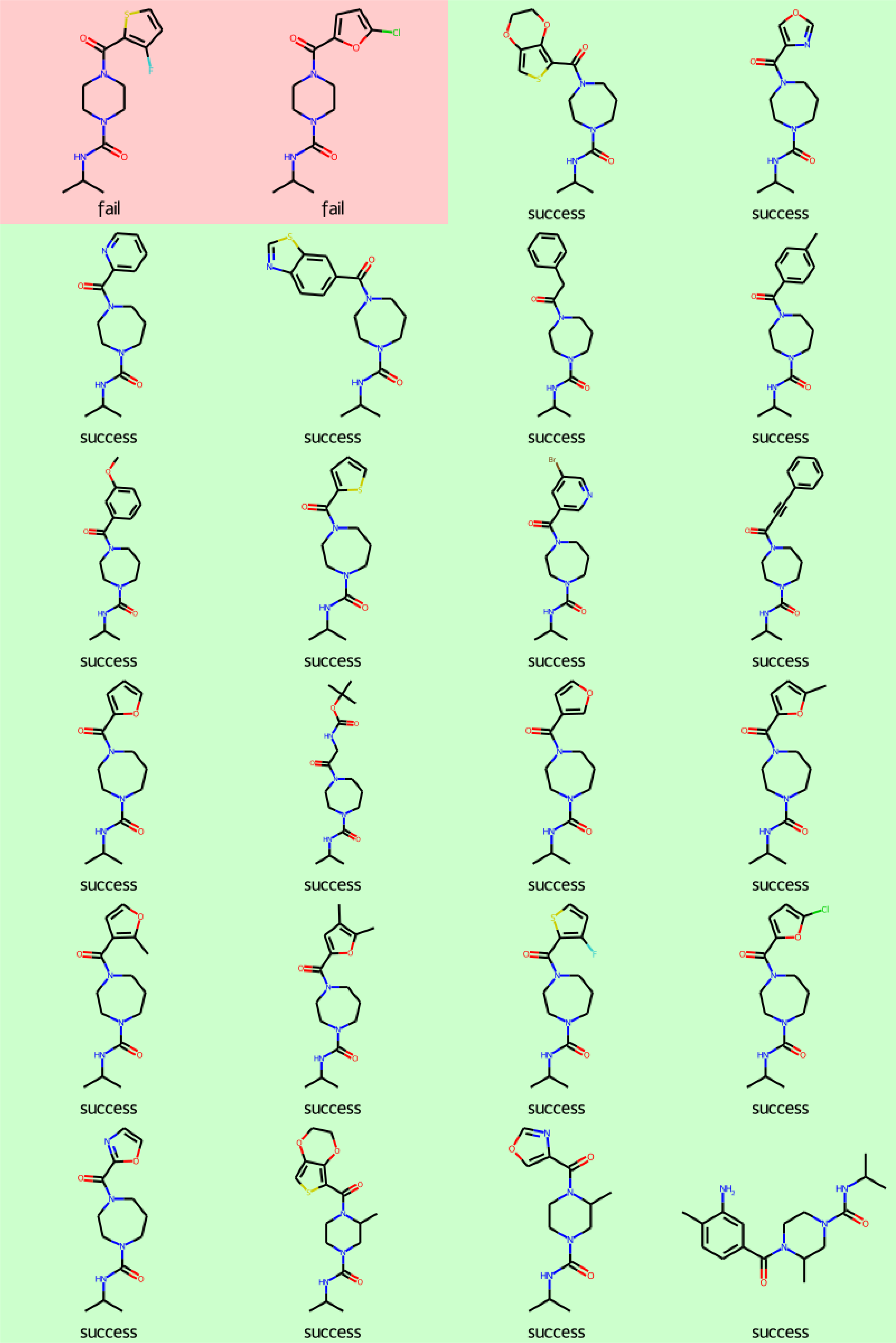
fail
fail
success
success
success
success
success
success
success
success
success
success
success
success
success
success
success
success
success
success
success
success
success
success

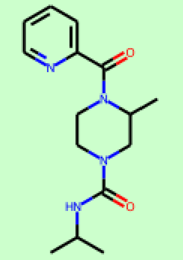
success
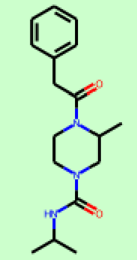
success
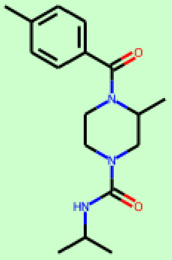
success
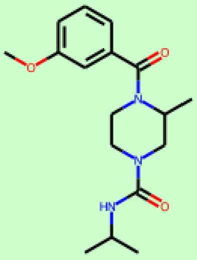
success
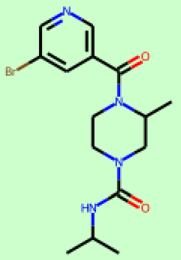
success
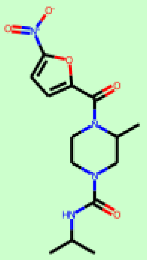
success
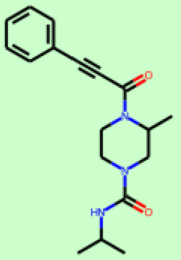
success
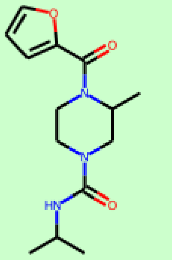
success
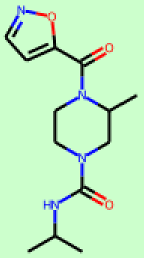
success
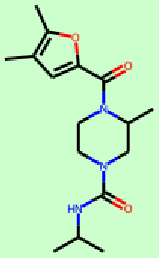
success
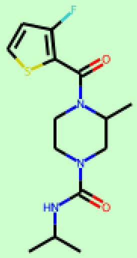
success
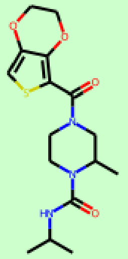
success
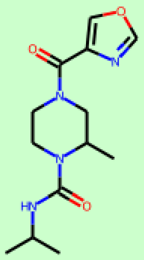
success
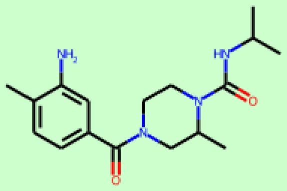
success
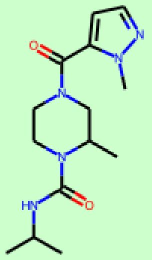
success
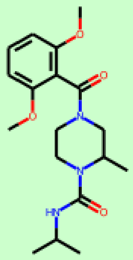
success
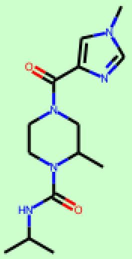
success
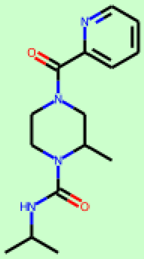
success
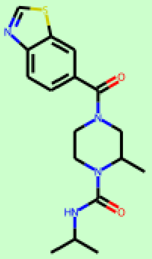
success
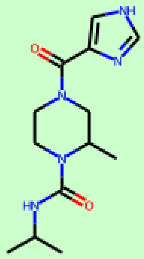
success
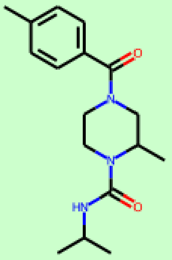
success
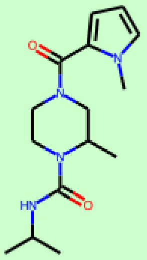
success
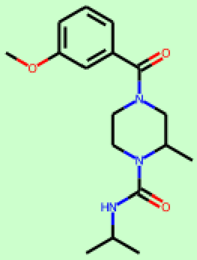
success
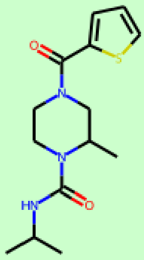
success

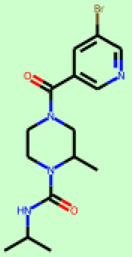
success
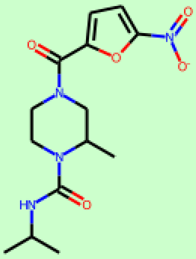
success
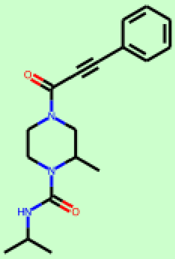
success
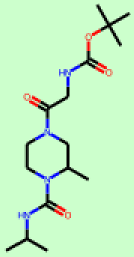
success
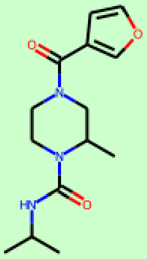
success
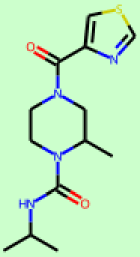
success
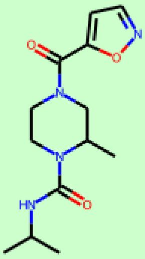
success
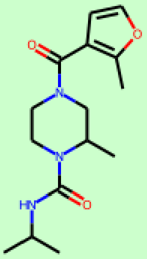
success
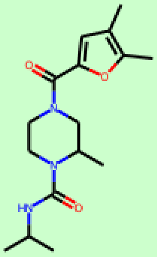
success
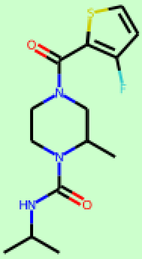
success
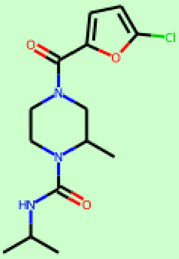
success
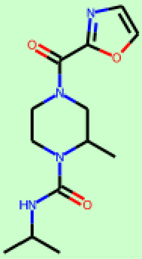
success
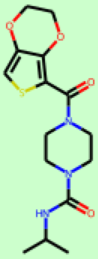
success
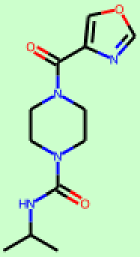
success
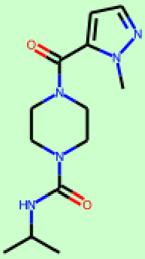
success
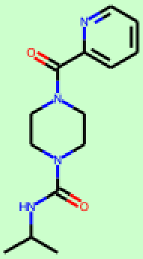
success
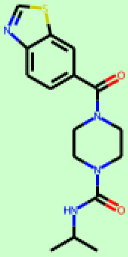
success
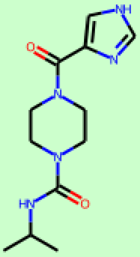
success
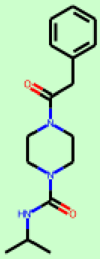
success
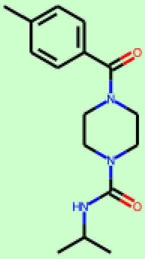
success
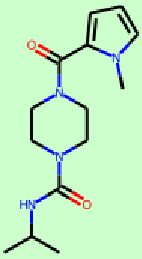
success
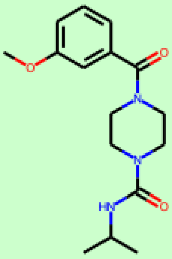
success
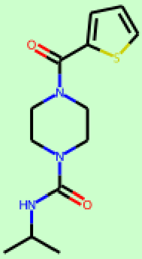
success
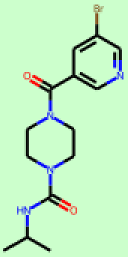
success

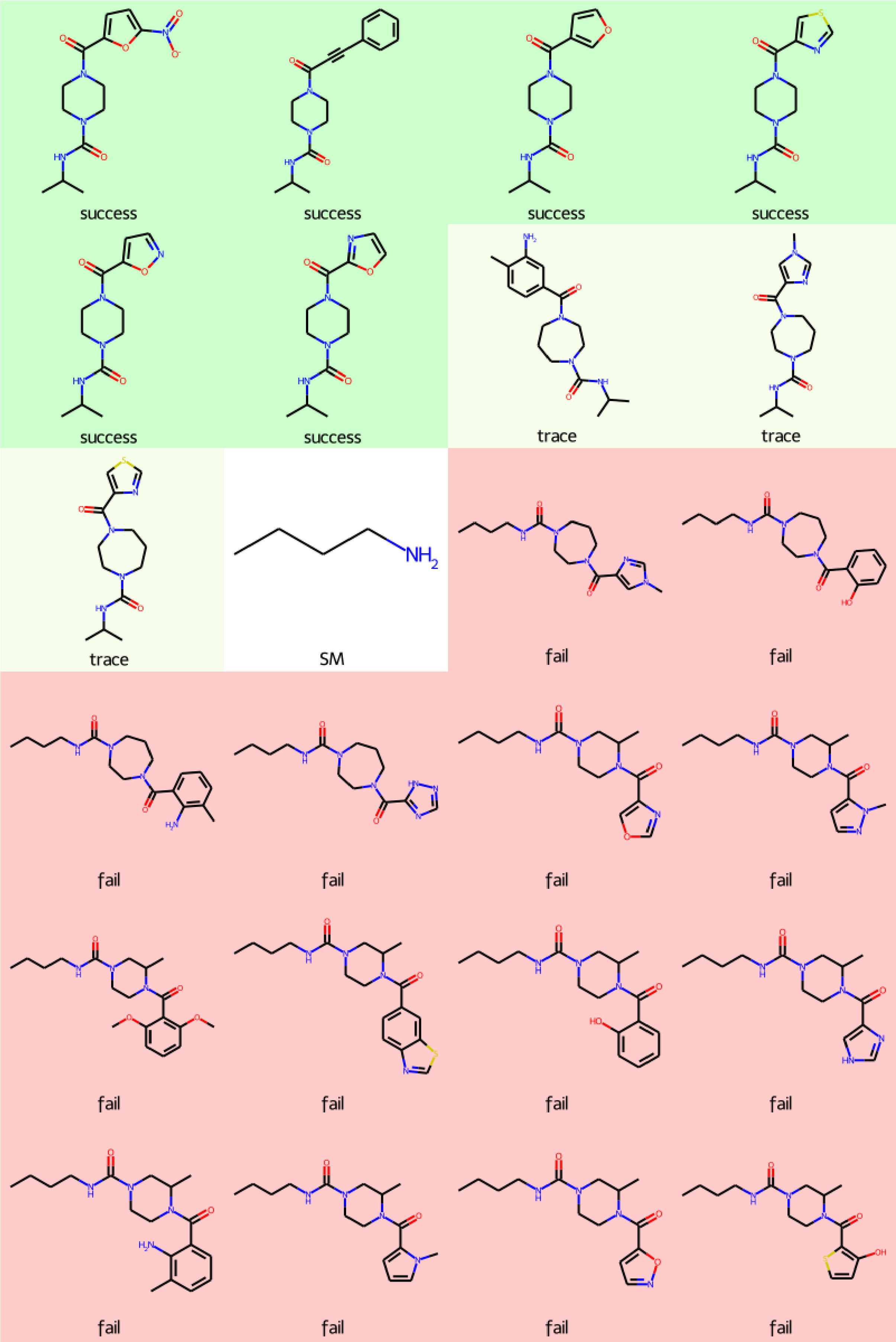
success
success
success
success
success
success
trace
trace
trace
SM
fail
fail
fail
fail
fail
fail
fail
fail
fail
fail
fail
fail
fail
fail
fail
fail

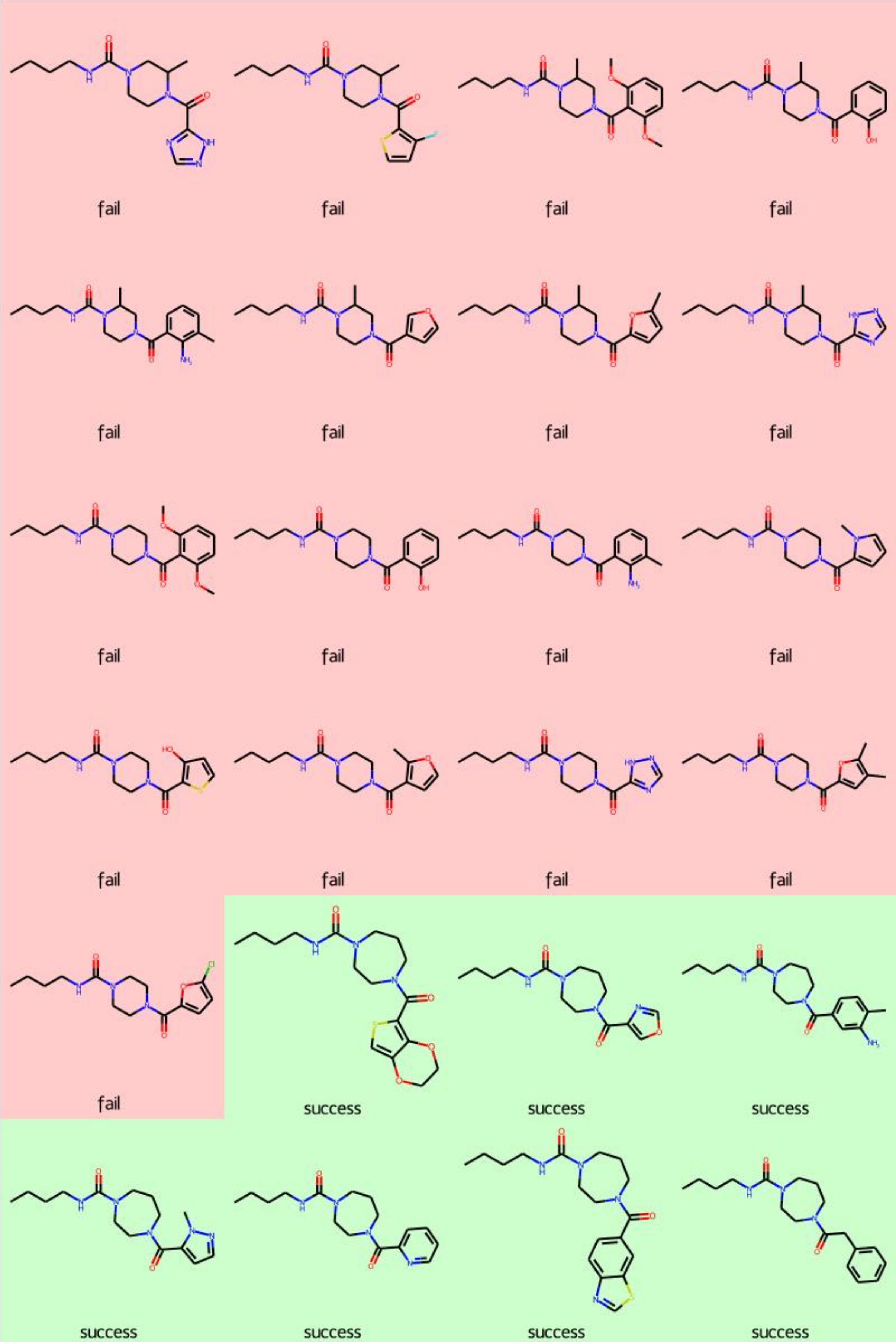
fail
fail
fail
fail
fail
fail
fail
fail
fail
fail
fail
fail
fail
fail
fail
fail
fail
success
success
success
success
success
success
success

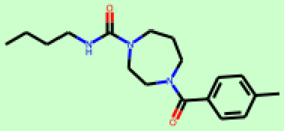

success

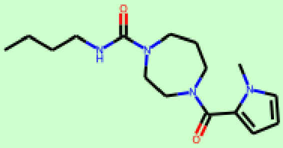

success

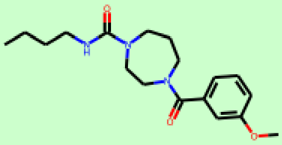

success

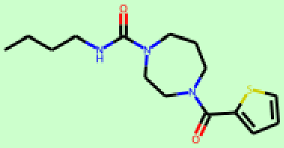

success

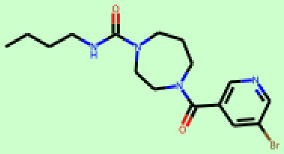

success

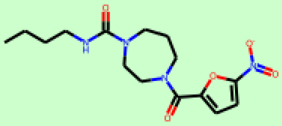

success

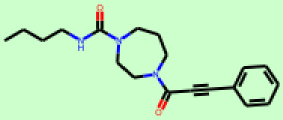

success

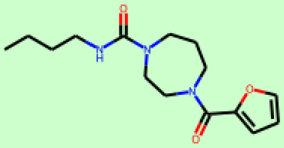

success

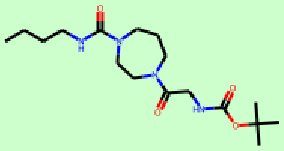

success

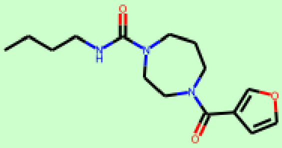

success

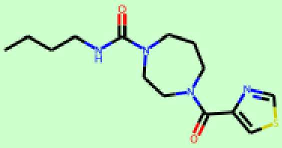

success

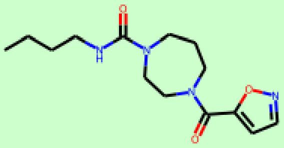

success

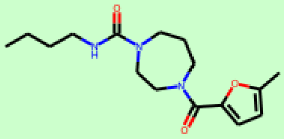

success

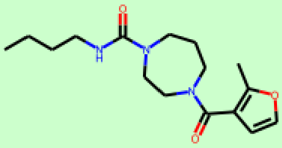

success

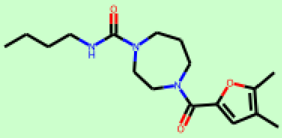

success

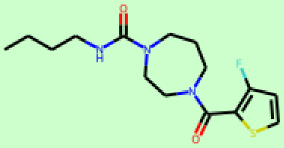

success

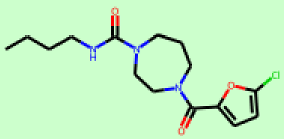

success

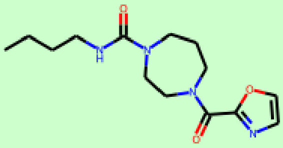

success

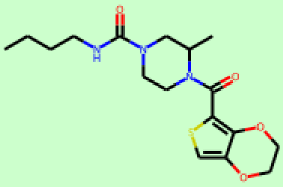

success

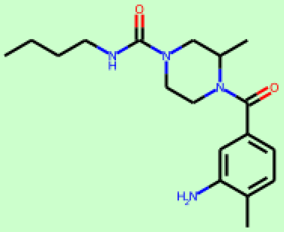

success

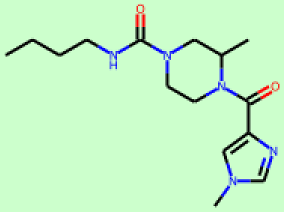

success

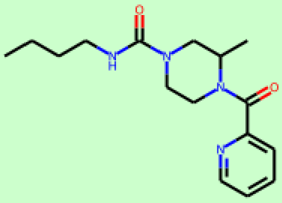

success

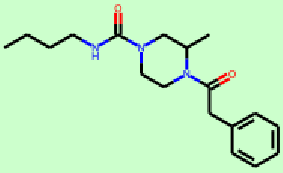

success

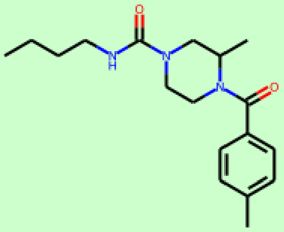

success

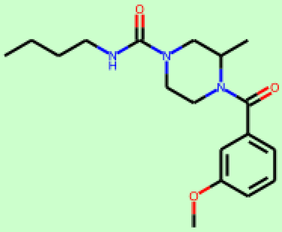

success

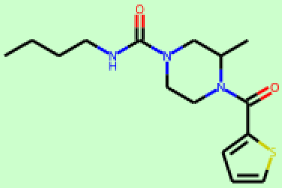

success

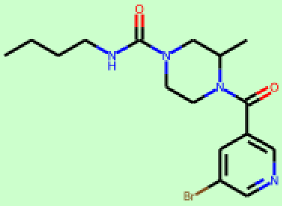

success

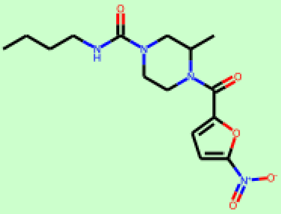

success

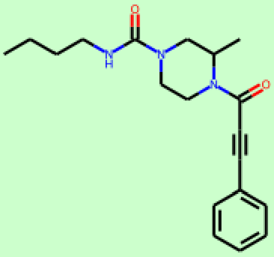

success

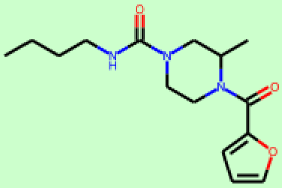

success

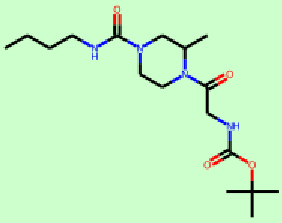

success

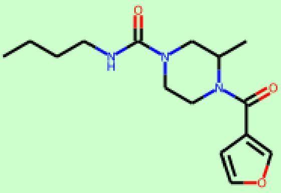

success

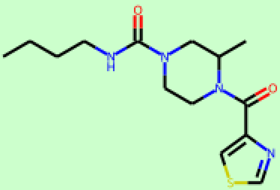

success

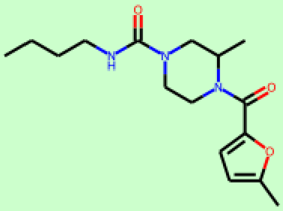

success

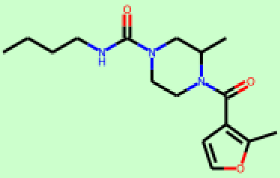

success

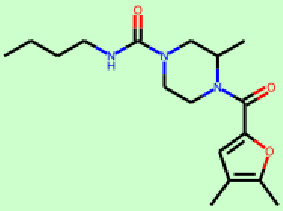

success

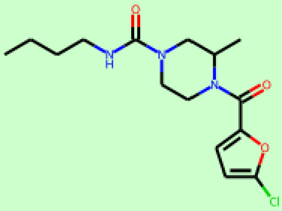

success

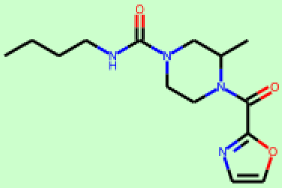

success

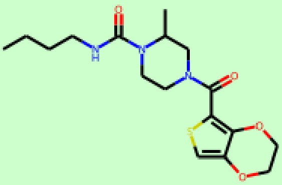

success

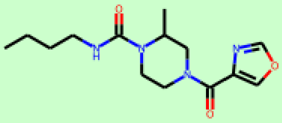

success

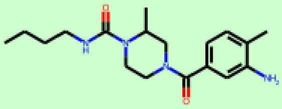

success

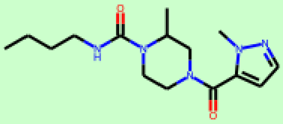

success

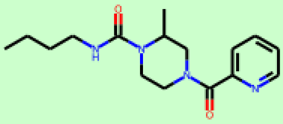

success

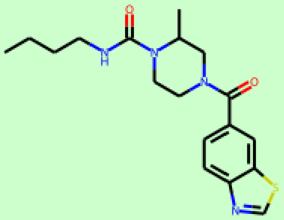

success

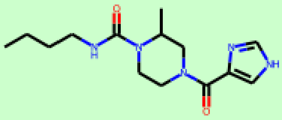

success

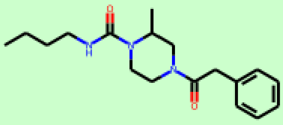

success

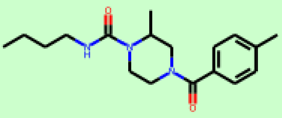

success

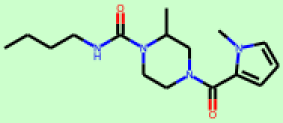

success

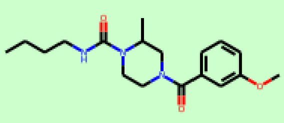

success

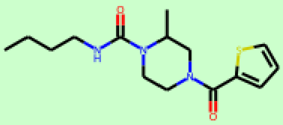

success

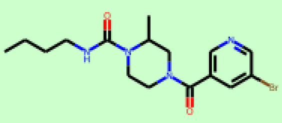

success

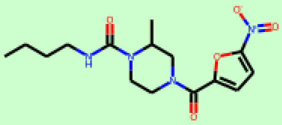

success

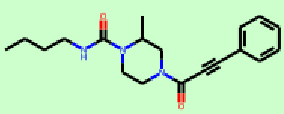

success

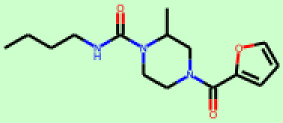

success

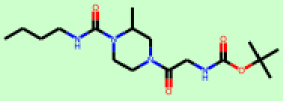

success

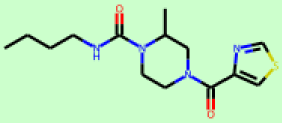

success

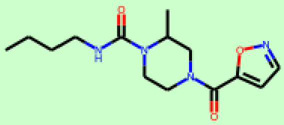

success

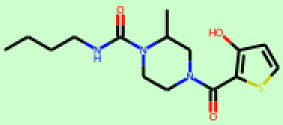

success

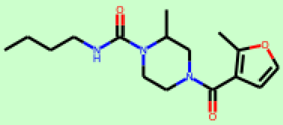

success

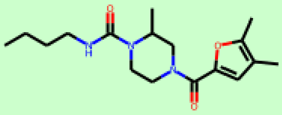

success

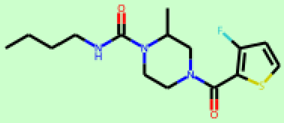

success

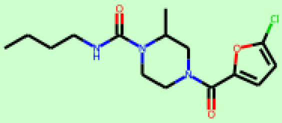

success

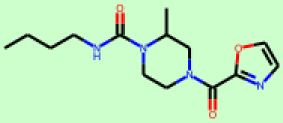

success

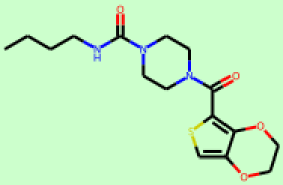

success

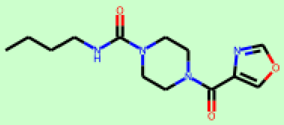

success

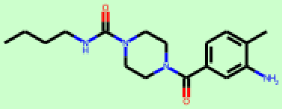

success

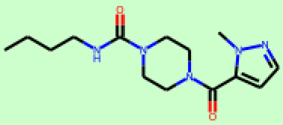

success

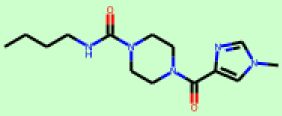

success

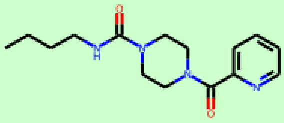

success

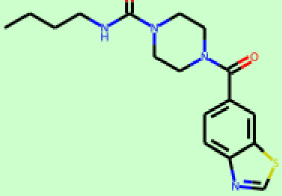

success

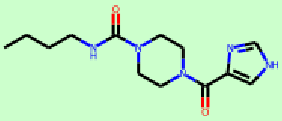

success

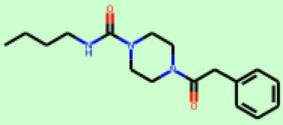

success

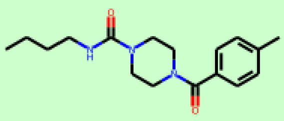
success

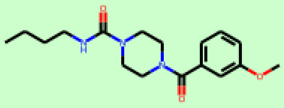
success

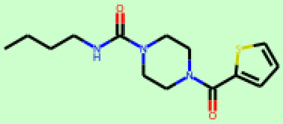
success

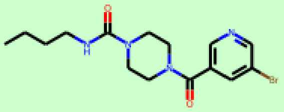
success

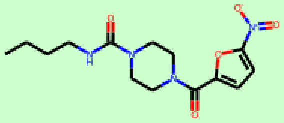
success

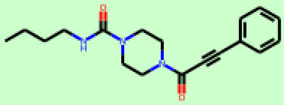
success

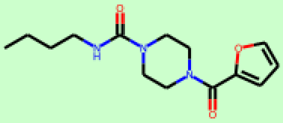
success

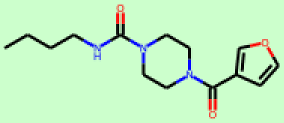
success

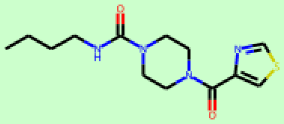
success

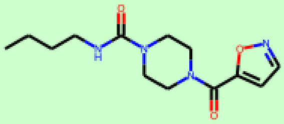
success

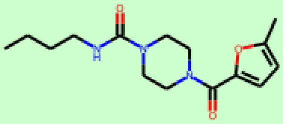
success

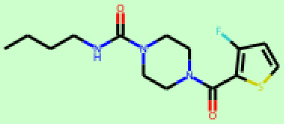
success

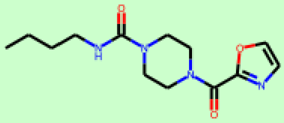
success

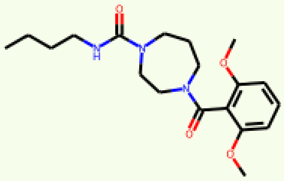
trace

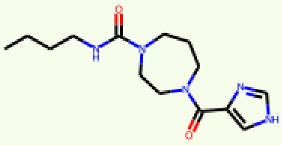
trace

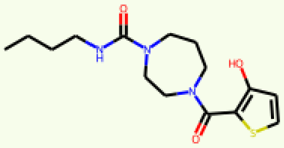
trace

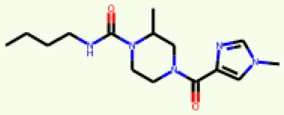
trace

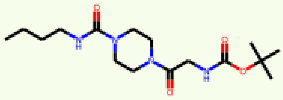
trace

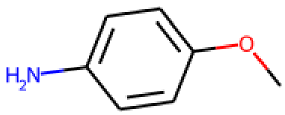
SM

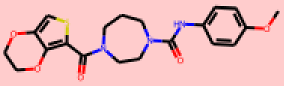
fail

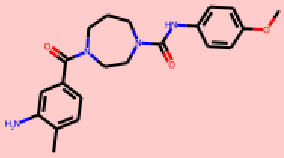
fail

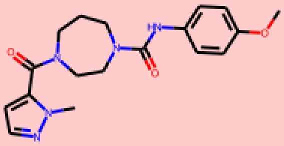
fail

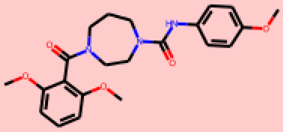
fail

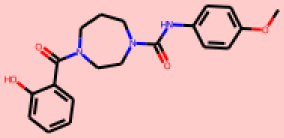
fail

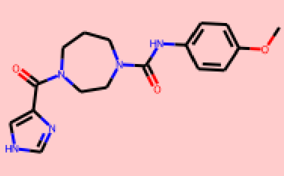

fail

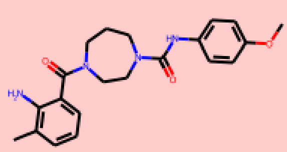

fail

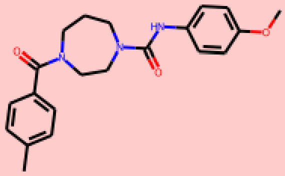

fail

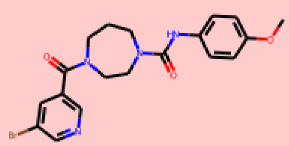

fail

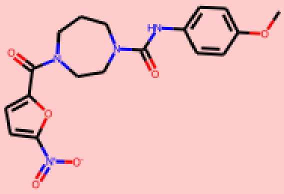

fail

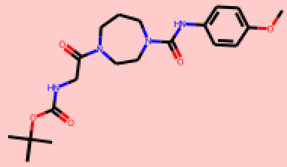

fail

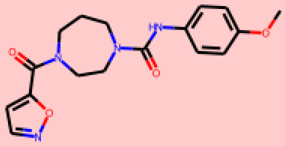

fail

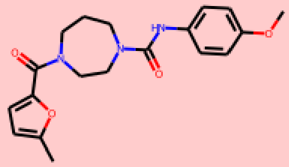

fail

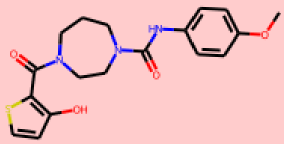

fail

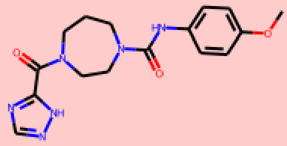

fail

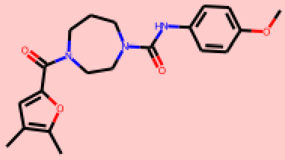

fail

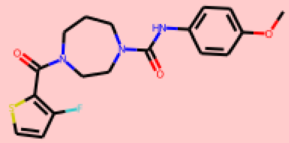

fail

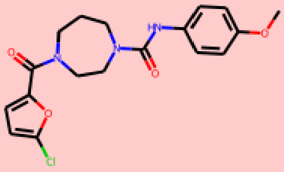

fail

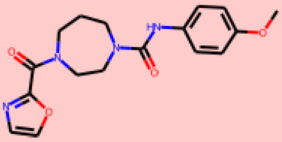

fail

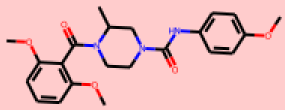

fail

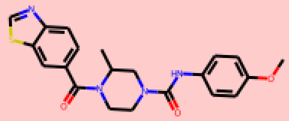

fail

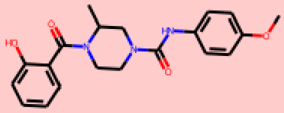

fail

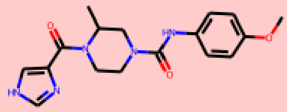

fail

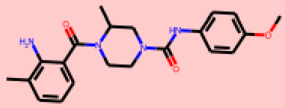

fail

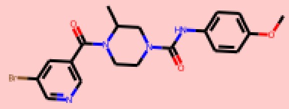

fail

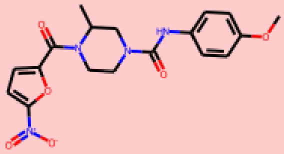

fail

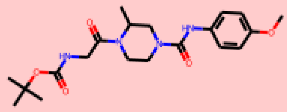

fail

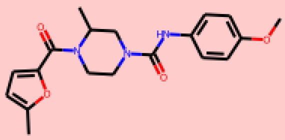

fail

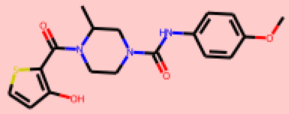

fail

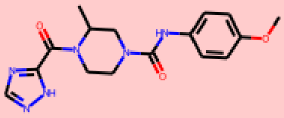 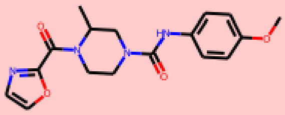 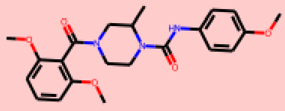 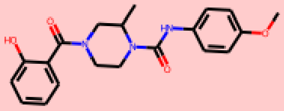

fail fail fail fail

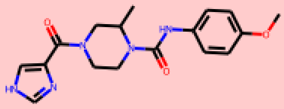 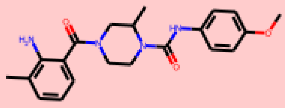 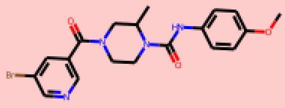 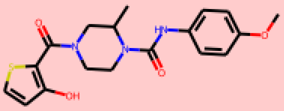

fail fail fail fail

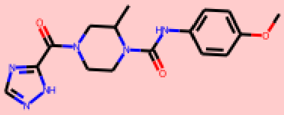 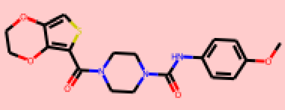 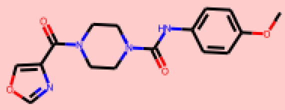 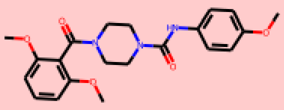

fail fail fail fail

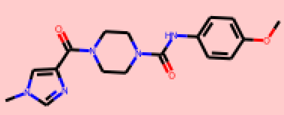 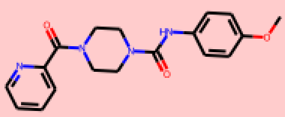 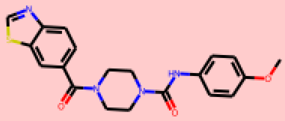 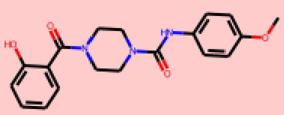

fail fail fail fail

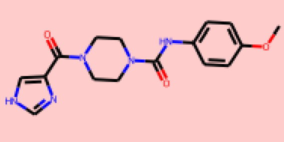 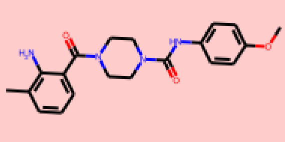 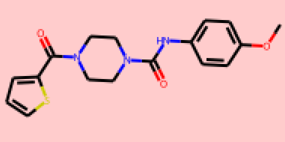 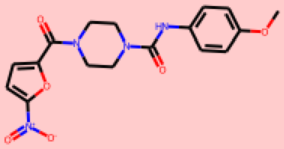

fail fail fail fail

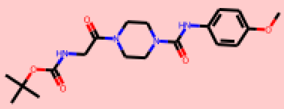 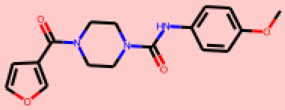 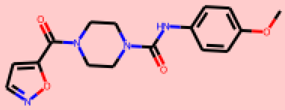 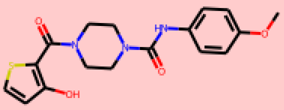

fail fail fail fail

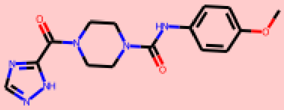
fail

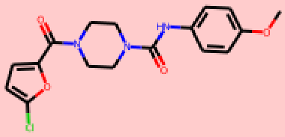
fail

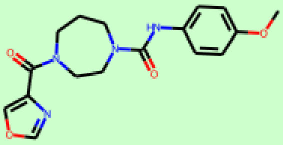
success

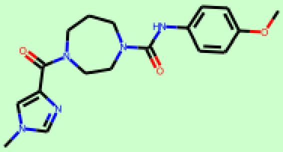
success

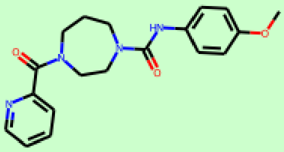
success

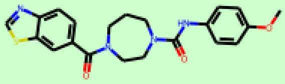
success

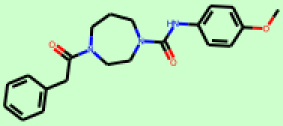
success

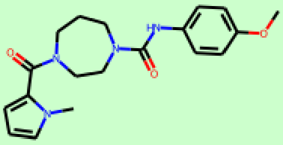
success

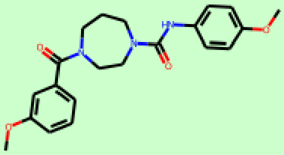
success

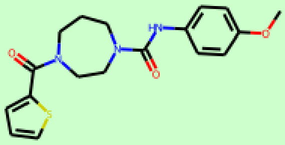
success

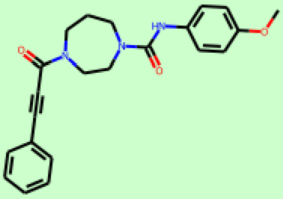
success

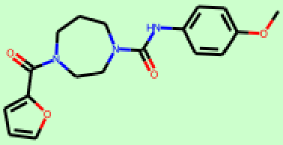
success

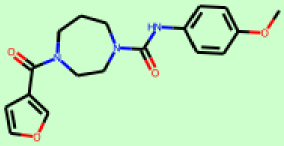
success

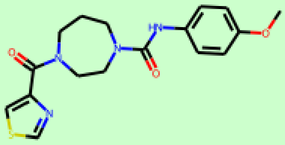
success

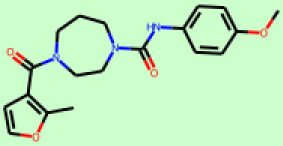
success

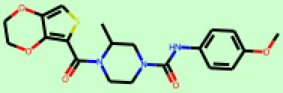
success

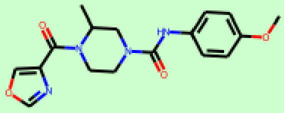
success

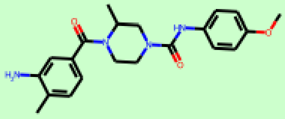
success

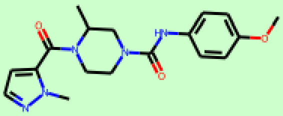
success

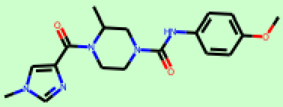
success

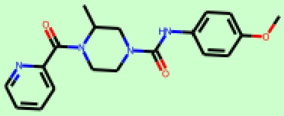
success

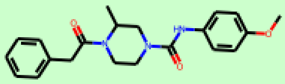
success

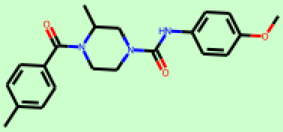
success

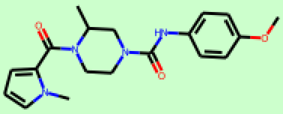
success

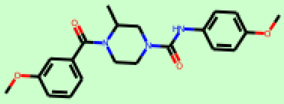

success

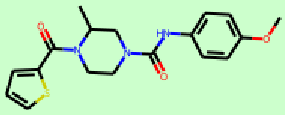

success

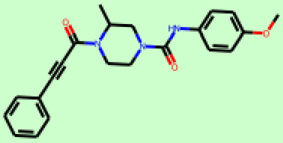

success

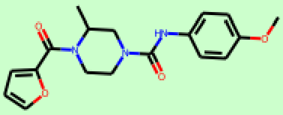

success

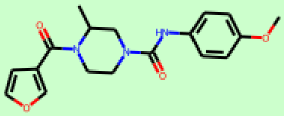

success

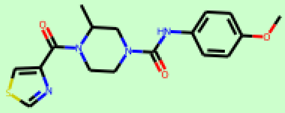

success

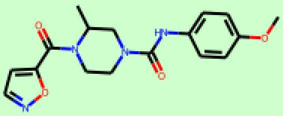

success

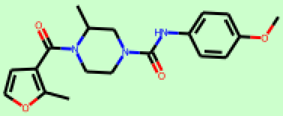

success

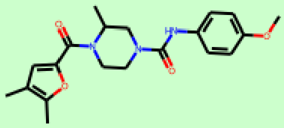

success

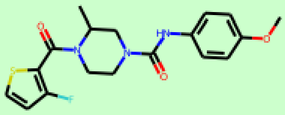

success

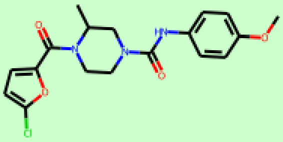

success

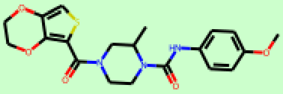

success

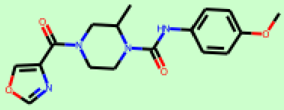

success

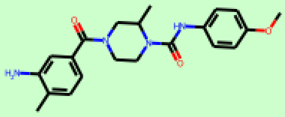

success

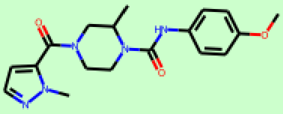

success

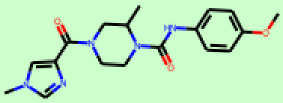

success

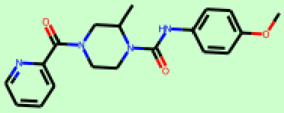

success

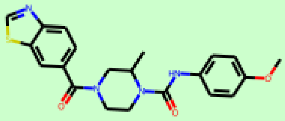

success

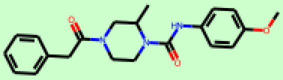

success

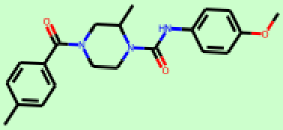

success

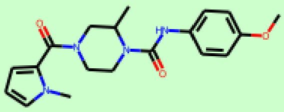

success

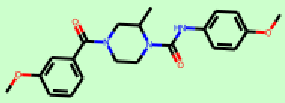

success

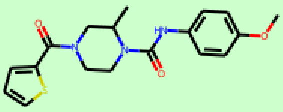

success

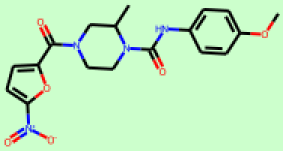

success

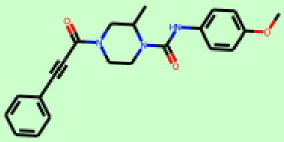
success
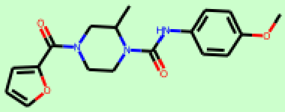
success
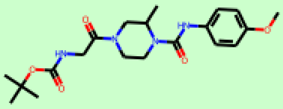
success
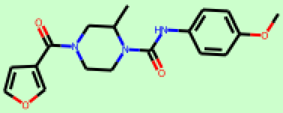
success
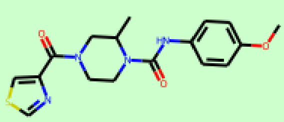
success
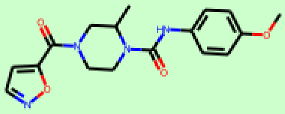
success
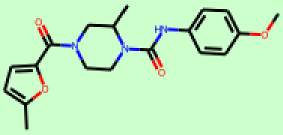
success
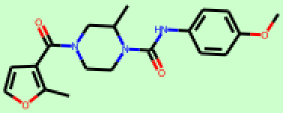
success
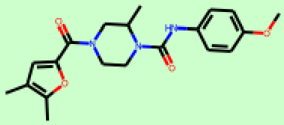
success
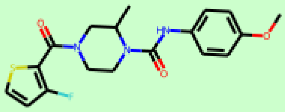
success
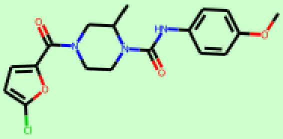
success
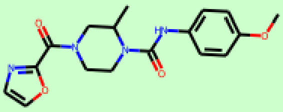
success
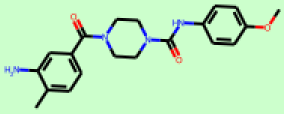
success
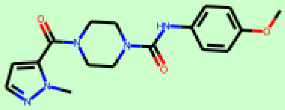
success
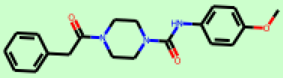
success
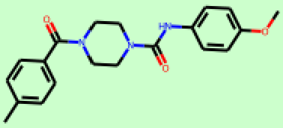
success
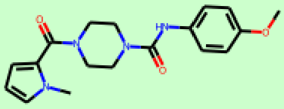
success
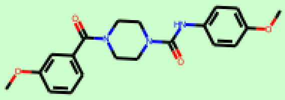
success
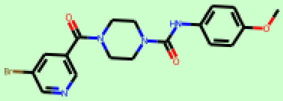
success
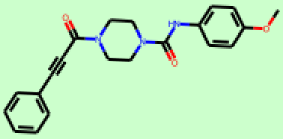
success
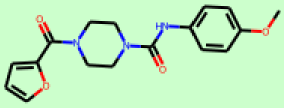
success
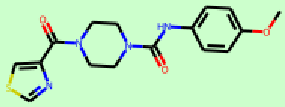
success
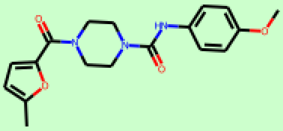
success
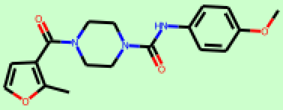
success

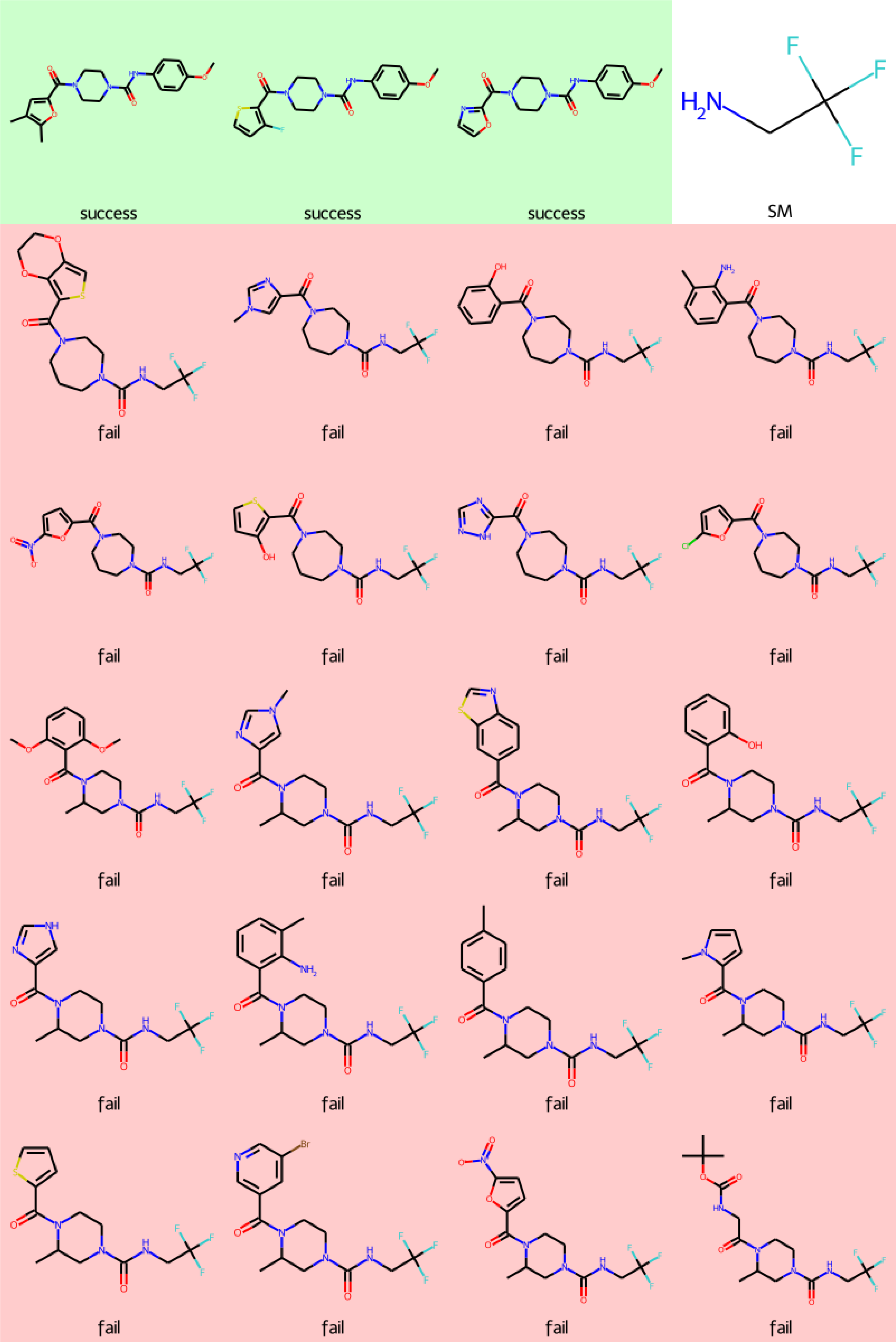
success
success
success
SM
fail
fail
fail
fail
fail
fail
fail
fail
fail
fail
fail
fail
fail
fail
fail
fail
fail
fail
fail
fail

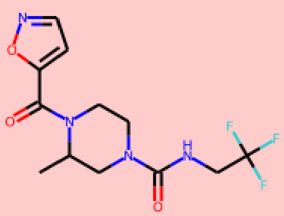
fail

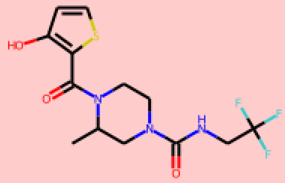
fail

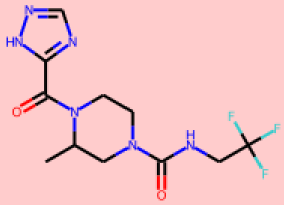
fail

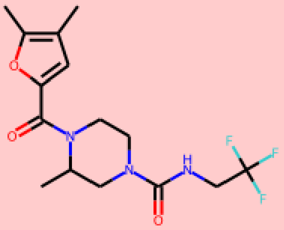
fail

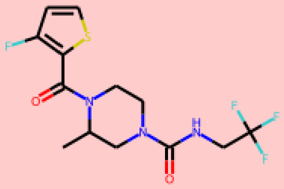
fail

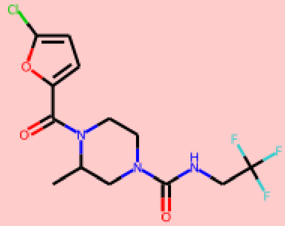
fail

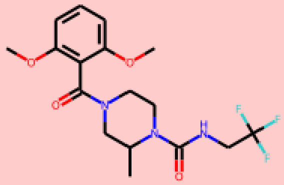
fail

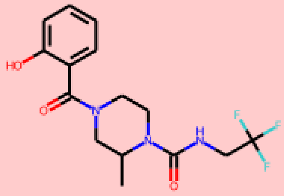
fail

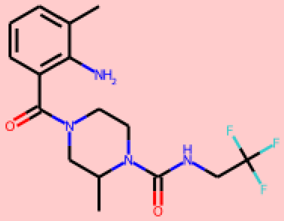
fail

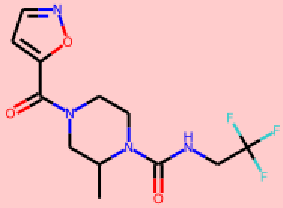
fail

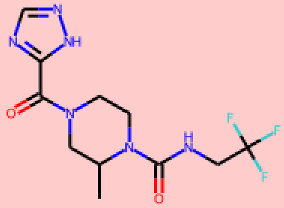
fail

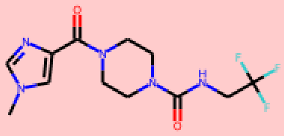
fail

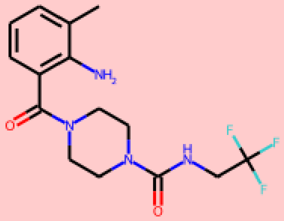
fail

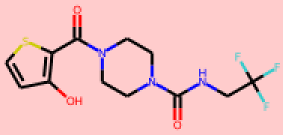
fail

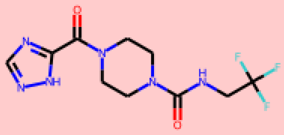
fail

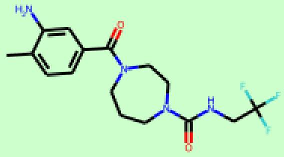
success

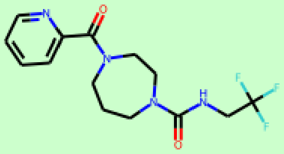
success

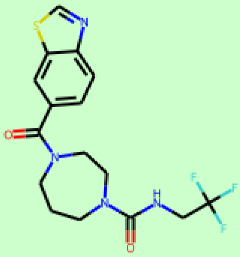
success

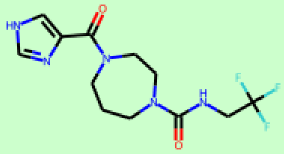
success

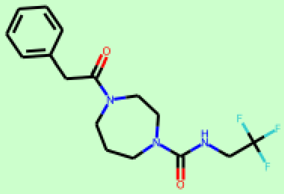
success

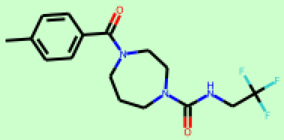
success

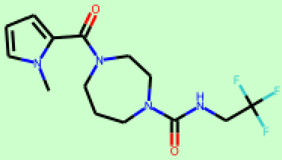
success

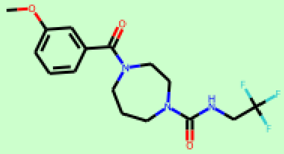
success

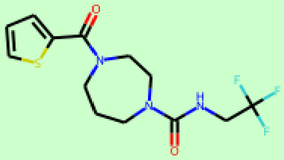
success

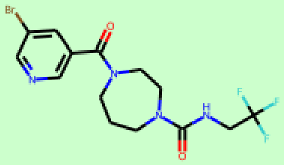

success

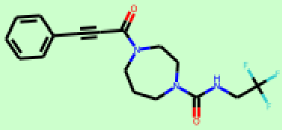

success

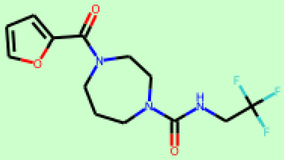

success

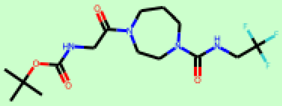

success

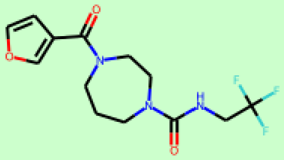

success

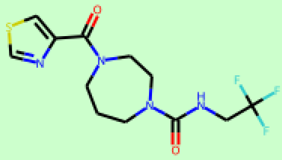

success

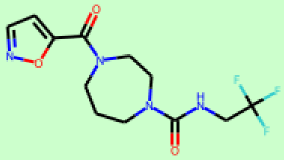

success

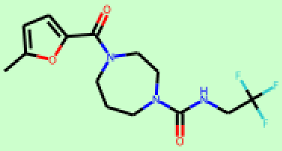

success

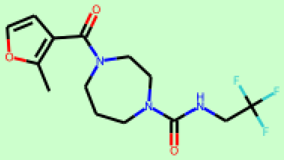

success

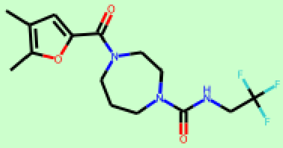

success

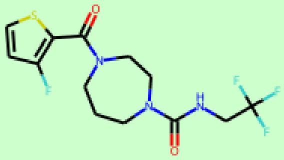

success

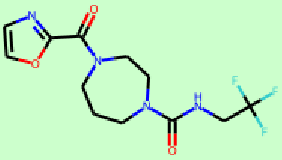

success

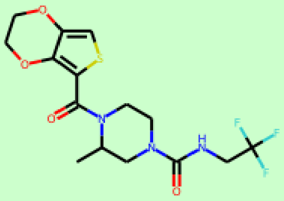

success

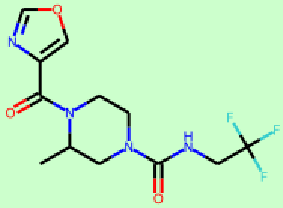

success

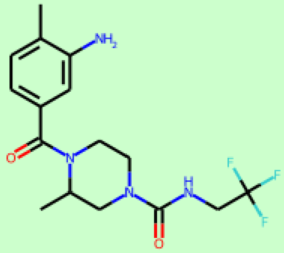

success

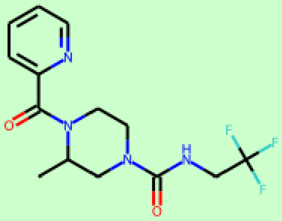

success

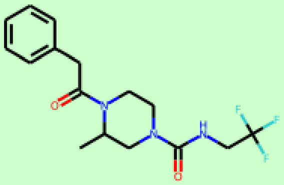

success

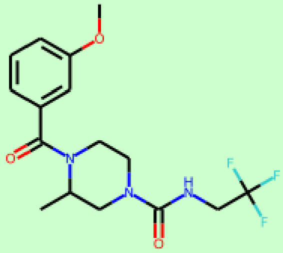

success

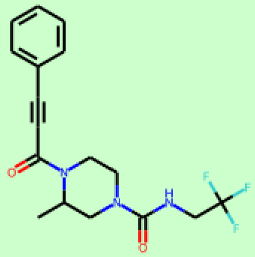

success

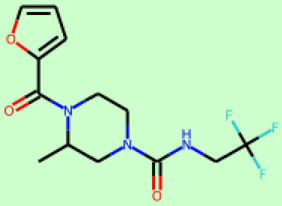

success

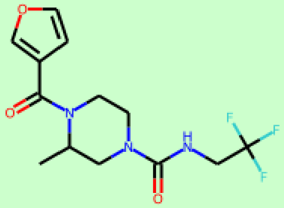

success

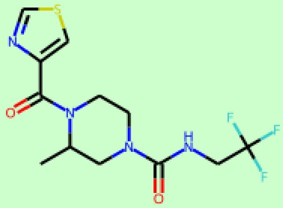

success

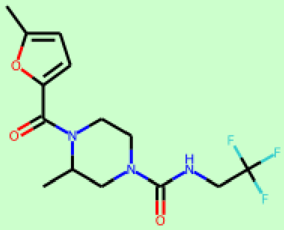

success

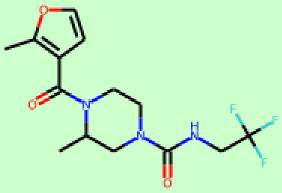

success

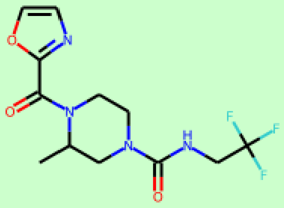

success

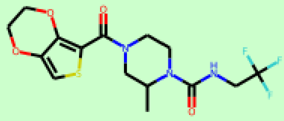

success

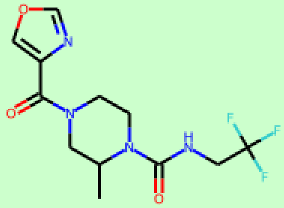

success

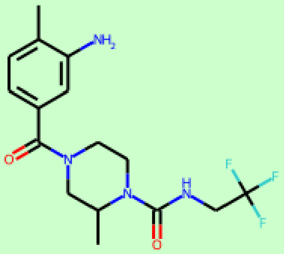

success

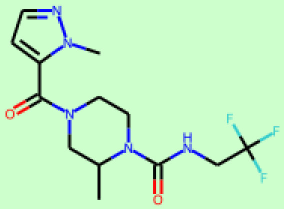

success

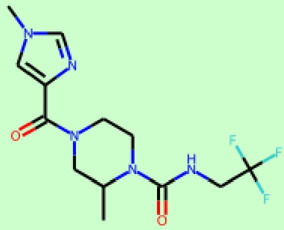

success

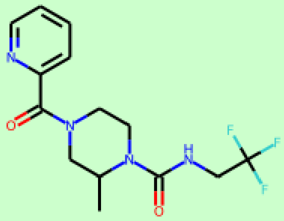

success

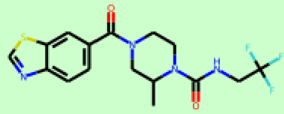

success

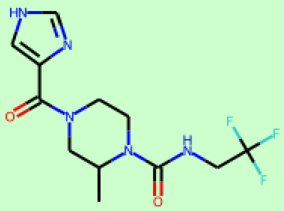

success

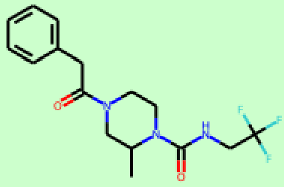

success

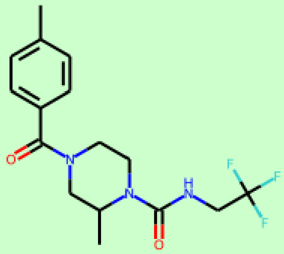

success

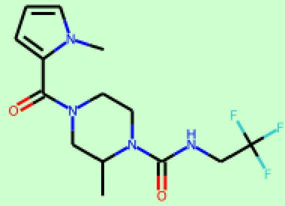

success

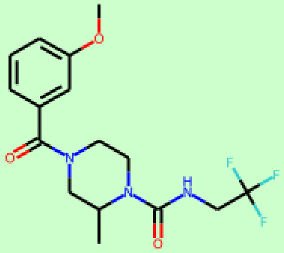

success

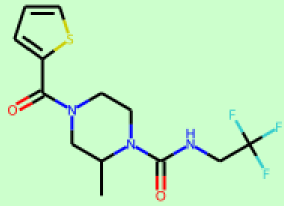

success

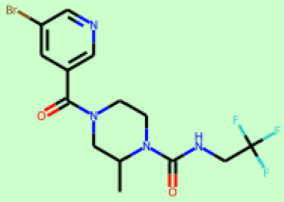

success

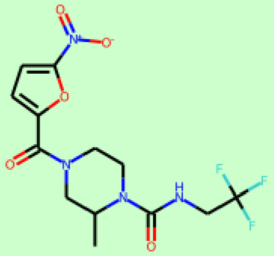

success

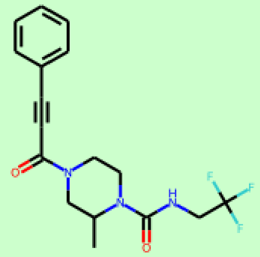

success

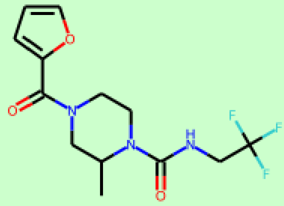

success

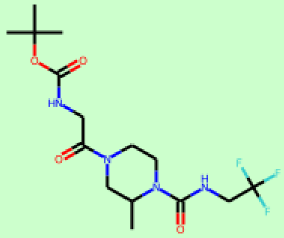

success

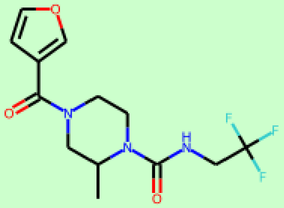

success

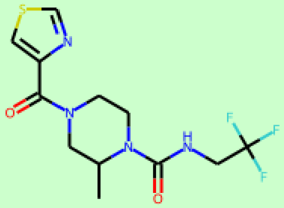

success

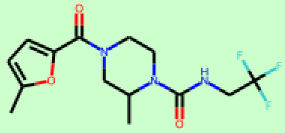

success

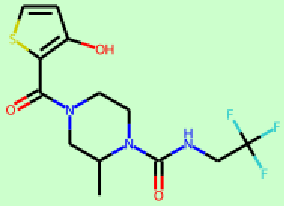

success

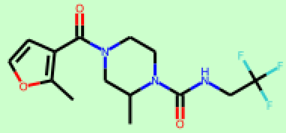

success

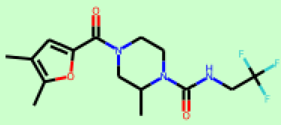

success

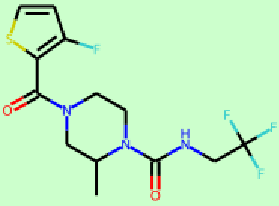

success

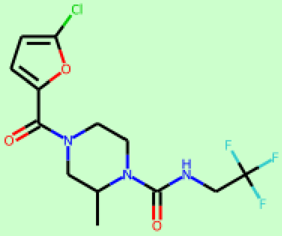

success

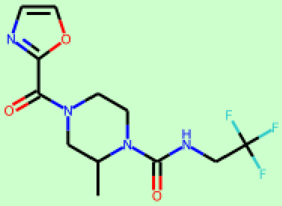

success

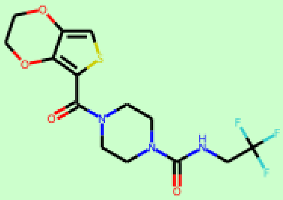

success

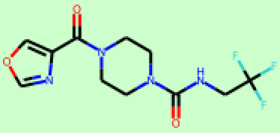

success

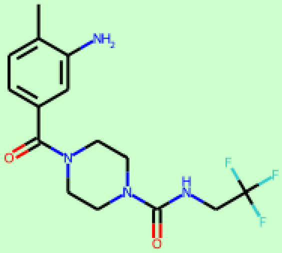

success

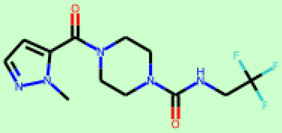

success

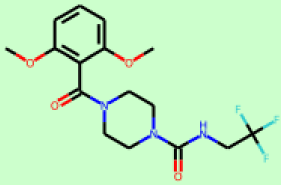

success

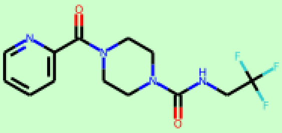

success

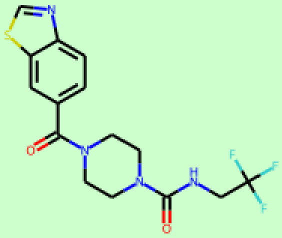

success

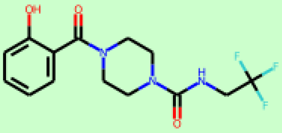

success

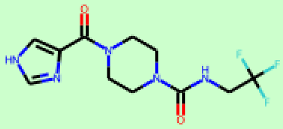

success

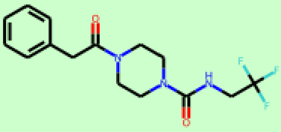

success

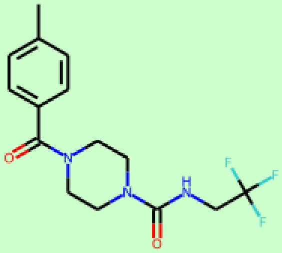

success

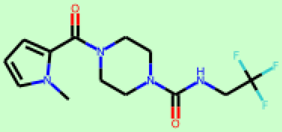

success

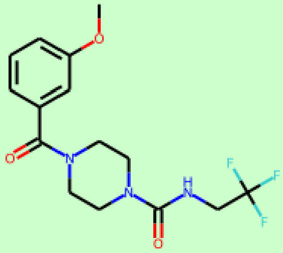

success

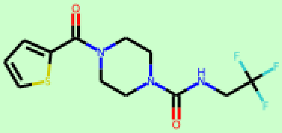

success

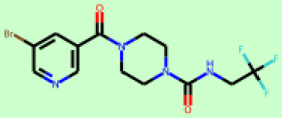

success

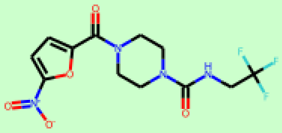

success

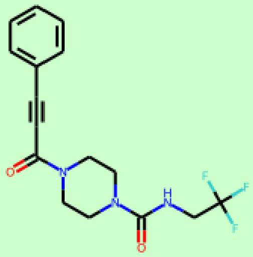

success

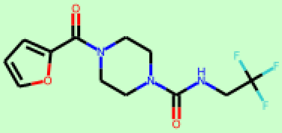

success

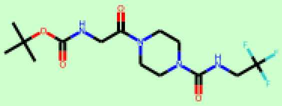

success

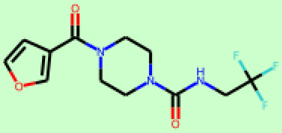

success

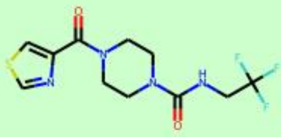

success

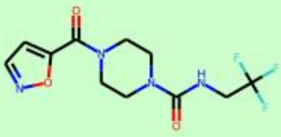

success

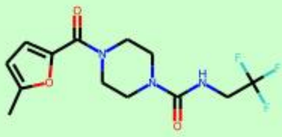

success

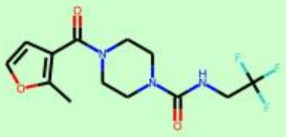

success

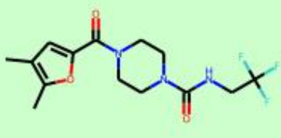

success

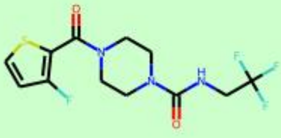

success

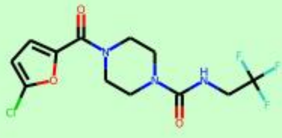

success

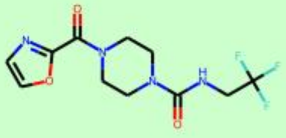

success

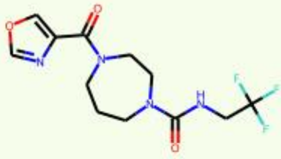

trace

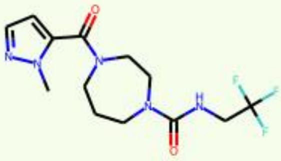

trace

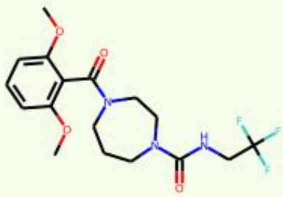

trace

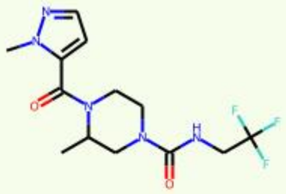

trace

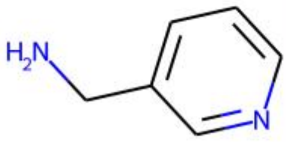

SM

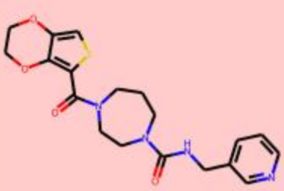

fail

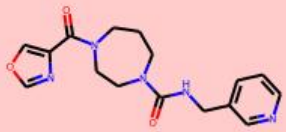

fail

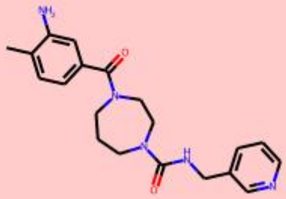

fail

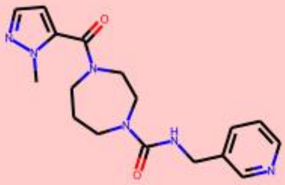

fail

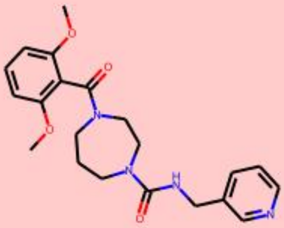

fail

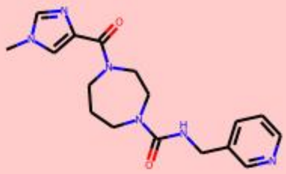

fail

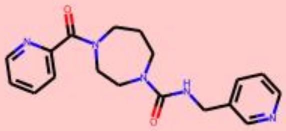

fail

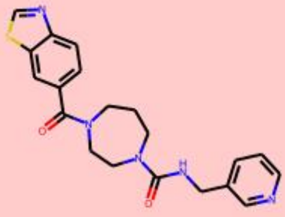

fail

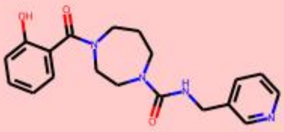

fail

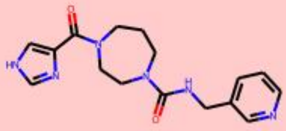

fail

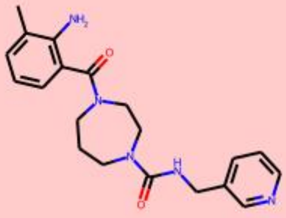

fail

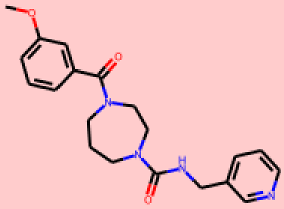

fail

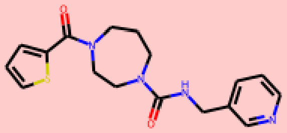

fail

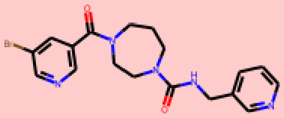

fail

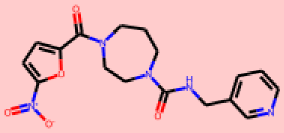

fail

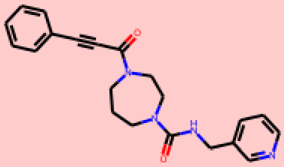

fail

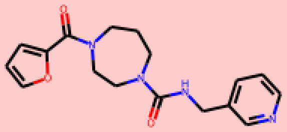

fail

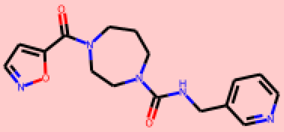

fail

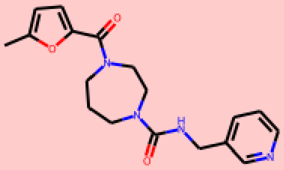

fail

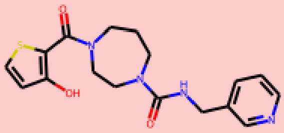

fail

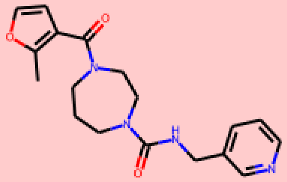

fail

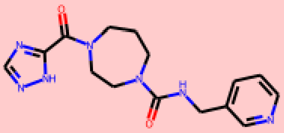

fail

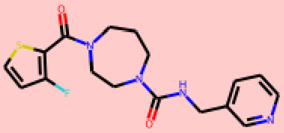

fail

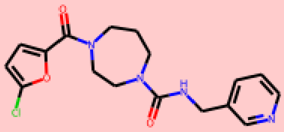

fail

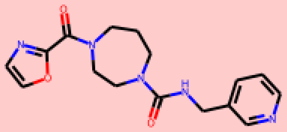

fail

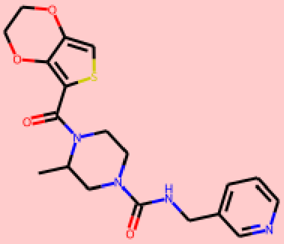

fail

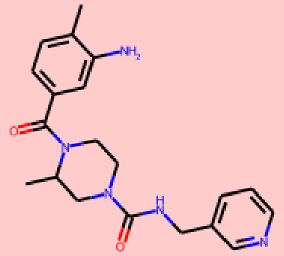

fail

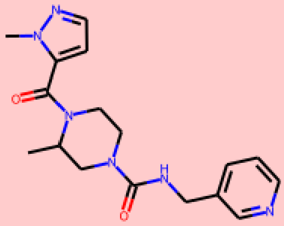

fail

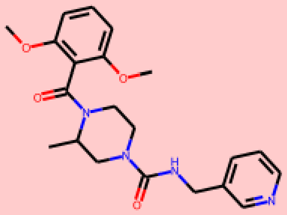

fail

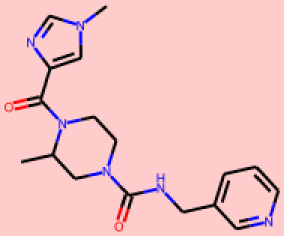

fail

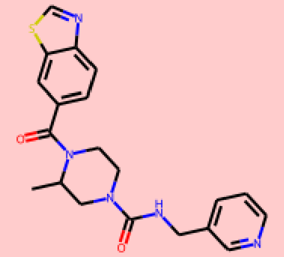

fail

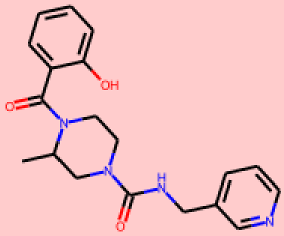

fail

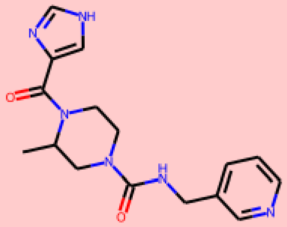

fail

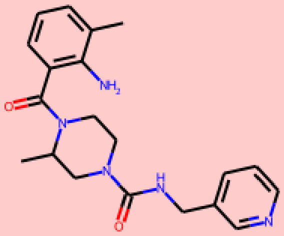

fail

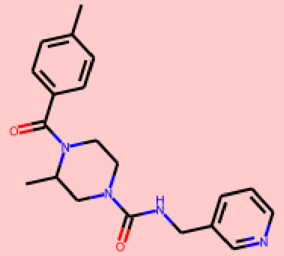

fail

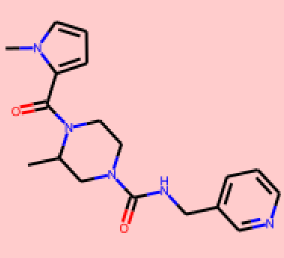
fail
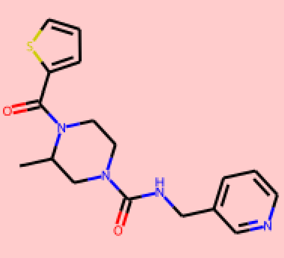
fail
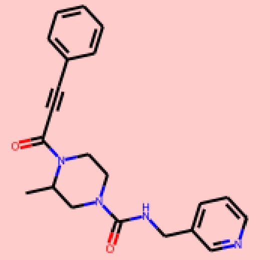
fail
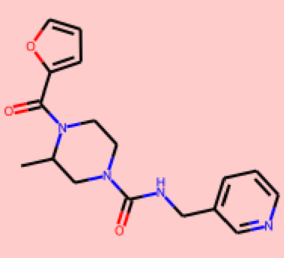
fail
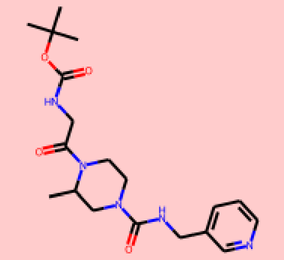
fail
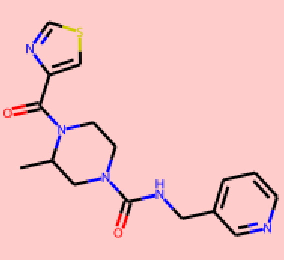
fail
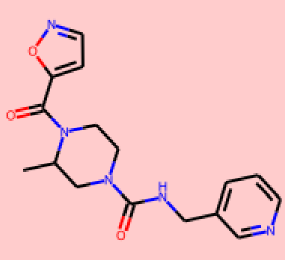
fail
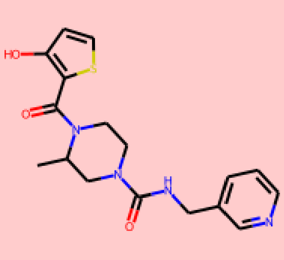
fail
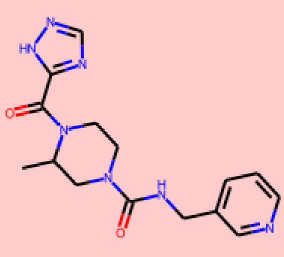
fail
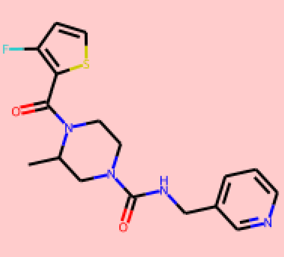
fail
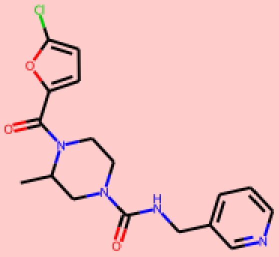
fail
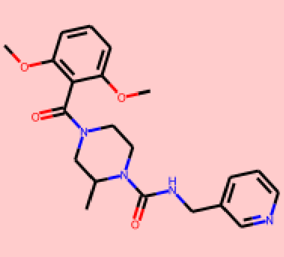
fail
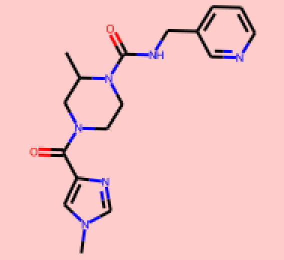
fail
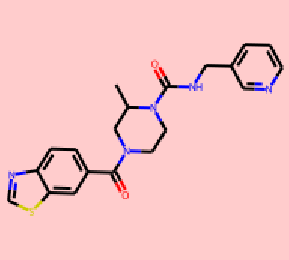
fail
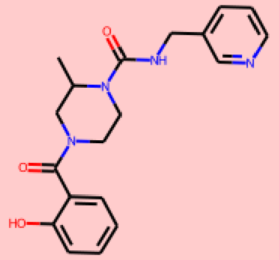
fail
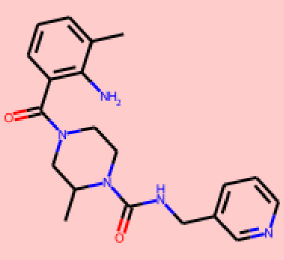
fail
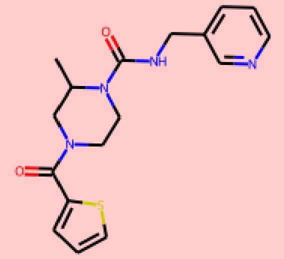
fail
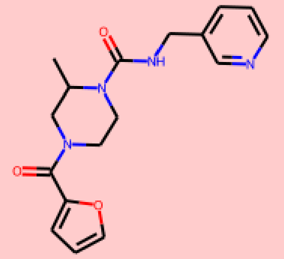
fail
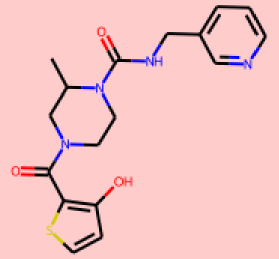
fail
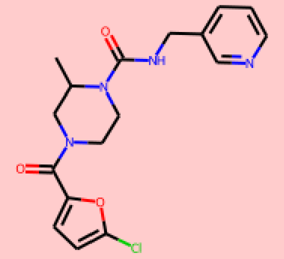
fail
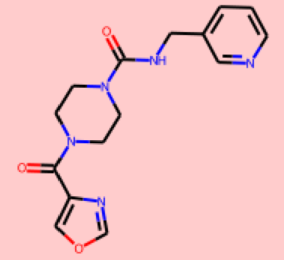
fail
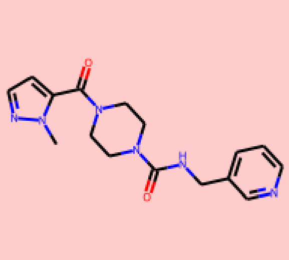
fail
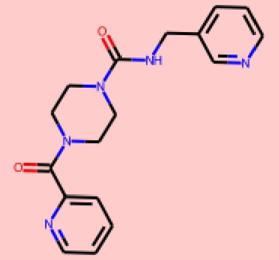
fail
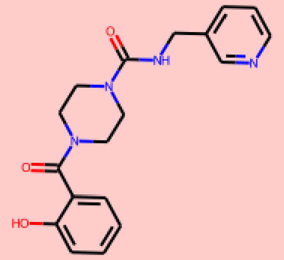
fail

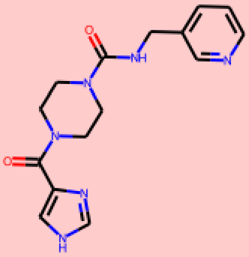
fail
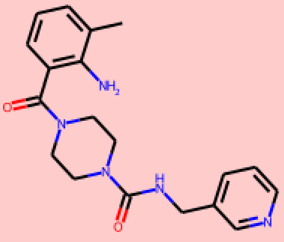
fail
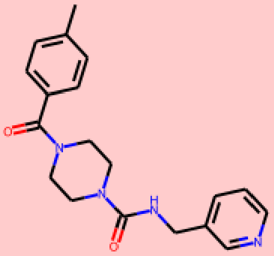
fail
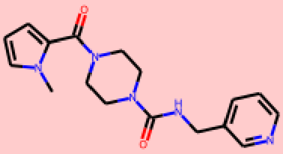
fail

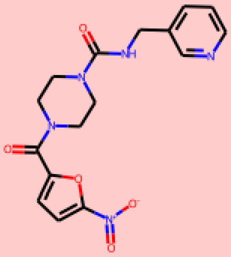
fail
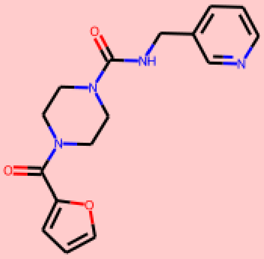
fail
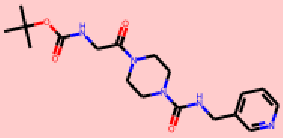
fail
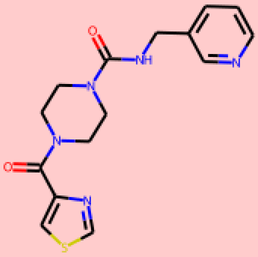
fail

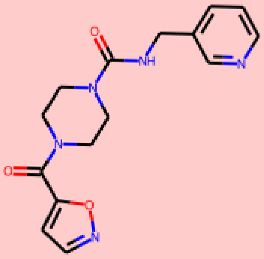
fail
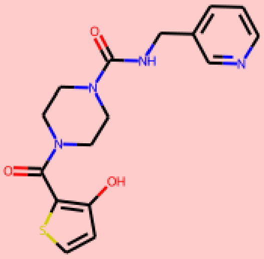
fail
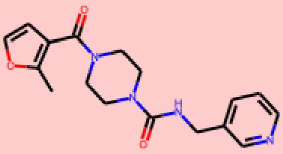
fail
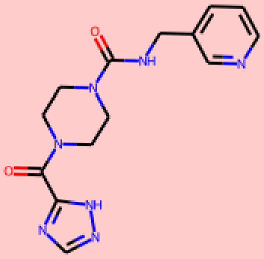
fail

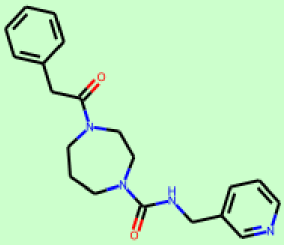
success
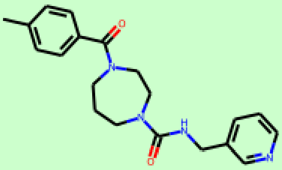
success
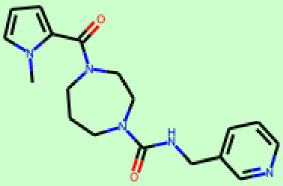
success
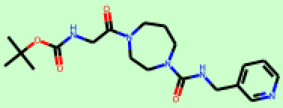
success

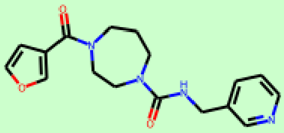
success
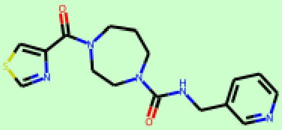
success
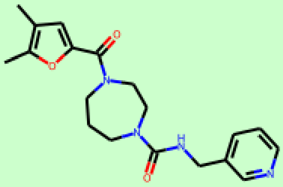
success
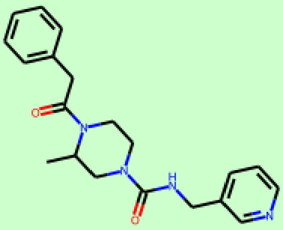
success

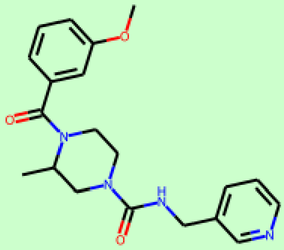
success
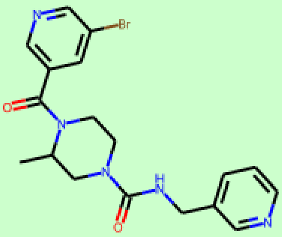
success
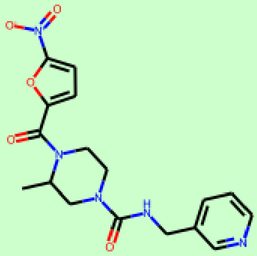
success
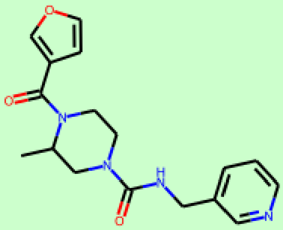
success

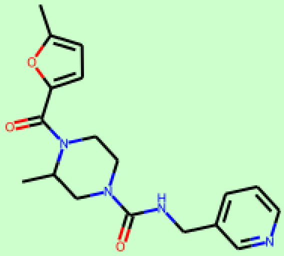

success

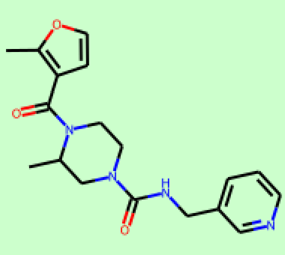

success

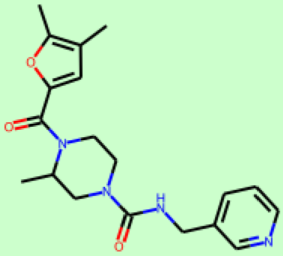

success

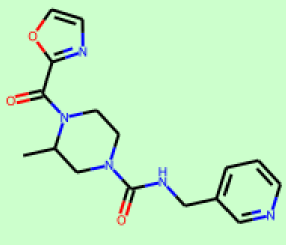

success

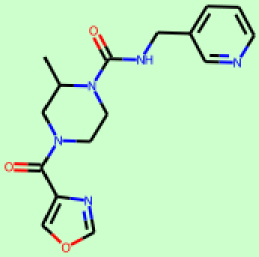

success

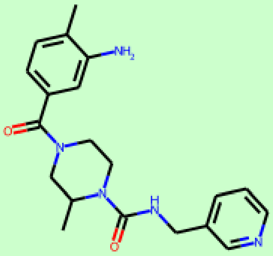

success

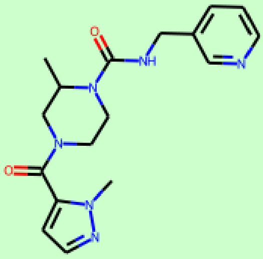

success

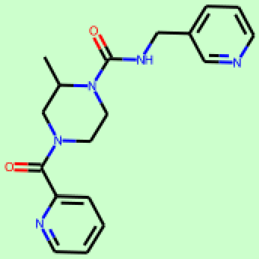

success

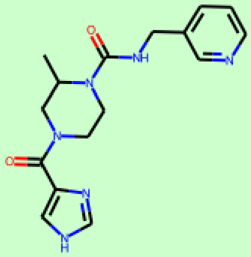

success

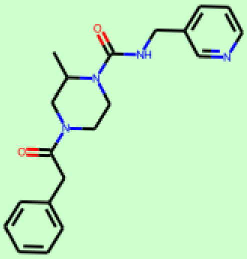

success

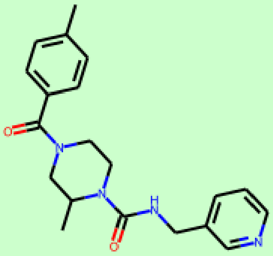

success

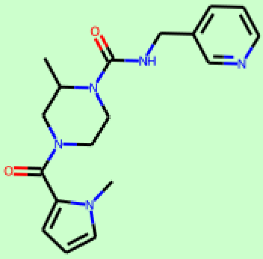

success

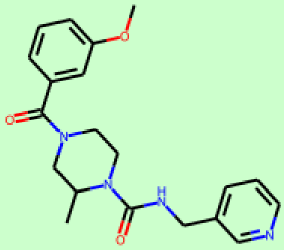

success

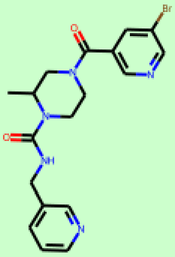

success

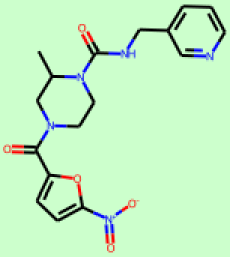

success

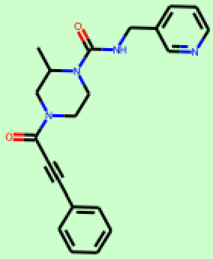

success

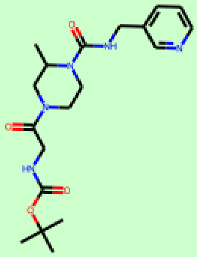

success

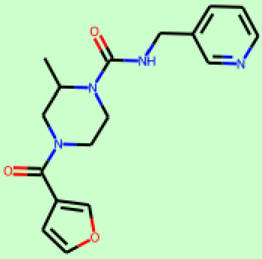

success

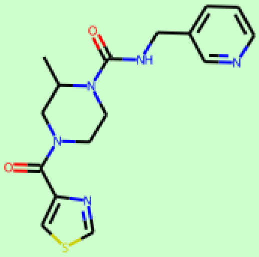

success

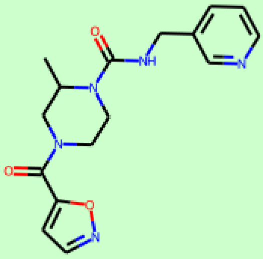

success

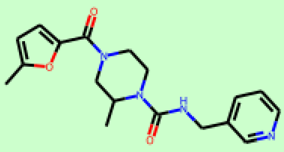

success

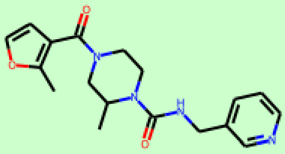

success

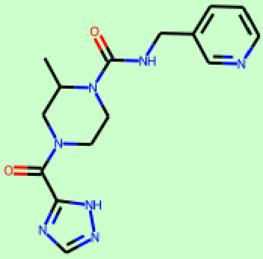

success

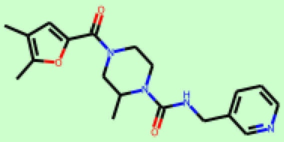

success

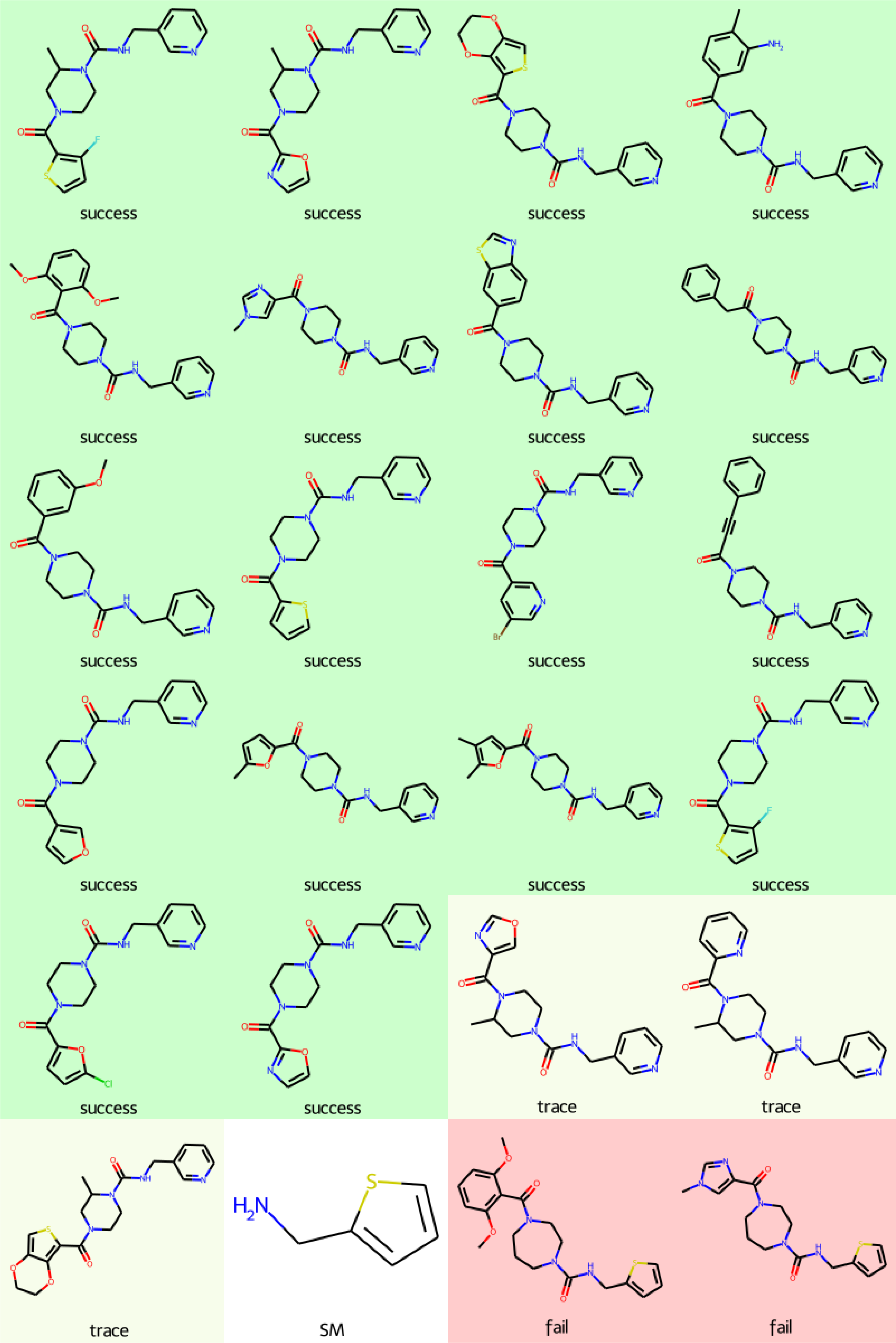
success
success
success
success
success
success
success
success
success
success
success
success
success
success
success
success
success
success
trace
trace
trace
H2N
SM
fail
fail

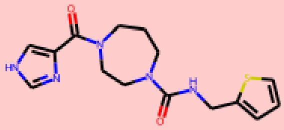

fail

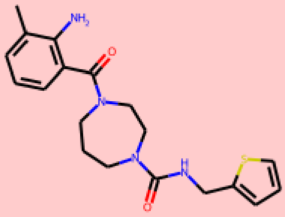

fail

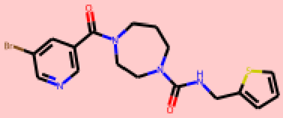

fail

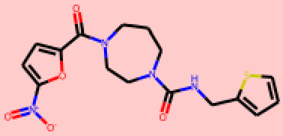

fail

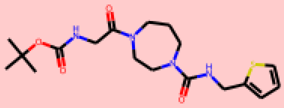

fail

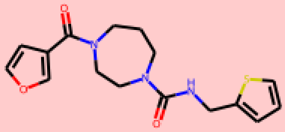

fail

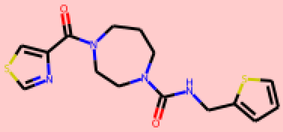

fail

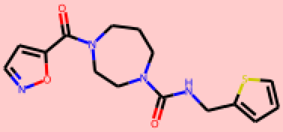

fail

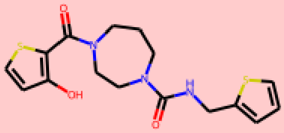

fail

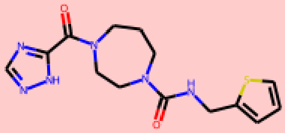

fail

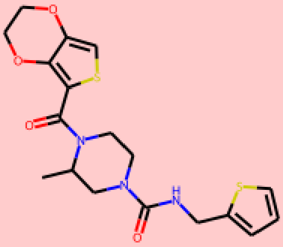

fail

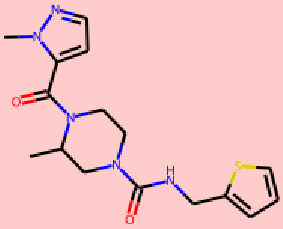

fail

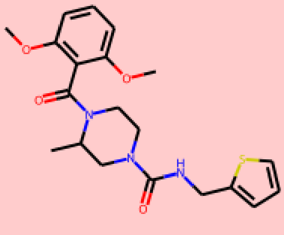

fail

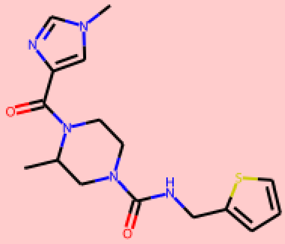

fail

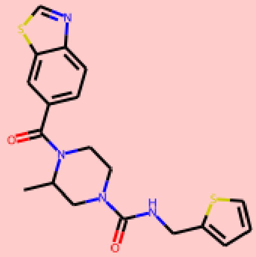

fail

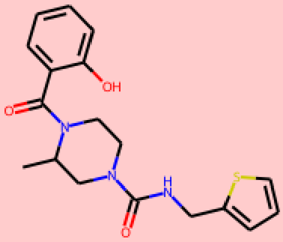

fail

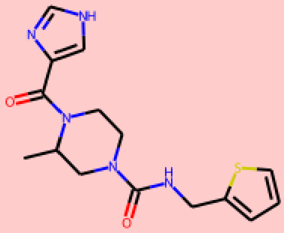

fail

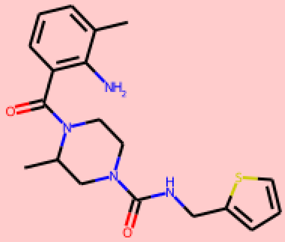

fail

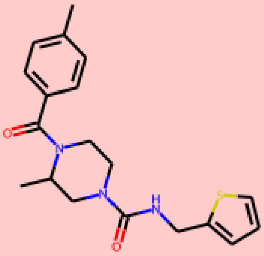

fail

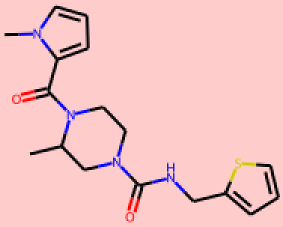

fail

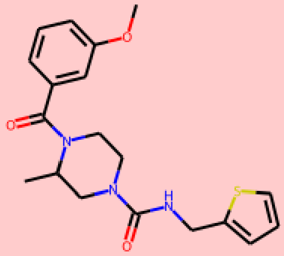

fail

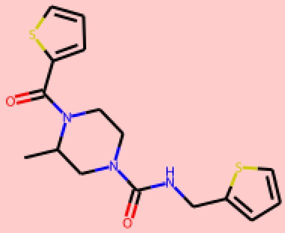

fail

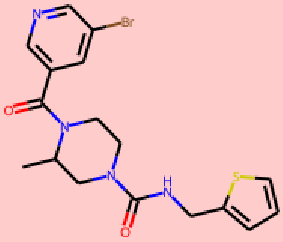

fail

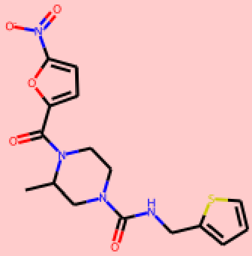

fail

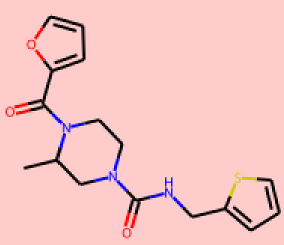
fail

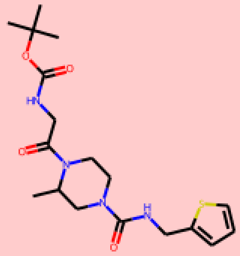
fail

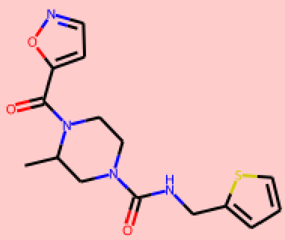
fail

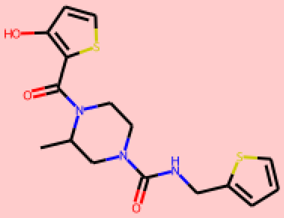
fail

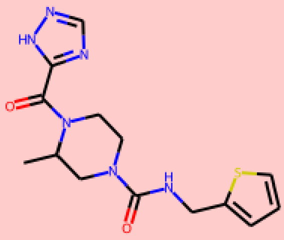
fail

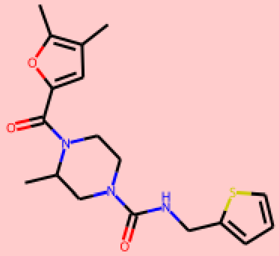
fail

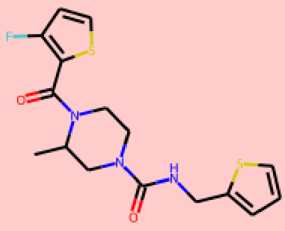
fail

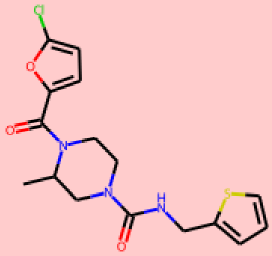
fail

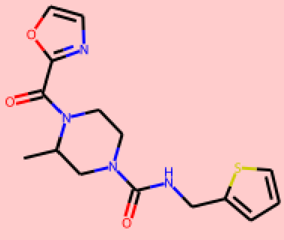
fail

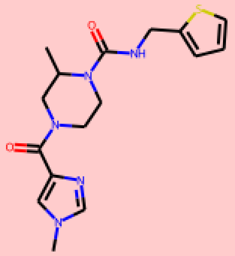
fail

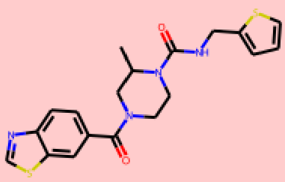
fail

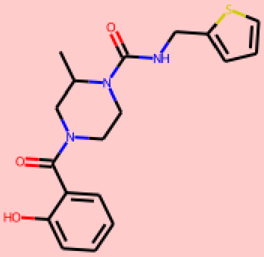
fail

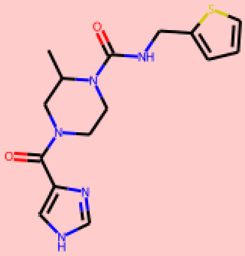
fail

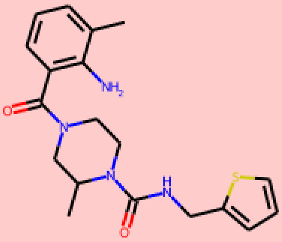
fail

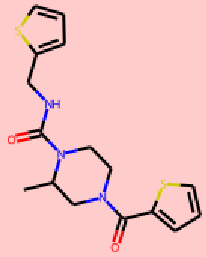
fail

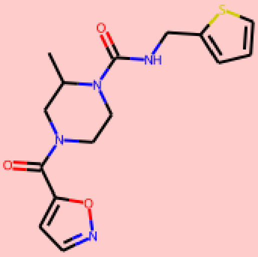
fail

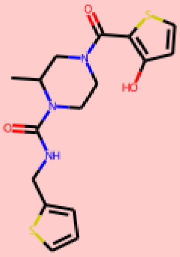
fail

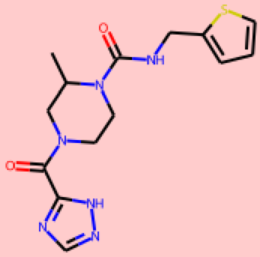
fail

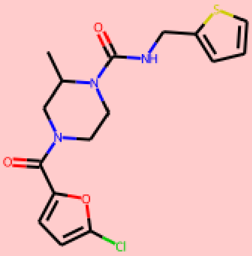
fail

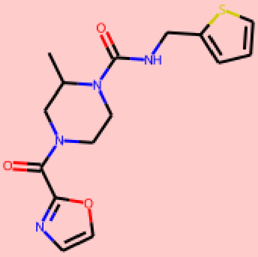
fail

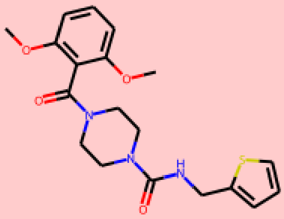
fail

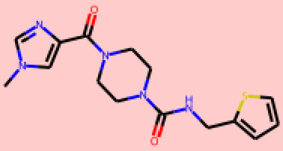
fail

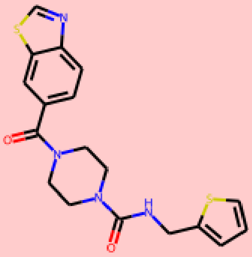
fail

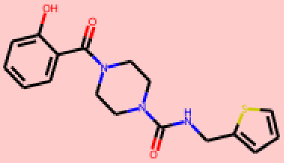
fail

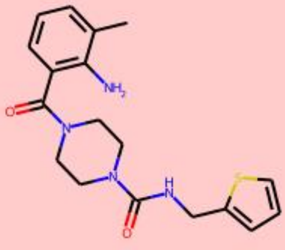

fail

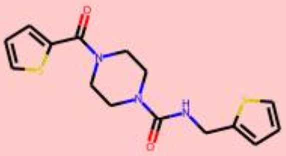

fail

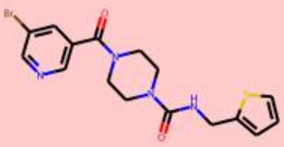

fail

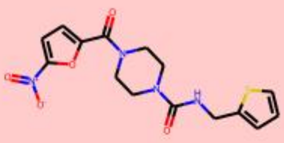

fail

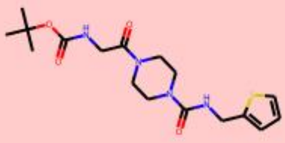

fail

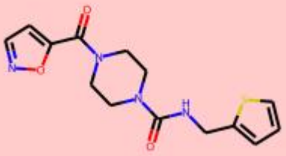

fail

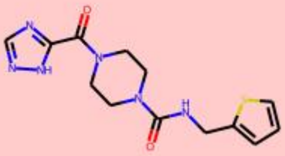

fail

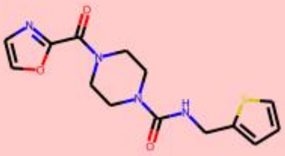

fail

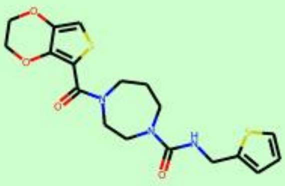

success

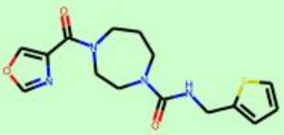

success

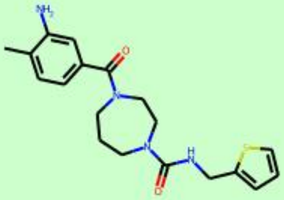

success

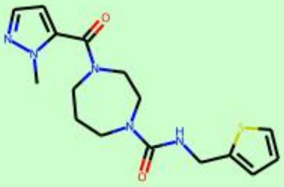

success

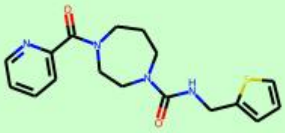

success

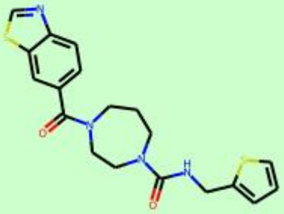

success

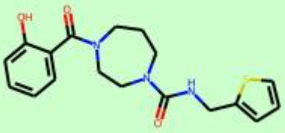

success

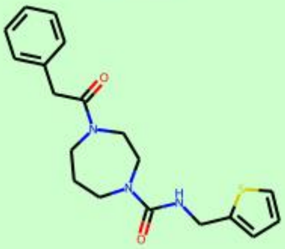

success

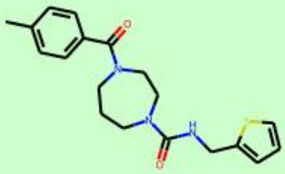

success

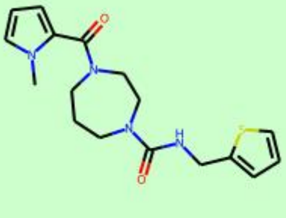

success

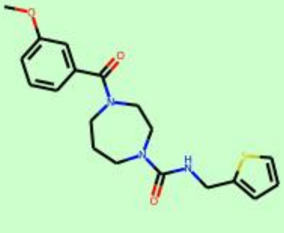

success

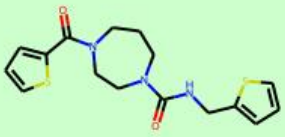

success

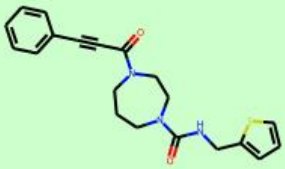

success

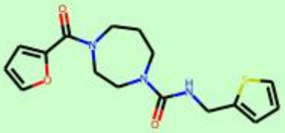

success

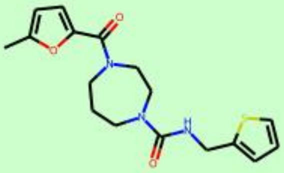

success

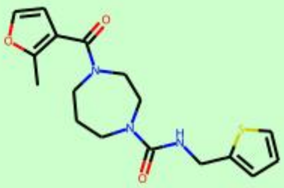

success

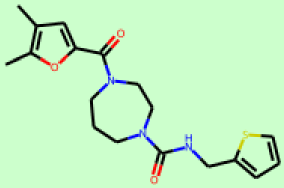

success

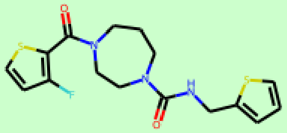

success

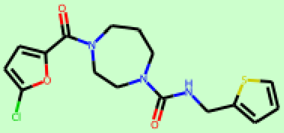

success

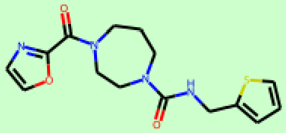

success

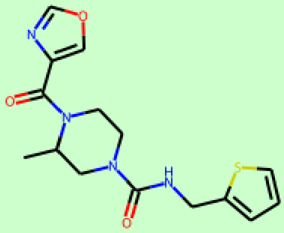

success

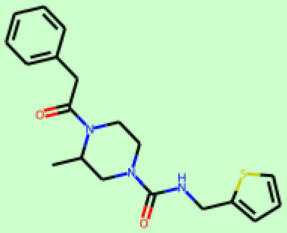

success

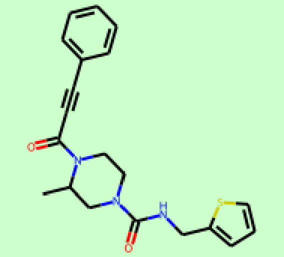

success

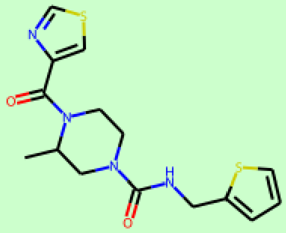

success

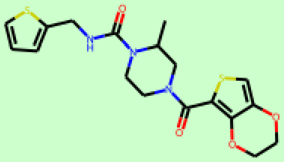

success

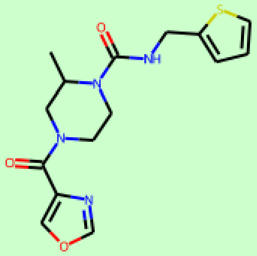

success

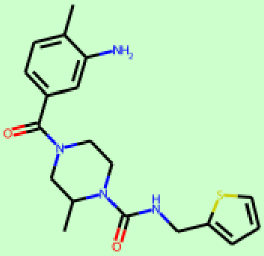

success

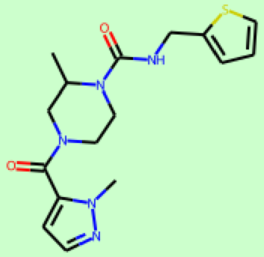

success

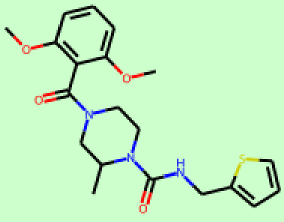

success

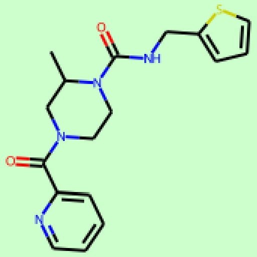

success

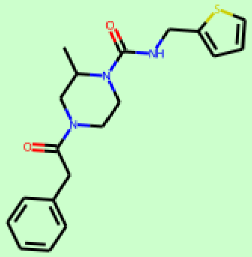

success

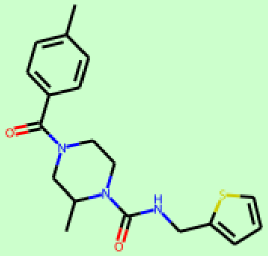

success

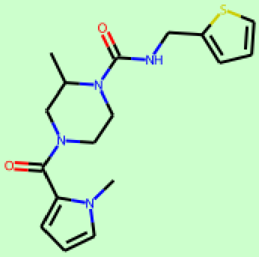

success

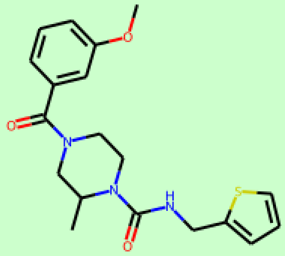

success

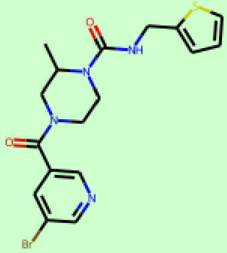

success

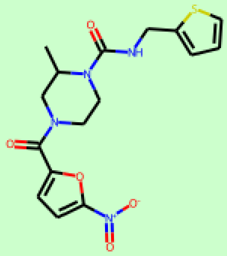

success

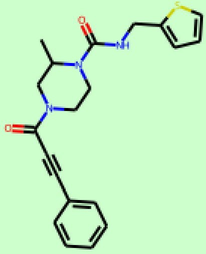

success

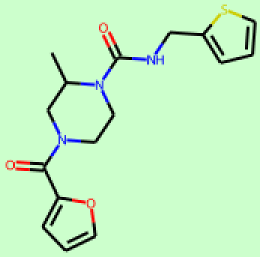

success

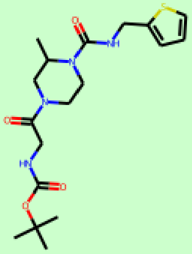

success

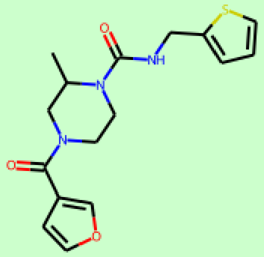

success

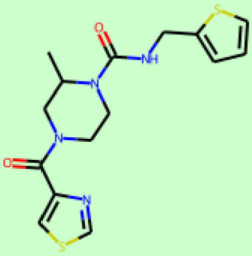

success

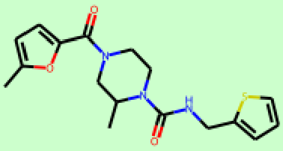

success

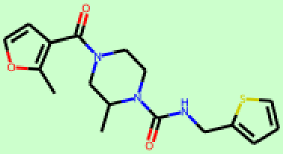

success

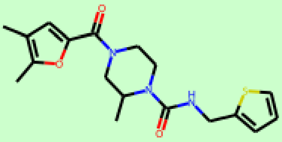

success

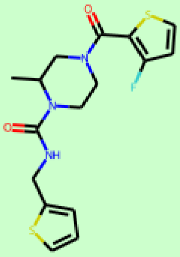

success

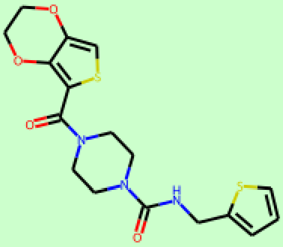

success

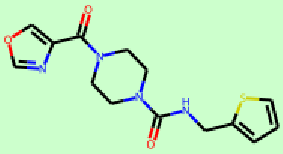

success

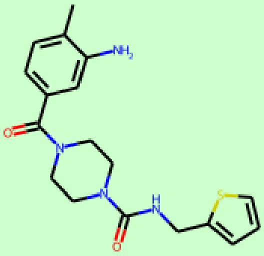

success

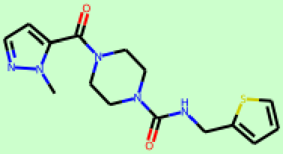

success

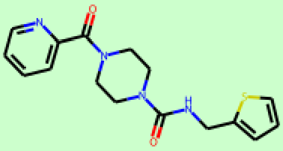

success

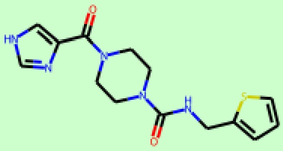

success

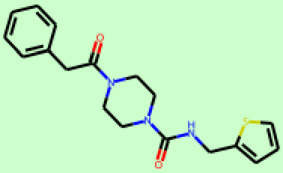

success

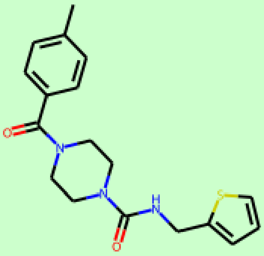

success

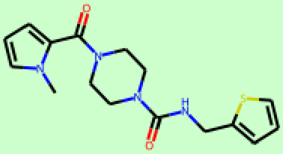

success

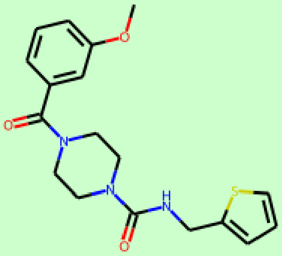

success

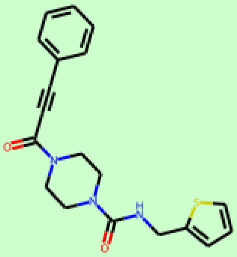

success

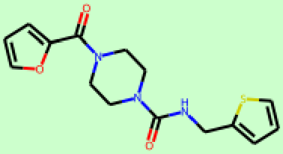

success

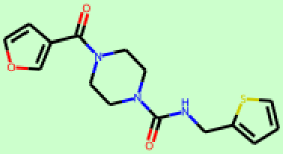

success

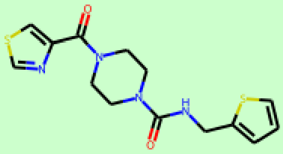

success

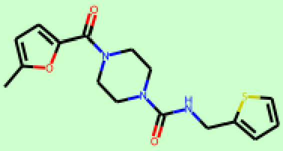

success

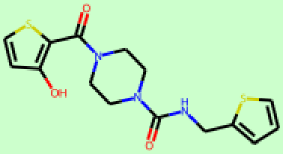

success

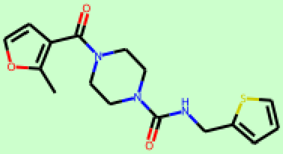

success

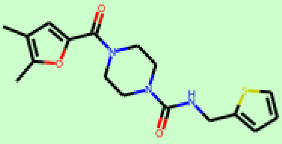

success

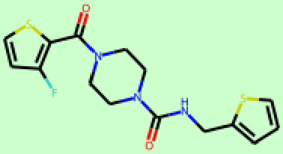

success

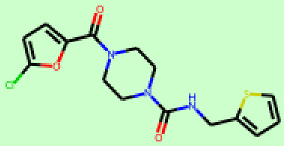

success

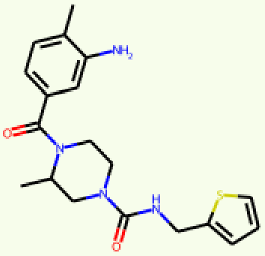

trace

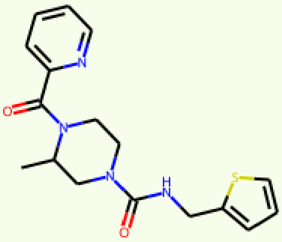

trace

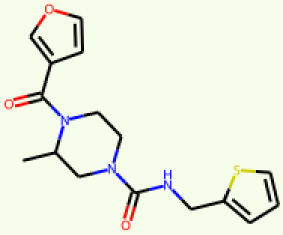

trace

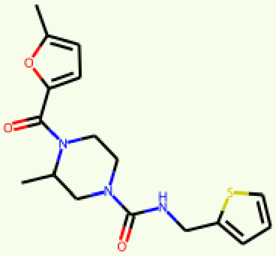

trace

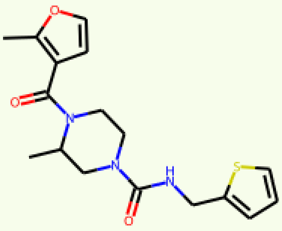

trace

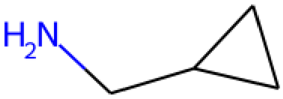

SM

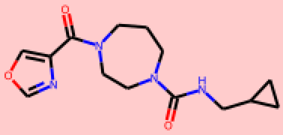

fail

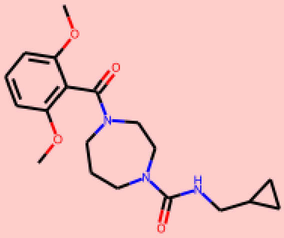

fail

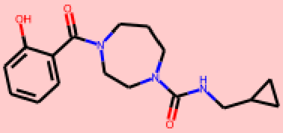

fail

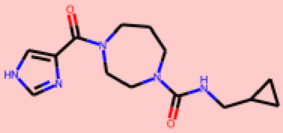

fail

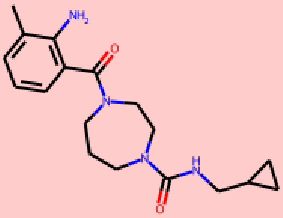

fail

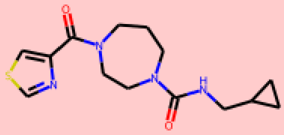

fail

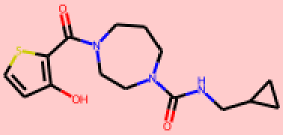

fail

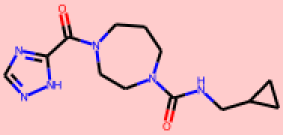

fail

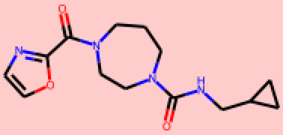

fail

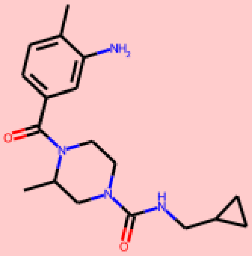

fail

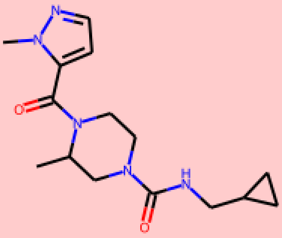

fail

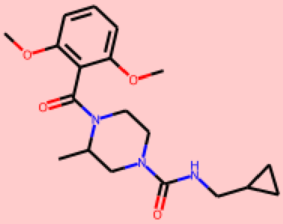

fail

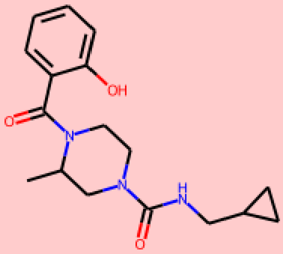

fail

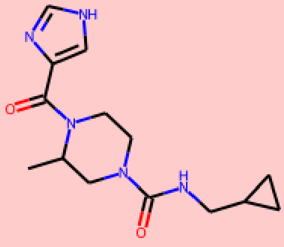

fail

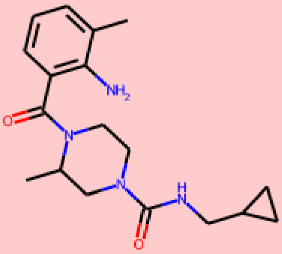

fail

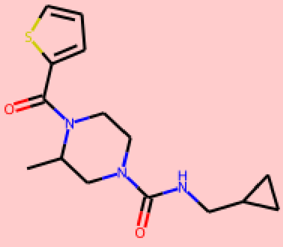

fail

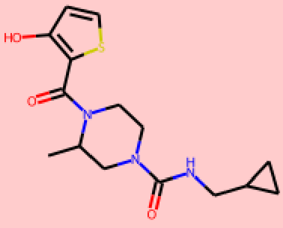

fail

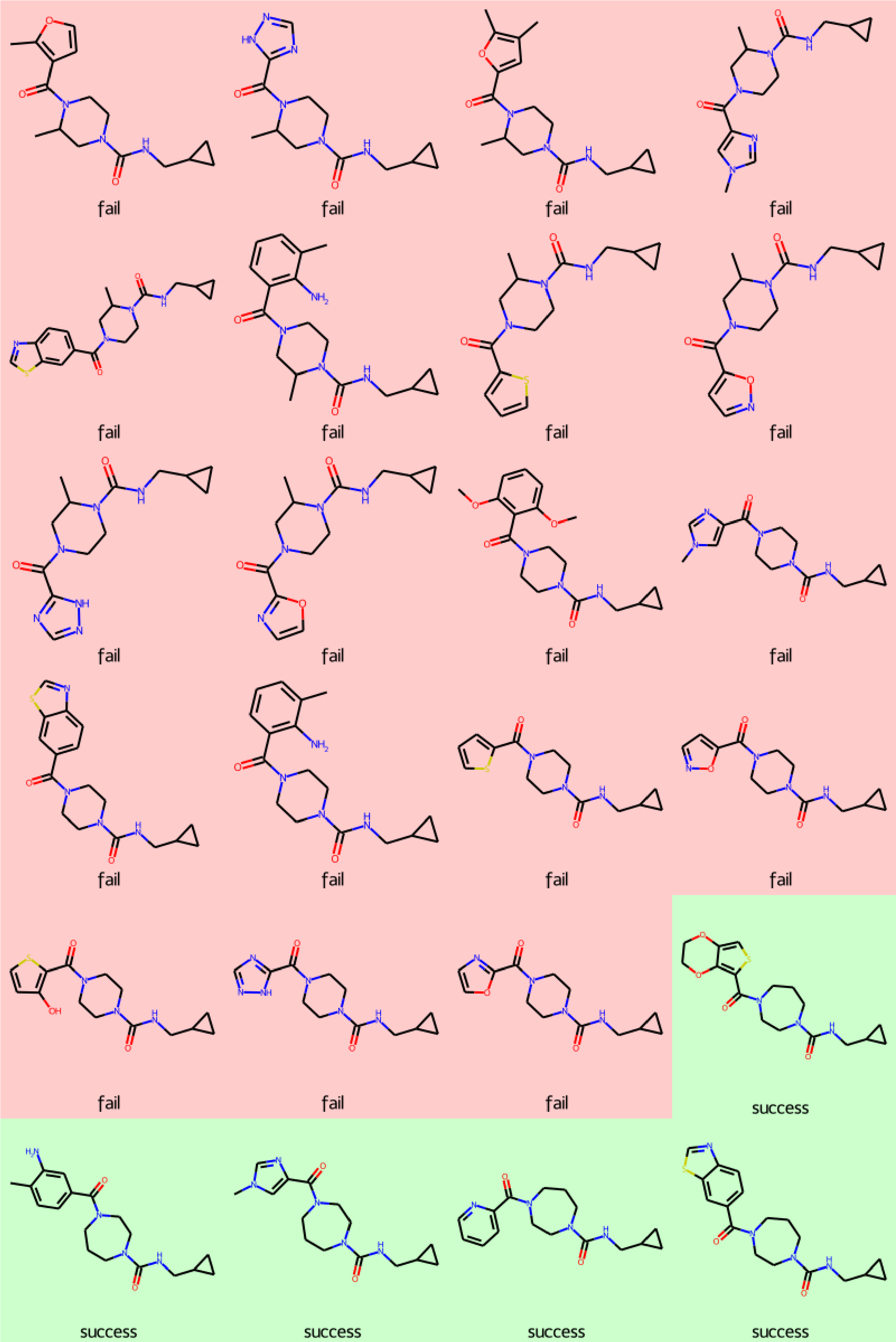
fail
fail
fail
fail
fail
fail
fail
fail
fail
fail
fail
fail
fail
fail
fail
fail
fail
fail
fail
success
success
success
success
success

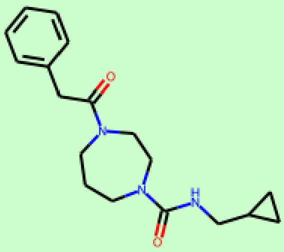

success

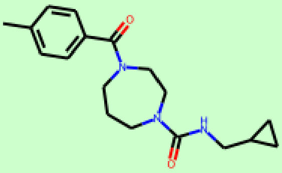

success

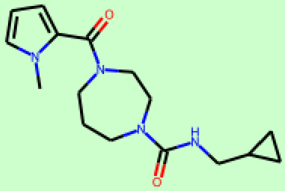

success

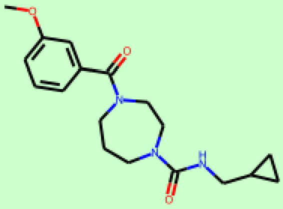

success

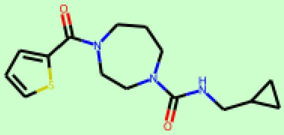

success

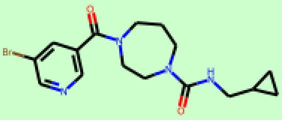

success

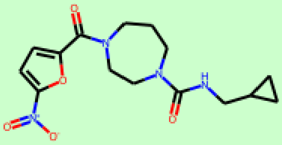

success

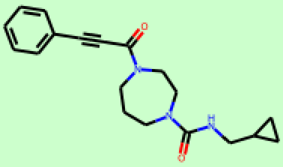

success

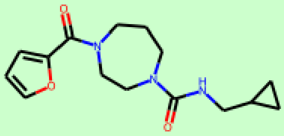

success

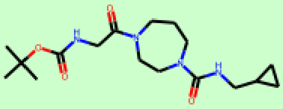

success

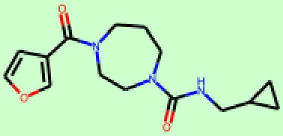

success

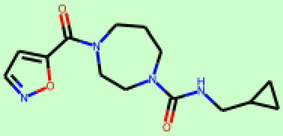

success

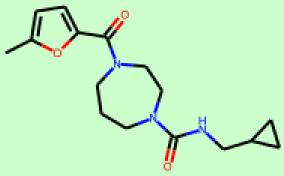

success

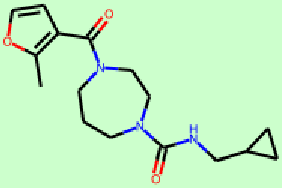

success

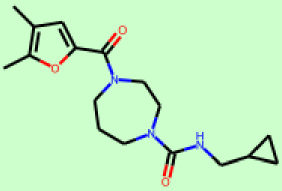

success

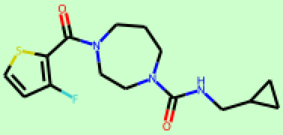

success

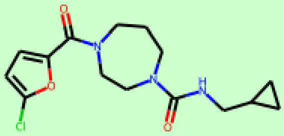

success

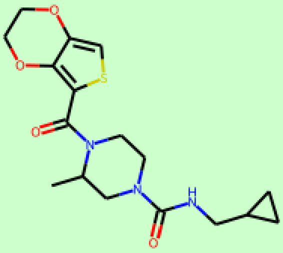

success

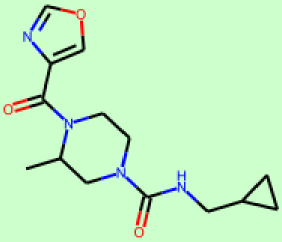

success

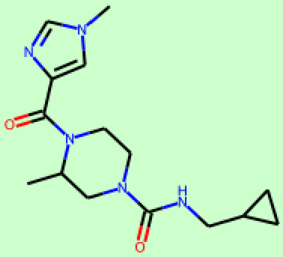

success

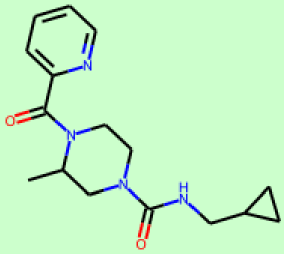

success

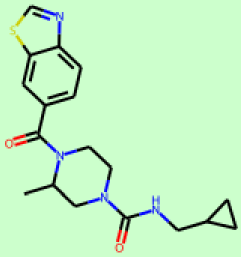

success

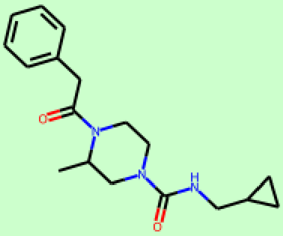

success

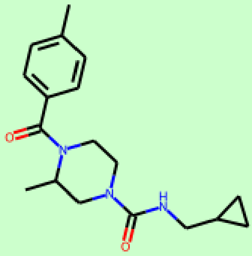

success

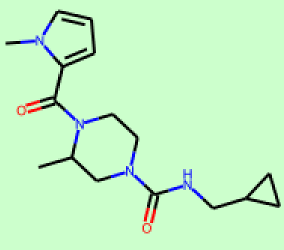
success

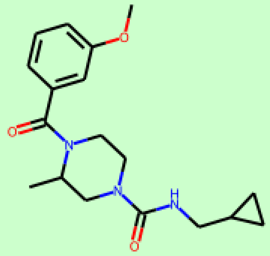
success

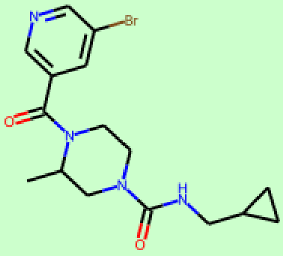
success

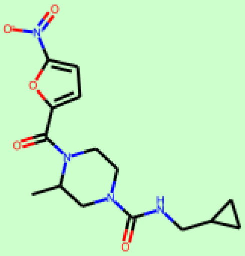
success

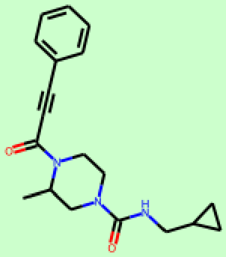
success

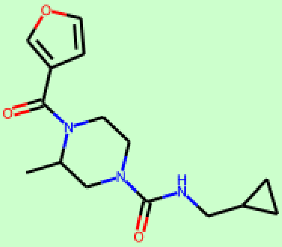
success

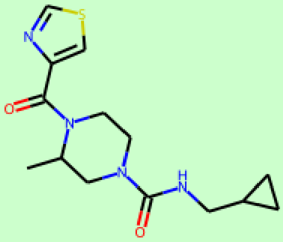
success

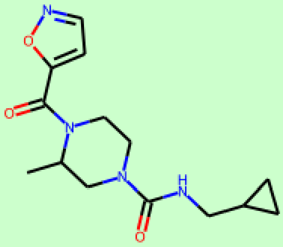
success

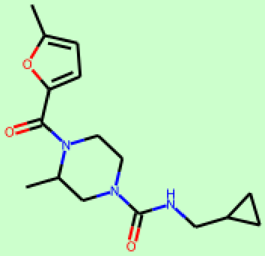
success

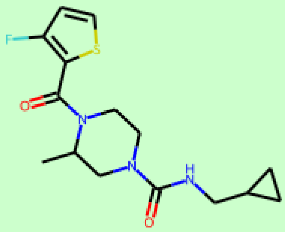
success

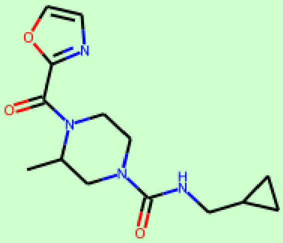
success

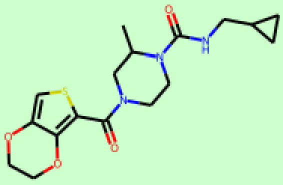
success

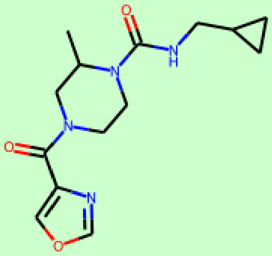
success

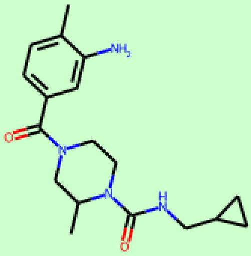
success

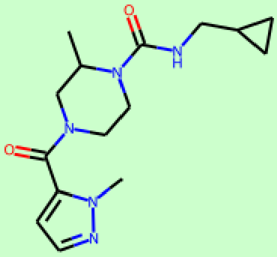
success

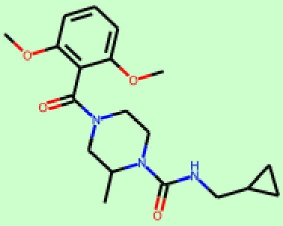
success

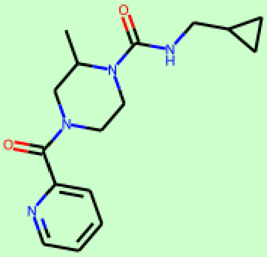
success

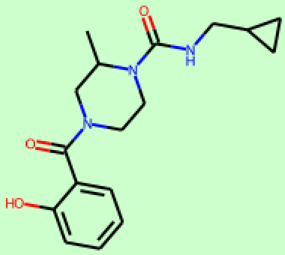
success

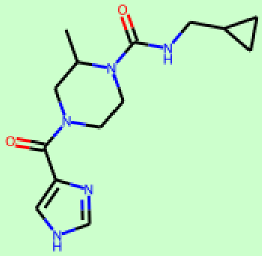
success

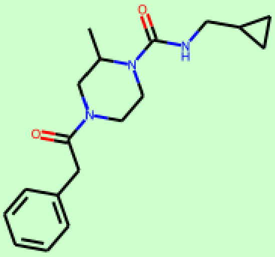
success

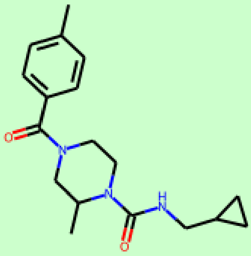
success

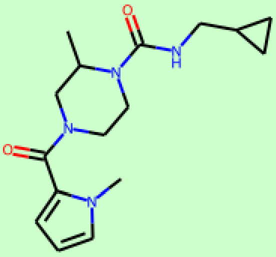
success

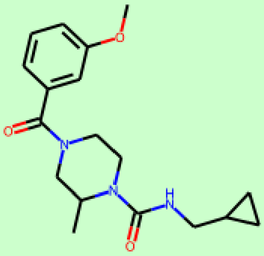
success

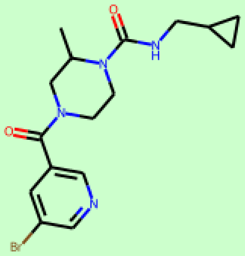
success

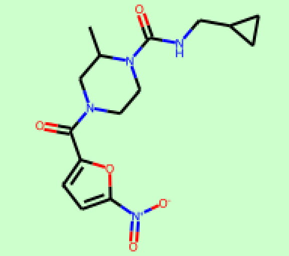
success
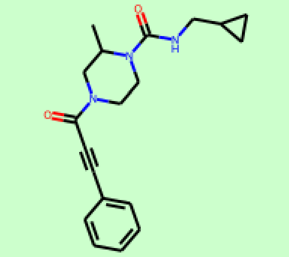
success
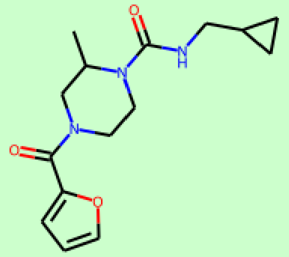
success
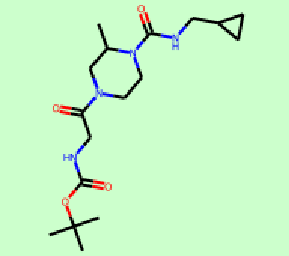
success
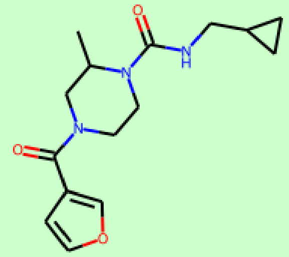
success
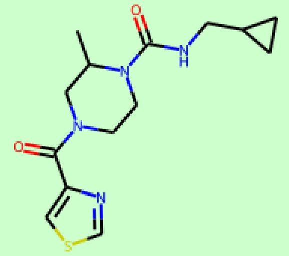
success
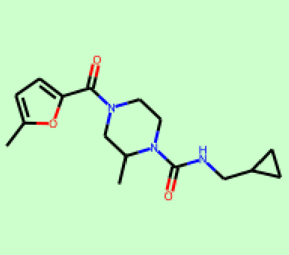
success
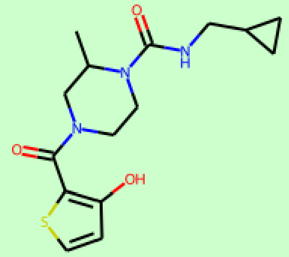
success
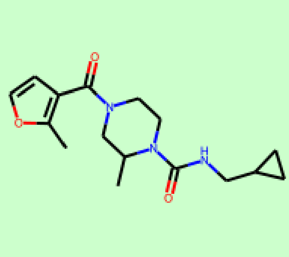
success
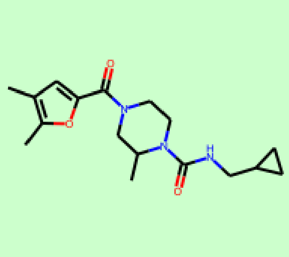
success
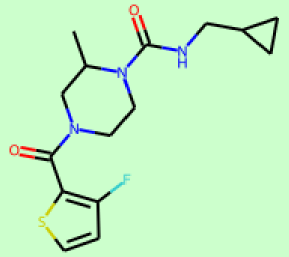
success
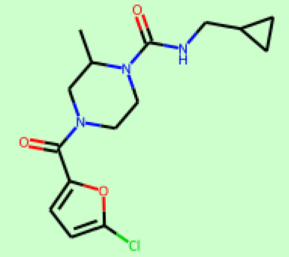
success
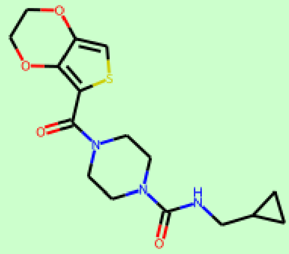
success
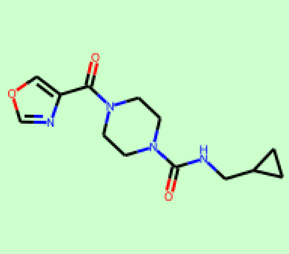
success
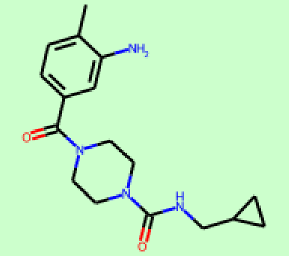
success
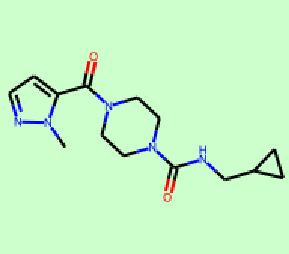
success
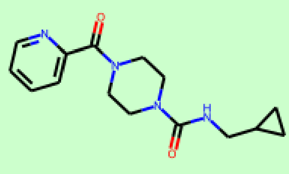
success
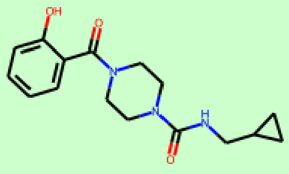
success
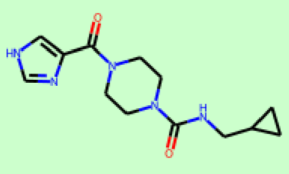
success
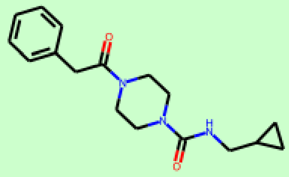
success
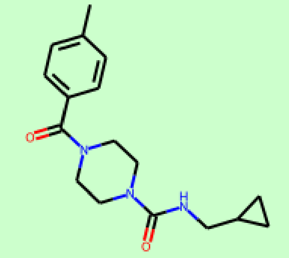
success
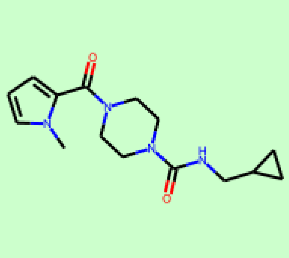
success
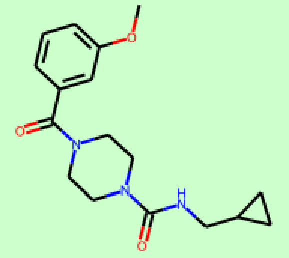
success
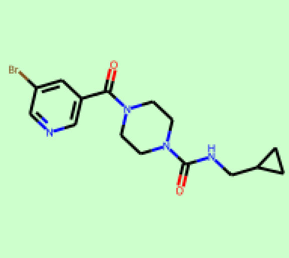
success

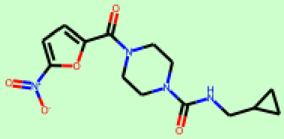
success
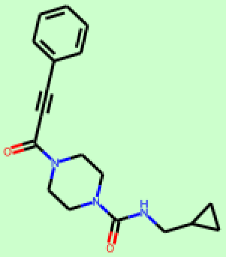
success
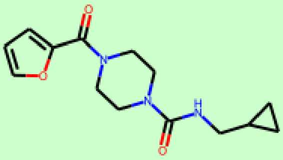
success
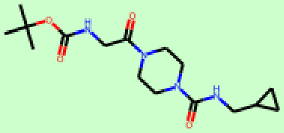
success
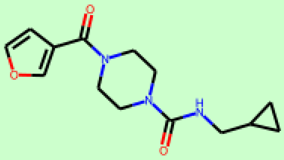
success
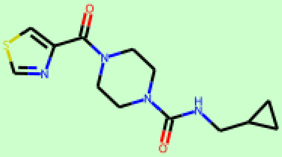
success
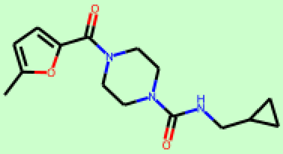
success
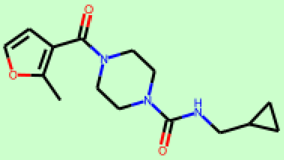
success
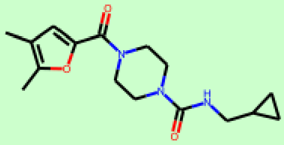
success
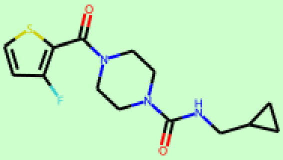
success
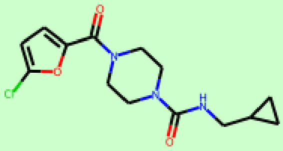
success
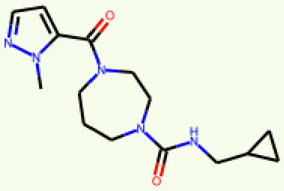
trace
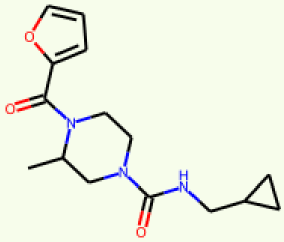
trace
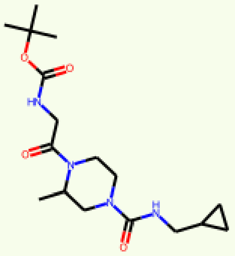
trace
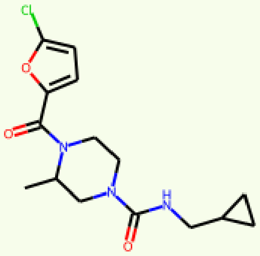
trace
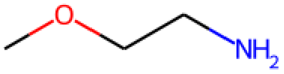
SM
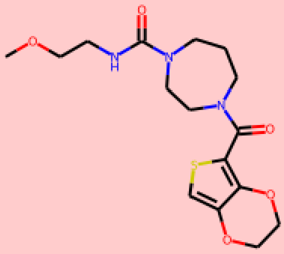
fail
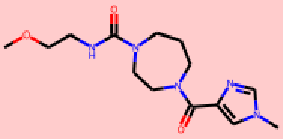
fail
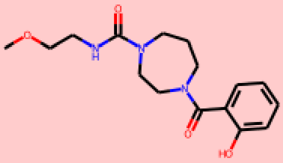
fail
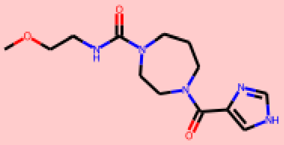
fail
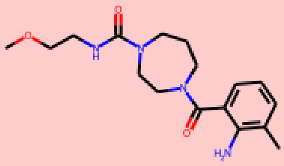
fail
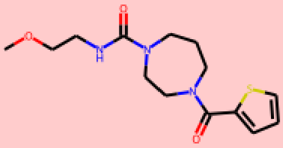
fail
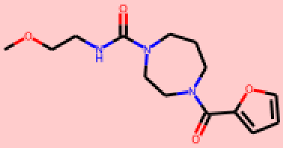
fail
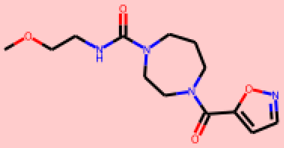
fail

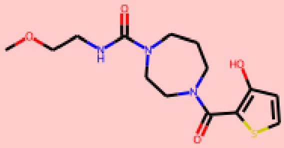
fail
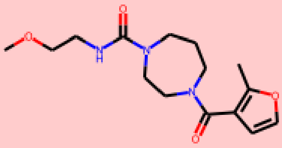
fail
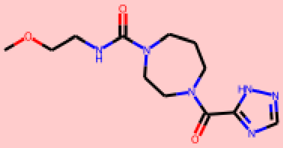
fail
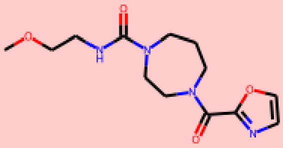
fail
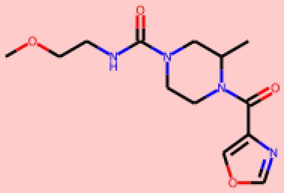
fail
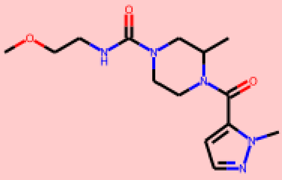
fail
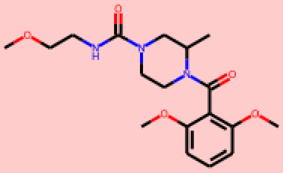
fail
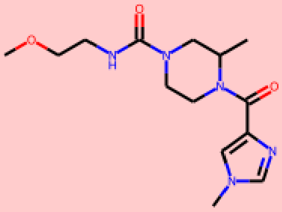
fail
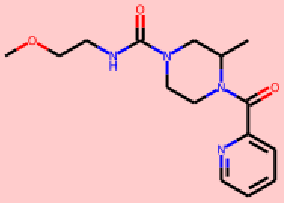
fail
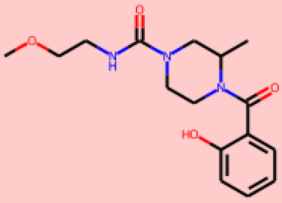
fail
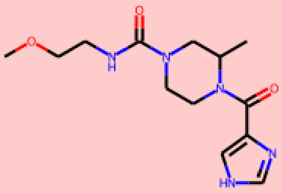
fail
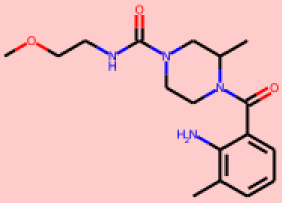
fail
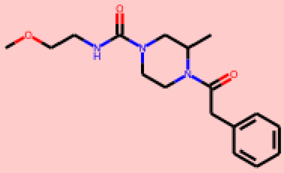
fail
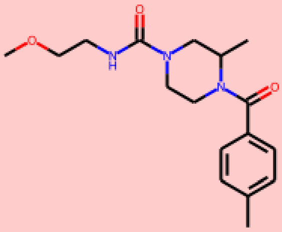
fail
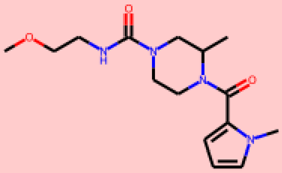
fail
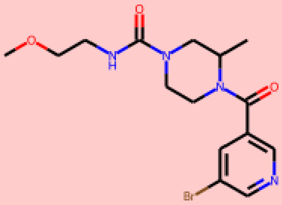
fail
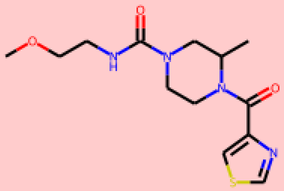
fail
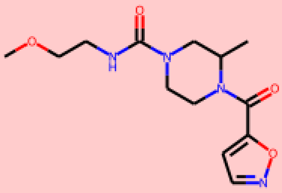
fail
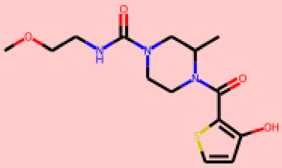
fail
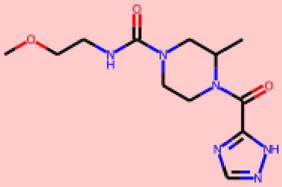
fail
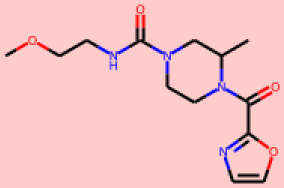
fail
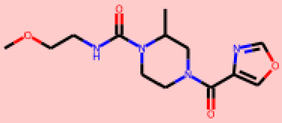
fail
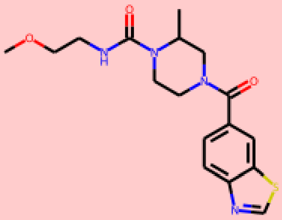
fail
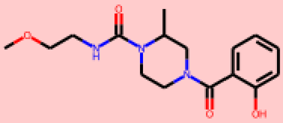
fail

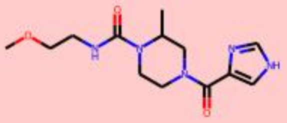

fail

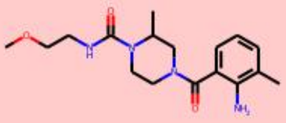

fail

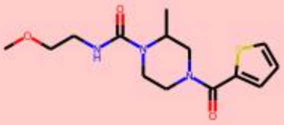

fail

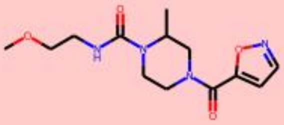

fail

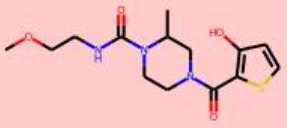

fail

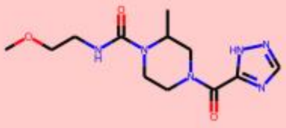

fail

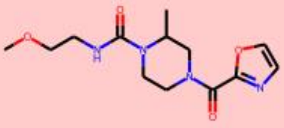

fail

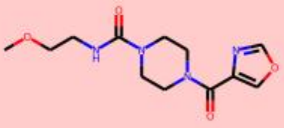

fail

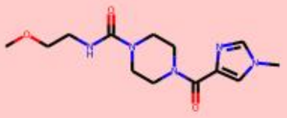

fail

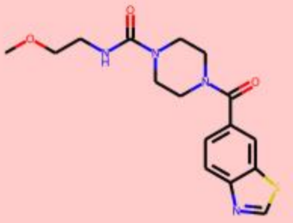

fail

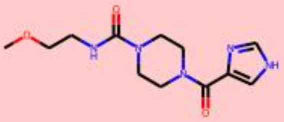

fail

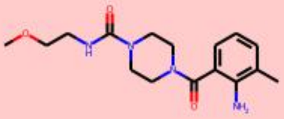

fail

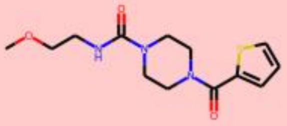

fail

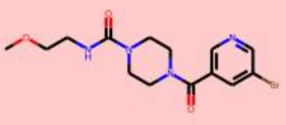

fail

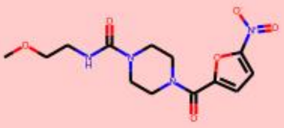

fail

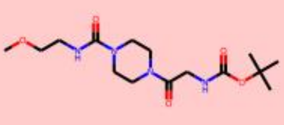

fail

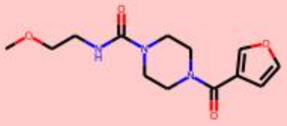

fail

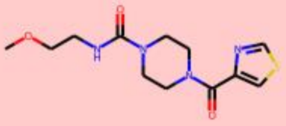

fail

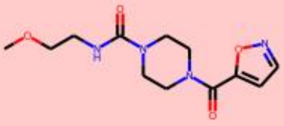

fail

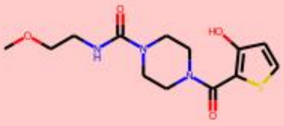

fail

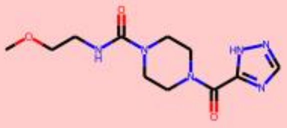

fail

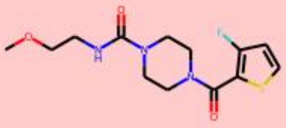

fail

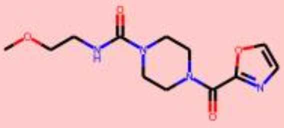

fail

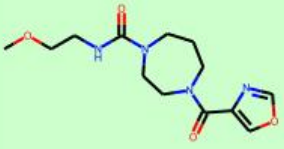

success

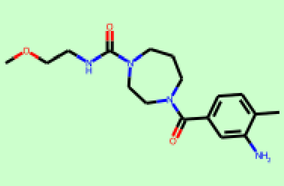

success

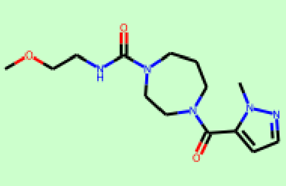

success

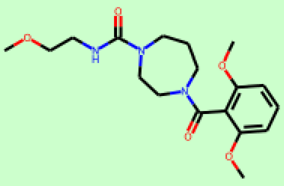

success

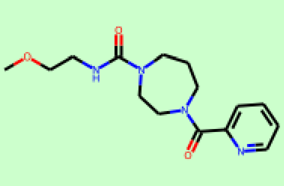

success

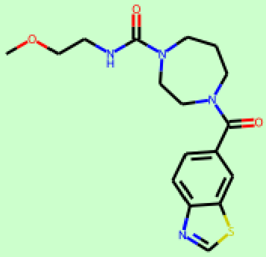

success

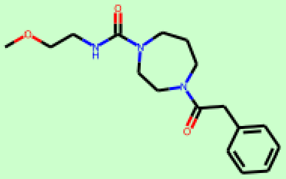

success

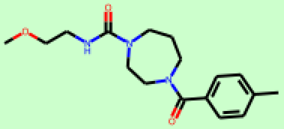

success

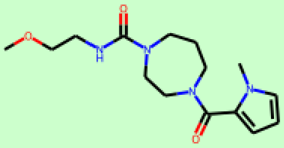

success

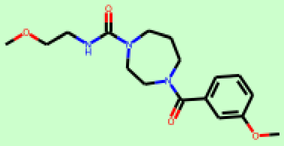

success

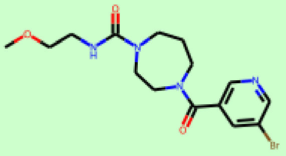

success

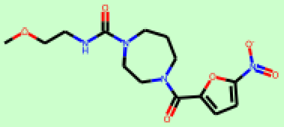

success

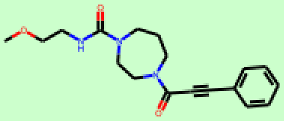

success

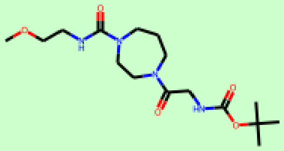

success

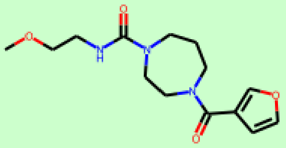

success

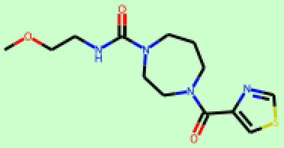

success

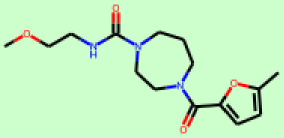

success

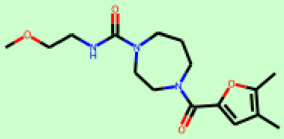

success

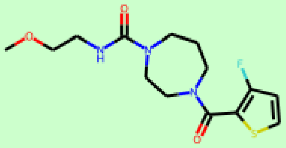

success

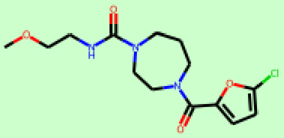

success

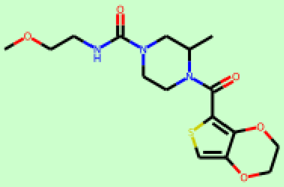

success

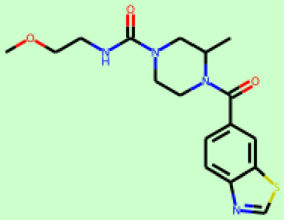

success

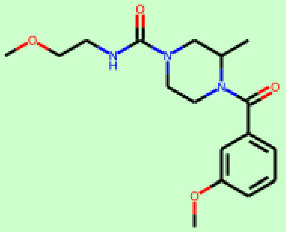

success

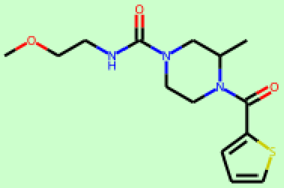

success

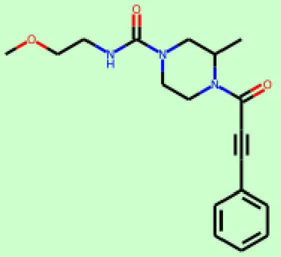

success

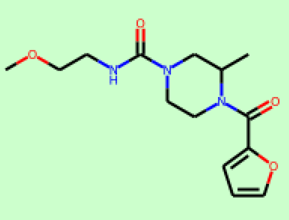

success

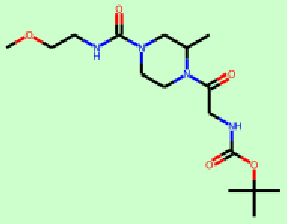

success

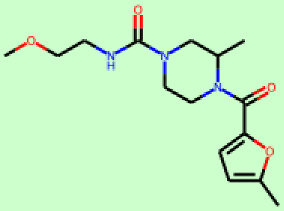

success

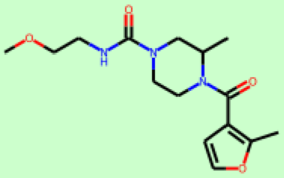

success

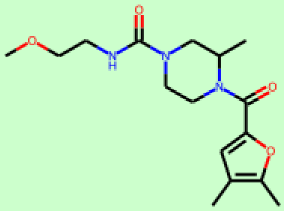

success

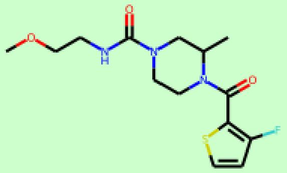

success

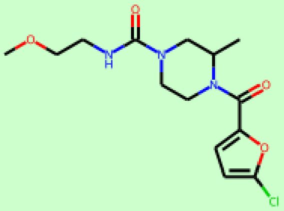

success

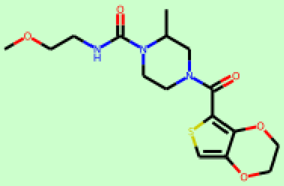

success

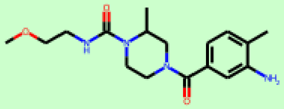

success

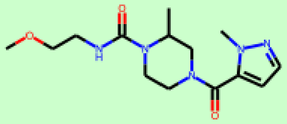

success

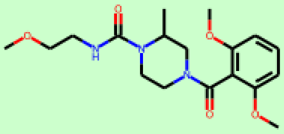

success

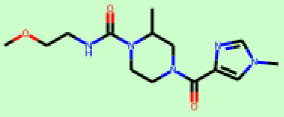

success

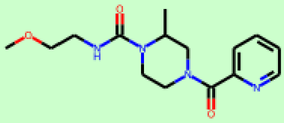

success

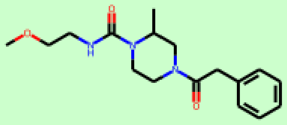

success

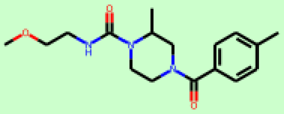

success

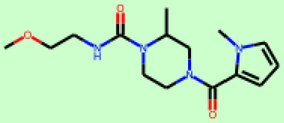

success

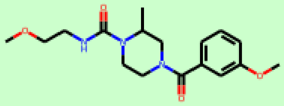

success

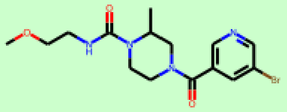

success

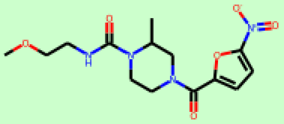

success

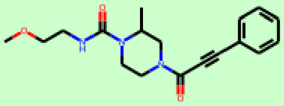

success

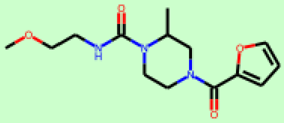

success

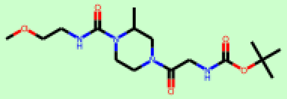

success

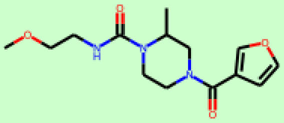

success

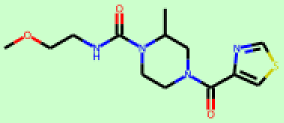

success

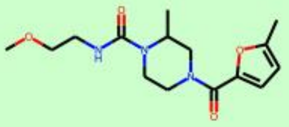

success

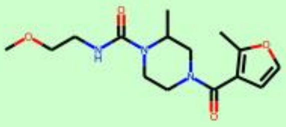

success

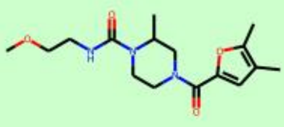

success

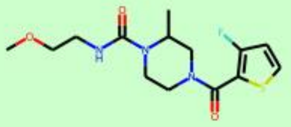

success

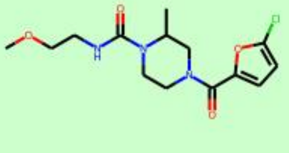

success

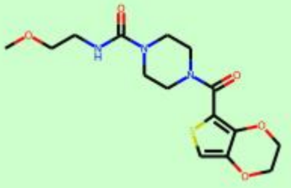

success

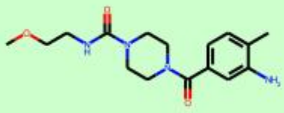

success

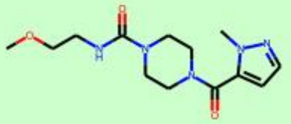

success

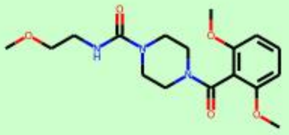

success

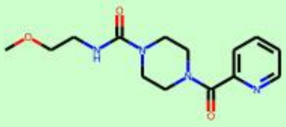

success

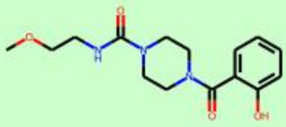

success

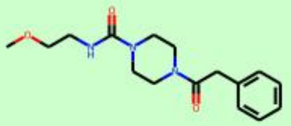

success

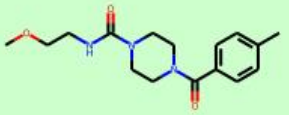

success

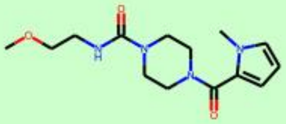

success

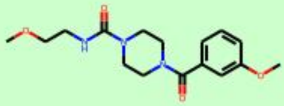

success

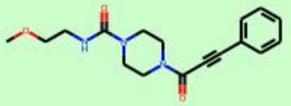

success

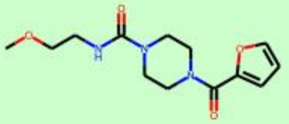

success

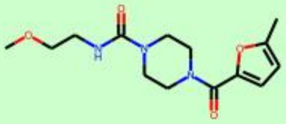

success

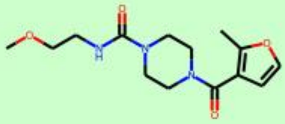

success

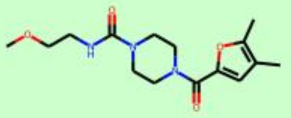

success

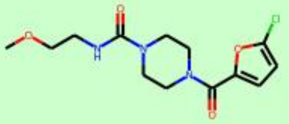

success

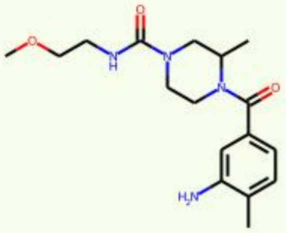

trace

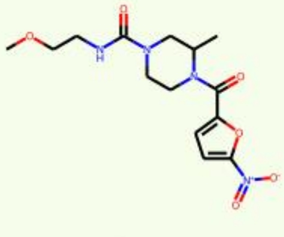

trace

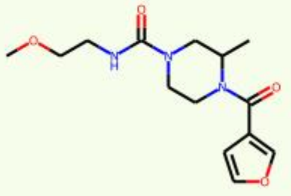

trace

8.6 Iteration 4.2

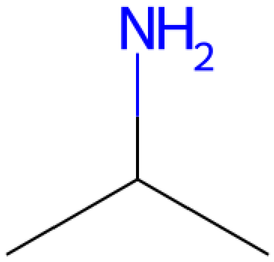
SM

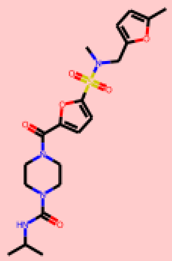
fail

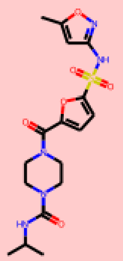
fail

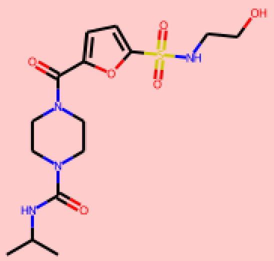
fail

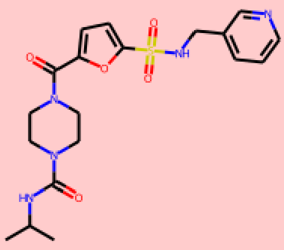
fail

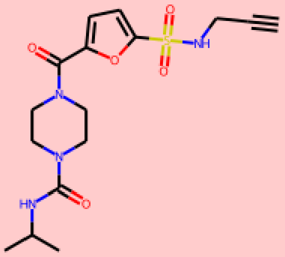
fail

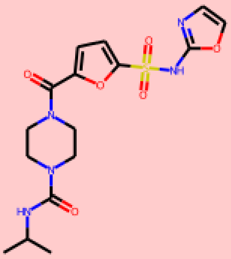
fail

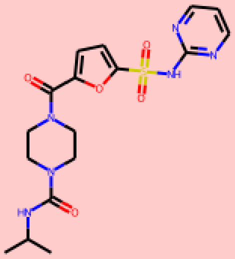
fail

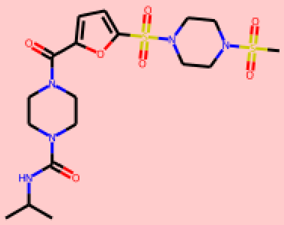
fail

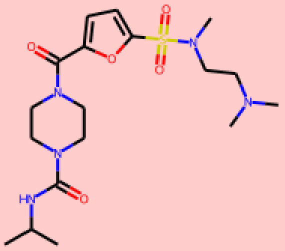
fail

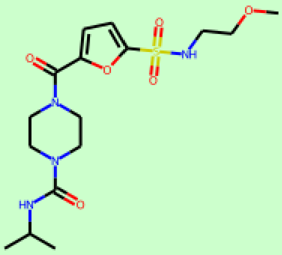
success

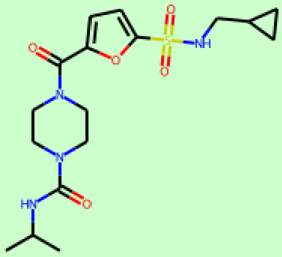
success

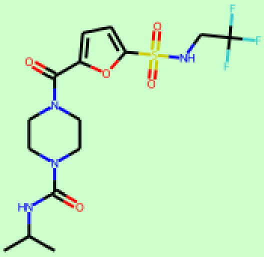
success

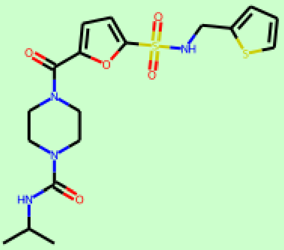
success

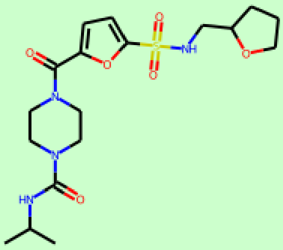
success

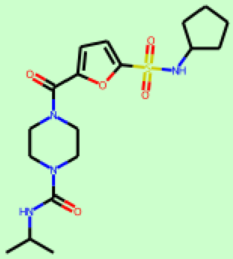
success

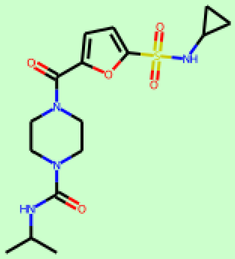
success

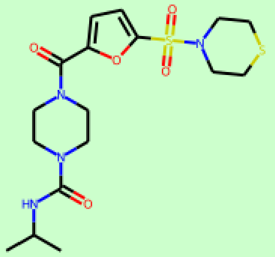
success

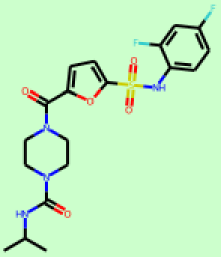
success

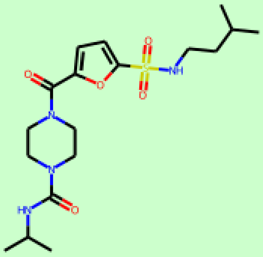
success

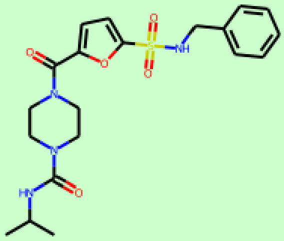
success

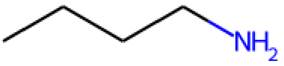
SM

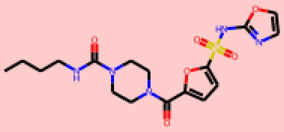
fail

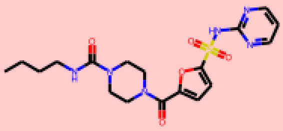
fail

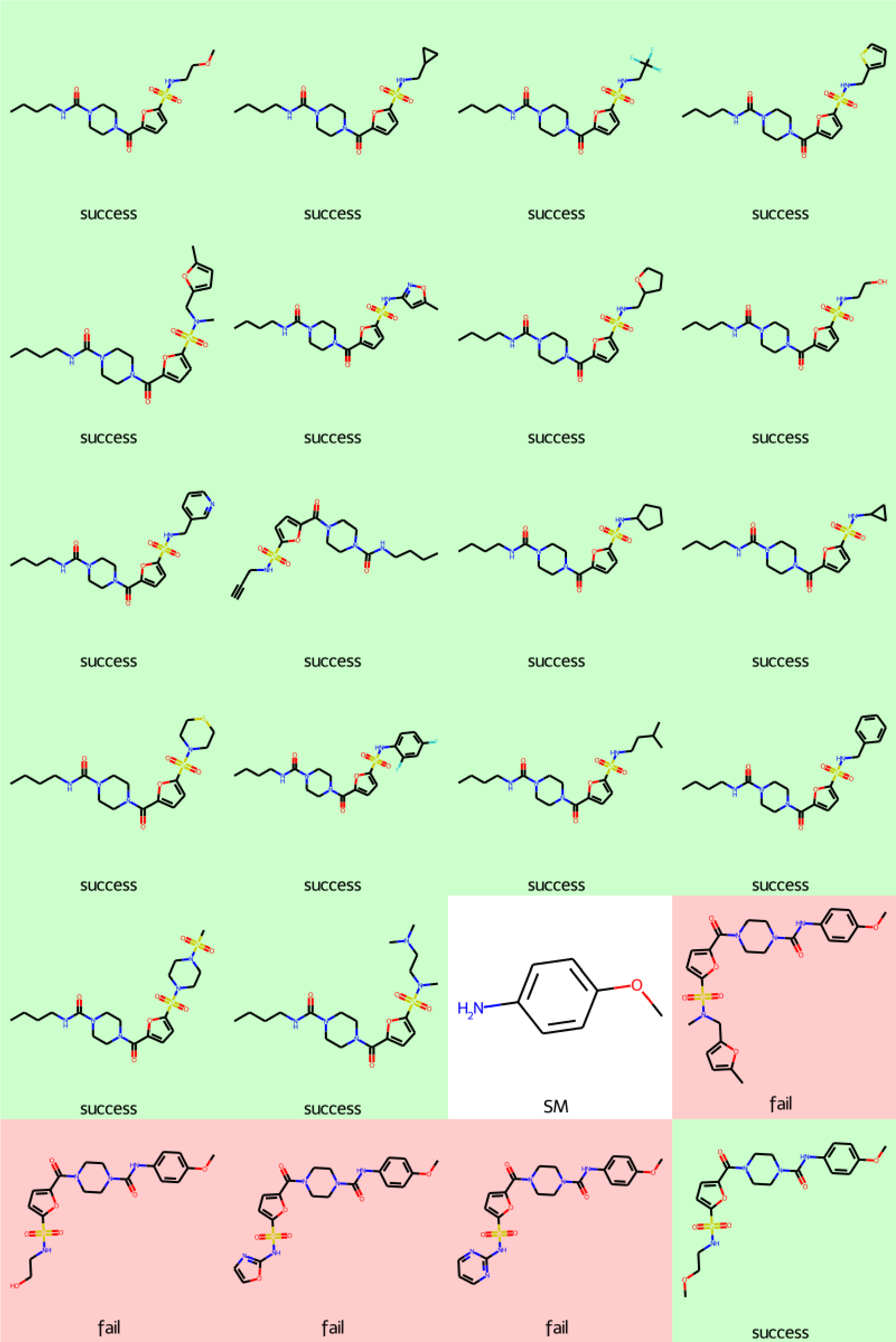
success
success
success
success
success
success
success
success
success
success
success
success
success
success
success
success
success
success
SM
fail
fail
fail
fail
success

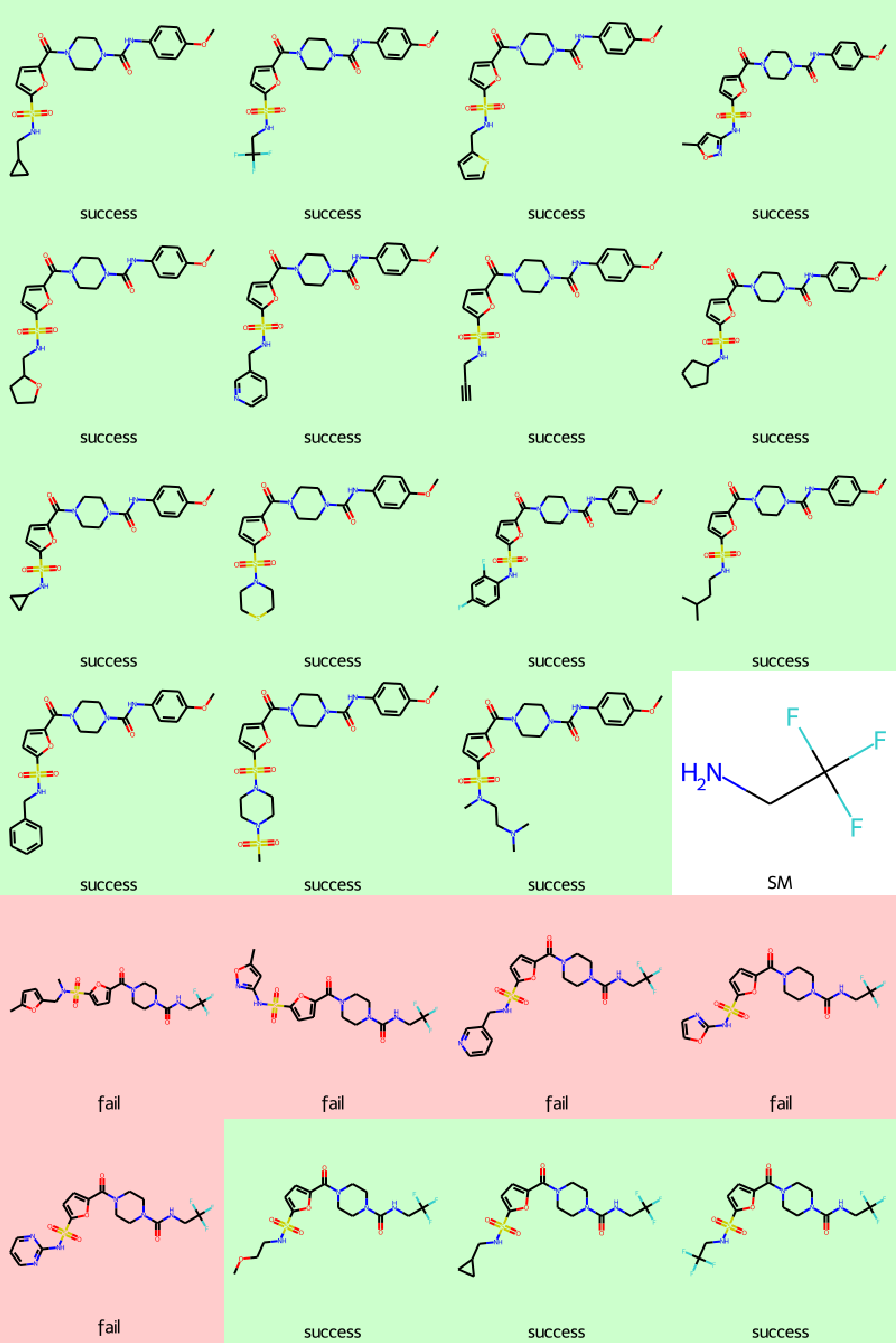
success
success
success
success
success
success
success
success
success
success
success
success
success
success
success
H2N
F
F
F
SM
fail
fail
fail
fail
fail
success
success
success

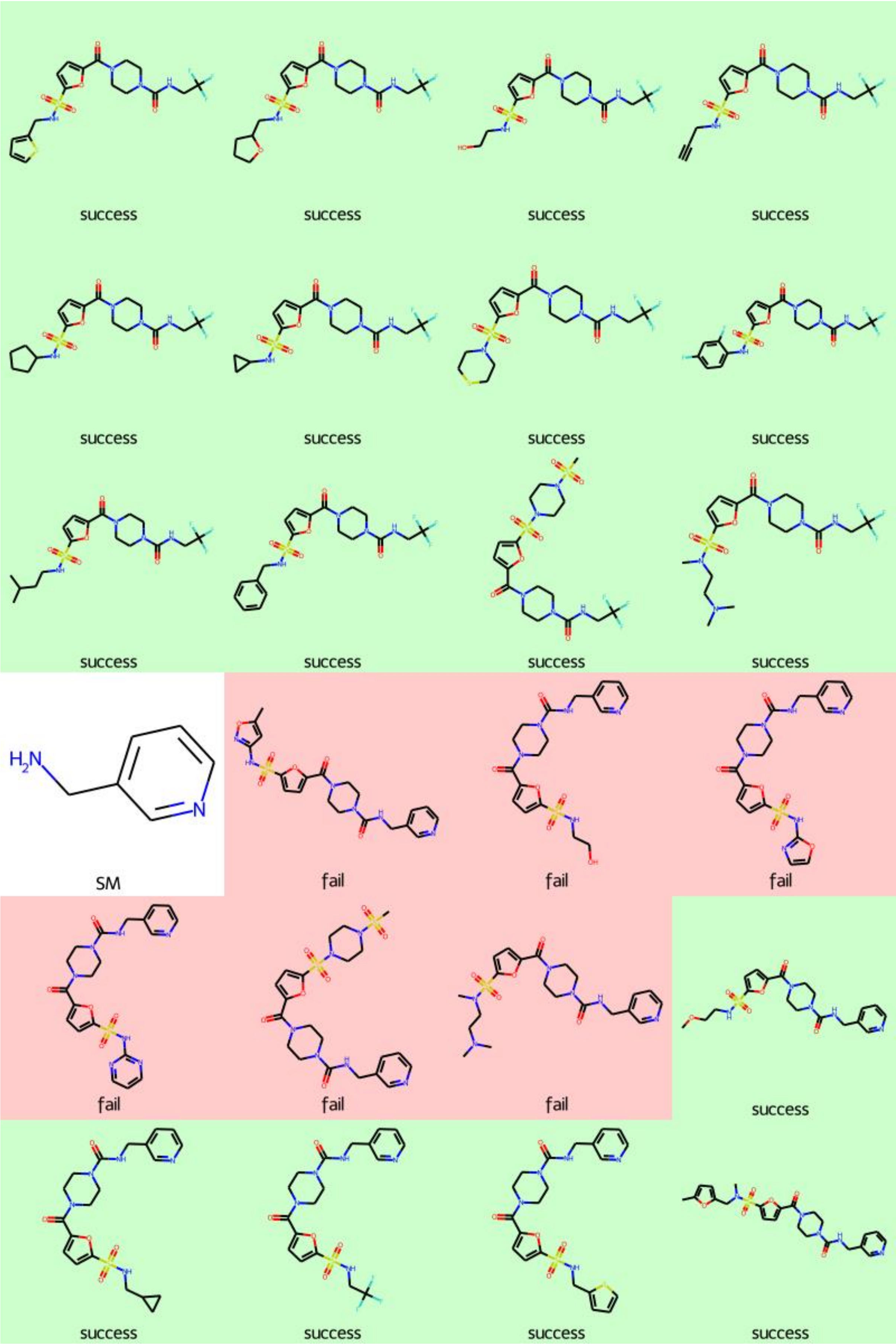
success
success
success
success
success
success
success
success
success
success
success
success
H2N
N
SM
fail
fail
fail
fail
fail
fail
success
success
success
success
success

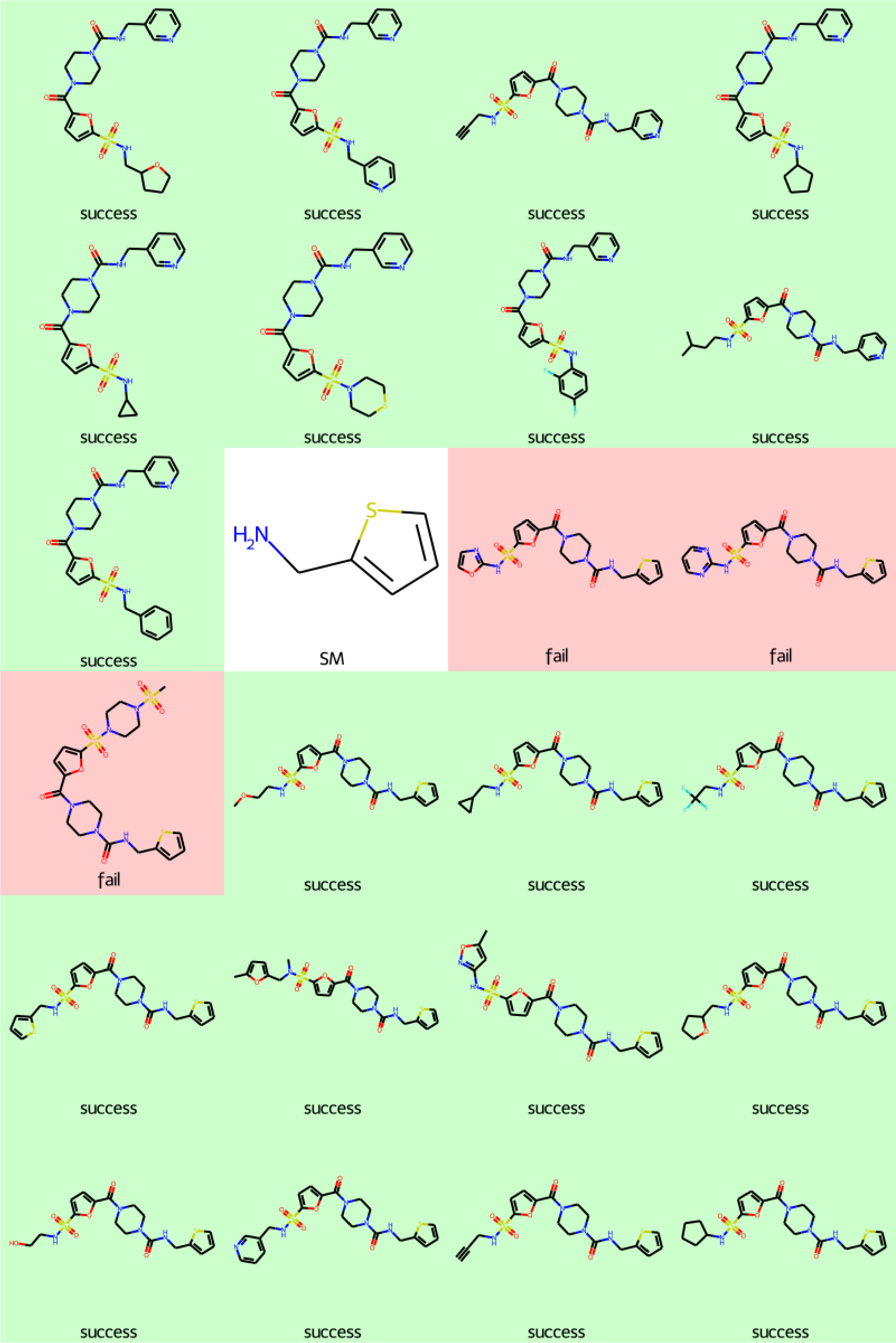
success
success
success
success
success
success
success
success
success
SM
fail
fail
fail
success
success
success
success
success
success
success
success
success
success
success

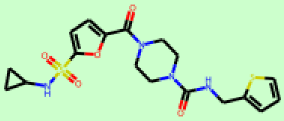
success
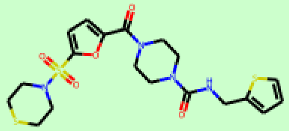
success
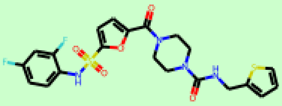
success
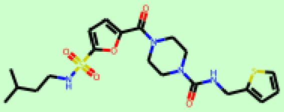
success
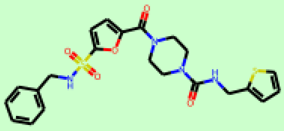
success
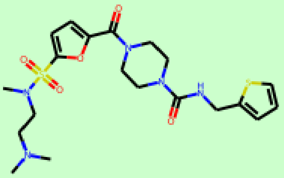
success
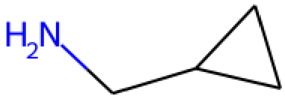

SM
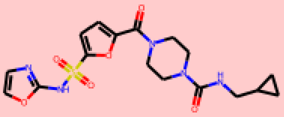
fail
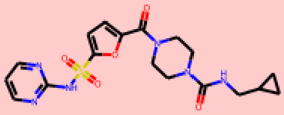
fail
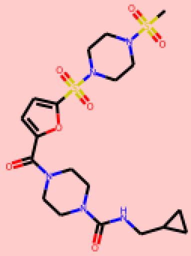
fail
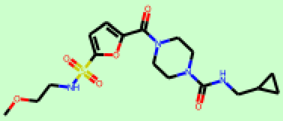
success
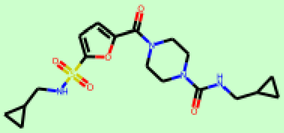
success
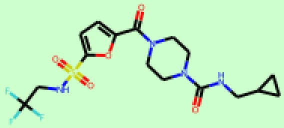
success
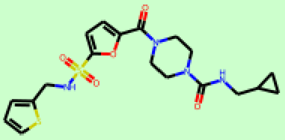
success
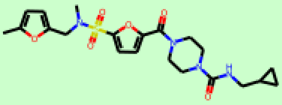
success
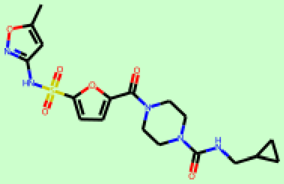
success
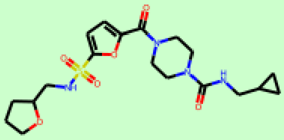
success
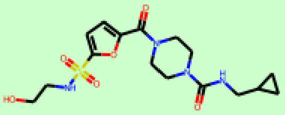
success
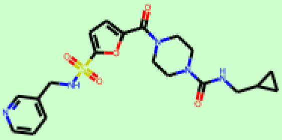
success
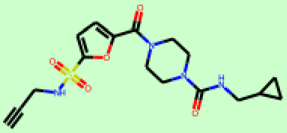
success
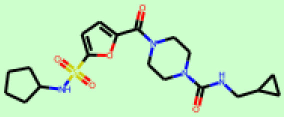
success
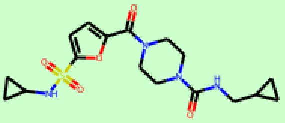
success
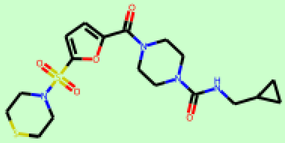
success
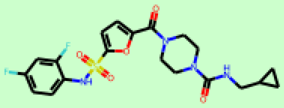
success

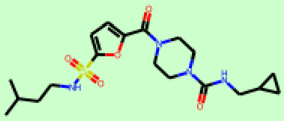

success

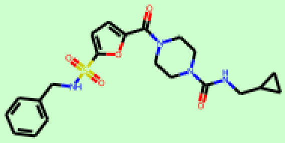

success

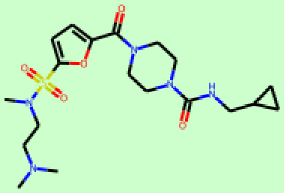

success

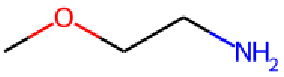

SM

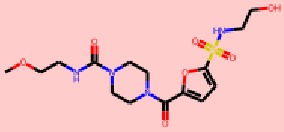

fail

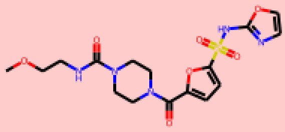

fail

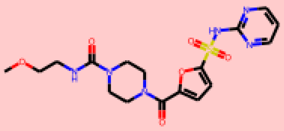

fail

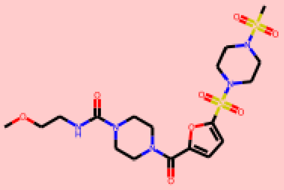

fail

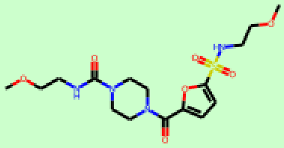

success

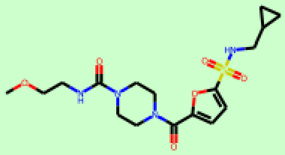

success

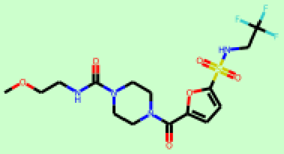

success

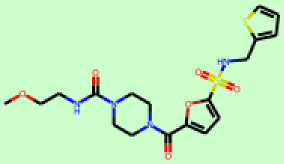

success

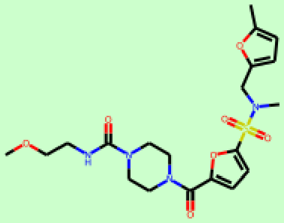

success

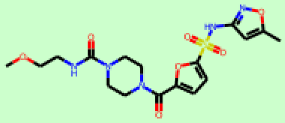

success

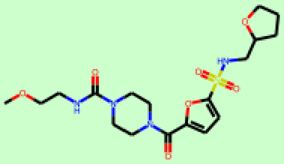

success

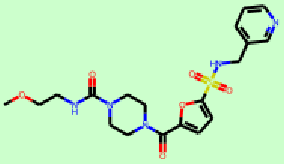

success

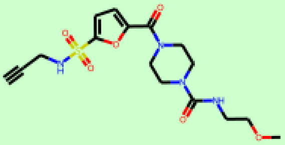

success

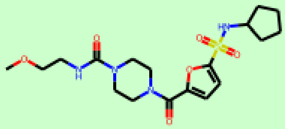

success

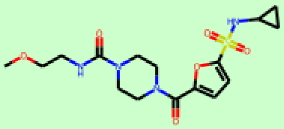

success

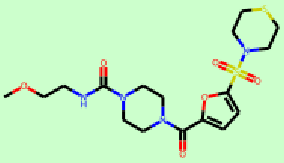

success

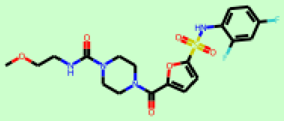

success

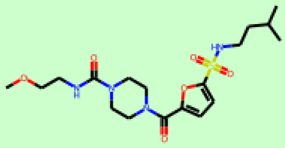

success

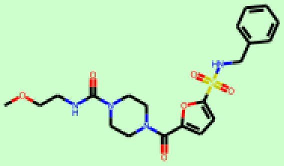

success

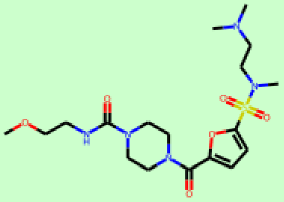

success

9 Spectra

9.1 NMR

NMR spectra for four resynthesized and purified structural hit compounds used for orthogonal biophysics readout.

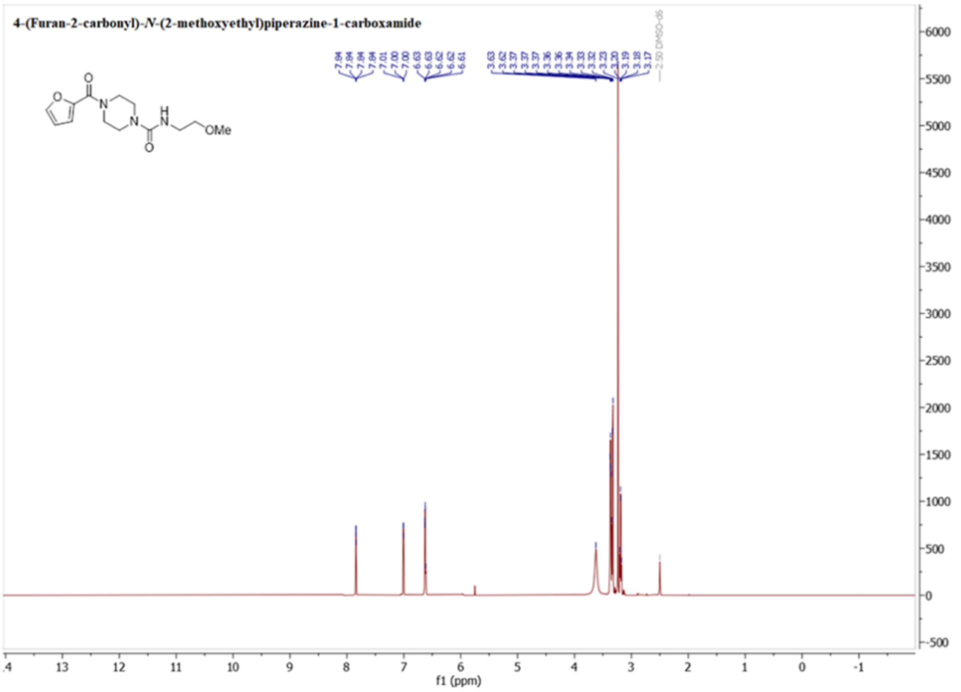

^{1}H NMR (600 MHz, d_6-DMSO) of 4-(furan-2-carbonyl)-*N*-(2-methoxyethyl)piperazine-1-carboxamide.

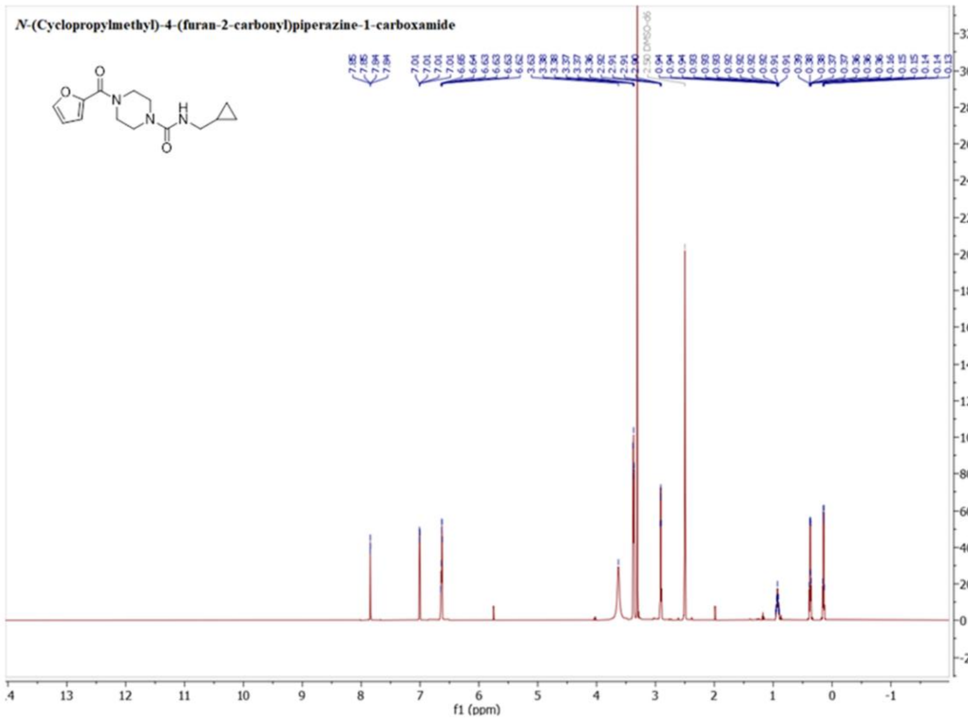

^{1}H NMR (600 MHz, d_6-DMSO) of *N*-(cyclopropylmethyl)-4-(furan-2-carbonyl)piperazine-1-carboxamide.

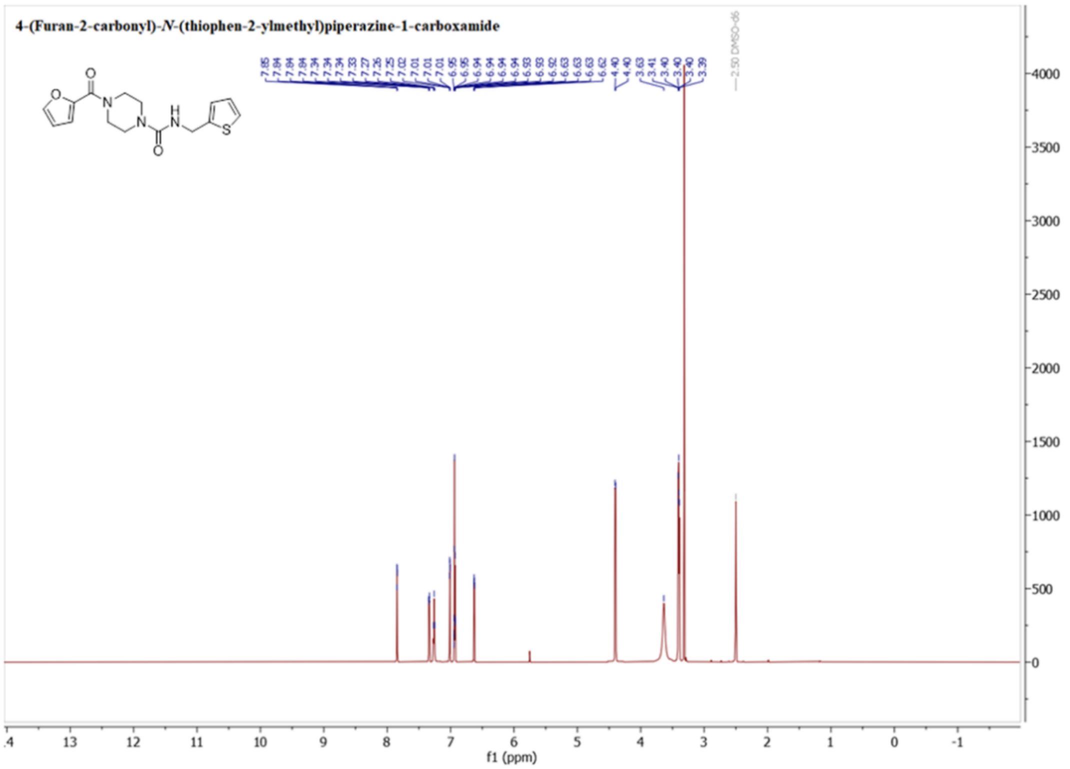

^{1}H NMR (600 MHz, d_6-DMSO) of 4-(furan-2-carbonyl)-*N*-(thiophen-2-ylmethyl)piperazine-1-carboxamide.

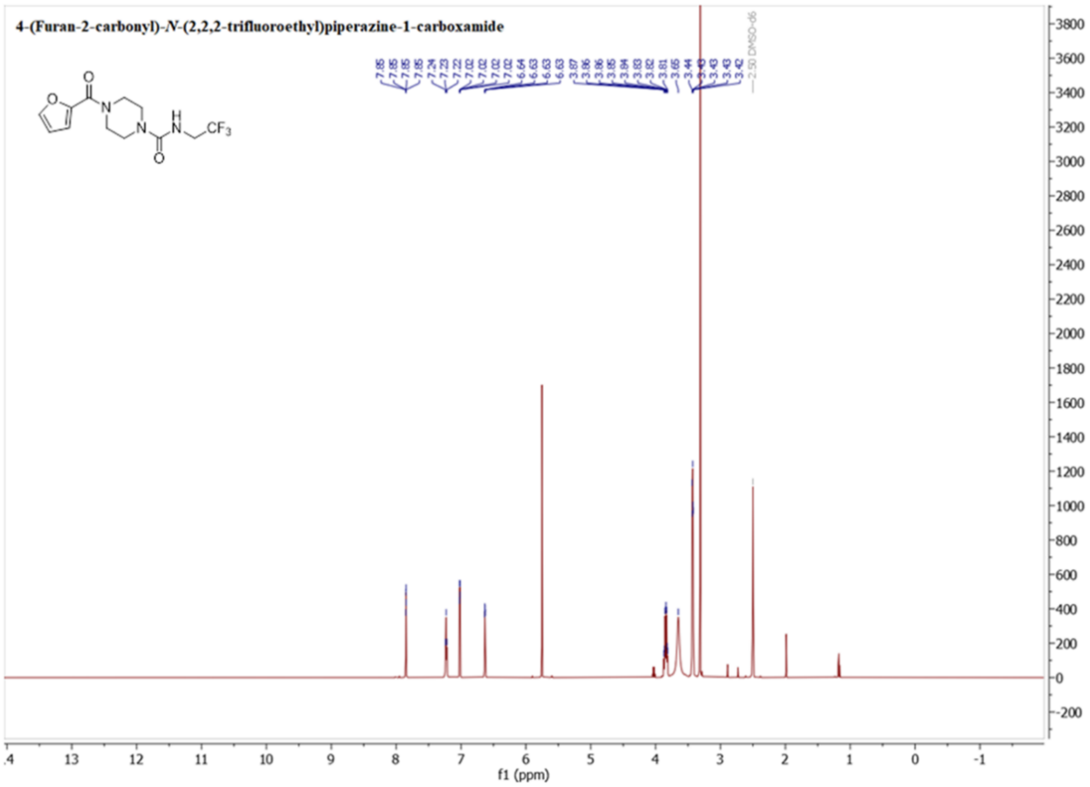

^{1}H NMR (600 MHz, d_6-DMSO) of 4-(furan-2-carbonyl)-*N*-(2,2,2-trifluoroethyl)piperazine-1-carboxamide.

10 2D-structures of reaction products bound in cocrystals

Lateral binding products

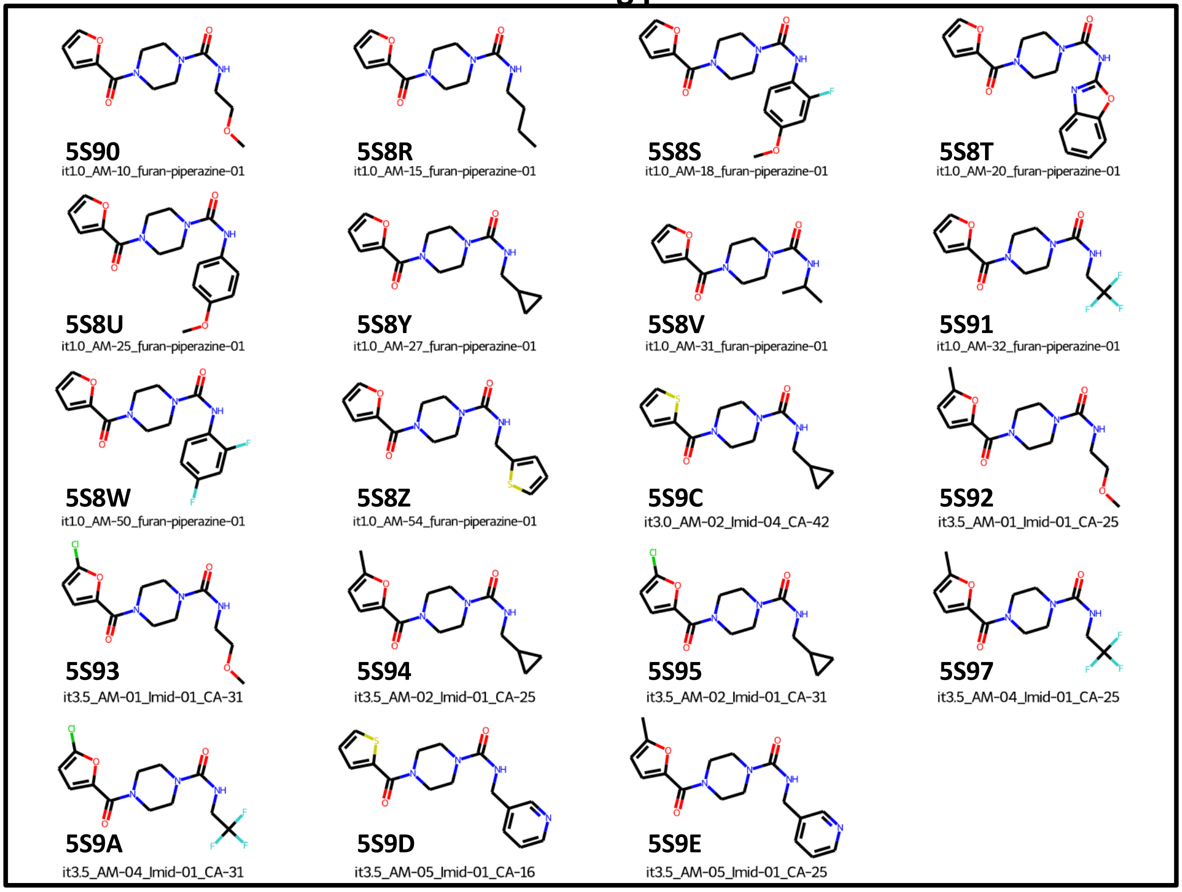

Diving binding products

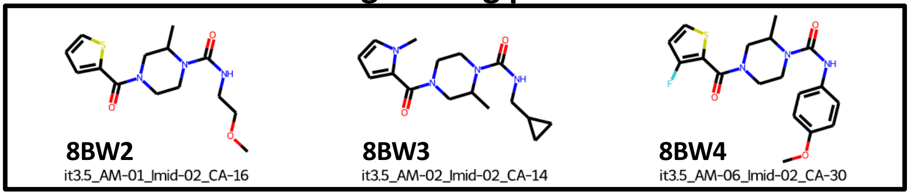

11 3D-structures of compounds bound in cocrystals

11.1 Starting fragment and diving reaction products

Starting fragment

5RKI

F709

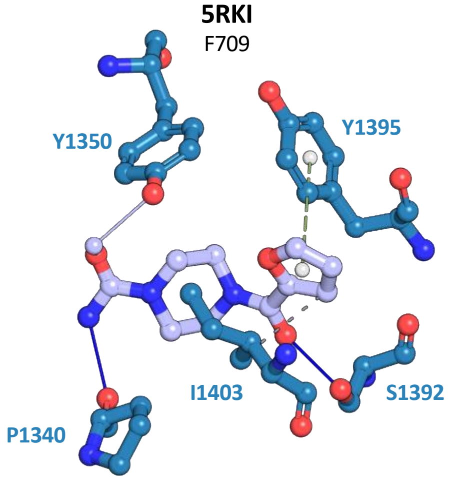

Diving binding products

8BW2

It3.5_AM-01_Imid-02_CA-16

8BW3

It3.5_AM-02_Imid-02_CA-14

8BW4

It3.5_AM-06_Imid-02_CA-30

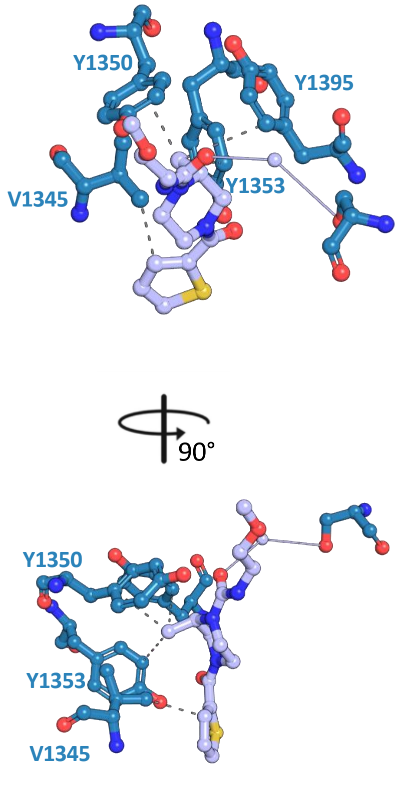

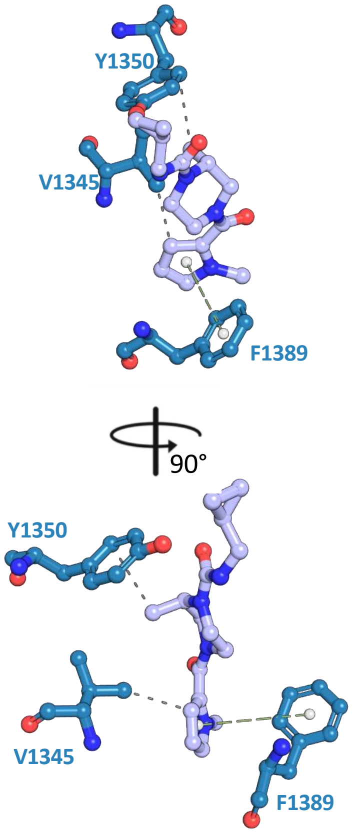

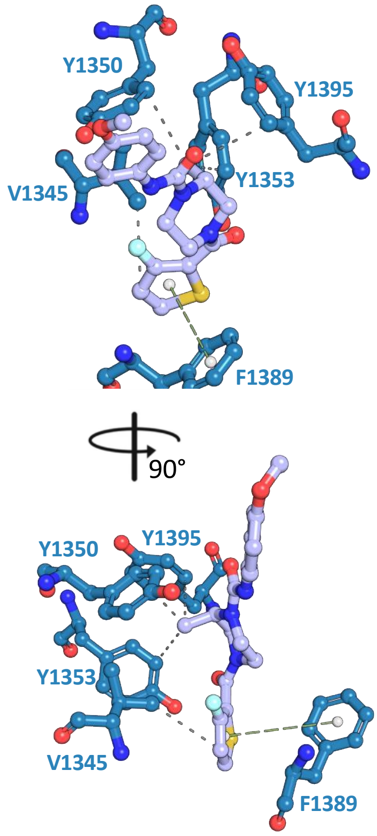

11.2 Laterally bound reaction products

Lateral binding products

5S90
It1.0_AM-10_furane-piperazine-01

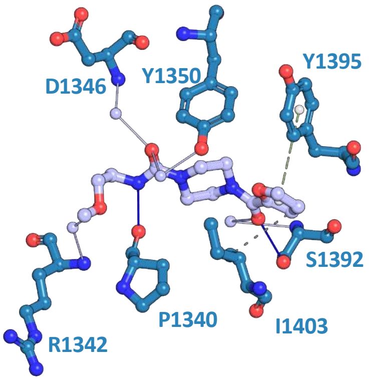

5S8R
It1.0_AM-15_furane-piperazine-01

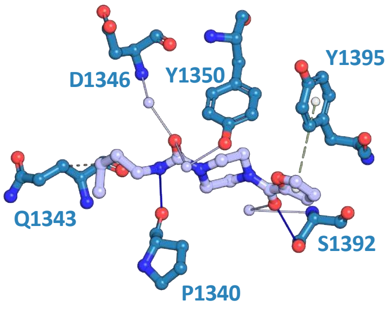

5S8S
It1.0_AM-18_furane-piperazine-01

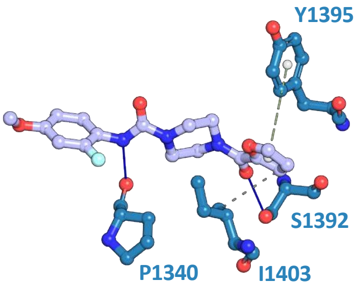

5S8T
It1.0_AM-20_furane-piperazine-01

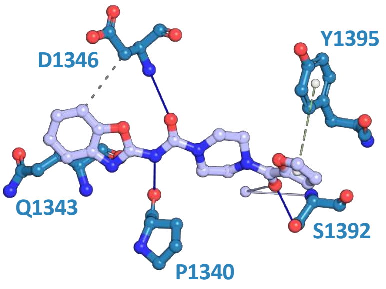

5S8U
It1.0_AM-25_furane-piperazine-01

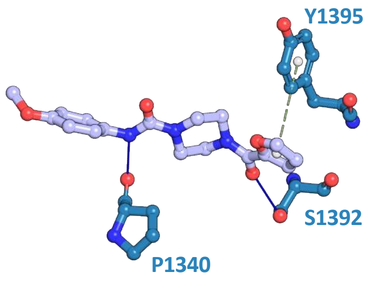

5S8Y
It1.0_AM-27_furane-piperazine-01

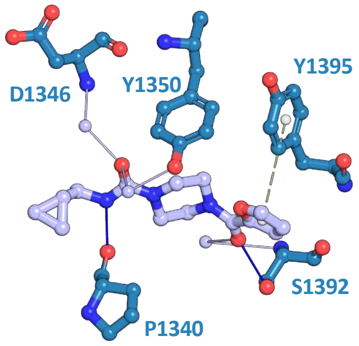

5S8V
It1.0_AM-31_furane-piperazine-01

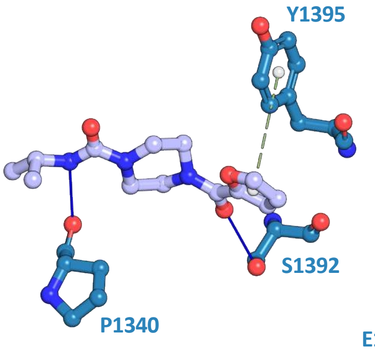

5S91
It1.0_AM-32_furane-piperazine-01

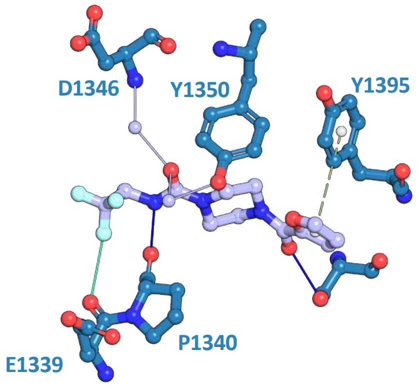

5S8W
It1.0_AM-50_furane-piperazine-01

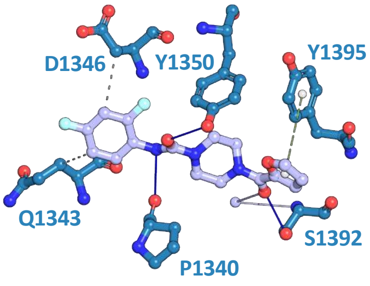

Lateral binding products

5S8Z
It1.0_AM-54_furane-piperazine-01

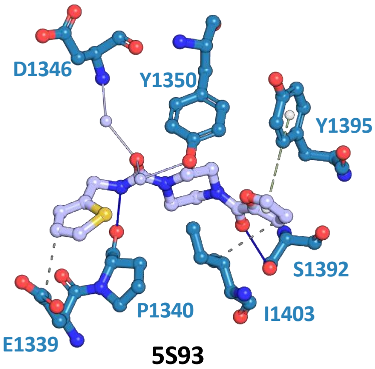

5S9C
It3.0_AM-02_Imid-04_CA-42

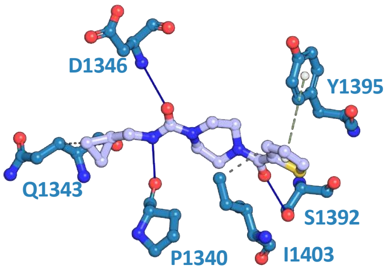

5S92
It3.5_AM-01_Imid-01_CA-25

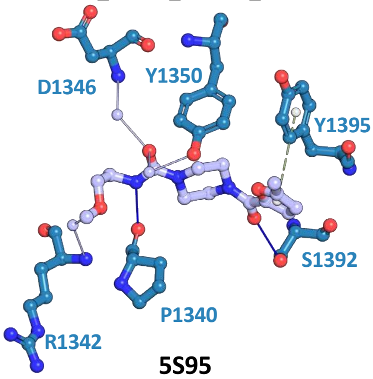

5S93
It3.5_AM-01_Imid-01_CA-31

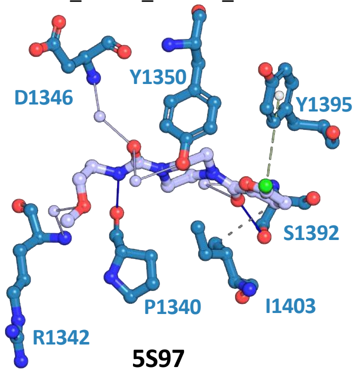

5S94
It3.5_AM-02_Imid-01_CA-25

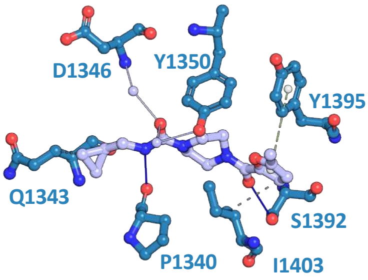

5S95
It3.5_AM-02_Imid-01_CA-31

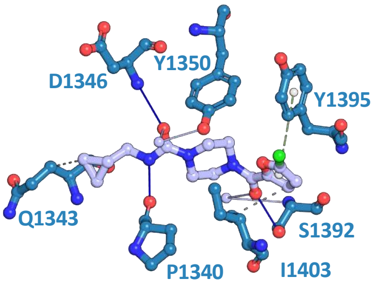

5S97
It3.5_AM-04_Imid-01_CA-25

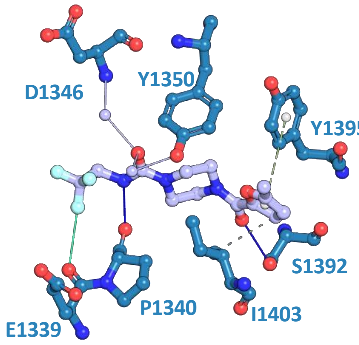

5S9A
It3.5_AM-04_Imid-01_CA-31

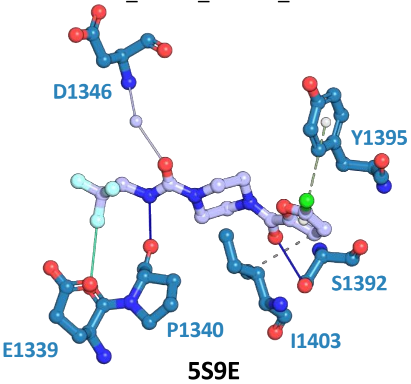

5S9D
It3.5_AM-05_Imid-01_CA-16

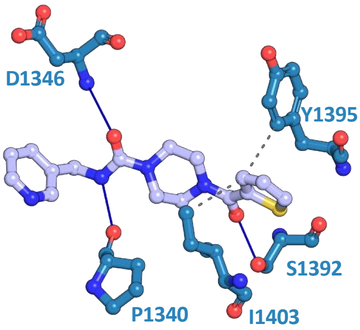

5S9E
It3.5_AM-05_Imid-01_CA-25

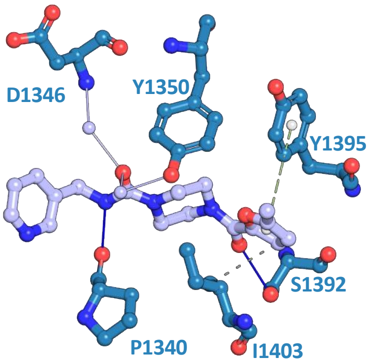

11.3 Starting materials

Starting materials binding

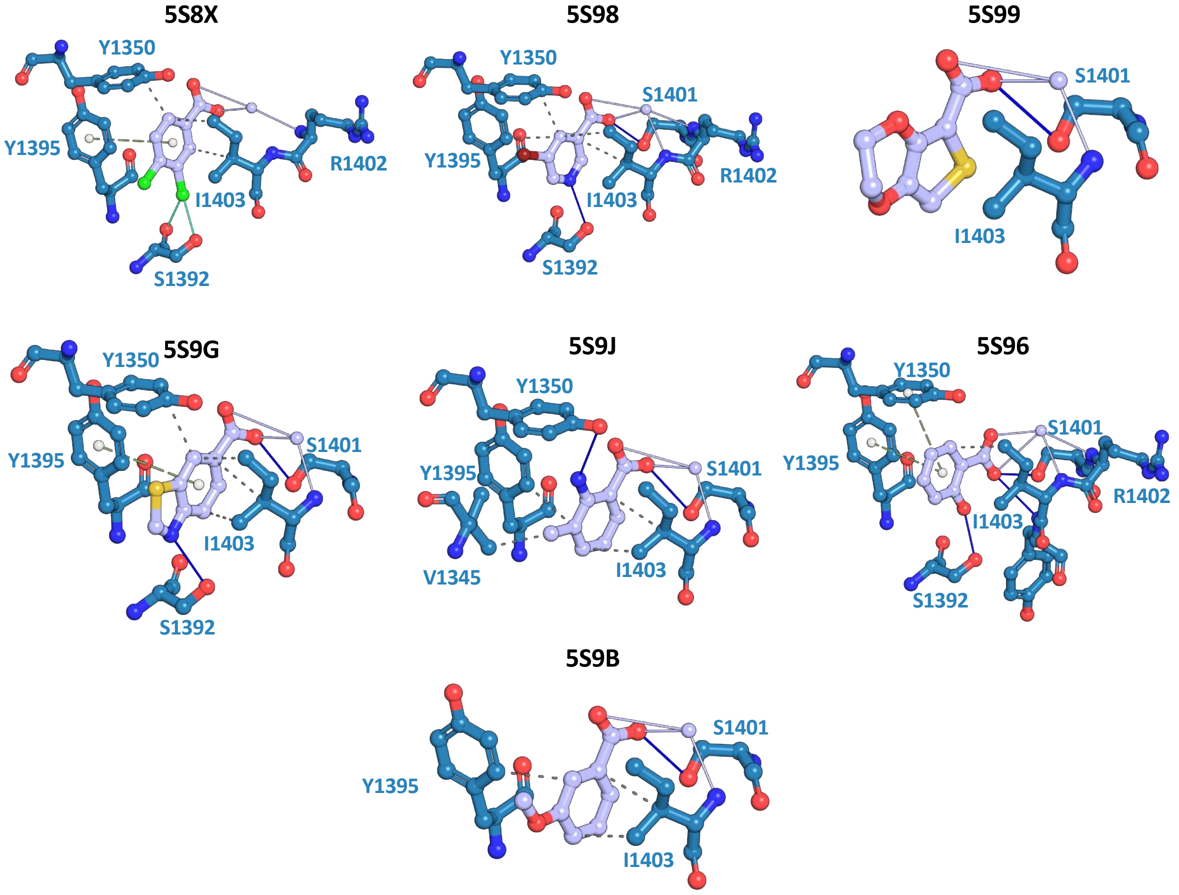

Supplement: Supplementary file 1 — Supporting Information [file ANIE-64-e202424373-s001.pdf]
